# Cysteine‐Specific Multifaceted Bioconjugation of Peptides and Proteins Using 5‐Substituted 1,2,3‐Triazines

**DOI:** 10.1002/advs.202308491

**Published:** 2024-03-11

**Authors:** Quan Zuo, Yiping Li, Xuanliang Lai, Guangjun Bao, Lu Chen, Zeyuan He, Xinyi Song, Ruiyao E, Pengxin Wang, Yuntao Shi, Huixin Luo, Wangsheng Sun, Rui Wang

**Affiliations:** ^1^ State Key Laboratory of Bioactive Substance and Function of Natural Medicines Institute of Materia Medica Chinese Academy of Medical Sciences and Peking Union Medical College Xian Nong Tan Street Beijing 100050 P. R. China; ^2^ Key Laboratory of Preclinical Study for New Drugs of Gansu Province School of Basic Medical Sciences and Research Unit of Peptide Science Chinese Academy of Medical Sciences Lanzhou University 199 West Donggang Road Lanzhou Gansu 730000 P. R. China

**Keywords:** 1,2,3‐triazines, cysteine, late‐stage functionalization, macrocycles, peptides, proteins

## Abstract

Peptide and protein postmodification have gained significant attention due to their extensive impact on biomolecule engineering and drug discovery, of which cysteine‐specific modification strategies are prominent due to their inherent nucleophilicity and low abundance. Herein, the study introduces a novel approach utilizing multifunctional 5‐substituted 1,2,3‐triazine derivatives to achieve multifaceted bioconjugation targeting cysteine‐containing peptides and proteins. On the one hand, this represents an inaugural instance of employing 1,2,3‐triazine in biomolecular‐specific modification within a physiological solution. On the other hand, as a powerful combination of precision modification and biorthogonality, this strategy allows for the one‐pot dual‐orthogonal functionalization of biomolecules utilizing the aldehyde group generated simultaneously. 1,2,3‐Triazine derivatives with diverse functional groups allow conjugation to peptides or proteins, while bi‐triazines enable peptide cyclization and dimerization. The examination of the stability of bi‐triazines revealed their potential for reversible peptide modification. This work establishes a comprehensive platform for identifying cysteine‐selective modifications, providing new avenues for peptide‐based drug development, protein bioconjugation, and chemical biology research.

## Graphical abstract

1,2,3‐Triazines are used to modify biological molecules containing a thiol group in an aqueous solution for the first time, and the resulting aldehyde group served as a handle for secondary labeling. The innovative strategy is applied to double biorthogonal functionalization, secondary labeling of peptides and proteins, and cyclization or dimerization of peptides, highlighting its advantages.

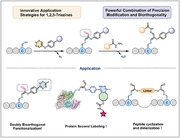

## 1 Introduction

**Figure 1:**
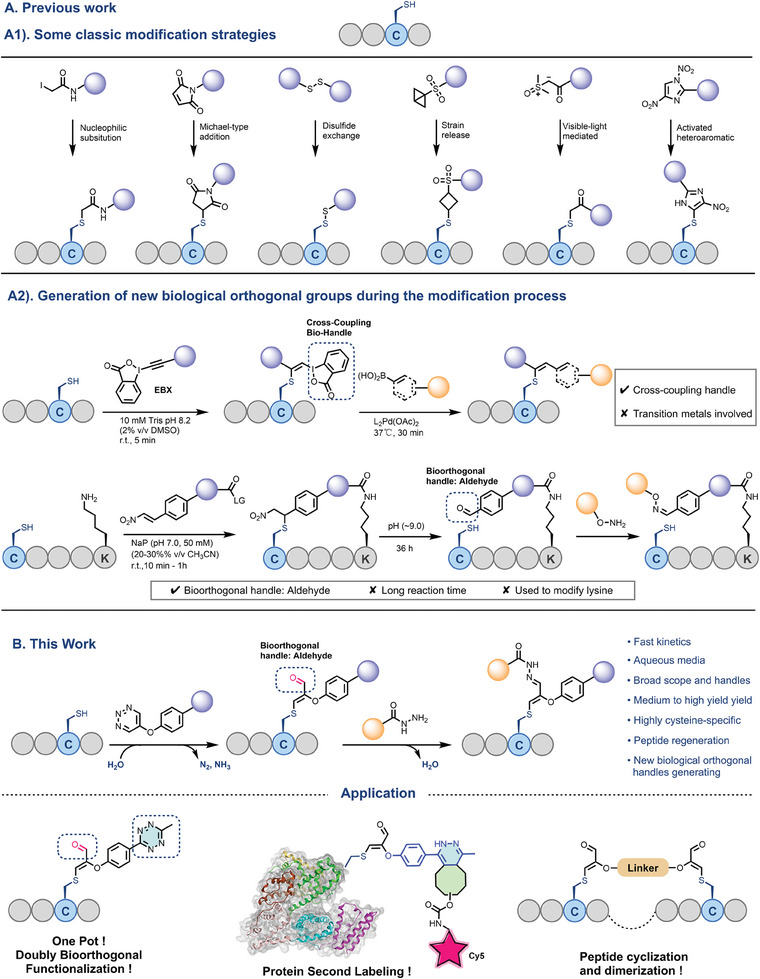
Late‐stage modification of cysteine‐containing peptides. A1) Classic cysteine modification strategies. A2) Previous multifaceted bioconjugation targeting cysteine residues. B) This work: Multifaceted bioconjugation targeting cysteine‐containing peptides and proteins using multifunctional 5‐substituted 1,2,3‐triazines.

Precise postchemical modification of peptides and/or proteins has become a hot topic in the nexus of biomolecule engineering, bioorthogonal chemistry, and drug discovery, leading to the generation of peptide drugs, antibody‒drug conjugates, and molecular imaging probes over the past two decades.^[^ ^1^ ^]^ To date, multiple strategies for directly targeting natural amino acids have been developed that^[^ ^2^ ^]^ rely on the introduction of chemical modifications at a single and distinct site on native peptides to form homogeneous and well‐defined bioconjugates. Among these methods, diverse chemical strategies and coupling reagents provide rich solutions for accessing cysteine (Cys)‐specific peptides and/or protein modifications due to the inherent strong nucleophilicity, low redox potential, and low abundance of Cys compared to other classic amino acids.^[^ ^3^ ^]^ In addition to classic strategies, including nucleophilic substitution (iodoacetamides),^[^ ^4^ ^]^ S‐Michael‐type addition (maleimide derivatives),^[^ ^5^ ^]^ and disulfide exchange reactions,^[^ ^6^ ^]^ there are also some innovative methods reported and applied, such as reactions with activated heteroaromatic compounds,^[^ ^7^ ^]^ hypervalent iodine reagents,^[^ ^8^ ^]^ cationic activation reagents,^[^ ^9^ ^]^ strain‐releasing reagents,^[^ ^10^ ^]^ and reactions via photocatalysis^[^ ^11^ ^]^ (**Figure** **1**A1). Despite these advances, there is a continued demand for methods that are simple, efficient, and “cost‐effective” and exhibit high atomic utilization.^[^ ^2a^ ^]^

With the ability to generate new orthogonally manoeuvrable functional groups for secondary labeling during the functionalization process, the potential for being “cost‐effective” becomes apparent. To our knowledge, there are currently several cases similar to this concept (Figure 1A2). The Waser group, for instance, utilized the reaction between ethynylbenziodoxolones (EBXs) and cysteine to yield stable vinylbenziodoxolone hypervalent iodine conjugates, which enabled the subsequent “orthogonal” functionalization of bioconjugates through Suzuki‐Miyaura cross‐coupling of vinyl hypervalent iodines that necessitated the involvement of heavy metals.^[^ ^12^ ^]^ Rai's design and synthesis of the LDM_C‐K_ reagent provided an orthogonally functional group via C─S bond formation and dissociation, which achieved the labeling of lysine instead of cysteine.^[^ ^13^ ^]^ Despite some remaining limitations, the innovative reaction pathways and versatile bioorthogonal applications of these methods have offered valuable inspiration. From this perspective, a cysteine‐specific modification strategy that generates new bioorthogonal groups during functionalization, boasting advantages such as the absence of transition metals, rapid reaction kinetics, and high chemo‐selectivity under mild and biocompatible conditions, remains to be developed.

1,2,3‐Triazines (*v*‐triazines) possess a unique structure comprising three contiguous nitrogen atoms within a six‐membered aromatic heterocycle, conferring upon them strong electrophilic properties.^[^ ^14^ ^]^ In comparison to the other regional isomers of triazines, including 1,2,4‐triazine and 1,3,5‐triazine, 1,2,3‐triazine has received comparatively less attention, particularly concerning biomolecule labeling within biological systems.^[^ ^15^ ^]^ Encouragingly, the reactions of 1,2,3‐triazine with primary amines, secondary amines, and thiols^[^ ^16^ ^]^ attracted our attention, especially since their products all carry an aldehyde group, which can be used as a powerful bioorthogonal handle for secondary labeling. Consequently, we focused on the less explored realm of 1,2,3‐triazines for the precise modification of peptides and proteins by exploiting the nucleophilicity of cysteine. However, there are three challenging issues to be addressed in this scenario. First, due to the instability of 1,2,3‐triazines, derivatives with complex functional groups have not yet been reported, so ensuring the stability and availability of products while diversifying their structures is key.^[^ ^15a^ ^]^ Second, determining how the reactivity of 1,2,3‐triazines with amines and thiols determines the chemoselectivity of peptide modification is still challenging. Third, balancing reactivity and hydrolysis via the use of 1,2,3‐triazine in the aqueous phase for the functionalization of water‐soluble biomolecules is difficult.

Taking all these aspects into consideration, and as part of our ongoing research interests in peptide chemistry,^[^ ^17^ ^]^ a general and novel platform was developed that utilizes the specific reaction of cysteine and 1,2,3‐triazine derivatives to achieve the late functionalization of peptides, peptide cyclization, and dimerization, as well as the secondary labeling of proteins (Figure 1B; see Section S5.2, Supporting Information, for details on the mechanism). Furthermore, we demonstrate that cost‐effective “one‐modification, doubly orthogonal labeling” can be achieved by utilizing the aldehyde group generated in this reaction. In addition, stability experiments demonstrated that the modified peptide exhibited regenerative capacity under high thiol conditions, thus providing a reversible modification strategy.

## 2 Results and Discussion

### 2.1 Reaction Optimization

With the objective of linking diverse functional groups and enhancing electrophilicity to expedite the modification process,^[^ ^18^ ^]^ we initially crafted **Tz‐1** (Figure S1, Supporting Information), which features an amide bond substitution at position 5. Then, glutathione (GSH, **1a**) was chosen as the model peptide to initiate our screening process. As anticipated, **Tz‐1** exhibited an exceedingly rapid reaction rate with GSH, and the nitrogen byproduct formed during the reaction was clearly observable throughout the entire reaction duration. Optimizing the solution pH to mildly acidic (pH 6.5) yielded the target product **s1** in a notably high yield, concurrently leading to a substantial conversion rate (Table S1, Supporting Information). However, efforts to modify other intricate peptides using **Tz‐1** have resulted in challenges in achieving singular‐site modification. This difficulty was likely attributed to the high electrophilicity and inherent instability of **Tz‐1**, which render it prone to reacting with other nucleophilic amino acids. These outcomes inspired us to search for 1,2,3‐triazine derivatives capable of striking an equilibrium between selectivity and reactivity by changing the substituent at position 5. 1,2,3‐Triazine derivatives featuring phenol substituents at position 5 were favored due to their efficient synthetic accessibility and appropriate electrophilic properties.^[^ ^15a^ ^]^ In this context, we conducted a comprehensive array of condition screenings utilizing **2a** (**Table** **1**).

**Table 1:** Optimization of reaction conditions.

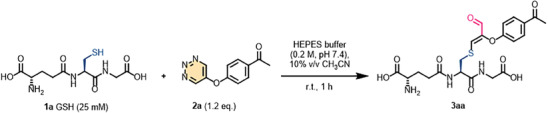		
Entry^a)^	Deviation from above conditions	Yield^b)^ [%]
1	None	98 (80.6^c)^)
2	Bis‐Tris buffer (pH 7.4)	94
3	PB buffer (pH 7.4)	95
4	PBS buffer (pH 7.4)	97
5	Tris‐HCl buffer (pH 7.4)	21
6	PIPES buffer (pH 7.4)	95
7	Mix 10 min	71
8	Mix 30 min	88
9	Concentration of GSH: 10 mm	94
10	Concentration of GSH: 100 mm	94
11	HEPES buffer (pH 8)	96
12	HEPES buffer (pH 9)	91
13	HEPES buffer (pH 6.5)	24
14	PIPES buffer (pH 5.5)	22
15	H_2_O^d)^	14
16	HEPES buffer (pH 7.0)	98
17	Add peptide (H‐RLAYSHKWD‐NH_2_)^e)^	90

^a)^ Standard reaction conditions (entry 1): Add **1a** (GSH, 25 mm) and **2a** (1.2 eq) in 0.2 m HEPES buffer at room temperature and mix for 1 h;

^b)^ Determined by the peak area of the HPLC chart (280 nm) using coumarin as an internal standard;

^c)^ Isolated yield by RP‐HPLC;

^d)^ The reaction solution was acidic (≈3);

^e)^ Add **1a**, **2a** (1.2 eq) and an equimolar peptide (H‐RLAYSHKWD‐NH_2_) to **1a** in 0.2 m buffer at room temperature.

Standard reaction conditions were established upon exploring a range of reaction parameters. We found that using **1a** (25 mm) and **2a** (1.2 equiv.) in HEPES buffer (0.2 m, pH 7.4) at room temperature under air for 1 h provided the best results, giving the GSH‐modified product **3aa** in 98% HPLC yield and 81% isolated yield (Table 1, entry 1). When considering alterations to the buffer system (Table 1, entries 2–6), excluding the Tris‐HCl buffer, the majority of reactions displayed excellent results, and the HPLC yields were above 90%. Reducing the reaction time (Table 1, entries 7–8) to as low as 10 min can also result in a yield of 71%, indicating exceptional reaction efficacy. Variations in substrate concentration had minimal influence on the yield (Table 1, entries 9–10). To comprehensively examine the impact of pH on the reaction, we subjected the buffers to varying pH values (Table 1, entries 11–16). The results showed that the yield was largely unaffected in neutral to weakly alkaline buffer solutions; however, under weakly acidic conditions, the reaction yield was significantly decreased. Intriguingly, altering the buffer to pure water (Table 1, entry 15) led to a reaction yield as low as 14%, highlighting the effectiveness of the buffer system in this reaction. As demonstrated in entry 17, GSH and an equimolar peptide (H‐RLAYSHKWD‐NH2) were added to the reaction system to verify cysteine selectivity. The results indicated that when cysteine and other active or nucleophilic amino acids coexist, Compound **2a** can selectively modify cysteine with precision.

### 2.2 Peptide Scope of Cys‐Selective Modification

**Figure 2:**
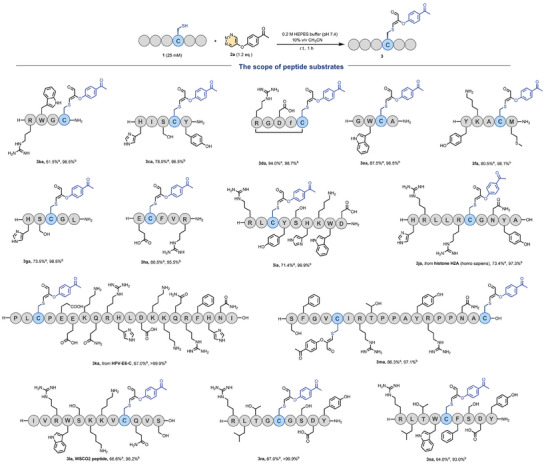
Substrate scope of cysteine‐containing peptides. a) The isolated yield was determined. b) The purity was determined based on the HPLC‐UV ratio.

With the optimized conditions in hand, we investigated the scope of peptides containing cysteine residues (**Figure** **2**). Initially, short peptide substrates consisting of 4 to 5 distinct functional amino acids were examined. The results indicated that peptides featuring nucleophilic amino acids such as Lys (**3fa**), Arg (**3ba**, **3** **ha**), Glu (**3** **ha**), or His (**3ca, 3ga**) exhibited good tolerance. Moreover, the cyclic peptide **3** **da** was generated in an impressive yield of 94%. We also assessed the modification reactivity of **2a** with longer biologically relevant peptides (10 to 20 amino acids), including **1j** (a 10‐mer sequence representing residues 32–41 of histone H2A in *Homo sapiens* ^[^ ^19^ ^]^), **1k** (the HPV‐E6‐C peptide, a 20‐mer sequence of the human papillomavirus E6 protein C‐terminal domain^[^ ^20^ ^]^), and **1l** (the WSCO2 peptide, an endogenous peptide inhibitor of the chemokine CXCR4 receptor^[^ ^21^ ^]^). As a result, these assessments yielded the desired cysteine‐modified products **3ja**, **3ka**, and **3la** in outstanding yields. Additionally, the peptide containing 18 amino acids and featuring two cysteine residues yielded the double‐modified product **3ma** with exceptional efficiency. Peptides **1r** and **1s** were used to study the impact of peptide hindrance on the reaction; these peptides were completely transformed after 30 and 60 min, generating **3ra** (67% yield) and **3sa** (64% yield), respectively. The results showed that the hindrance of peptide had almost no effect on the yield of the reaction, although it had a slight effect on the rate of reaction.

**Figure 3:**
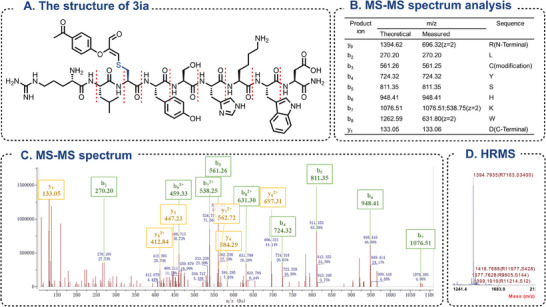
Tandem MS analysis of conjugate **3ia**. A) The structure of **3ia**. B) MS‐MS spectrum analysis. C) MS‐MS spectrum. D) Q‐TOF‐HRMS spectrum, *m*/*z*: calcd for C_65_H_87_N_17_O_16_S^+^ 1394.6315, found 1394.6333.

Subsequently, we introduced a mutation that replaced the alanine in H‐RLAYSHKWD‐NH2 with cysteine, resulting in the formation of **1i**, which displayed efficient reactivity with **2a**, yielding a remarkable 71.4% yield. The purified product **3ia** was confirmed through Q‐TOF HRMS and tandem mass spectrometry analysis, as depicted in **Figure** **3**. The accompanying figure reveals that only cysteine underwent a reaction with **2a**, yielding the modified product. This observation provided additional evidence for the specific cysteine‐modifying ability of 1,2,3‐triazine derivatives.

### 2.3 Modification of Peptides with Functionalized 1,2,3‐Triazines

**Figure 4:**
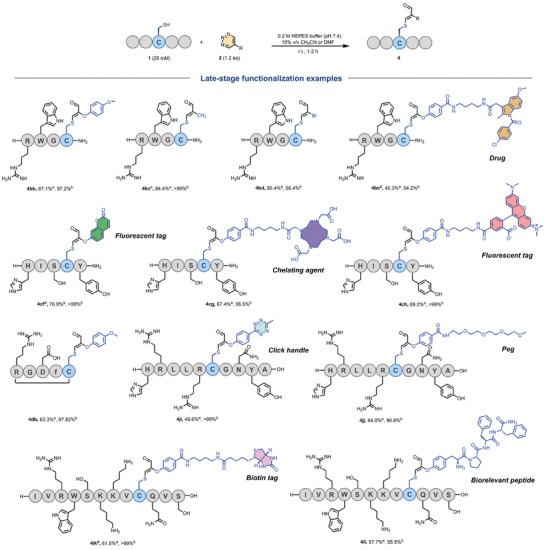
Modification of peptides with functionalized 1,2,3‐triazines. Reaction conditions: peptide (25 mm), 1,2,3‐triazine (1.2 equiv) in HEPES buffer (0.2 m, pH 7.4, 10% v/v CH_3_CN) at room temperature for 1–2 h. a) Isolated yield. b) HPLC purity. c) React for 2 h d) 10% v/v DMF.

Having achieved favorable reaction efficiencies across diverse cysteine‐containing peptides, we embarked on a deeper investigation into the potential for functionalized 1,2,3‐triazines (**Figure** **4**). Triazine compounds featuring distinct functional units were synthesized and subjected to reactions with both short and elongated peptides, yielding a diverse array of peptide conjugates. For instance, Compound **2b**, through substitution of the acetyl group on the benzene ring with a methoxy group, exhibited seamless reactivity with **1b** and **1d**, resulting in modified peptide products (**4bb** and **4db**). Shifting the 5‐position substituent from phenol to methyl (**2c**) and activating the bromine substitution (**2d**) facilitated effective reactions with **1b** within 1–2 h, yielding target Compounds **4bc** and **4bd**.

Peptide‒drug conjugates (PDCs) and radionuclide–drug conjugates (RDCs) have garnered increasing attention as innovative therapeutic approaches for amalgamating peptides and chemotherapeutics.^[^ ^22^ ^]^ Illustratively, **4be** and **4cg** are prime examples of peptides coupled with drug molecules and metal chelators. In addition, an array of widely used biorelevant groups, encompassing fluorescent dyes such as coumarin and 5‐carboxy‐tetramethylrhodamine (5‐TAMRA), polyethylene glycol (PEG) polymers, and affinity labels (biotin), could be incorporated into 1,2,3‐triazine reagents, yielding corresponding products with robust isolated yields (**4cf**, **4ch**, **4jj**, **4lk**). Notably, **2i**, a hybrid of 1,2,3‐triazine and tetrazine, was synthesized, in which the bioorthogonal group tetrazine^[^ ^23^ ^]^ could be introduced into peptides via the reaction of 1,2,3‐triazine with cysteine, such as **4ji**. Furthermore, we extended this strategy to accommodate cysteine‐containing and tyrosine‐containing peptide conjugation, thus expanding the range of available substrates. Endomorphin‐2,^[^ ^24^ ^]^ a highly selective high‐affinity µ‐opioid receptor agonist, was reacted with the WSCO2 peptide (**1l**) to obtain **4ll**.

### 2.4 One‐Pot Triple Functionalization of Cys Utilizing the Bioorthogonal Group Generated During the Modification Process

**Figure 5:**
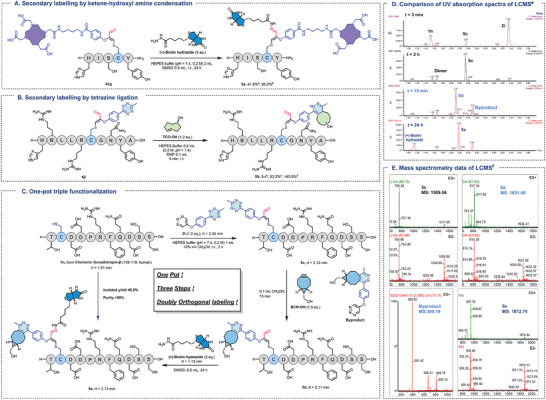
Multifaceted bioconjugation. A). Secondary labeling of **4cg** by ketone‐hydroxyl amine condensation. B). Secondary labeling of **4jj** by tetrazine ligation. C). One‐pot triple functionalization of **1n** to form **5e**. D). Comparison of UV absorption spectra at corresponding times. E). Some of the key mass spectrometry data. a) Isolated yield. b) HPLC purity. c) Mean ratio of the two isomers. d) Average HPLC purity of the two isomers. e) UV absorption area between 190 and 480 nm. f) ESI (electron spray ionization).

The broad peptide scope and versatile handles of this reaction instilled us with confidence, and we proceeded to validate the initial concept of leveraging the generated aldehyde groups for achieving triple functionalization in one pot. Aldehydes serve as ancient biological orthogonal linkages known to react with hydroxylamine or hydrazide under acidic to neutral conditions.^[^ ^25^ ^]^ Consequently, we initiated testing by reacting the conjugate **4cg** with (+)‐biotin hydrazine in HEPES buffer and monitoring the product conversion over 24 h. As illustrated in Figure S2 (Supporting Information), the conversion was nearly complete after 24 h, yielding a separation yield of 41.8% (**Figure** **5**A). Furthermore, we employed **4ji** for secondary labeling via a bioorthogonal tetrazine reaction.^[^ ^23b^ ^]^ Impressively, the reaction reached completion within 5 min, as depicted in Figure 5B. Considering that the products of the reaction between TCO‐OH and tetrazine were isomers, BCN‐OH was selected for the following study to ensure product homogeneity while not affecting the reaction rate.

Based on the above, the compatibility of the Cys‐specific modification with doubly orthogonal labeling was then studied. As shown in Figure 5C, the cysteine residue of **1n** (chorionic gonadotropin‐β, 109–119; human^[^ ^26^ ^]^) was modified efficiently by **2i** to release product **5c**, which contained a tetrazine moiety and a newly formed aldehyde group (Step 1). Then, BCN‐OH and (+)‐biotin hydrazine were sequentially added to the reaction mixture and reacted for 15 min and 24 h, respectively, to obtain triple‐labeled peptide **5e** (Steps 2 and 3). The final product was purified in satisfactory yield (46.0%). Figure 5D,E shows the comparison of UV absorption spectra at different time points and the mass spectrometry data of key compounds. This novel modification strategy opened up new avenues for dual bioorthogonal labeling, indicating the promising application potential of this approach in the field of biochemistry.

### 2.5 Bi‐Triazines‐Based Peptide Cyclization and Dimerization

**Figure 6:**
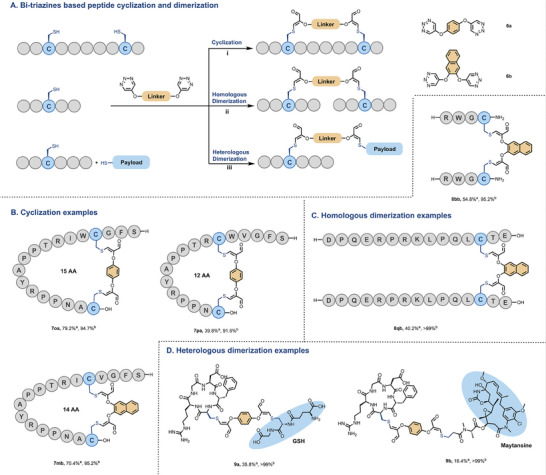
Bi‐triazines‐based peptide cyclization and dimerization. Reaction conditions: i) Peptide (1 mm)bi‐triazine (2 equiv.) in HEPES buffer (0.2 m, pH 7.4) at room temperature for 2–4 h; ii) peptide (25 mm) and **6b** (0.5 equiv.) in HEPES buffer (0.2 m, pH 7.4) at room temperature for 2–12 h; and iii) dissolve for 1 day in HEPES buffer (27.78 mm) and add it in batches to **6a** (1 equiv.) solution in DMF within 2 h. Then, the other payload (GSH or maytansine, 1.1 equiv.) was added, and the mixture was allowed to react at room temperature for 1 h. a) Isolated yield. b) HPLC purity.

Peptides have garnered substantial interest among pharmaceutical researchers owing to their heightened safety and exceptional selectivity, especially their potential to target “undruggable” entities.^[^ ^27^ ^]^ Nevertheless, their intrinsic limitations, such as brief half‐life, rapid plasma clearance, and limited membrane permeability, impose constraints on their utility.^[^ ^27b^ ^]^ To counter these constraints, prevalent strategies include peptide macrocyclization,^[^ ^28^ ^]^ dimerization,^[^ ^29^ ^]^ and coupling with payloads.^[^ ^22a^ ^]^ Therefore, we designed and synthesized a b‐triazine compound by linking two triazine moieties via a linker, which was subsequently employed under fine‐tuning reaction conditions to accommodate distinct demands (**Figure** **6**A). Here, we used hydroquinone and 2,3‐dihydroxynaphthalene as linkers to obtain Compound **6a** and **6b**, respectively. **6c** (Figure S1, Supporting Information) was also synthesized, although it has been confirmed that it cannot be used for reacting with Cys due to its unique chemical properties attributed to its biphenyl structure.

Initially, we envisioned that bi‐triazines would allow facile Cys‐Cys crosslinking to give peptide macrocycles. As expected, macrocyclic peptides, including **7oa**, **7pa**, and **7mb**, were successfully obtained with a moderate separation yield. This was achieved by treating the peptide at a concentration of 1 mm with 2 equivalents of bi‐triazines (Figure 6B). In addition to cyclic peptides, peptide dimers have been reported to have better biological activities than monomers, such as antitumour^[^ ^30^ ^]^ and antibacterial^[^ ^31^ ^]^ effects, and to act as imaging agents.^[^ ^32^ ^]^ Therefore, we used **6b** for homologous dimerization of peptides to generate peptide dimers ranging from tetrapeptides to 15 peptides (Figure 6C). In particular, **8qb** is a dimer of the biologically related peptide HPV‐E6‐N,^[^ ^20^ ^]^ with a molecular weight of 3883.8853, as verified by MALDI‐TOF MS (Figure S3, Supporting Information). Heterologous dimerization can also be used for the conjugation of peptide carriers and all kinds of payloads, including peptides and small toxin molecules. As an illustrative example, we conjugated **1d** to GSH and maytansine, which contain a thiol group, to afford **9a** and **9b**. This proof‐of‐concept is presented in Figure 6D.

### 2.6 Stability Study of the Peptide Conjugates

The stability of conjugation products holds paramount importance in devising robust strategies for peptide and protein modification. To assess the stability of triazine‐modified peptides across varied conditions, we incubated product **4db** (0.5 mM) in six distinct buffer solutions at varying pH values: 3, 5, 7.4, 9, 11, and 12.5 (Figure S4). Under acidic to weakly alkaline conditions, it exhibited commendable stability for up to 72 hours. However, as alkalinity increases, stability gradually diminishes. Furthermore, the effect of oxidation and reduction conditions on its stability has been ascertained. Intriguingly, even after a 24‐hour incubation in an H2O2 solution (5 mM), 85% of 4db remained intact (Figure S4).

**Figure 7:**
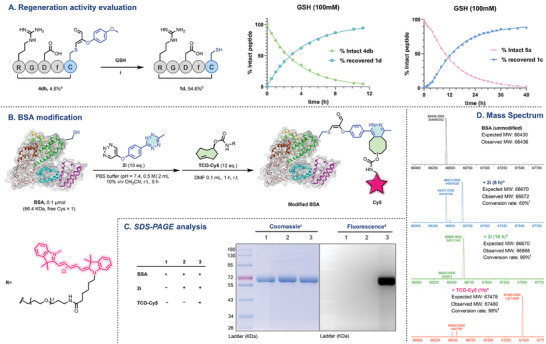
Regeneration activity evaluation of the modified peptide and modified Cys‐containing protein. i) **4db** or **5a** was dissolved in GSH (100 mm in PBS) to afford 0.5 mm peptide solutions. a) The ratio of the **4db** HPLC peak area at 220 nm at 11.25 h to that at 0 h. b) The ratio between the HPLC peak area at 220 nm after 1 day and that after 11.25 h for 0.5 mm 1 day. c) Coomassie blue staining. d) Excitation light source at 620 nm, emission filter at 699 nm, reversed‐phase image in Photoshop. e) Incubated in buffer at 37 °C. f) The conversion rate was calculated by the ratio of the intensity of mass spectrometry; refer to the supporting information for additional details.

Notably, as depicted in **Figure** **7**A, the modified peptide **4db** exhibited a gradual decrease over time, reaching a mere 4.6% after 11.25 h and two new substances were generated. Through HPLC and LCMS comparison, these were identified as the unmodified parent peptide 1d and newly formed GSH nucleophilic product, respectively. A similar experiment with a hydrazone product like **5a** was also performed. 5a exhibited a gradual decrease over time, reaching 2% after 48 h and the recovery rate of peptide **1c** reached 88% (Figure 7A). This implied that modified peptides are susceptible to external thiols, potentially reverting to their native unmodified state, suggesting the feasibility of peptide/protein regeneration.^[^ ^7^, ^33^ ^]^ Especially, by adjusting whether the aldehyde group is used to form new a conjugation product, the peptide regeneration rate can be fine‐tuned. This intriguing result provided us with a new approach for reversible peptide stapling, probing protein function, and designing new cleavable linkers for ADC or PDC drug studies.^[^ ^34^ ^]^

### 2.7 Modification of Cys‐Containing Proteins

Encouraged by these results, we proceeded to assess the potential applicability of our method for protein modification, considering its profound influence on both structure and function. We conducted trials involving two distinctively functionalized triazines (**2e** and **2i**) and incubated them with bovine serum albumin (BSA) containing a free cysteine residue. We first performed qualitative validation through SDS‒PAGE (sodium dodecyl sulfate‒polyacrylamide gel electrophoresis) and gel fluorescence imaging experiments. As illustrated in Figure S5 (Supporting Information), BSA was treated with **2e** (10 eq.) in PBS (pH 7.4, 0.5 m) at room temperature for 5 h to afford TAMRA‐labeled BSA. In addition, **2i**, which contains a tetrazine moiety, can also be used for secondary labeling of proteins. Here, we employed TCO‐Cy5 to react with the tetrazine moiety, achieving fluorescent labeling of BSA in two steps. As shown in Figure 7B, BSA and **2i** were incubated for 5 h in PBS (pH 7.4, 0.5 m). Following this, TCO‐Cy5 was introduced and allowed to react for 1 h, resulting in the successful generation of Cy5‐labeled BSA (Figure 7C). Based on this, we then fine‐tuned the reaction conditions and monitored the degree of BSA modification and conversion by mass analysis. As shown in Figure 7D, after incubating BSA and **2i** in PBS at 37 °C for 8 h, a conversion rate of 60% was achieved, which was extended to 16 h, with a conversion rate of 99%. **2e**‐related mass spectrometry data are shown in Figure S283 (Supporting Information), indicating a 70% conversion rate of BSA under the same reaction conditions. Overall, this strategic avenue not only facilitates the facile attainment of diverse protein modification variants through bioorthogonal reactions but also holds promise for numerous applications.

## 3 Conclusion

In summary, a highly versatile method involving natural cysteine moieties with broad applicability for the modification of peptides and proteins, as well as for the cyclization and dimerization of peptides, was developed. To the best of our knowledge, this represents the inaugural instance of employing 1,2,3‐triazine in biomolecular‐specific modification within a physiological solution. Through strategic modification at the 5‐position of the 1,2,3‐triazine structure, we identified a compound that displayed balanced activity and selectivity toward diverse cysteine‐containing peptides, thereby allowing for precise site modification. Additionally, we harnessed 1,2,3‐triazine compounds bearing various functional groups to facilitate conjugation with peptides, broadening the scope of their potential applications. Furthermore, by utilizing the aldehyde groups generated by the reaction of 1,2,3‐triazine with cysteine, this strategy can achieve the triple labeling requirement of “one modification, two bioorthogonal groups” in one pot, considered to be “cost‐effective”, and expand the toolbox of chemical biology. More interestingly, bi‐triazines were synthesized and used for cyclization, homodimerization, and heterodimerization of peptides. Moreover, stability experiments have shown that GSH can regenerate peptides, which presents an avenue for reversible stapling to enable the targeted delivery of bioactive peptides. In the end, the use of 1,2,3‐triazine for direct modification and secondary labeling of proteins has also been proven effective. Overall, we established a platform for cysteine‐specific modification of peptides or proteins with 5‐substituted 1,2,3‐triazine derivatives for the first time. Further research on other biologically relevant peptides and protein targets is currently the focus of our research, and we expect that this approach can facilitate the development of peptide drugs.

## Conflict of Interest

The authors declare no conflict of interest.

## Supporting information

Supporting Information

## Data Availability

The data that support the findings of this study are available in the supplementary material of this article.
