# Supplementary material for: Cysteine‐Specific Multifaceted Bioconjugation of Peptides and Proteins Using 5‐Substituted 1,2,3‐Triazines
Source: Adv Sci (Weinh). 2024 Mar 11;11(21):2308491. doi: 10.1002/advs.202308491 (PMC11151024; doi:10.1002/advs.202308491)
Supplement: Supplementary file 1 — Supporting Information [file ADVS-11-2308491-s001.pdf]

ADVANCED SCIENCE
Open Access

Supporting Information

for *Adv. Sci.*, DOI 10.1002/advs.202308491

Cysteine-Specific Multifaceted Bioconjugation of Peptides and Proteins Using 5-Substituted 1,2,3-Triazines

Quan Zuo, Yiping Li, Xuanliang Lai, Guangjun Bao, Lu Chen, Zeyuan He, Xinyi Song, Ruiyao E, Pengxin Wang, Yuntao Shi, Huixin Luo, Wangsheng Sun and Rui Wang**

WILEY-VCH

Supporting Information

Cysteine-specific Multifaceted Bioconjugation of Peptides and Proteins Using 5-Substituted 1,2,3-Triazines

Quan Zuo, Yiping Li, Xuanliang Lai, Guangjun Bao, Lu Chen, Zeyuan He, Xinyi Song, Ruiyao E, Pengxin Wang, Yuntao Shi, Huixin Luo, Wangsheng Sun,* and Rui Wang*

Table of Contents

Supplemental Items ... 3

Experimental Procedures ... 6

1 General information ... 6

1.1 Reagents ... 6

1.2 Instruments ... 6

2 UPLC-MS, analytical HPLC and preparative HPLC information ... 7

2.1 UPLC-MS analysis ... 7

2.2 Analytical HPLC ... 7

2.3 Preparative HPLC ... 8

3 Peptides preparation ... 9

4 Chemical synthesis of functionalized 1,2,3-Triazines ... 32

5 Reaction optimization ... 49

5.1 Modify GSH with **Tz-1** and reaction optimization ... 49

5.2 Modify GSH with **2a** and reaction optimization ... 50

6 Peptide scope of Cys-selective modification ... 63

7 Modification of peptide with functionalized 1,2,3-Triazines ... 97

8 Secondary labelling and one-pot triple functionalization ... 125

8.1 Secondary labelling by ketone-hydroxyl amine condensation ... 125

8.2 Secondary labelling by tetrazine ligation ... 126

8.3 One-pot triple functionalization ... 129

9 Bi-triazines based peptide cyclization and dimerization ... 135

9.1 Intramolecular cyclization using bi-triazines ... 135

9.2 Intermolecular coupling between peptides by bi-triazines ... 141

9.3 Heterologous dimerization using bi-triazines ... 145

10 Stability study of the peptide conjugates ... 151

11 Modification of Cys-containing proteins ... 161

11.1 Direct modification of proteins using **2e** ... 162

11.2 Secondary labeling of proteins using **2i** and **TCO-Cy5** ... 163

12 Spectra of new compounds ... 166

References ... 189

Author Contributions ... 189

Supplemental Items

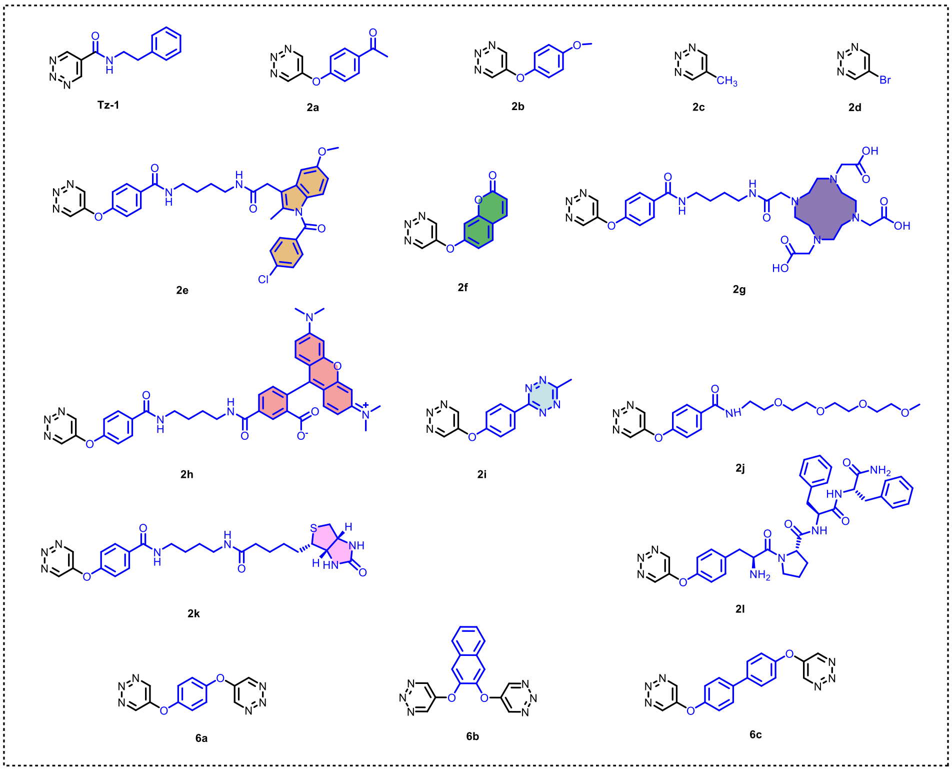

Figure S1. Functionalized 1,2,3-Triazines

Table S1. Optimization of reaction conditions

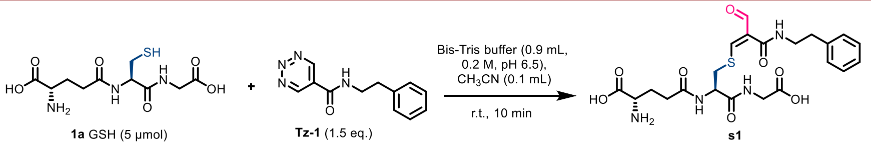

Entry[a]	Deviation from above conditions	Yield[b] (%)
1	None	95
2	PBS buffer (pH 8)	5
3	PBS buffer (pH 7.4)	6
4	PBS buffer (pH 7)	8
5	PBS buffer (pH 6.5)	80
6	Mcilvaine (pH 6.5)	63
7	3 min	92

[a] Standard reaction conditions (entry 1): 5 µmol **1a**, **Tz-1** (1.5 eq) in 1 mL Bis-Tris buffer (0.2 M) at room temperature for 10 min. [b] Determined by the peak area of HPLC chart (285 nm) using coumarin as an internal standard.

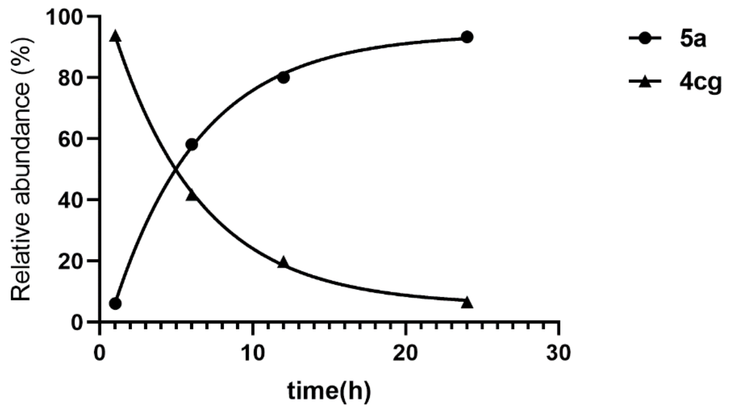

Figure S2. The relative abundance curves of **5a** and **4cg** during the reaction process monitored by LCMS-UV at 300nm.

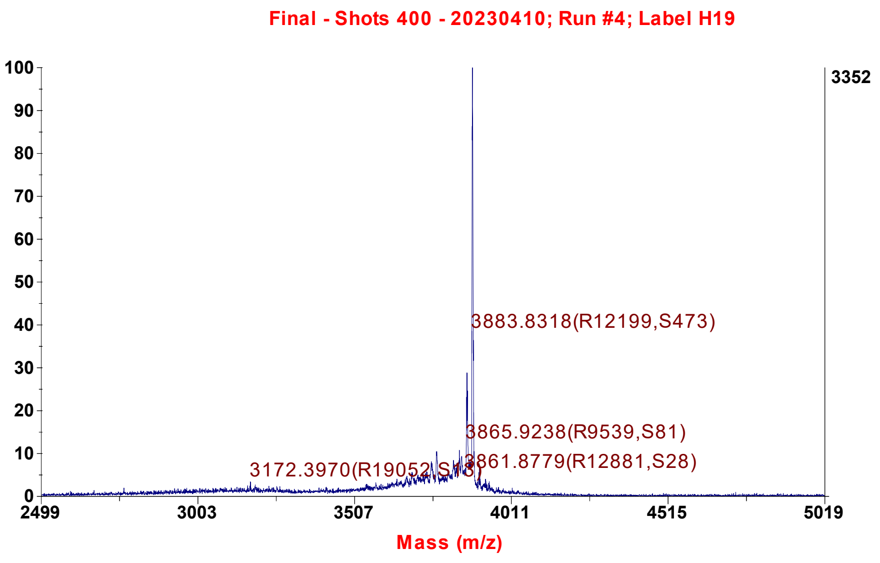

Figure S3. MADIL-TOF-HRMS spectrum of **8qb**.

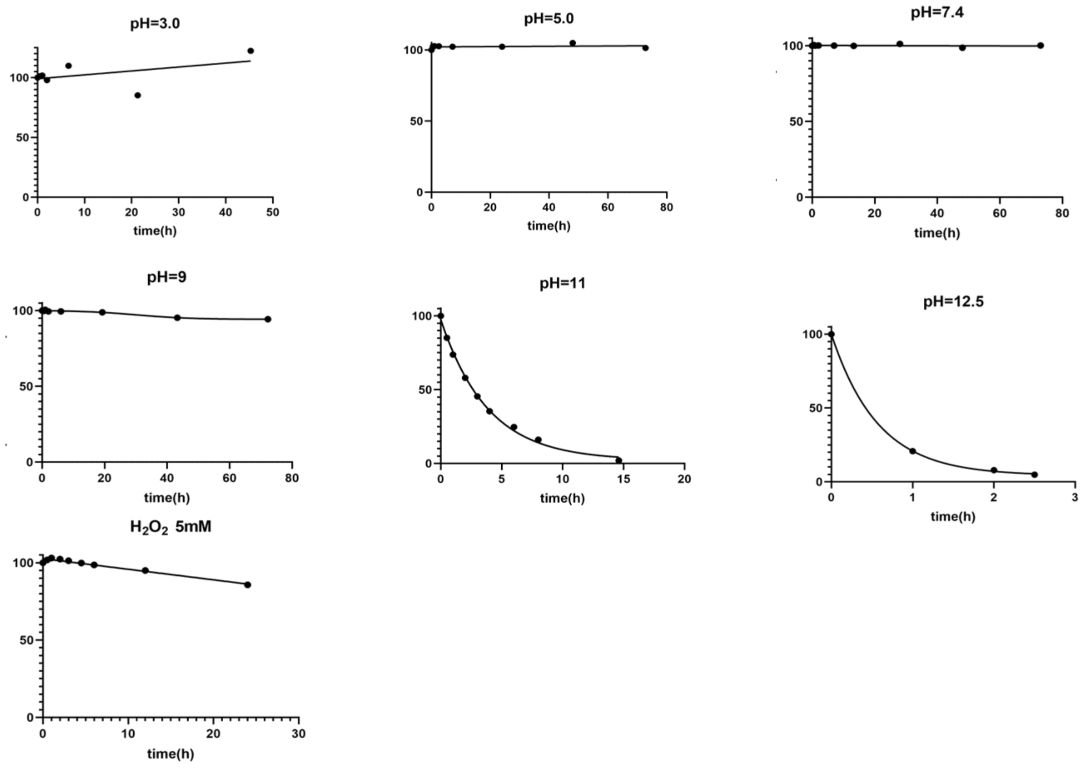

Figure S4. Stability of modified peptide **4db**.

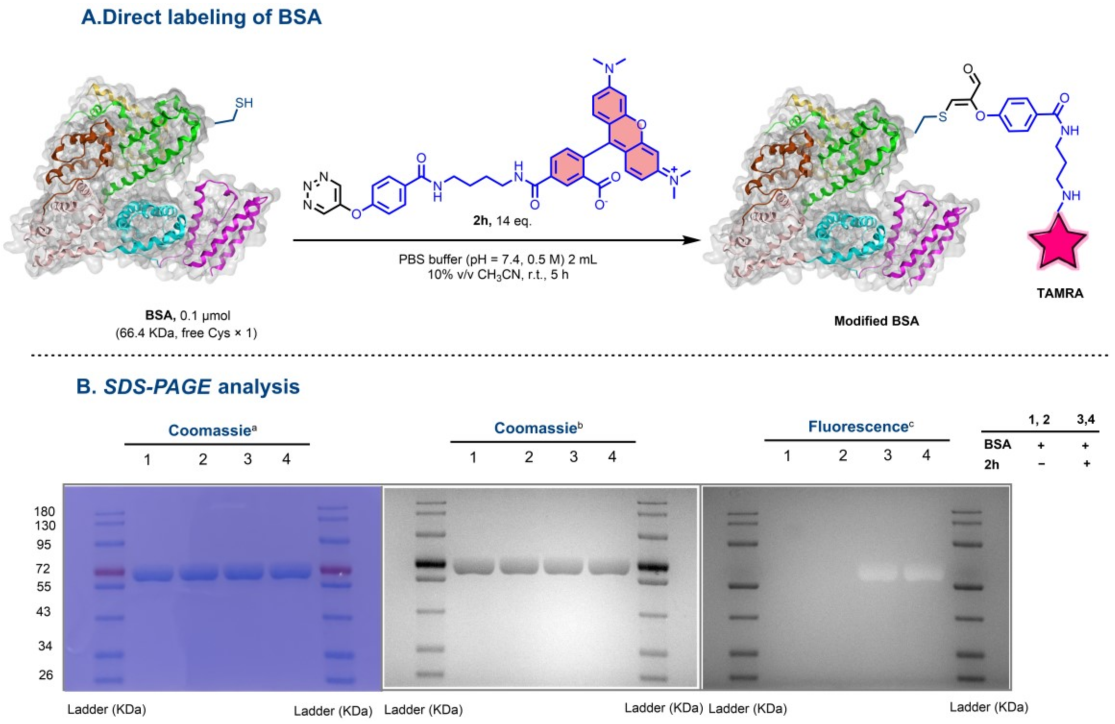

Figure S5. Direct modification of BSA using **2h.** [a] coomassie blue staining. [b] Gel imaging in Tanon imaging system. [c] Excitation light source in 530 nm, emission filter in 590 nm.

Experimental Procedures

1 General information

1.1 Reagents

Commercial available reagents and solvents were purchased from Energy Chemical, J&K Scientific, Innochem or Bidepharm and all these reagents were used directly without further purification unless otherwise noted. All kinds of buffer were purchased from MACKLIN Reagent. RP-HPLC solvents were purchased as HPLC grade from Innochem. Unless stated otherwise, all reactions were carried out in flame-dried glassware. All solvents were purified and dried according to standard methods prior to use. Some peptides were obtained using standard Fmoc SPPS-chemistry, the other peptides were ordered from Genscript Biotech Corporation and Hangzhou Zhuan Tai Biotechnology.

1.2 Instruments

^{1}H and ^{13}C NMR spectra were mainly recorded on Bruker AVANCE NEO and internally referenced to TMS signal or residual proton solvent signals. Data for ^{1}H NMR were recorded as follows: chemical shift (δ, ppm), multiplicity (s = singlet, d = doublet, t = triplet, m = multiplet, q = quartet or unresolved, dd = doublet of doublet, coupling constant(s) in Hz, integration). Data for ^{13}C NMR were reported in terms of chemical shift (δ, ppm). High-resolution mass spectra (HRMS) were acquired on Agilent Technologies 6520 Accurate-Mass Q-TOF LC/MS. MS/MS analyses were acquired on a Thermo Fisher Orbitrap Fusion™ Lumos™ Tribrid™. MALDI-TOF MS data were collected with a Waters ABI 4800plus. LC-MS spectra were performed on a Waters SQ Detector 2 mass spectrometer coupled to an Acquity ultra-performance liquid chromatography (UPLC) system. Purity analysis was carried out on Waters 2998. Semi-preparative HPLC was carried out on a SHIMADZU Essentia LC-16P using a Waters XBridge® Peptide BEH C18 (5 μm, 19 mm x 150 mm) Column. TLC analysis was visualized by fluorescence quenching under UV light (254 nm and 365 nm). Small molecule compounds were purified using Biotage IsoleraOne Flash.

2 UPLC-MS, analytical HPLC and preparative HPLC information

2.1 UPLC-MS analysis

LC–MS was performed on a SQ Detector 2 mass spectrometer coupled to an Acquity high-performance liquid chromatography (UPLC) system, equipped with UPCMA, UPBSM+, and UP-SMFTN+. Water (solvent A) and acetonitrile with 0.1% formic acid (solvent B), were used as the mobile phase at a flow rate of 0.3 mL/min. Low-resolution mass spectrometric measurements were acquired using the following parameters: positive electrospray ionization (ESI), desolvation temperature = 350 °C, source gas flow = 645 L / h, capillary voltage = 3.0 kV, cone voltage = 40 V; negative electrospray ionization (ESI), desolvation temperature = 350 °C, source gas flow = 645 L / h, capillary voltage = 2.2 kV, cone voltage = 50 V.

Following LC methods were used:

Method A (Column: Waters ACQUITY UPLC® BEH C18, 2.1 × 50 mm, 1.7 µm, flow rate 0.3 mL/min)

Time (min)	Solvent A (%)	Solvent B (%)
0	95	5
0.20	95	5
4.00	0	100
4.50	0	100
4.51	95	5
5.00	95	5

Method B (Column: Waters ACQUITY UPLC® Peptide BEH C18, 2.1 × 100 mm, 1.7 µm, flow rate 0.3 mL/min)

Time (min)	Solvent A (%)	Solvent B (%)
0	95	5
5.5	45	55
8.00	0	100
9.00	0	100
9.01	95	5
10	95	5

Method C (Waters ACQUITY UPLC® BEH C18, 2.1 × 50 mm, 1.7 µm, flow rate 0.3 mL/min)

Time (min)	Solvent A (%)	Solvent B (%)
0	95	5
0.20	95	55
8.00	0	100
8.50	0	100
8.51	95	5
10	95	5

2.2 Analytical HPLC

Analytical RP-HPLC analysis was performed on a Waters 2998, equipped with SMFTN-R, and QSM-R. All RP-HPLC analyses were done with 0.1% (v/v) trifluoroacetic acid (TFA) in water (RP-HPLC solvent A) and acetonitrile with 0.1% (v/v) TFA (RP-HPLC solvent B) as mobile phases. Typically, method D and E were used for analytical RP-HPLC analyses unless otherwise stated, method F was used for stability study.

Method D (Waters XBridge® BEH C-18, 4.6 × 150 mm, 2.5 µm, flow rate 1 mL/min)

Time (min)	Solvent A (%)	Solvent B (%)
0	95	5
30	0	100
30.01	0	100
35	0	100
35.01	95	5
40	95	5

Method E (Waters XBridge® Peptide BEH C-18, 4.6 × 250 mm, 10 µm, flow rate 1 mL/min)

Time (min)	Solvent A (%)	Solvent B (%)
0.0	90	10
20.0	10	90
35.0	0	100
40.0	90	10

Method F (Waters XBridge® BEH C-18, 4.6 × 150 mm, 2.5 µm, flow rate 1 mL/min)

Time (min)	Solvent A (%)	Solvent B (%)
0	95	5
20	0	100
20.50	0	100
25	0	100
26	95	5
30	95	5

2.3 Preparative HPLC

Purification of peptides on a semi-preparative scale was performed on a SHIMADZU Essentia LC-16P using a Waters XBridge® Peptide BEH C18 (5 µm, 19 mm x 150 mm) Column at a flow rate of 6-8 mL/min. All peptides were separated by RP-HPLC using acetonitrile with 0.1% (v/v) TFA and water as mobile phases. Derived 1,2,3-triazines were separated by RP-HPLC using acid free acetonitrile and water as mobile phases (**Attention** : If there was acid, the separation yield of 1,2,3-triazines would be severely affected and it was difficult to obtain clean products).

3 Peptides preparation

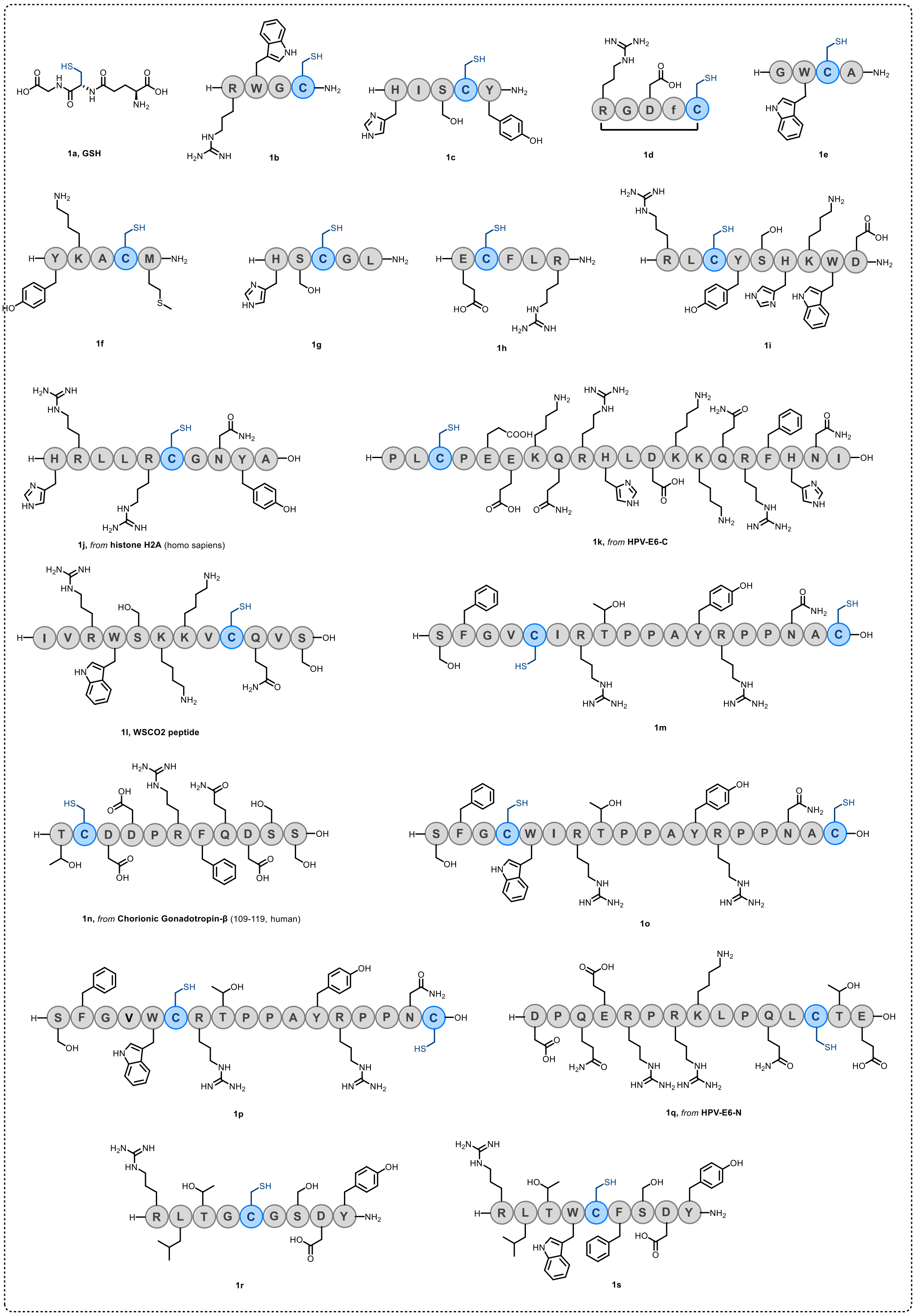

Figure S6. Cys-containing peptide substrates.

Solid-Phase Peptide Synthesis (SPPS):

Peptide synthesis was carried out manually using standard Fmoc SPPS-chemistry and Rink amide resin (0.352 mmol/g resin, 0.2 mmol scale). The remaining steps mainly refer to the literature[1].

Peptide cleavage and deprotection:

Peptides were deprotected and cleaved from the resin under reducing conditions, by treatment with 2.5% v/v water and 2.5% v/v triisopropylsilane in neat trifluoroacetic acid (10 mL). The resulting mixture was shaken for 3 hours, at room temperature. The resin was removed by filtration and peptides were precipitated in cold diethyl ether (50 mL). Peptides were pelleted by centrifugation at 4000 rpm, at 4 °C, for 5 minutes. Finally, the mother liquors were carefully removed and crude peptides were dried under vacuum.

Peptide purification and analyses:

Peptides were dissolved in water with a minimum amount of organic co-solvent (acetonitrile, dimethylformamide or dimethyl sulfoxide). Peptides were then purified on preparative RP-HPLC. Fractions containing the desired peptide were lyophilized. The purity was assessed by analyzing using Method D or E, and low-resolution mass (LRMS) measurements were also acquired using Method B.

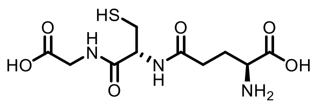

1a

1a: Analytical **HPLC** using Method D, RT (retention time) = 2.259 min, the HPLC purity is 100%.

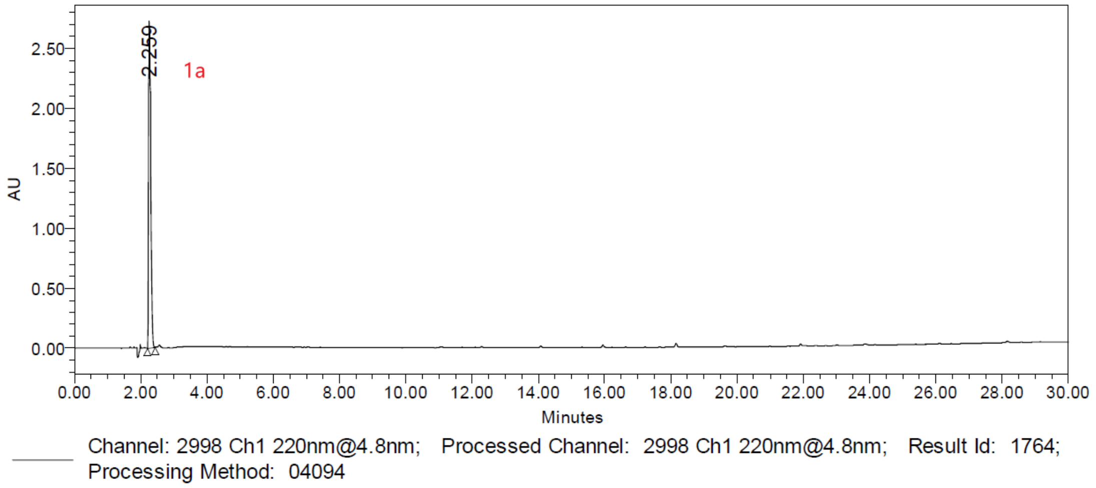

Processed Channel Descr.: 2998 Ch1 220nm@4.8nm

	Processed Channel Descr.	RT	Area	% Area	Height
1	2998 Ch1 220nm@4.8nm	2.259	12403984	100.00	2720661

Figure S7. HPLC-UV chromatogram at 220 nm of **1a**.

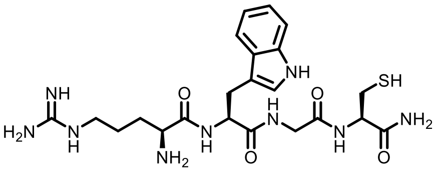

1b

1b (H-RWGC-NH2) was obtained by solid phase synthesis.

Analytical **HPLC** using Method D, RT = 6.828 min, the HPLC purity is 100.0%. **LRMS** (ESI+) *m*/*z*: 520.42 [M + H]$^+$, (ESI-) *m*/*z*: 518.53 [M - H]$^-$.

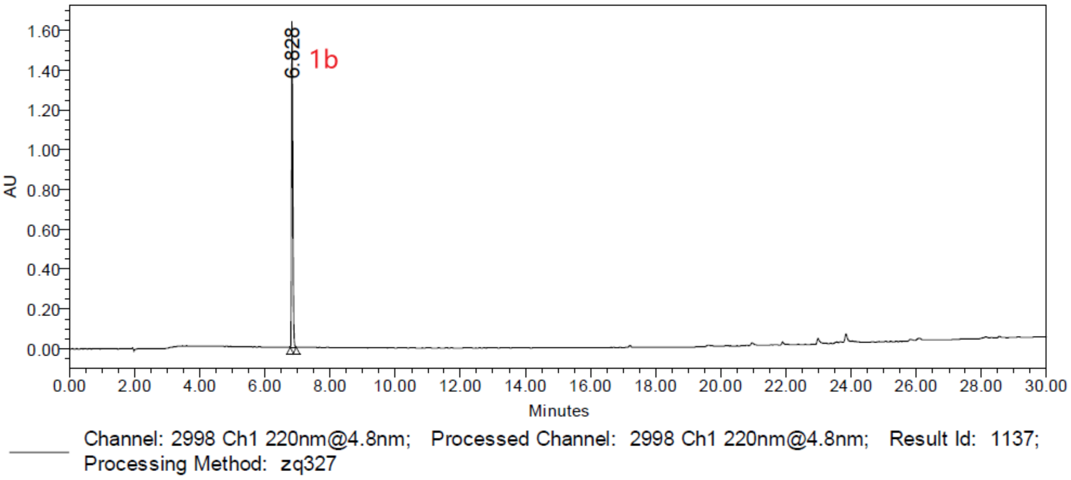

Processed Channel Descr.: 2998 Ch1 220nm@4.8nm

	Processed Channel Descr.	RT	Area	% Area	Height
1	2998 Ch1 220nm@4.8nm	6.828	4419737	100.00	1635453

Figure S8. HPLC-UV chromatogram at 220 nm of **1b**.

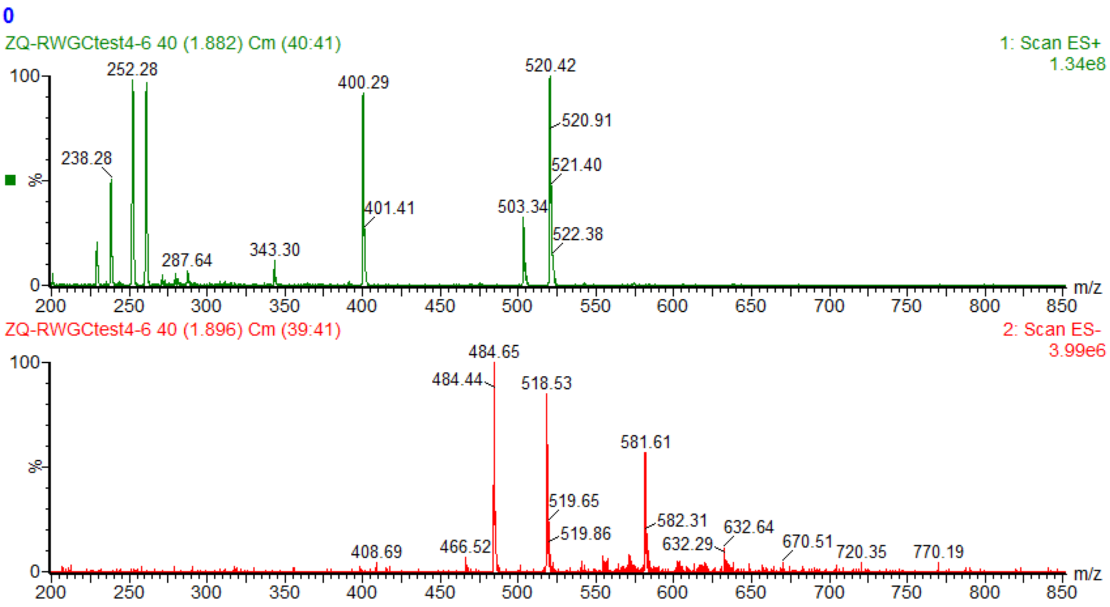

Figure S9. ESI-MS spectrum of **1b**.

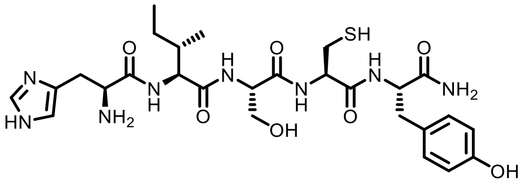

1c

1c (HISCY) was obtained by solid phase synthesis.

Analytical **HPLC** using Method D, RT = 6.347 min, the HPLC purity is 98.06%. **LRMS** (ESI+) *m/z*: 621.51 [M + H]$^+$, (ESI-) *m/z*: 619.48 [M - H]$^-$.

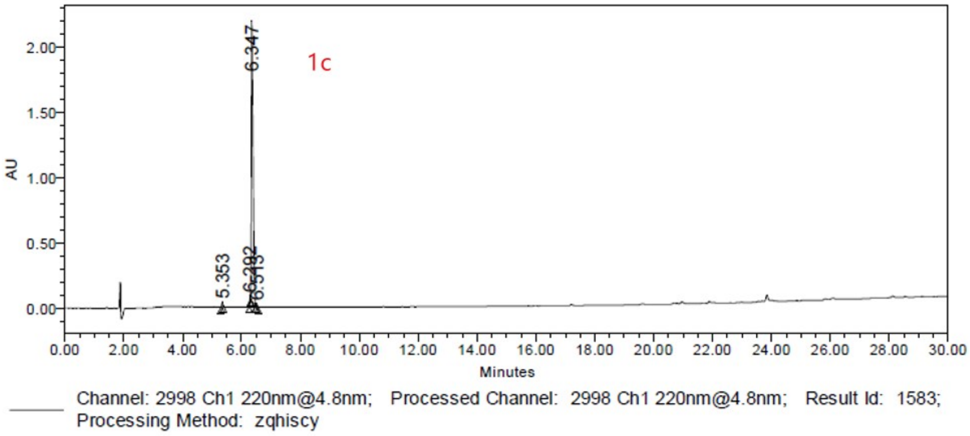

Processed Channel Descr.: 2998 Ch1 220nm@4.8nm

	Processed Channel Descr.	RT	Area	% Area	Height
1	2998 Ch1 220nm@4.8nm	5.353	68872	0.78	29971
2	2998 Ch1 220nm@4.8nm	6.292	72255	0.81	51280
3	2998 Ch1 220nm@4.8nm	6.347	8713411	98.06	2150688
4	2998 Ch1 220nm@4.8nm	6.513	31657	0.36	16227

Figure S10. ESI-MS spectrum of **1c**.

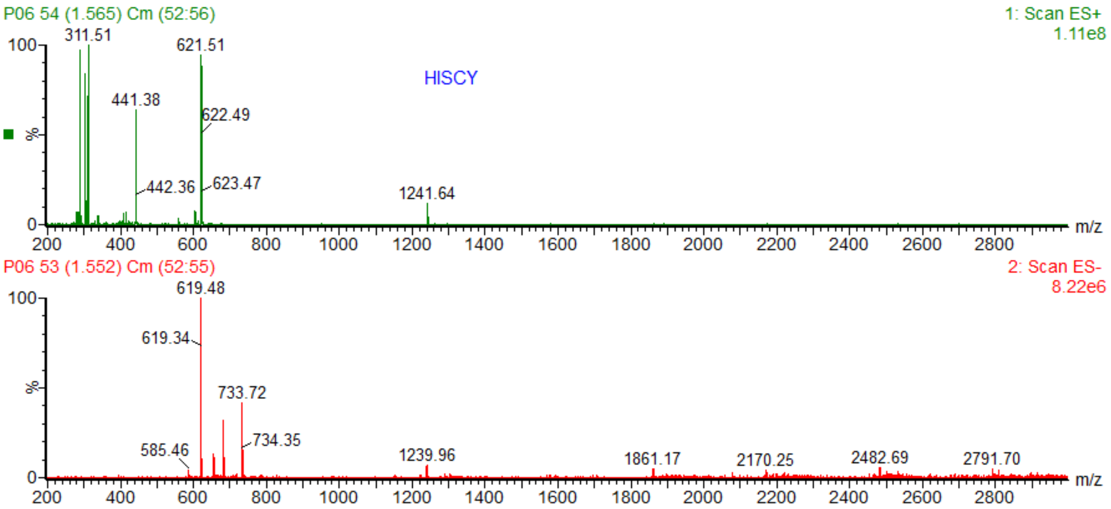

Figure S11. ESI-MS spectrum of **1c**.

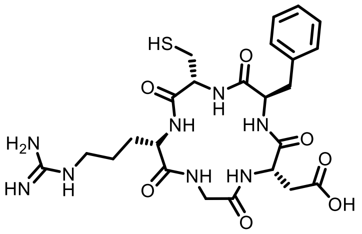

1d

1d (cyclo-RGDfC) was bought from Hangzhou Zhuan Tai Biotechnology Co., Ltd.

The HPLC purity is 98.26%. **LRMS** (ESI+) *m/z*: 579.05 $[M + H]^+$.

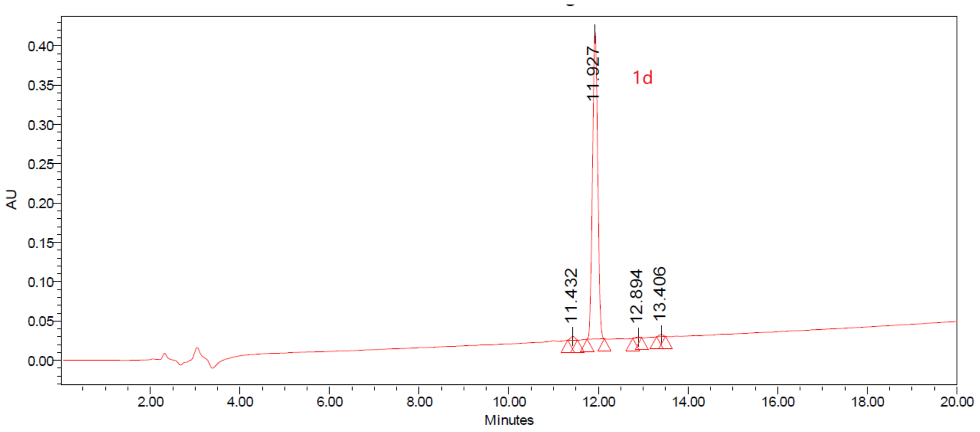

Peak Results

	RT	Area	Height	% Area	Channel Id
1	11.432	32192	4759	1.01	10237
2	11.927	3142897	391208	98.26	10237
3	12.894	7455	1023	0.23	10237
4	13.406	15998	2339	0.50	10237

Figure S12. HPLC-UV chromatogram at 220 nm of **1d**.

MASS SPECTROMETRY REPORT

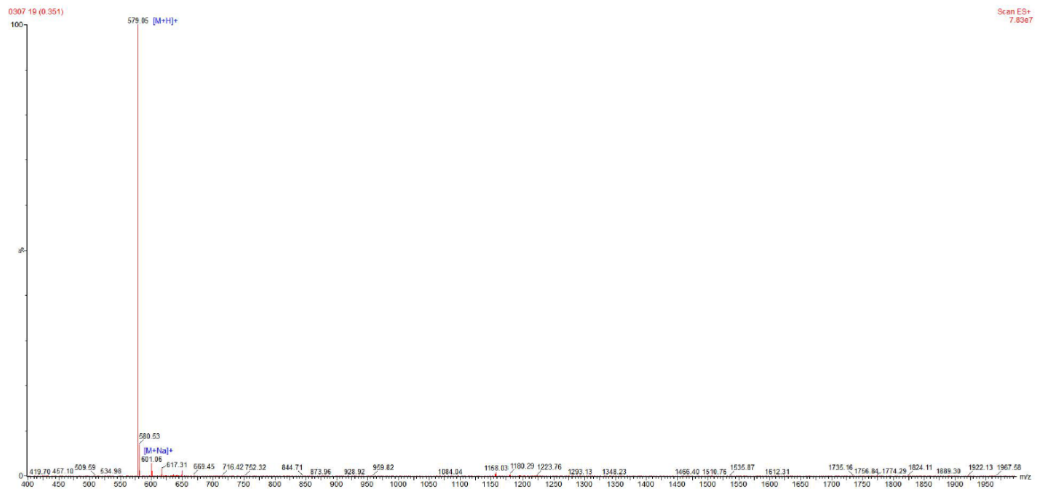

Sample Description		Instrument	Waters ZQ2000		
Analyzed date:	2022-10-14	Probe:	ESI	Probe Bias:	+4.5kv
Analyst:	PY	Nebulizer Gas Flow:	1.5L/min	Detector:	1.5kv
Sample:	C(RGDfC)	CDL:	-20.0v	T. Flow:	0.2ml/min
M.W.:	578.65	CDL Temp.:	250 °C	B. Conc.:	50%H2O/50%ACN
Lot. No.:	AP130591	Block Temp.:	200 °C		

Figure S13. ESI-MS spectrum of **1d**.

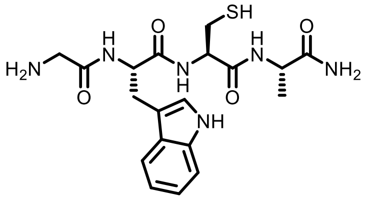

1e

1e (GWCA) was obtained by solid phase synthesis.

Analytical **HPLC** using method E, RT = 9.560 min, the HPLC purity is 100%. **LRMS** (ESI+) *m*/*z*: 869.59 [2M + H]$^+$, (ESI-) *m*/*z*: 433.61 [M - H]$^-$.

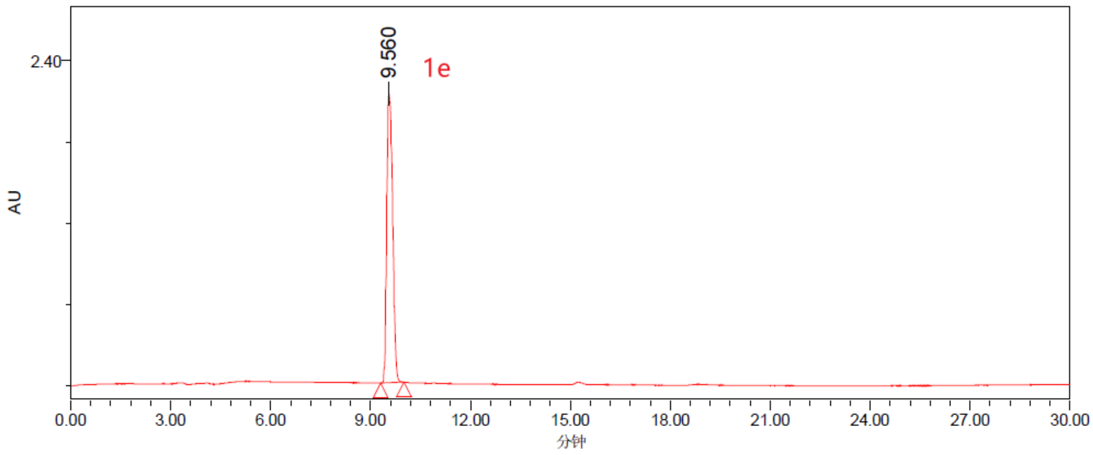

	RT	Area	% Area	Height
1	9.560	26213649	100	2123213

Figure S14. HPLC-UV chromatogram at 220 nm of **1e**.

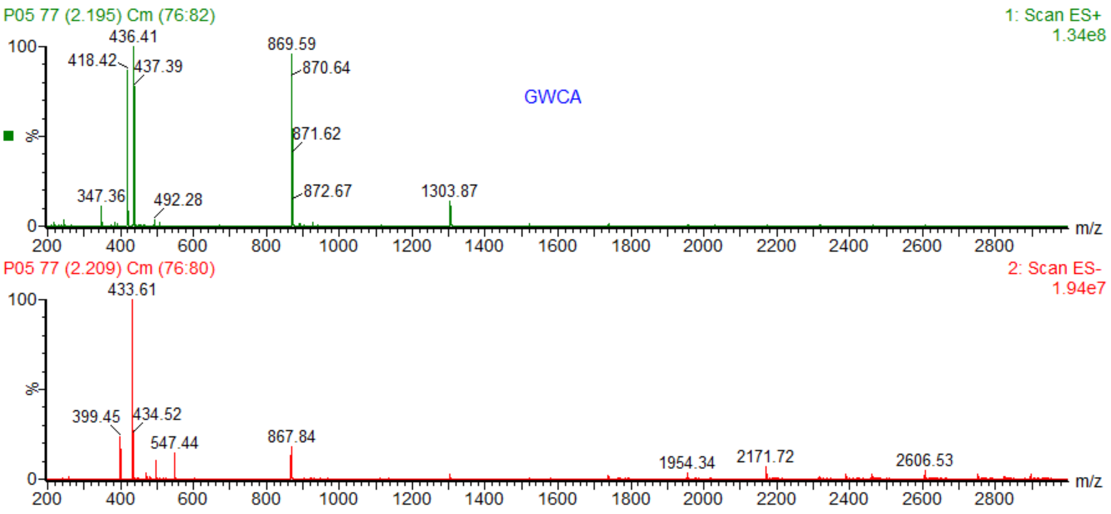

Figure S15. ESI-MS spectrum of **1e**.

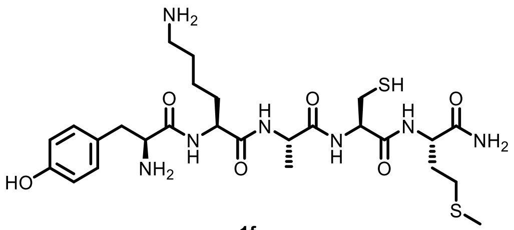

1f

1f (YKACM) was obtained by solid phase synthesis.

Analytical **HPLC** using method E, RT = 9.560 min, the HPLC purity is 100%. **LRMS** (ESI+) *m/z*: 614.23 [M + H]$^+$, (ESI-) *m/z*: 612.69 [M - H]$^-$.

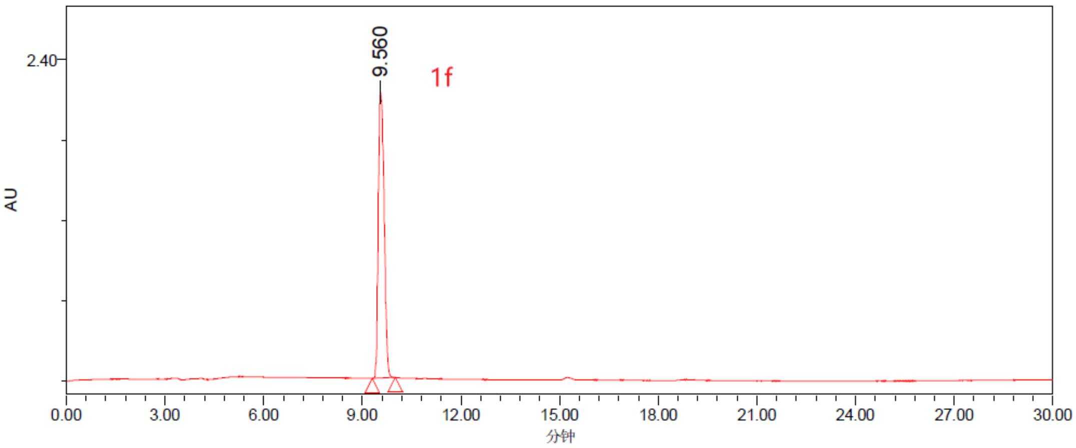

	RT	Area	% Area	Height
1	9.560	26213649	100	2123213

Figure S16. HPLC-UV chromatogram at 220 nm of **1f**.

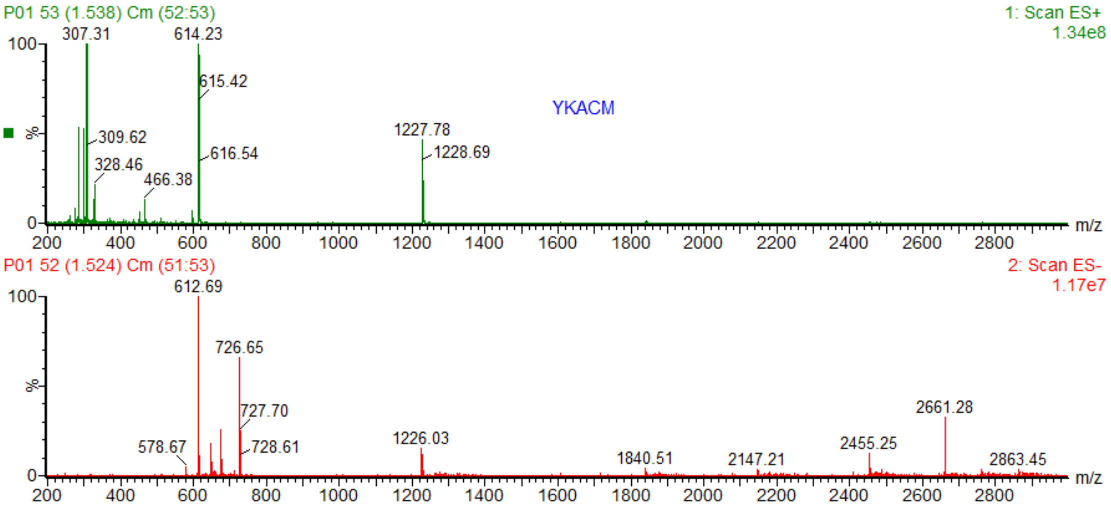

Figure S17. ESI-MS spectrum of **1f**.

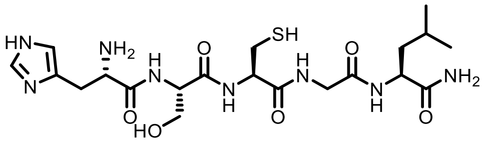

1g

1g (HSCGL) was obtained by solid phase synthesis.

Analytical **HPLC** using method E, RT = 8.158 min, the HPLC purity is 100%. **LRMS** (ESI+) *m/z*: 515.24 $[M + H]^+$, (ESI-) *m/z*: 513.70 $[M - H]^-$.

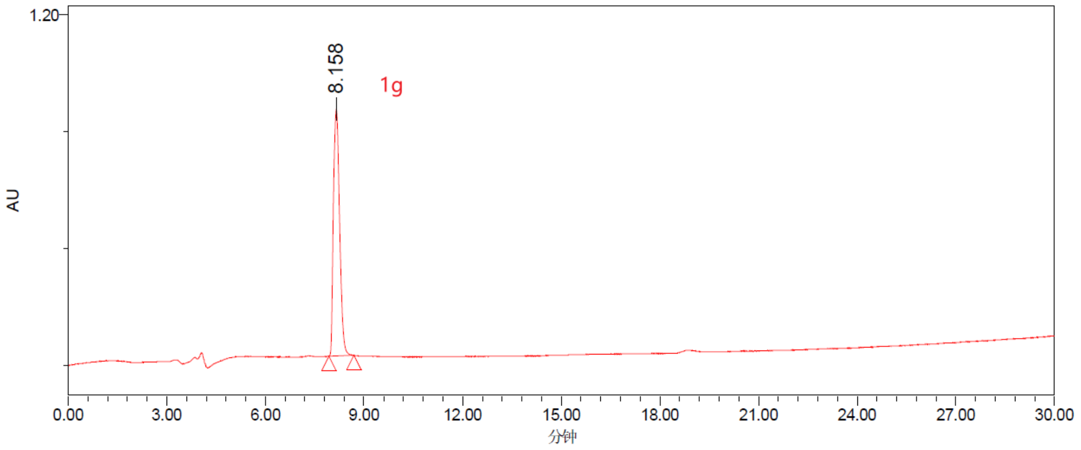

	RT	Area	% Area	Height
1	8.158	11104216	100	844816

Figure S18. HPLC-UV chromatogram at 220 nm of **1g**.

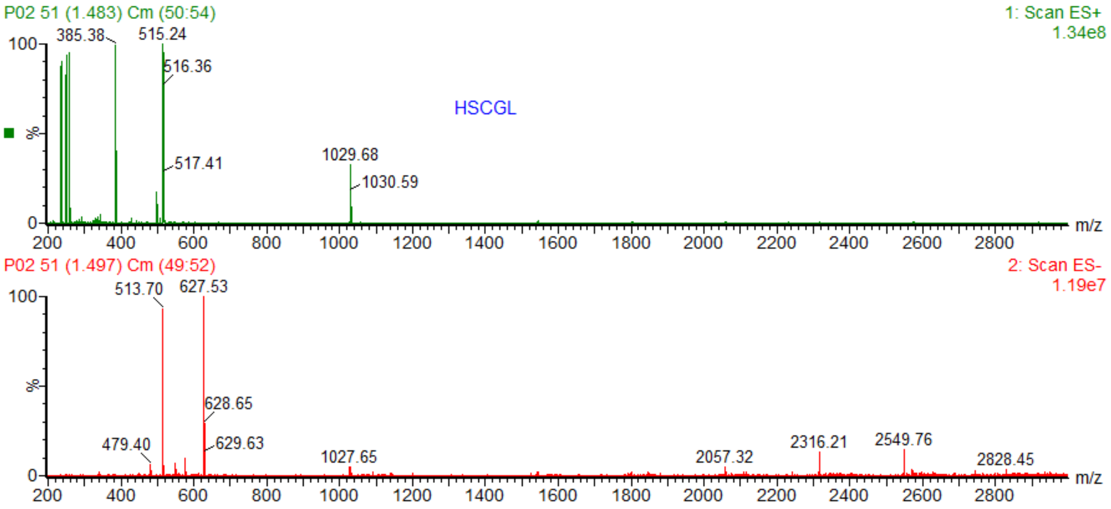

Figure S19. ESI-MS spectrum of **1g**.

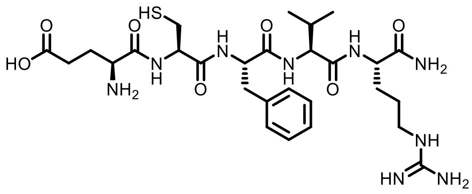

1h

1h (ECFVR) was obtained by solid phase synthesis.

Analytical **HPLC** using method E, RT = 9.297 min, the HPLC purity is 100%. **LRMS** (ESI+) *m/z*: 652.59 [M + H]$^+$, (ESI-) *m/z*: 650.77 [M - H]$^-$.

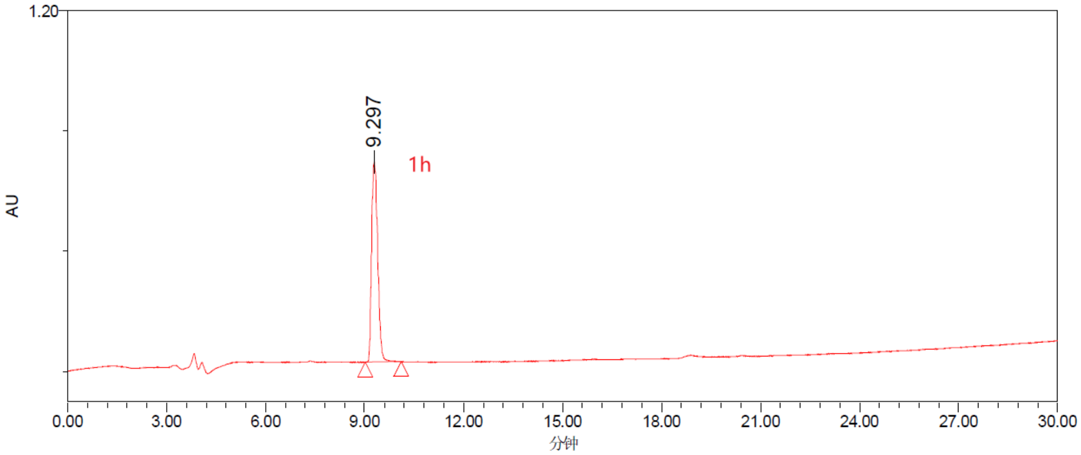

	RT	Area	% Area	Height
1	9.297	8484681	100	665644

Figure S20. HPLC-UV chromatogram at 220 nm of **1h**.

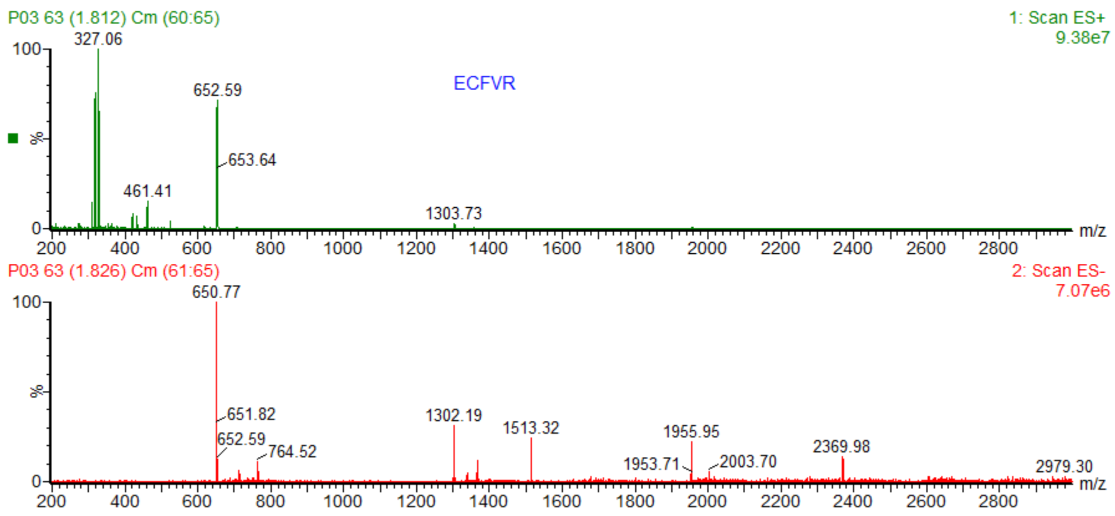

Figure S21. ESI-MS spectrum of **1h**.

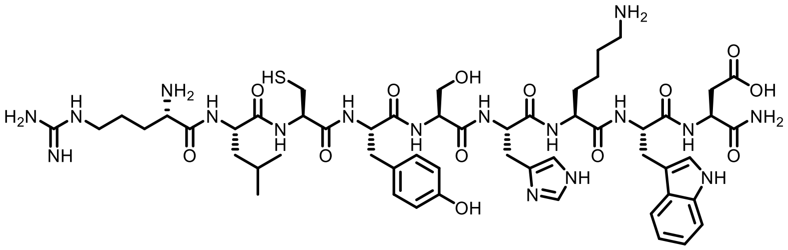

1i

1i (RLCYSHKWD) was obtained by solid phase synthesis.

Analytical **HPLC** using method E, RT = 10.146 min, the HPLC purity is 100%. **LRMS** (ESI+) *m/z*: 1206.57 $[M + H]^+$, (ESI-) *m/z*: 1204.82 $[M - H]^-$. **HRMS** (ES+) *m/z*: $[M + H]^+$ calcd for $C_{54}H_{79}N_{17}O_{13}S^+$ 1206.5842, found 1206.5856.

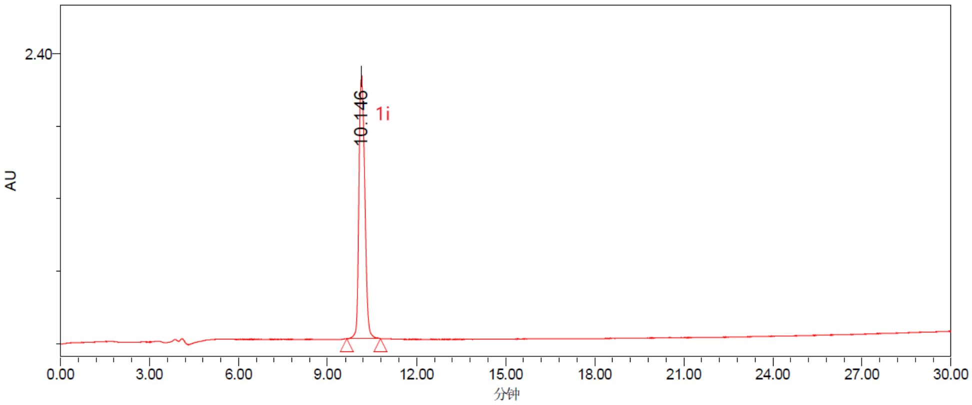

	RT	Area	% Area	Height
1	10.146	29956178	100	2136714

Figure S22. HPLC-UV chromatogram at 220 nm of **1i**.

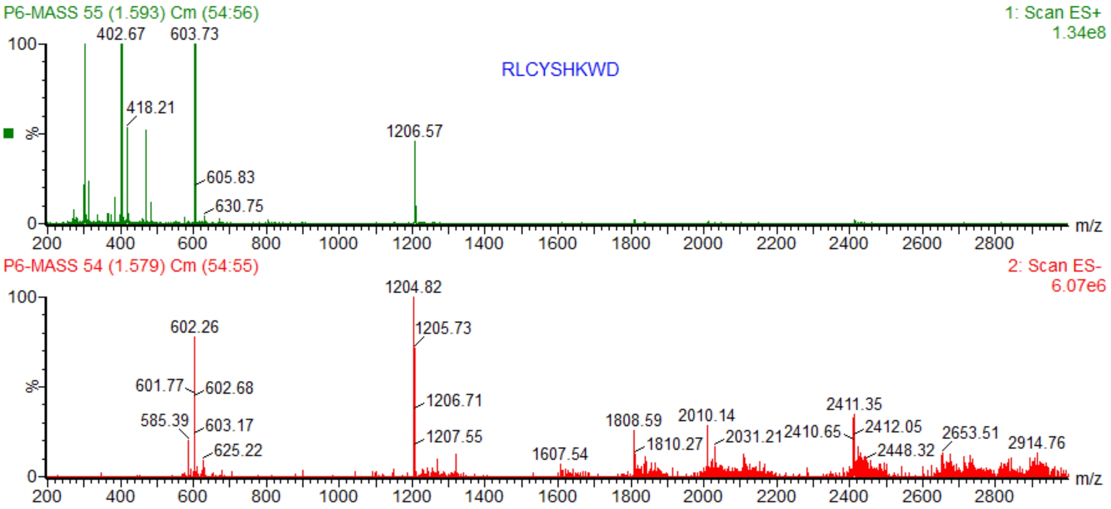

Figure S23. ESI-MS spectrum of **1i**.

Analysis Report

Agilent Trusted Answers

+ Scan (rt: 3.884 min) Sub (2) **C54 H79 N17 O13 S**

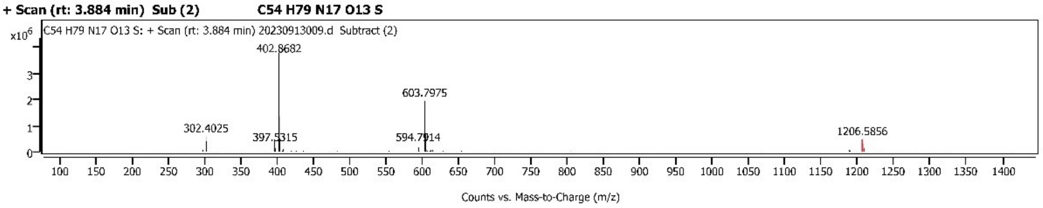

Spectrum Peaks

m/z	Z	Abund	Abund %	m/z (Calc)	Diff (ppm)	Ion Species	Formula	Ion Type
1206.5856	1	465795	12.23	1206.5837	1.62	(M+H)+	C54 H79 N17 O13 S	
1207.5871	1	326380	8.57	1207.5864	0.54	(M+H)+	C54 H79 N17 O13 S	
1208.5874	1	139820	3.67	1208.5876	-0.15	(M+H)+	C54 H79 N17 O13 S	
1209.5878	1	44279	1.16	1209.5887	-0.73	(M+H)+	C54 H79 N17 O13 S	
297.8993		73688	1.93					
298.1491		44553	1.17					
302.4025	2	593719	15.58					
302.6528	2	409121	10.74					
302.9032	2	185108	4.86					
303.1530	2	64437	1.69					
396.8639	1	483050	12.68					
397.1981		355264	9.32					
397.5315	2	155903	4.09					
397.8660	1	54377	1.43					
402.8682	2	3810032	100.00					
403.0435		219818	5.77					
403.2020	1	2851778	74.85					
403.3793	2	193529	5.08					
403.5355	2	1367027	35.88					
403.8688	2	517086	13.57					
404.2023	1	130937	3.44					
407.5379	2	55922	1.47					
407.8715		39336	1.03					
408.1996		156729	4.11					
408.5330	2	108990	2.86					
408.8661		54899	1.44					
419.5266	2	43323	1.14					
436.5513	2	48991	1.29					
594.7914	2	244786	6.42					
595.2928	2	169501	4.45					
595.7931	2	77569	2.04					
603.2941		49432	1.30					
603.5454		64163	1.68					
603.7975	2	1951799	51.23					
604.2987	2	1377483	36.15					
604.7989	2	672902	17.66					
605.2987	2	218752	5.74					
605.7972	2	64136	1.68					
607.5439		40460	1.06					
611.7945	2	83942	2.20					
612.2947	2	56571	1.48					
614.7871	2	67227	1.76					
615.2885	2	51613	1.35					
1188.5731	1	85551	2.25					
1189.5750	1	57200	1.50					

Spectrum Identification Table

Best	ID Source	Name	Formula	Species	m/z	Diff (ppm)	CAS	Score	Score (Lib)	Score (DB)	Score (MFG)	Lib/DB
Yes	MFG		C54 H79 N17 O13 S	(M+H)+	1206.5856	0.83		97.96			97.96	

Figure S24. Q-TOF-HRMS spectrum of **1i**.

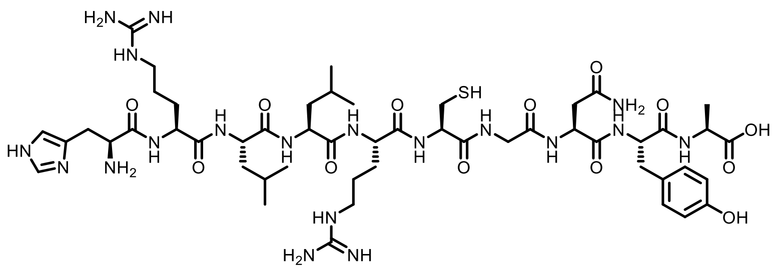

1j, *from* ***histone H2A*** *(homo sapiens)*

1j (HRLLRCGNYA) was bought from GenScript Biotech Corporation.

The **HPLC** purity is 97.51%. **LRMS** (ESI+) *m*/*z*: 1202.7 [M + H]$^+$.

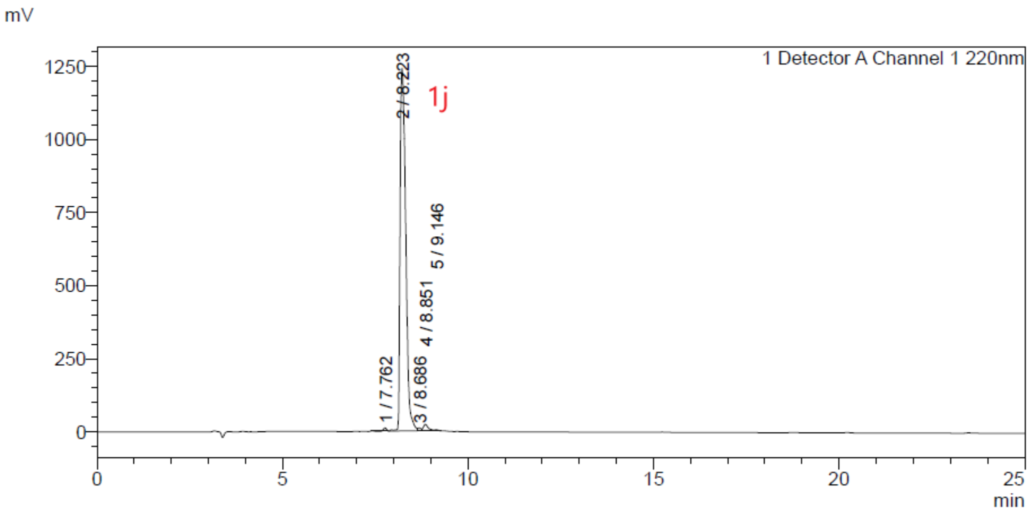

<Peak Table>

Detector A Channel 1 220nm

Peak#	Ret. Time	Area	Height	Area%
1	7.762	45708	10552	0.342
2	8.223	13051311	1241505	97.512
3	8.686	64571	9952	0.482
4	8.851	201024	22175	1.502
5	9.146	21694	3370	0.162
Total		13384308	1287554	100.000

Figure S25. HPLC-UV chromatogram at 220 nm of **1j**.

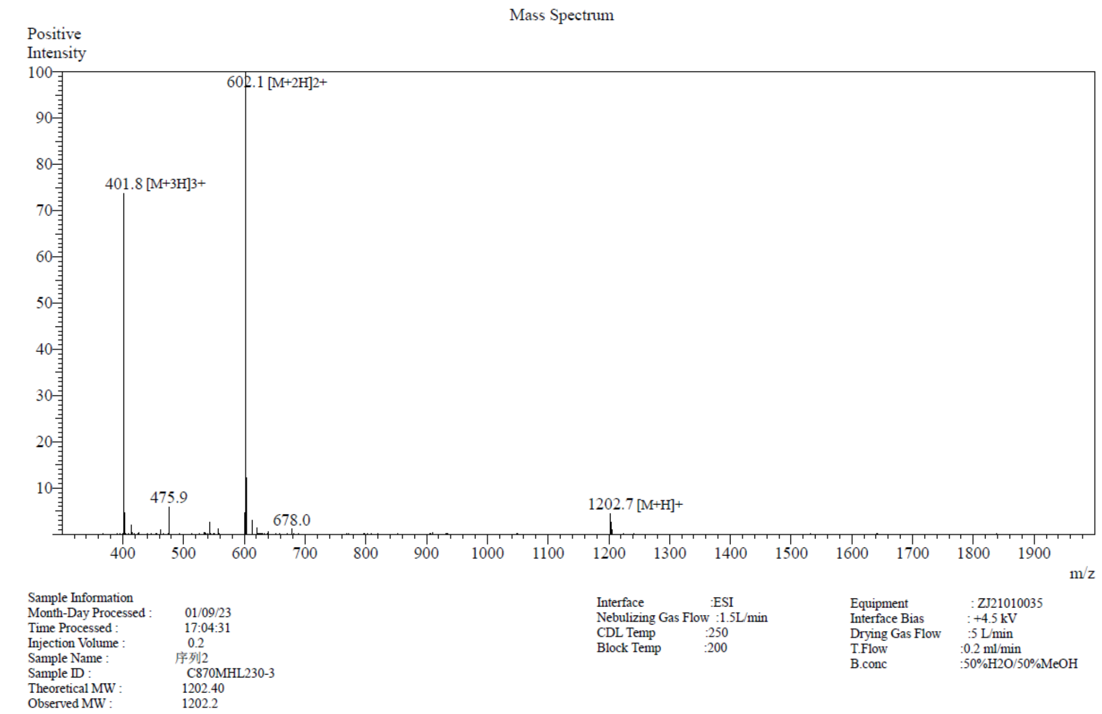

Figure S26. ESI-MS spectrum of **1j**.

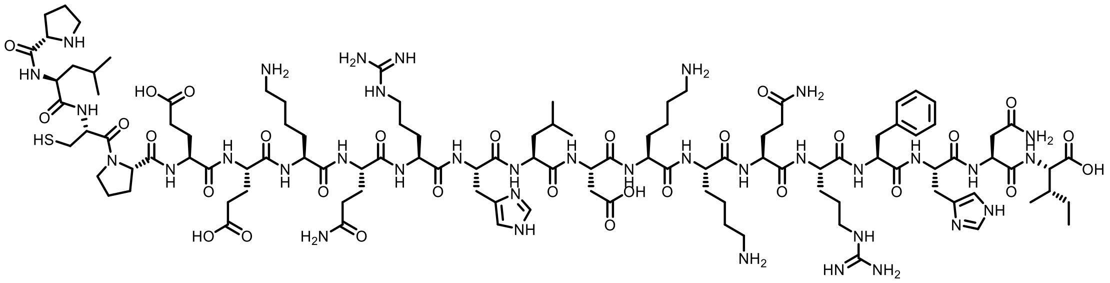

1k, from ***HPV-E6-C***

1k (PLCPEEKQRHLDKKQRFHNI) was obtained by solid phase synthesis.

Analytical **HPLC** using Method E, RT = 10.645 min, the HPLC purity is 100%. **HRMS** (ES+) *m/z*: $[M + 2H]^{2+}$ calcd for $C_{110}H_{178}N_{36}O_{30}S^{+2}$ 1259.1732, found1259.1657.

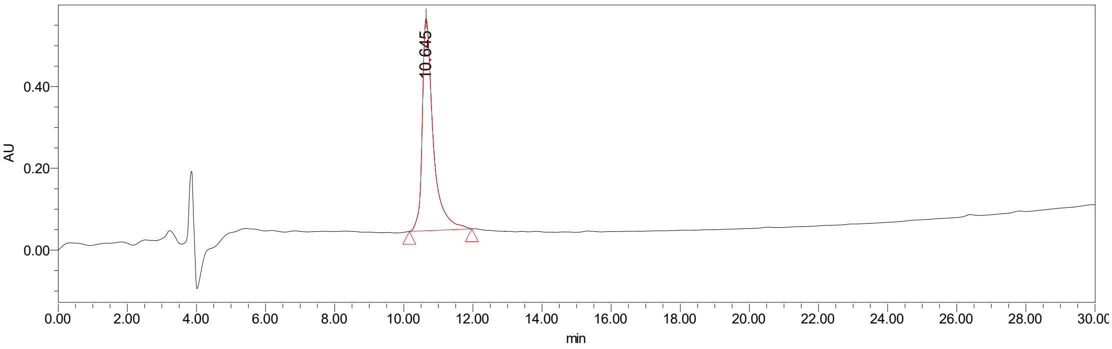

	RT	Area	% Area	Height
1	10.645	11449302	100.00	519202

Figure S27. HPLC-UV chromatogram at 220 nm of **1k**.

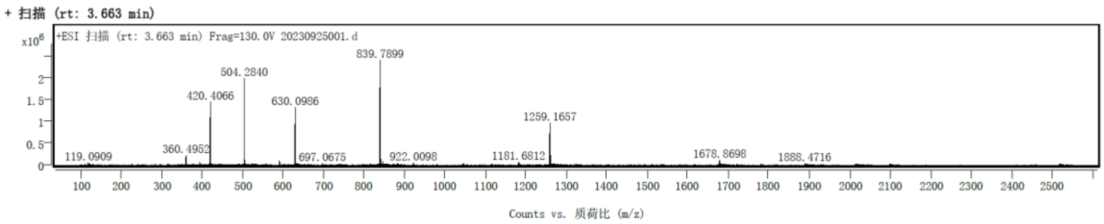

Figure S28. Q-TOF-HRMS spectrum of **1k**.

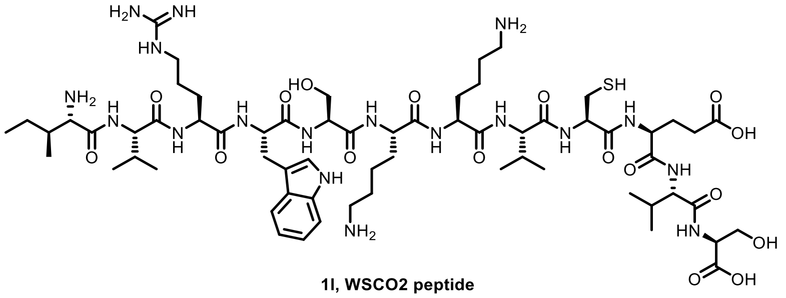

1l, WSCO2 peptide

1l (IVRWSKKVCQVS) was bought from GenScript Biotech Corporation.

The **HPLC** purity is 98.20%. **LRMS** (ESI+) *m/z*: 717.2 $[M + 2H]^{2+}$.

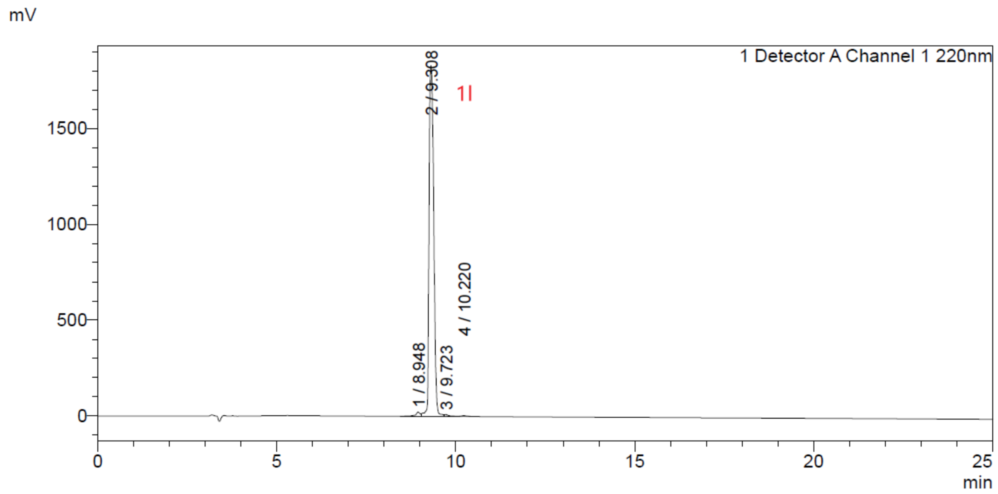

<Peak Table>

Detector A Channel 1 220nm

Peak#	Ret. Time	Area	Height	Area%
1	8.948	195941	21904	1.169
2	9.308	16458822	1824291	98.199
3	9.723	79599	8802	0.475
4	10.220	26266	3426	0.157
Total		16760629	1858423	100.000

Figure S29. HPLC-UV chromatogram at 220 nm of **1l**.

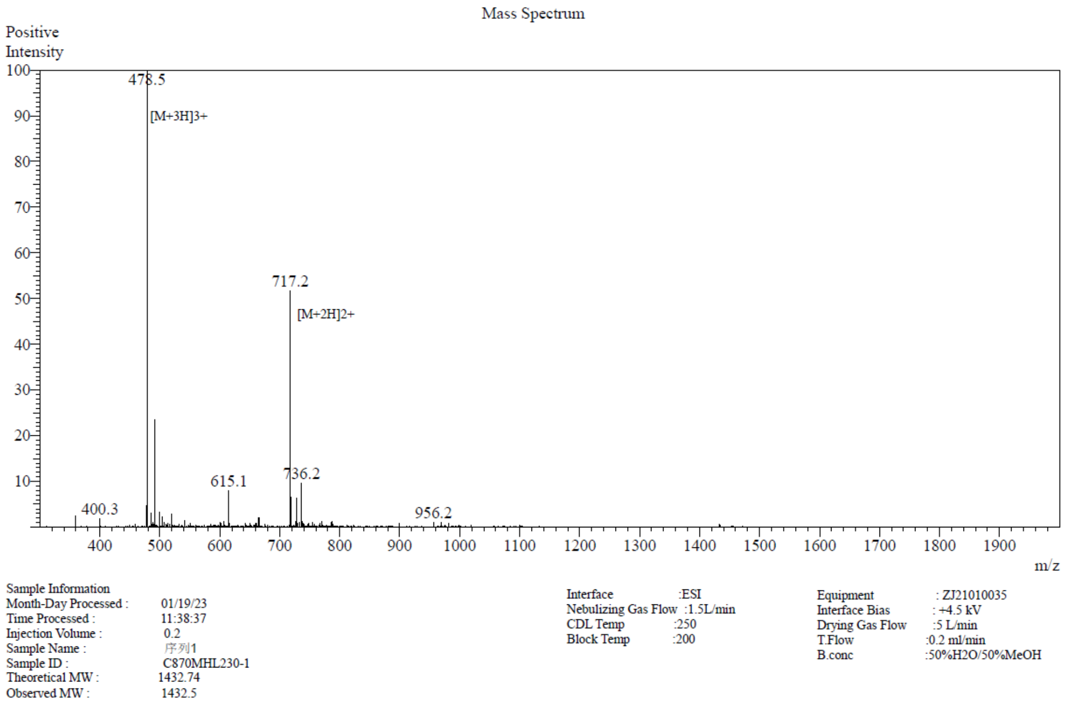

Figure S30. Q-TOF-HRMS spectrum of **1l**.

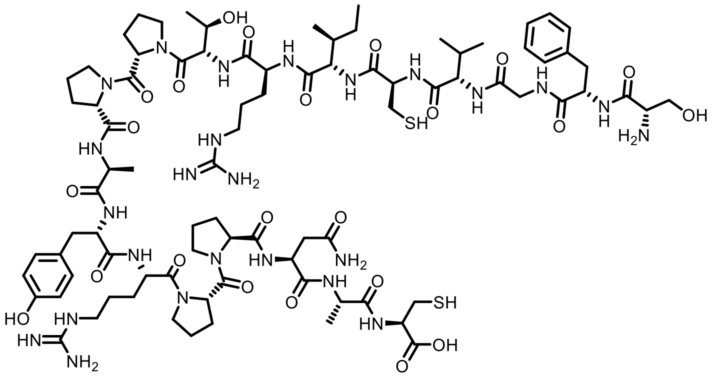

1m

1m (SFGVCIRTPPAYRPPNAC) was bought from GenScript Biotech Corporation.

The HPLC purity is 90.47%. **LRMS** (ESI+) *m/z*: 975.2 $[M + 2H]^{2+}$.

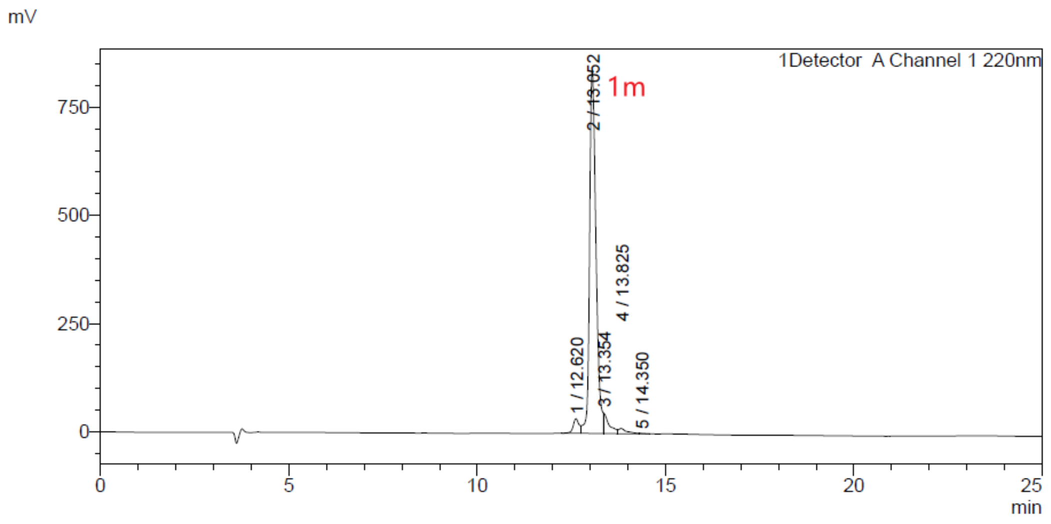

<Peak Table>

Detector A Channel 1 220nm

Peak#	Ret. Time	Area	Height	Area%
1	12.620	361141	33555	3.295
2	13.052	9916971	840057	90.471
3	13.354	481280	47615	4.391
4	13.825	190758	12357	1.740
5	14.350	11316	1315	0.103
Total		10961465	934899	100.000

Figure S31. HPLC-UV chromatogram at 220 nm of **1m**.

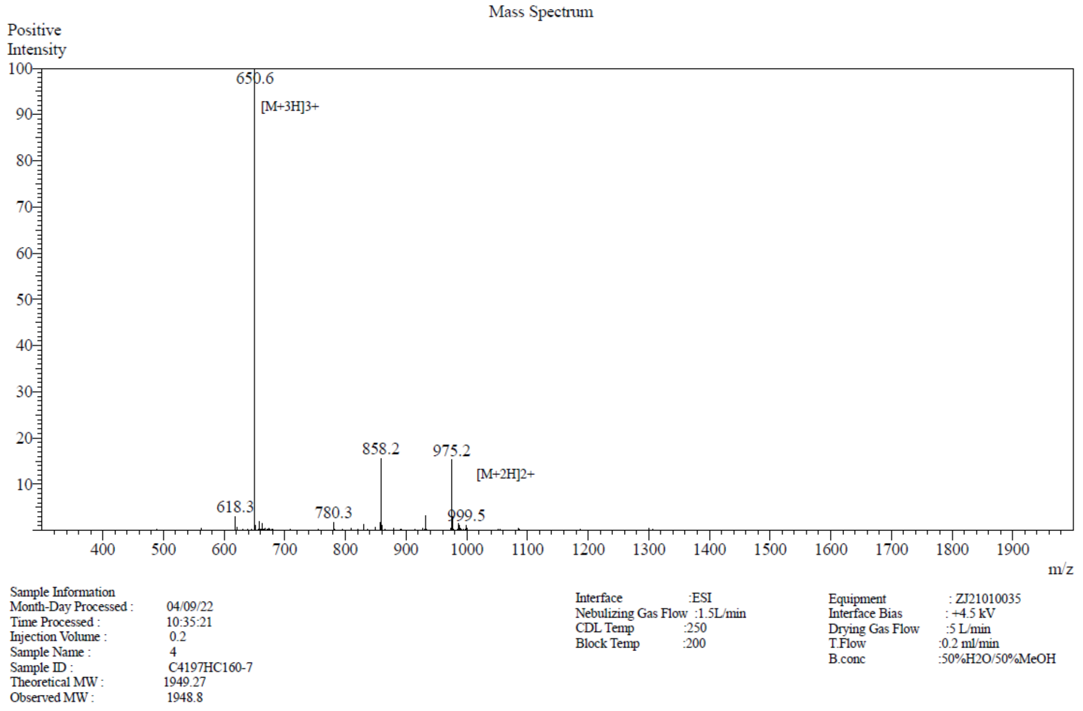

Figure S32. ESI-MS spectrum of **1m**.

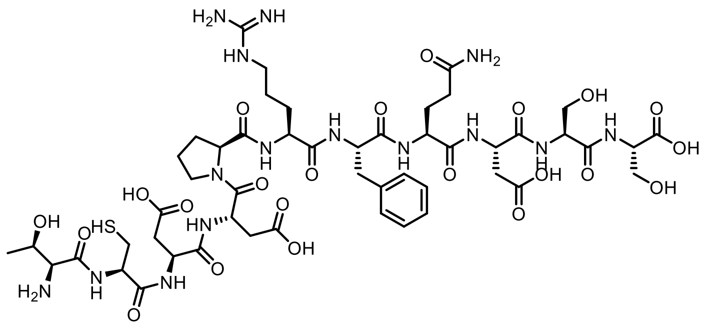

1n, *from **Chorionic Gonadotropin-B (109-119, human)***

1n (TCDDPRFQDSS) was obtained by solid phase synthesis.

Analytical **HPLC** using Method E, RT = 8.304 min, the HPLC purity is 93.06%. **HRMS** (ES+) *m/z*: $[M + H]^+$ calcd for $C_{50}H_{75}N_{15}O_{22}S^+$ 1270.5009, found1270.4948.

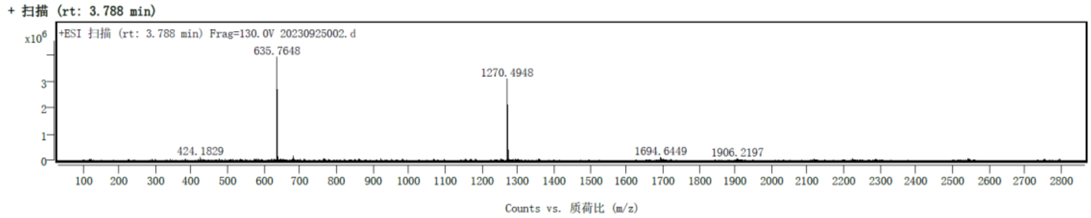

Figure S33. Q-TOF-HRMS spectrum of **1n**.

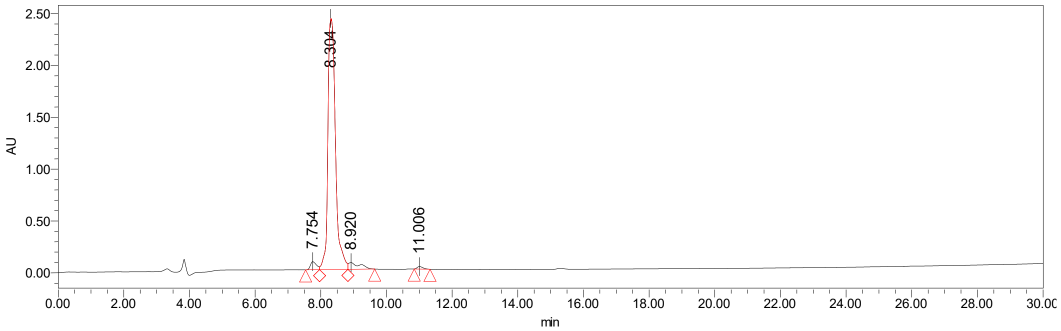

	RT	Area	% Area	Height
1	7.754	1001801	2.41	78009
2	8.304	38684674	93.06	2421538
3	8.920	1585439	3.81	64622
4	11.006	299924	0.72	24620

Figure S34. HPLC-UV chromatogram at 220 nm of **1n**.

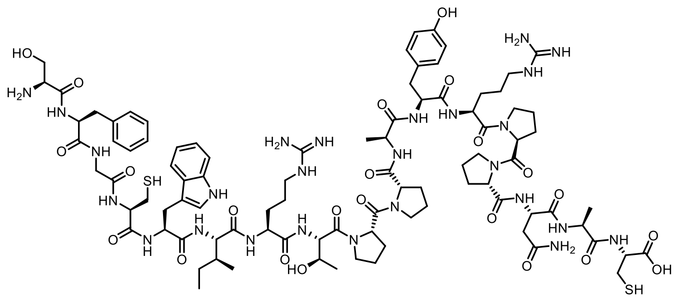

1o

1o (H-SFGCWIRTPPAYRPPNAC-OH) was bought from GenScript Biotech Corporation.

The HPLC purity is 90.51%. **LRMS** (ESI+) *m/z*: 1018.9 $[M + 2H]^{2+}$.

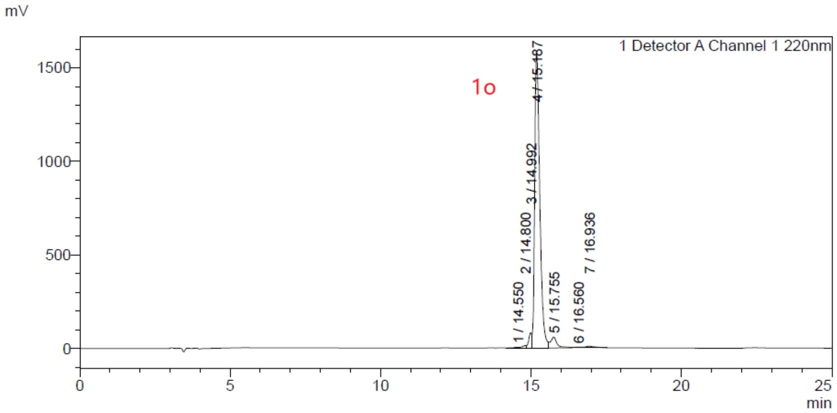

<Peak Table>

Detector A Channel 1 220nm

Peak#	Ret. Time	Area	Height	Area%
1	14.550	57133	3874	0.278
2	14.800	113963	13074	0.555
3	14.992	593267	81426	2.891
4	15.187	18574726	1565428	90.507
5	15.755	929021	58251	4.527
6	16.560	59573	3386	0.290
7	16.936	195347	8591	0.952
Total		20523028	1734031	100.000

Figure S35. HPLC-UV chromatogram at 220 nm of **1o**.

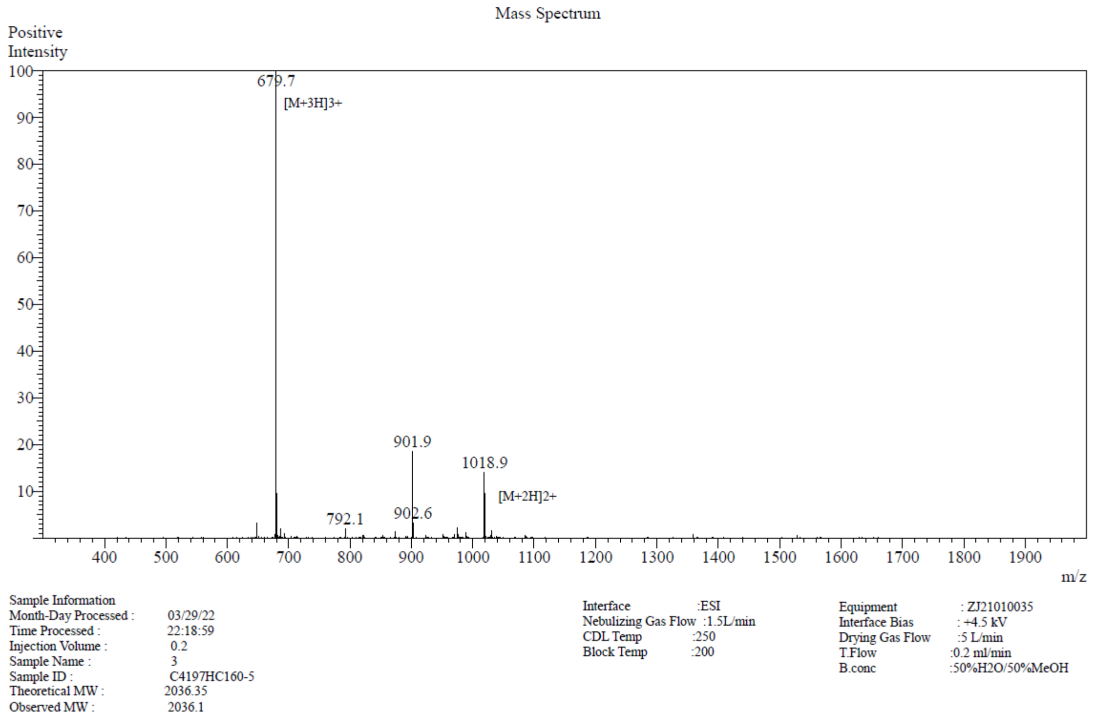

Figure S36. ESI-MS spectrum of **1o**.

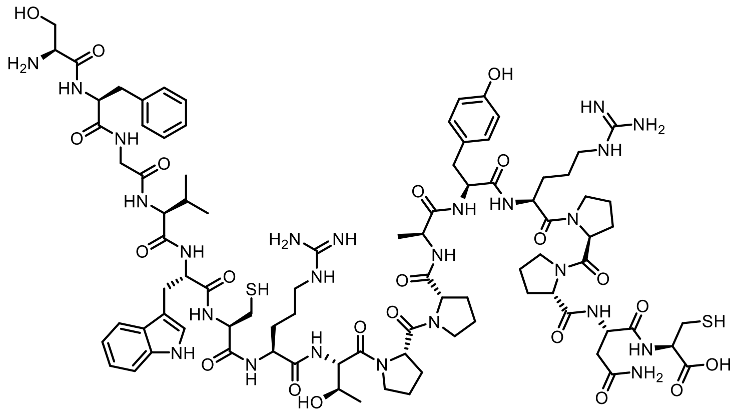

1p

1p (H-SFGVWCRTPPAYRPPNC-OH) was bought from GenScript Biotech Corporation.

The HPLC purity is 90.29%. **LRMS** (ESI+) *m/z*: 976.5 $[M + 2H]^{2+}$.

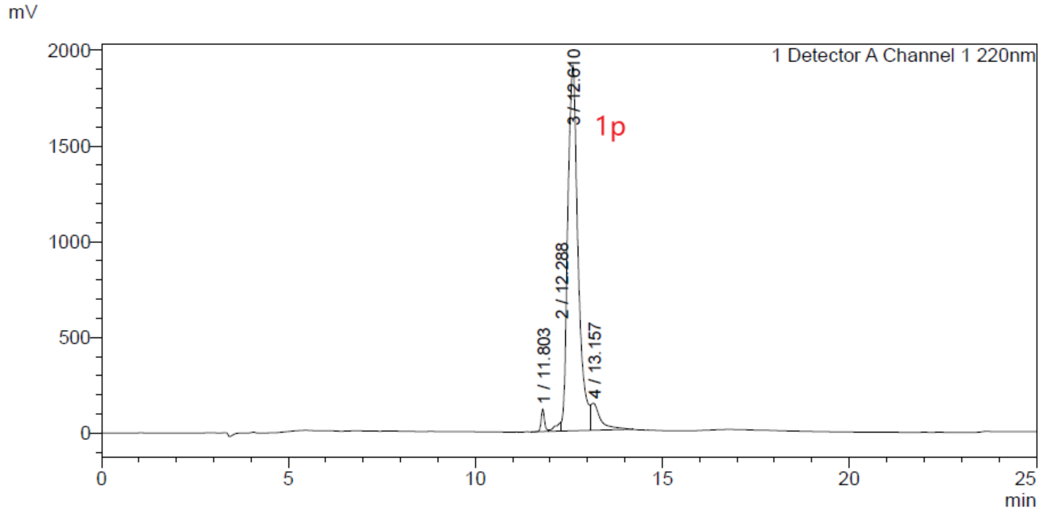

<Peak Table>

Detector A Channel 1 220nm

Peak#	Ret. Time	Area	Height	Area%
1	11.803	747078	118425	1.858
2	12.288	494152	45226	1.229
3	12.610	36308522	1911707	90.292
4	13.157	2662403	141888	6.621
Total		40212154	2217246	100.000

Figure S37. HPLC-UV chromatogram at 220 nm of **1p**.

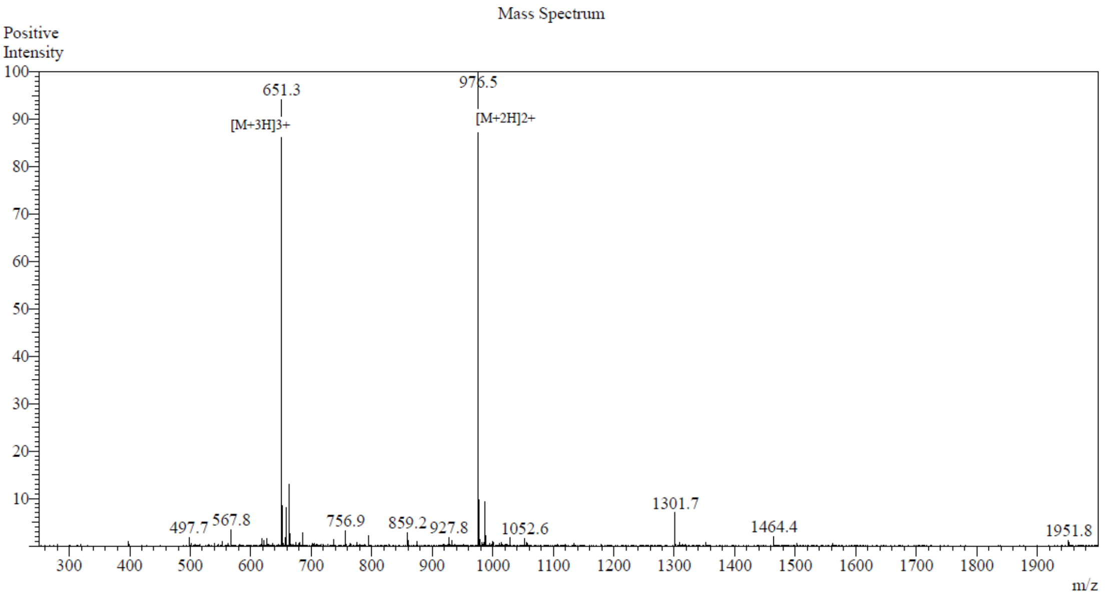

Sample Information	
Month-Day Processed :	03/25/22
Time Processed :	12:07:53
Injection Volume :	0.2
Sample Name :	5
Sample ID :	C4197HC160-9
Theoretical MW :	1951.24
Observed MW :	1951.0

Interface	:ESI
Nebulizing Gas Flow	:1.5L/min
CDL Temp	:250
Block Temp	:200

Equipment	: GK11010007
Interface Bias	: +4.5 kV
Drying Gas Flow	:5 L/min
T.Flow	:0.2 ml/min
B.conc	:50%H2O/50%MeOH

Figure S38. ESI-MS spectrum of **1p**.

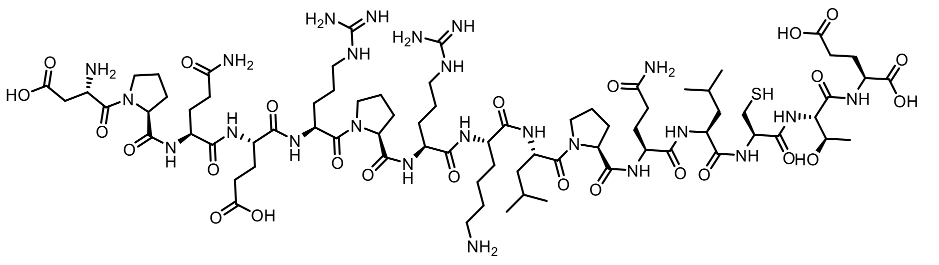

1q, *from* ***HPV-E6-N***

1q (H-DPQERPRKLPQLCTE-OH) was obtained by solid phase synthesis.

Analytical **HPLC** using Method E, RT = 10.672 min, the HPLC purity is 97.15%. **HRMS** (ES+) *m/z*: $[M + 2H]^{2+}$ calcd for $C_{76}H_{128}N_{24}O_{25}S^{+}$ 905.4680, found 905.4737.

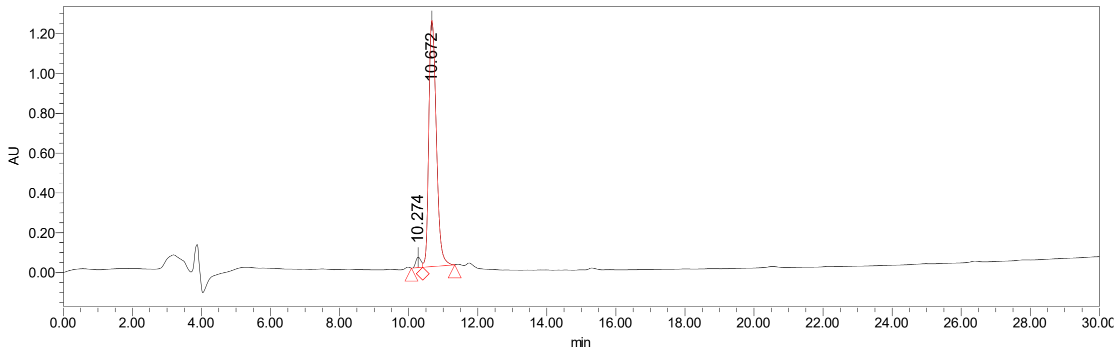

	RT	Area	% Area	Height
1	10.274	548990	2.85	53011
2	10.672	18724749	97.15	1236995

Figure S39. HPLC-UV chromatogram at 220 nm of **1q**.

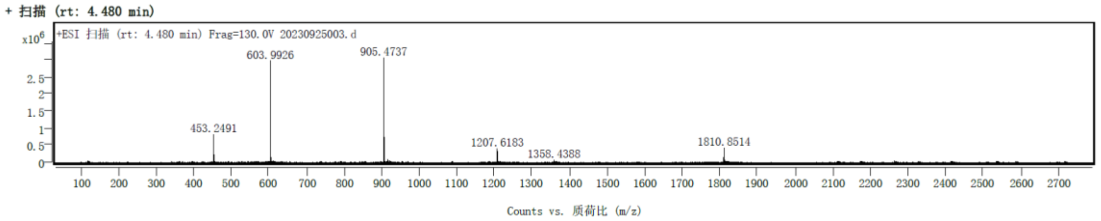

Figure S40. Q-TOF-HRMS spectrum of **1q**.

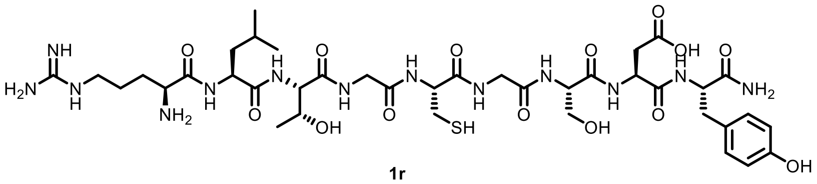

1r

1r (RLTGCGSDY) was obtained by solid phase synthesis.

Analytical **HPLC** using Method D, RT = 5.900 min, the HPLC purity is 94.34%. **HRMS** (ES+) *m/z*: [M + H]$^+$ calcd for $C_{39}H_{63}N_{13}O_{14}S^+$ 970.4411, found 970.4408.

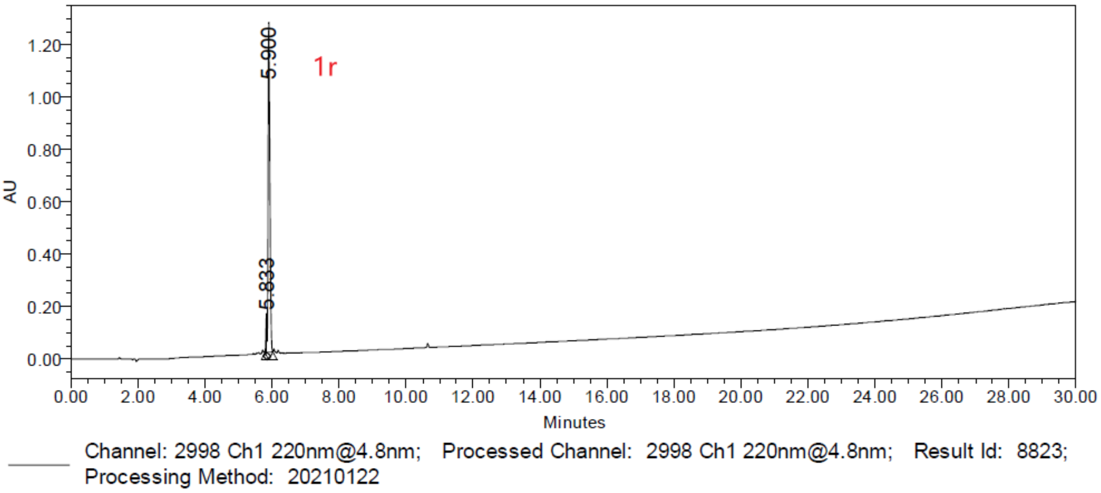

Processed Channel Descr.: 2998 Ch1 220nm@4.8nm

	Processed Channel Descr.	RT	Area	% Area	Height
1	2998 Ch1 220nm@4.8nm	5.833	277102	5.66	147586
2	2998 Ch1 220nm@4.8nm	5.900	4616660	94.34	1260101

Figure S41. HPLC-UV chromatogram at 220 nm of **1r**.

Analysis Report

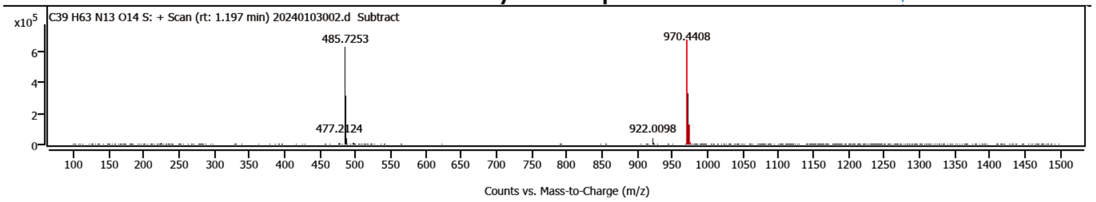

Spectrum Peaks

m/z	Z	Abund	Abund %	m/z (Calc)	Diff (ppm)	Ion Species	Formula	Ion Type
970.4408	1	667993	100.00	970.4411	-0.33	(M+H)+	C39 H63 N13 O14 S	
971.4435	1	322865	48.33	971.4438	-0.33	(M+H)+	C39 H63 N13 O14 S	
972.4424	1	117594	17.60	972.4441	-1.76	(M+H)+	C39 H63 N13 O14 S	
973.4431	1	32647	4.89	973.4451	-2.06	(M+H)+	C39 H63 N13 O14 S	
974.4439	1	7733	1.16	974.4464	-2.53	(M+H)+	C39 H63 N13 O14 S	
477.2124		7419	1.11					
485.7253	2	623175	93.29					
486.2268	2	308641	46.20					
486.7266	2	116050	17.37					
487.2259	2	35305	5.29					
487.7275	2	9023	1.35					
496.7145		7015	1.05					
922.0098		34925	5.23					

Spectrum Identification Table

Best	ID Source	Name	Formula	Species	m/z	Diff (ppm)	CAS	Score	Score (Lib)	Score (DB)	Score (MFG)	Lib/DB
Yes	MFG		C39 H63 N13 O14 S	(M+H)+	970.4408	-0.54		99.18			99.18	

Figure S42. Q-TOF-HRMS spectrum of **1r**.

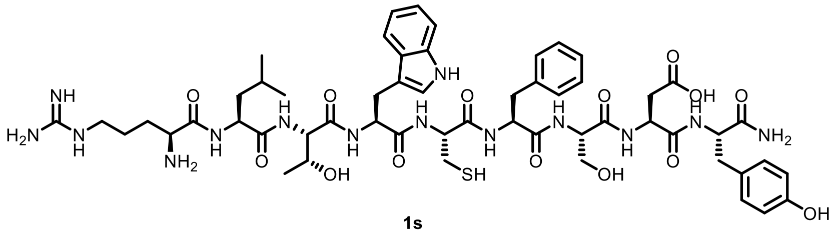

1s

1s (RLTWCFSDY) was obtained by solid phase synthesis.

Analytical **HPLC** using Method D, RT = 10.648 min, the HPLC purity is 100.00%. **HRMS** (ES+) *m*/*z*: [M + H]$^+$ calcd for $C_{55}H_{76}N_{14}O_{14}S^+$ 1189.5459, found 1189.5431.

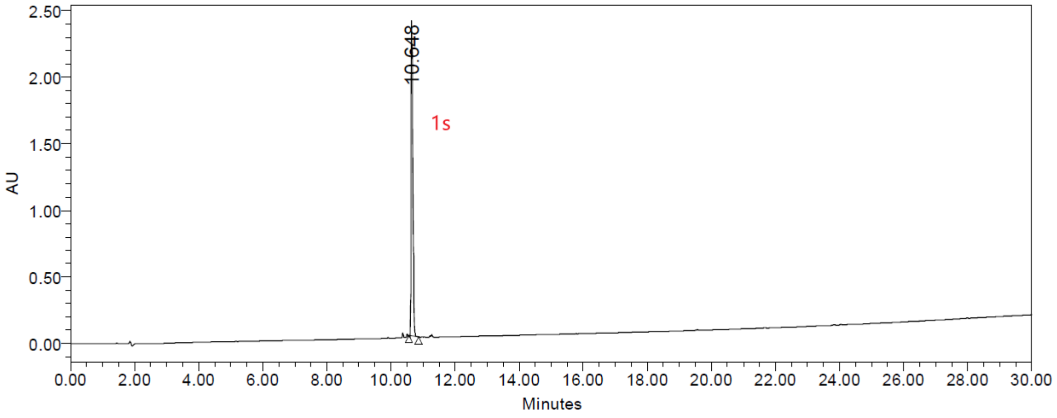

Channel: 2998 Ch1 220nm@4.8nm; Processed Channel: 2998 Ch1 220nm@4.8nm; Result Id: 8821; Processing Method: 20240122

Processed Channel Descr.: 2998 Ch1 220nm@4.8nm

	Processed Channel Descr.	RT	Area	% Area	Height
1	2998 Ch1 220nm@4.8nm	10.648	9277402	100.00	2365275

Figure S43. HPLC-UV chromatogram at 220 nm of **1s**.

Analysis Report

Agilent Trusted Answers

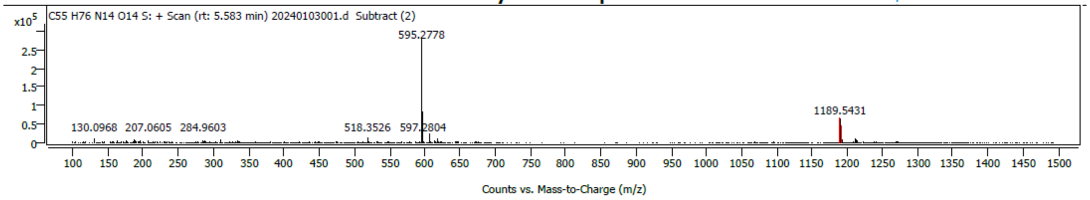

Spectrum Peaks

m/z	Z	Abund	Abund %	m/z (Calc)	Diff (ppm)	Ion Species	Formula	Ion Type
1189.5431	1	64842	23.31	1189.5459	-2.38	(M+H)+	C55 H76 N14 O14 S	
1190.5469	1	43656	15.69	1190.5488	-1.60	(M+H)+	C55 H76 N14 O14 S	
1191.5469	1	18699	6.72	1191.5500	-2.56	(M+H)+	C55 H76 N14 O14 S	
1192.5477	1	5966	2.14	1192.5511	-2.89	(M+H)+	C55 H76 N14 O14 S	
130.0968		10509	3.78					
163.0395		4273	1.54					
176.1363		3368	1.21					
187.1270		6230	2.24					
189.1165		3306	1.19					
195.0651		5045	1.81					
207.0605		3371	1.21					
284.9603		4318	1.55					
286.9578		4128	1.48					
309.2262		6211	2.23					
333.2895		5111	1.84					
518.3526	1	12106	4.35					
519.3555	1	3338	1.20					
595.2778	2	278188	100.00					
595.7792	2	189000	67.94					
596.2799	2	83056	29.86					
596.7793	2	26766	9.62					
597.2804	2	7979	2.87					
606.2680	2	24004	8.63					
606.7686	2	16759	6.02					
607.2701	2	8422	3.03					
607.7663	2	2916	1.05					
614.2513	2	5068	1.82					
614.7530	2	3096	1.11					
617.2583	2	8761	3.15					
617.7610	2	5858	2.11					
1211.5235	1	9303	3.34					
1212.5278	1	6575	2.36					
1213.5276	1	3484	1.25					

Spectrum Identification Table

Best	ID Source	Name	Formula	Species	m/z	Diff (ppm)	CAS	Score	Score (Lib)	Score (DB)	Score (MFG)	Lib/DB
Yes	MFG		C55 H76 N14 O14 S	(M+H)+	1189.5431	-2.21		95.48			95.48	

Figure S44. Q-TOF-HRMS spectrum of **1s**.

4 Chemical synthesis of functionalized 1,2,3-Triazines

1,2,3-Triazine derivatives carrying different functional groups were synthesized, as shown in Figure **S1**. The synthesis procedure mainly refer to supplemental reference[2] and the detailed steps are as follows.

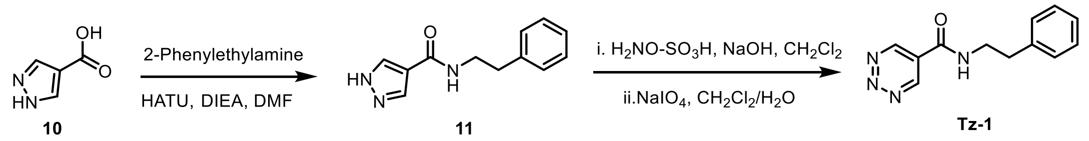

2-Phenylethylamine (3.63 g, 3.8 mL, 30 mmol) was added to a suspension of 4-pyrazolecarboxylic acid (2.24 g, 20 mmol), HATU (11.4 g, 30 mmol), and DIEA (3.87 g, 5 mL, 30 mmol) in DMF (30 mL), and stirred at room temperature for 5 h. The solvent was then evaporated to dryness, then the remaining solid was washed three times with CH_2Cl_2, acetonitrile, and water respectively. Because of its extremely poor solubility, it was used directly in the next reaction without purification. The solid was dissolved with sodium hydroxide solution (100.0 mL, 3.7 M in water), and was added solid hydroxylamine-O-sulfonic acid (60.0 mmol, 6.8 g) slowly, then the reaction system was stirred at 0 °C for 2 h. After that, the reaction mixture was extracted three times with CH_2Cl_2. The combined organic layer was washed with saturated brine, dried over with anhydrous Na_2SO_4 and concentrated in vacuo. The crude product was dissolved in a mixed solution of CH_2Cl_2 (60.0 mL)/H_2O (20 mL), then $NaIO_4$ (40.0 mmol, 8.56 g) was slowly added and the reaction system was stirred at 0 °C for 12.0 h. The reaction mixture was extracted three times with CH_2Cl_2. The combined organic layer was washed with saturated brine, dried over with anhydrous Na_2SO_4 and concentrated in vacuo. The crude residue was purified through column chromatography on silica gel with PE/EA (1:1) as the eluent to afford compound **Tz-1** (513 mg, 11.3% yield).

^{1}H NMR (300 MHz, Chloroform-*d*) δ 9.25 (d, J = 1.5 Hz, 2H), 7.34 (dd, J = 8.5, 6.8 Hz, 2H), 7.24 (m, 3H), 6.71 (s, 1H), 3.80 (q, J = 6.6 Hz, 2H), 2.98 (t, J = 6.9 Hz, 2H). **^{13}C NMR** (75 MHz, $CDCl_3$) δ 161.40, 146.68, 137.92, 128.97, 128.73, 127.06, 122.76, 41.53, 35.20. **LRMS** (ESI+) *m/z*: 229.16 $[M + H]^+$, (ESI-) *m/z*: 227.58 $[M - H]^-$.

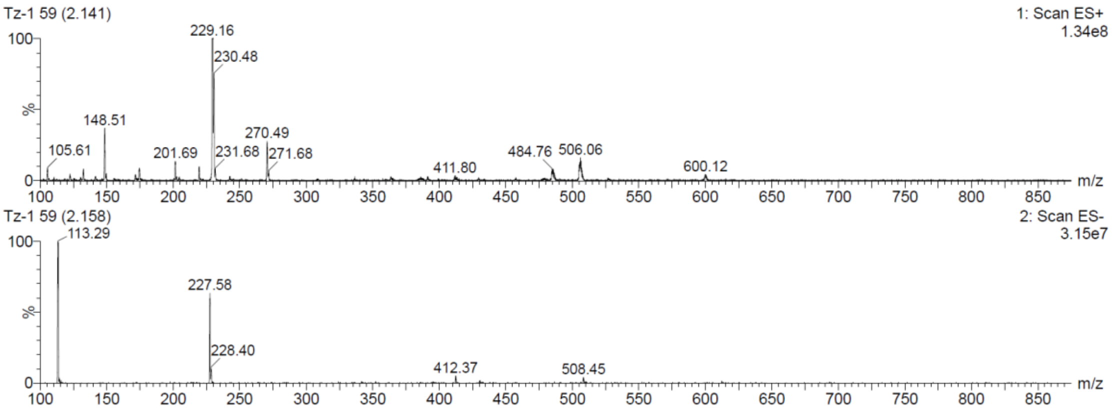

Figure S45. ESI-MS spectrum of **Tz-1**.

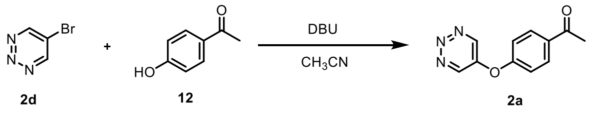

p-Hydroxyacetophenone (1 mmol, 136 mg) and 5-bromo-1,2,3-triazine (1 mmol, 159 mg) were added to a 25 mL flame-dried round-bottom flask equipped with a stir bar. 10 mL acetonitrile was added to dissolve, and then DBU (2 mmol, 304 mg, 2 eq.) was added. The reaction system was stirred at room temperature for 1.0 h. After triazine was completely consumed, monitored by LCMS analysis, the solvent was removed in vacuo. The crude residue was directly purified through column chromatography on a silica gel with PE/EA (1:1) as the eluent to afford the desired product **2a** (161 mg, 75% yield).

^{1}H NMR (700 MHz, Methanol-*d*$_4$) δ 8.87 (s, 2H), 8.07 (d, *J* = 8.8 Hz, 2H), 7.30 (d, *J* = 8.8 Hz, 2H), 2.54 (s, 3H). **^{13}C NMR** (176 MHz, Methanol-*d*$_4$) δ 198.76, 157.34, 152.17, 141.73, 136.85, 132.47, 121.66, 26.76. Spectroscopic data was consistent with the values reported in literature[2a]. **LRMS** (ESI+) *m/z*: 216.14 [M + H]$^+$.

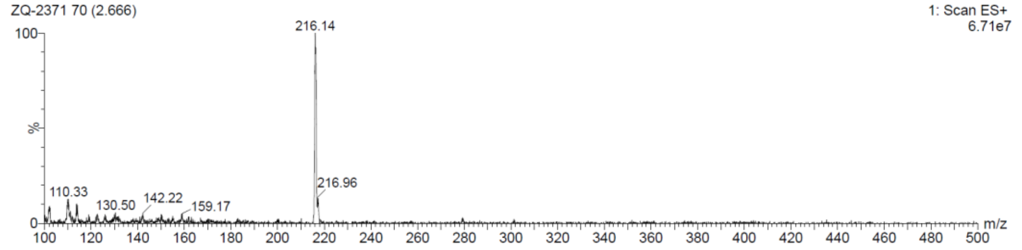

Figure S46. ESI-MS spectrum of **2a**.

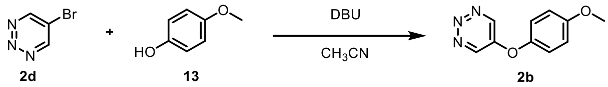

4-Methoxyphenol (1.5 mmol, 273 mg, 1.5 eq.) and 5-bromo-1,2,3-triazine (1 mmol, 160 mg) were added to a 25 mL flame-dried round-bottom flask equipped with a stir bar. 10 mL acetonitrile was added to dissolve, and then DBU (2 mmol, 304 mg, 2 eq.) was added. The reaction system was stirred at room temperature for 1.0 h. After triazine was completely consumed, monitored by LCMS analysis, the solvent was removed in vacuo. The crude residue was directly purified through column chromatography on a silica gel with PE/EA (2:1) as the eluent to afford the desired product **2b** (187 mg, 92% yield).

^{1}H NMR (300 MHz, Chloroform-*d*) δ 8.73 (s, 2H), 7.06 (m, 2H), 7.00 (m, 2H), 3.84 (s, 3H). **^{13}C NMR** (75 MHz, Chloroform-*d*) δ 158.33, 151.45, 145.07, 139.60, 121.61, 115.90, 55.87. The data was consistent with the values reported in literature[2a]. **LRMS** (ESI+) *m/z*: 204.14 [M + H]$^+$.

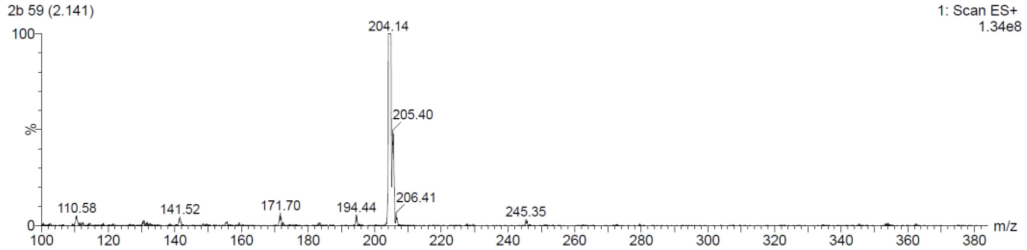

Figure S47. ESI-MS spectrum of **2b**.

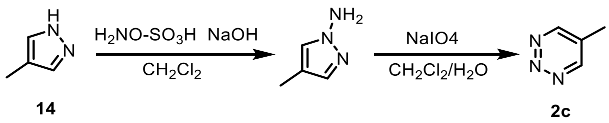

Solid hydroxylamine-O-sulfonic acid (45.0 mmol, 5.1 g) was slowly added to a solution of 4-methyl-1H-pyrazole (15.0 mmol, 1.23 g) in 3.7 M NaOH (75.0 mL), then the reaction system was stirred at 0 °C for 2 h. After that, the reaction mixture was extracted three times with CH_2Cl_2. The combined organic layer was washed with saturated brine, dried over with anhydrous Na_2SO_4 and concentrated in vacuo. The crude product was dissolved in a mixed solution of CH_2Cl_2 (60.0 mL)/H_2O (20 mL), then $NaIO_4$ (30.0 mmol, 6.42 g) was slowly added and the reaction system was stirred at 0 °C for 12.0 h. The reaction mixture was extracted three times with CH_2Cl_2. The combined organic layer was washed with saturated brine, dried over with anhydrous Na_2SO_4 and concentrated in vacuo. The crude residue was purified through column chromatography on silica gel with PE/EA (2:1) as the eluent to afford compound **2c** (322 mg, 23% yield).

^{1}H NMR (300 MHz, Chloroform-*d*) δ 8.92 (s, 2H), 2.38 (s, 3H). **^{13}C NMR** (75 MHz, $CDCl_3$) δ 151.05, 129.31, 16.09. The data was consistent with the values reported in literature[2b].

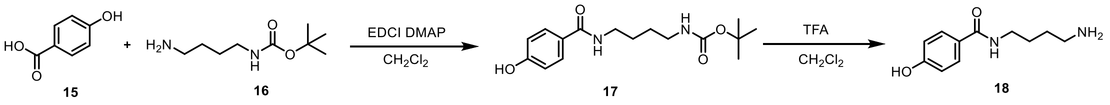

Compound 17

Add 1-ethyl-(3-(3-dimethylamino)propyl)-carbodiimide hydrochloride (2.86 g, 15 mmol) which dissolved in anhydrous CH_2Cl_2 (20 mL) to a stirred solution of 4-hydroxybenzoic acid (1.38 g, 10 mmol), N-Boc-1,4-diaminobutane hydrochloride (2.68 g, 12 mmol) and dimethylamino-pyridine (1.83 g, 15 mmol) in anhydrous CH_2Cl_2 (20 mL) at 0 °C. Stir the reaction mixture for 5 min and then at room temperature for 3 h. Extract the reaction mixture with CH_2Cl_2 and water. Wash the reaction mixture to neutral with saturated NaCl solution. Dry the organic layer over anhydrous magnesium sulfate. Filter the organic layer. Evaporate the solvent at reduced pressure to obtain light yellow solid. Purify the solid by silica gel chromatography with a gradient elution of CH_2Cl_2/MeOH (20:1) to obtain **17** (2.46 g, 80% yield).

^{1}H NMR (700 MHz, Methanol-*d*$_4$) δ 7.59 (d, *J* = 8.7 Hz, 2H), 6.71 (d, *J* = 8.7 Hz, 2H), 3.25 (t, *J* = 7.0 Hz, 2H), 2.96 (t, *J* = 6.9 Hz, 2H), 1.50 (m, 2H), 1.43 (m, 2H), 1.32 (s, 9H). **^{13}C NMR** (176 MHz, Methanol-*d*$_4$) δ 168.68, 160.56, 157.19, 128.81, 125.19, 114.65, 78.48, 39.64, 39.18, 27.41, 27.07, 26.50. **LRMS** (ESI+) *m/z*: 309.35 $[M + H]^+$, (ESI-) *m/z*: 307.33 $[M - H]^-$.

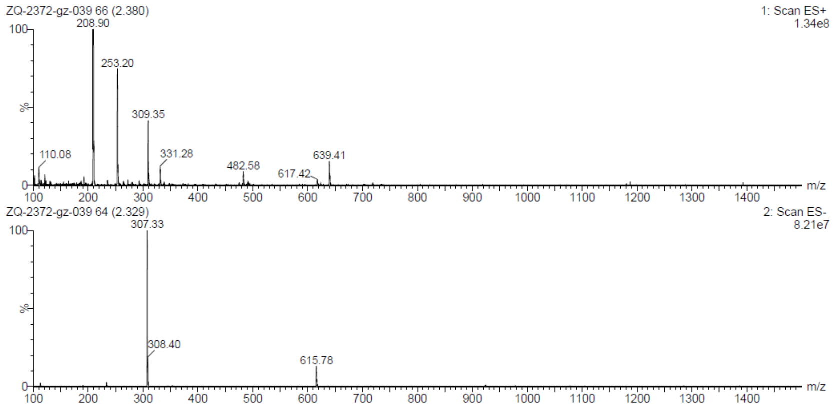

Figure S48. ESI-MS spectrum of **17**.

Compound 18

17 (0.2 mmol) was dissolved in CH_2Cl_2 (2 mL), cooled to 0°C, then TFA (2 mL) was added. After the consumption of the starting material (1 h, monitored by TLC), the mixture was evaporated and then saturated aqueous $NaHCO_3$ was added. The aqueous layer was extracted twice with CH_2Cl_2 (15 mL), and the organic layer was washed with brine and dried over anhydrous Na_2SO_4. The solvent was removed under vacuum, to afford the **18**, which were employed without further purification. The data was consistent with the values reported in literature[3].

^{1}**H NMR** (700 MHz, Methanol-d_4) δ 7.60 (d, J = 8.7 Hz, 2H), 6.72 (d, J = 8.7 Hz, 2H), 3.30 (t, J = 6.5 Hz, 2H), 2.88 (m, 2H), 1.60 (m, 4H). ^{13}**C NMR** (176 MHz, Methanol-d_4) δ 170.21, 162.15, 130.19, 126.29, 116.08, 40.41, 39.85, 27.67, 25.91. **LRMS** (ESI+) m/z: 209.59 [M + H]$^+$.

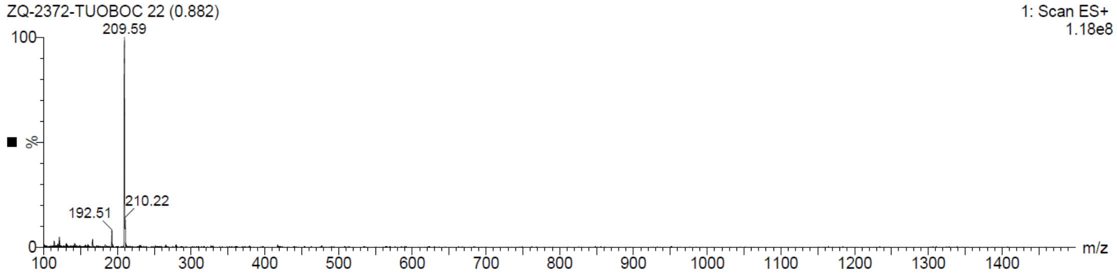

Figure S49. ESI-MS spectrum of **18**.

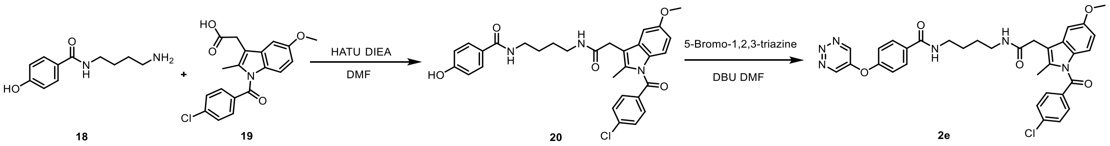

Compound 20

18 (358 mg, 1.5 mmol) was added to a suspension of Indomethacin (358 mg, 1 mmol), HATU (570 mg, 1.5 mmol), and DIEA (387 mg, 530 uL, 3 mmol) in DMF (10 mL), and stirred at room temperature for 10 h. Then, CH_2Cl_2 and water were added. The separated aqueous phase was extracted with CH_2Cl_2 (3 × 40 mL). The combined organic phases were washed with water, dried with Na_2SO_4, and evaporated to dryness, followed by purification by RP-HPLC to give the white solid **20** (412 mg, 75% yield).

^{1}**H NMR** (700 MHz, DMSO-d_6) δ 9.93 (s, 1H), 8.19 (t, J = 5.7 Hz, 1H), 8.06 (t, J = 5.7 Hz, 1H), 7.68 (ddd, J = 9.0, 4.8, 2.2 Hz, 4H), 7.64 (m, 2H), 7.11 (d, J = 2.6 Hz, 1H), 6.93 (d, J = 9.0 Hz, 1H), 6.77 (m, 2H), 6.70 (dd, J = 9.0, 2.6 Hz, 1H), 3.73 (s, 3H), 3.48 (s, 2H), 3.20 (q, J = 6.6 Hz, 2H), 3.07 (q, J = 6.5 Hz, 2H), 2.22 (s, 3H), 1.45 (m, 4H). ^{13}**C NMR** (176 MHz, DMSO) δ 169.25, 167.89, 165.82, 159.96, 155.56, 137.57, 135.12, 134.31, 131.19, 130.95, 130.28, 129.07, 129.04, 125.38, 114.72, 114.60, 114.50, 111.31, 101.83, 55.42, 38.74, 38.56, 31.21, 26.82, 26.75, 13.44. **LRMS** (ESI+) m/z: 548.17 [M + H]$^+$, (ESI-) m/z: 546.41 [M - H]$^-$.

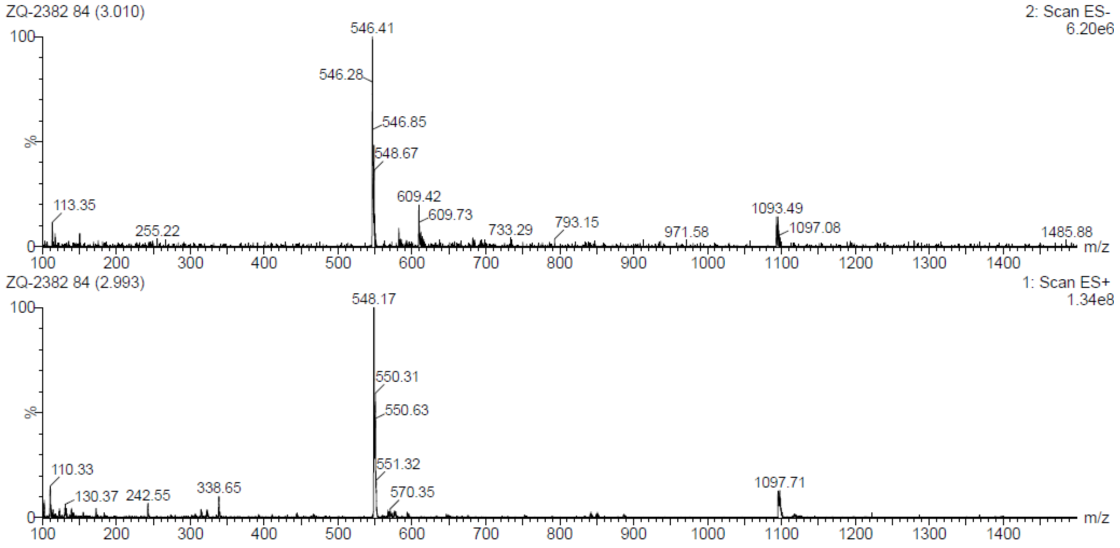

Figure S50. ESI-MS spectrum of **20**.

Compound 2e

20 (0.25 mmol, 135 mg) and 5-bromo-1,2,3-triazine (0.27 mmol, 36.9 mg, 1 eq.) were added to a 10 mL flame-dried round-bottom flask equipped with a stir bar. 3 mL DMF was added to dissolve, then DBU (0.5 mmol, 75 mg, 2 eq.) was added. The reaction system was stirred at room temperature for 1.0 h. After triazine was completely consumed, monitored by LCMS analysis, the solvent was removed in vacuo. The crude residue was directly purified through RP-HPLC to afford the desired product **2e** (86 mg, 55% yield).

^{1}H NMR (700 MHz, Chloroform-*d*) δ 8.80 (s, 2H), 7.99 (d, J = 8.4 Hz, 2H), 7.67 (m, 2H), 7.49 (m, 2H), 7.20 (d, J = 8.3 Hz, 2H), 6.90 (d, J = 2.5 Hz, 1H), 6.85 (d, J = 9.1 Hz, 2H), 6.68 (dd, J = 9.0, 2.5 Hz, 1H), 5.88 (t, J = 6.2 Hz, 1H), 3.79 (s, 3H), 3.67 (s, 2H), 3.47 (q, J = 6.1 Hz, 2H), 3.29 (q, J = 6.3 Hz, 2H), 2.39 (s, 3H), 1.56 (m, 4H). **^{13}C NMR** (176 MHz, $CDCl_3$) δ 170.62, 168.48, 166.15, 156.27, 154.13, 150.50, 139.93, 139.77, 136.54, 133.59, 133.57, 131.31, 131.07, 130.45, 130.20, 129.35, 120.40, 115.22, 112.87, 112.12, 101.29, 55.94, 39.91, 39.14, 32.34, 27.39, 26.14, 13.42. **LRMS** (ESI+) *m*/*z*: 627.44 $[M + H]^+$, (ESI-) *m*/*z*: 625.65 $[M - H]^-$.

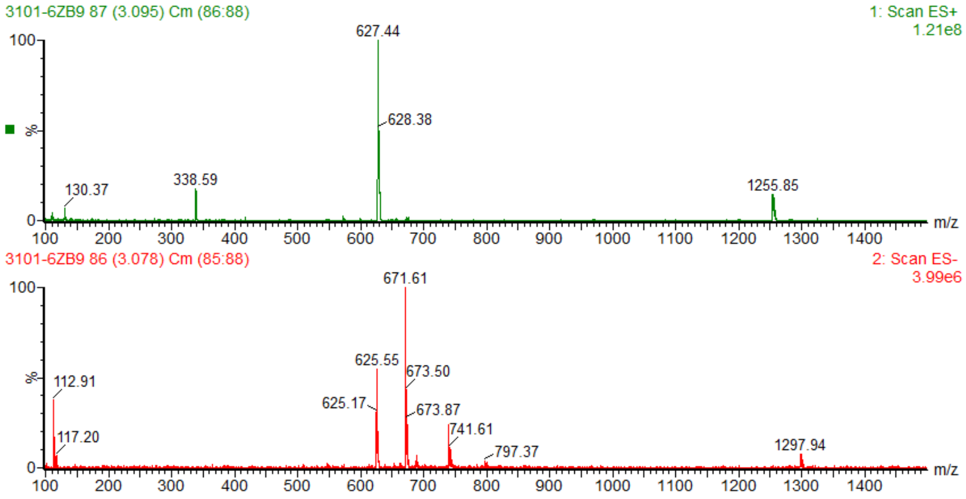

Figure S51. ESI-MS spectrum of **2e**.

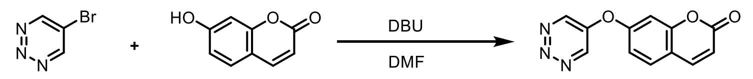

2d 21 2f

Compound 2f

7-Hydroxycoumarin (0.5 mmol, 81 mg) and 5-bromo-1,2,3-triazine (0.55 mmol, 88 mg, 1.5 eq.) were added to a 10 mL flame-dried round-bottom flask equipped with a stir bar. 3 mL DMF was added to dissolve, then DBU (1 mmol, 152 mg, 152 uL, 2 eq.) was added. The reaction system was stirred at room temperature for 1.0 h. After triazine was completely consumed, monitored by LCMS analysis, the solvent was removed in vacuo. The crude residue was directly purified through RP-HPLC to afford the desired product **2f** (80 mg, 66% yield).

^{1}**H NMR** (700 MHz, Chloroform-*d*) δ 8.84 (s, 2H), 7.75 (d, *J* = 9.6 Hz, 1H), 7.64 (d, *J* = 8.4 Hz, 1H), 7.16 (d, *J* = 2.3 Hz, 1H), 7.07 (dd, *J* = 8.4, 2.3 Hz, 1H), 6.49 (d, *J* = 9.6 Hz, 1H).^{13}**C NMR** (176 MHz, $CDCl_3$) δ 159.64, 155.70, 154.42, 150.14, 142.45, 140.01, 130.35, 117.81, 117.23, 116.46, 109.34. LRMS (ESI+) *m*/*z*: 241.73 $[M + H]^{+}$.

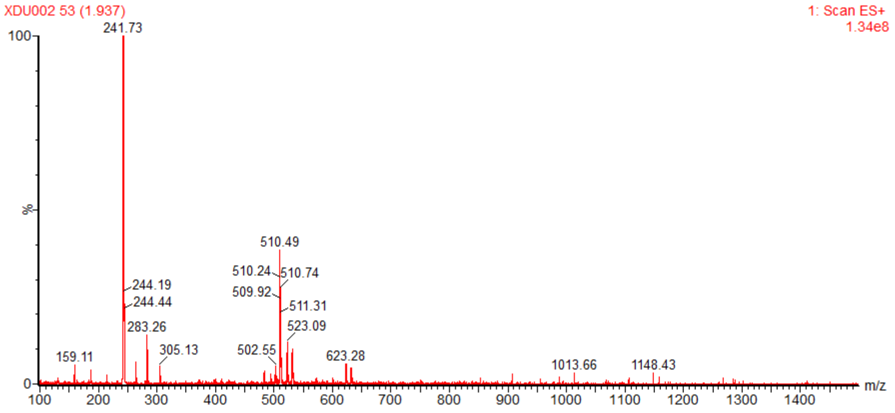

Figure S52. ESI-MS spectrum of **2f**.

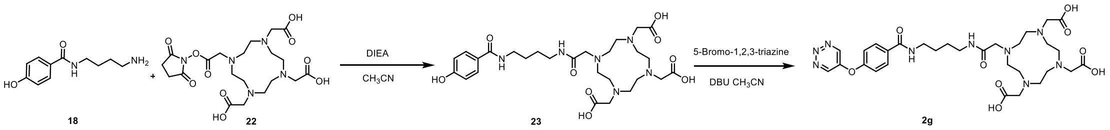

Compound 23

To a solution of **18** (41.6 mg, 0.2 mmol) in dry CH_3CN (5 mL) was added DIEA (132 uL, 0.8 mmol). The mixture was stirred until dissolved. DOTA-NHS-ester (100 mg, 0.2 mmol) was added to the reaction mixture and stirred for 12 h at room temperature (HPLC monitored completion of reaction). Upon completion of the reaction, the solvent was evaporated and the crude product was purified by RP-HPLC to afford a white solid **23** (35.6 mg, 30%).

^{1}**H NMR** (700 MHz, Deuterium Oxide) δ 7.58 (d, *J* = 8.7 Hz, 2H), 6.85 (d, *J* = 8.7 Hz, 2H), 3.63 (s, 9H), 3.27 (t, *J* = 6.6 Hz, 4H), 3.14 (s, 15H), 1.50 (m, 4H). ^{13}**C NMR** (176 MHz, Deuterium Oxide) δ 170.15, 163.05, 162.85, 159.03, 129.23, 125.56, 117.11, 115.46, 115.33, 55.11, 39.38, 39.13, 25.90, 25.71. **LRMS** (ESI+) *m*/*z*: 595.81 $[M + H]^{+}$, (ESI-) *m*/*z*: 593.66 $[M - H]^{-}$.

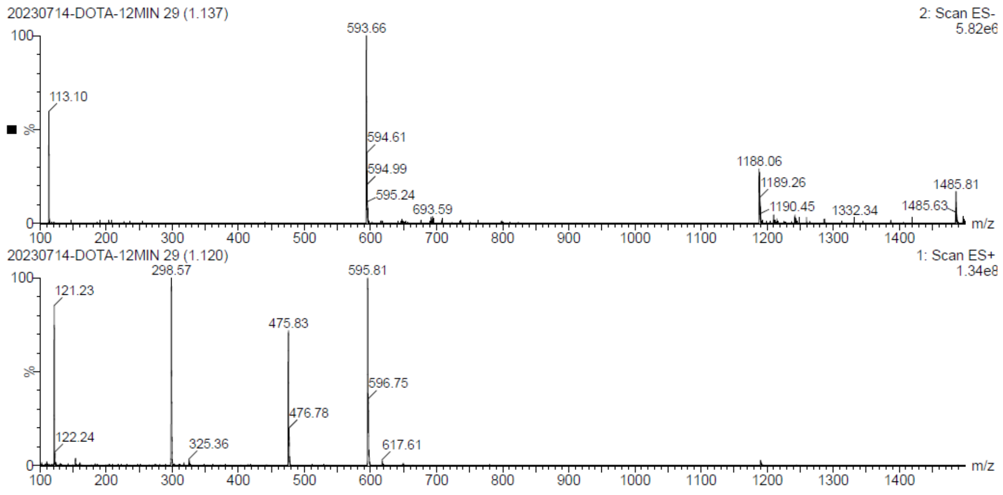

Figure S53. ESI-MS spectrum of **23**.

Compound 2g

23 (0.24 mmol, 143 mg) and 5-bromo-1,2,3-triazine (0.27 mmol, 43 mg, 1.1 eq.) were added to a 10 mL flame-dried round-bottom flask equipped with a stir bar. 3 mL CH_3CN was added to dissolve, then DBU (0.48 mmol, 74 mg, 2 eq.) was added. The reaction system was stirred at room temperature for 1.0 h. After **23** was completely consumed, monitored by LCMS analysis, the solvent was removed in vacuo. The crude residue was directly purified through RP-HPLC to afford the desired product **2g** (51 mg, 31% yield).

LRMS (ESI+) *m/z*: 674.31 $[M + H]^+$, (ESI-) *m/z*: 672.68 $[M - H]^-$. **HRMS** (ES+) *m/z*: $[M + H]^+$ calcd for $C_{30}H_{43}N_9O_9^+$ 674.3263, found 674.3260. Analytical **HPLC** using Method D, RT = 6.17 min, the purity is 95.68%.

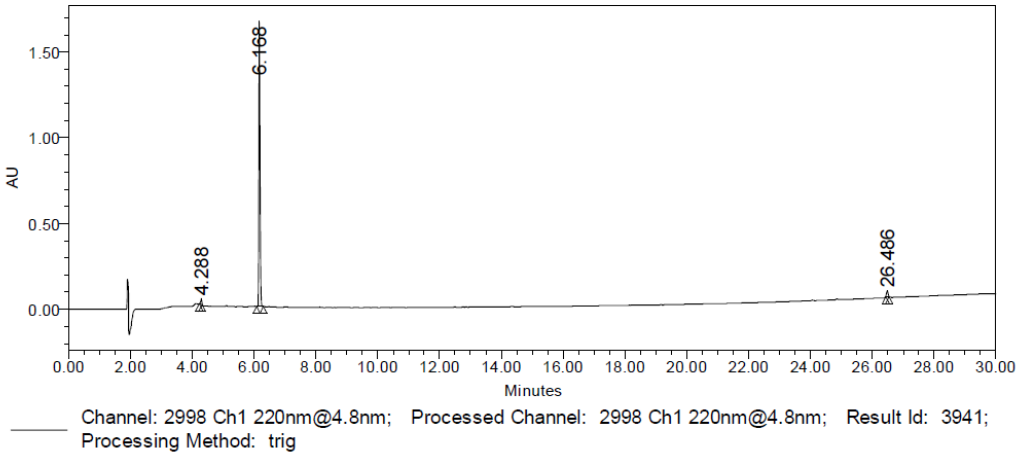

Processed Channel Descr.: 2998 Ch1 220nm@4.8nm

	Processed Channel Descr.	RT	Area	% Area	Height
1	2998 Ch1 220nm@4.8nm	4.288	80688	1.82	33098
2	2998 Ch1 220nm@4.8nm	6.168	4231411	95.68	1665158
3	2998 Ch1 220nm@4.8nm	26.486	110224	2.49	32001

Figure S54. HPLC-UV chromatogram at 220 nm of **2g**.

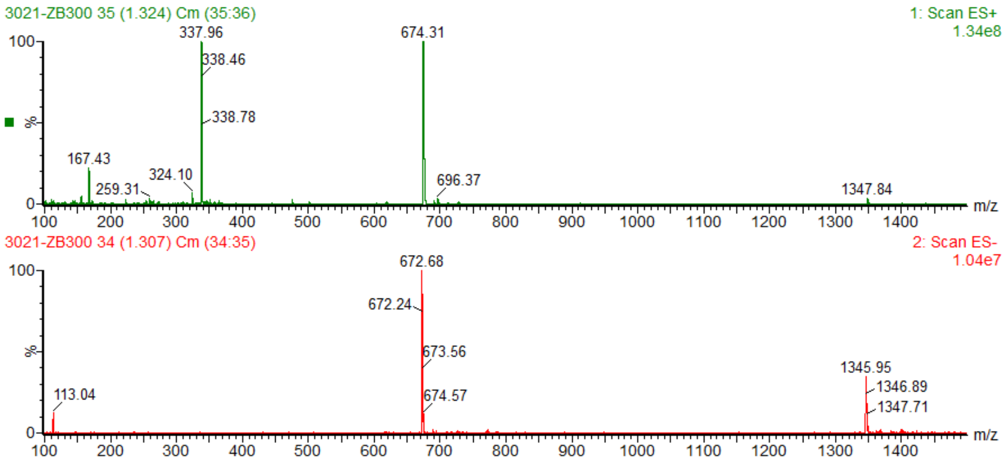

Figure S55. ESI-MS spectrum of **2g**.

Analysis Report

Agilent Trusted Answers

Chromatogram Peaks

Peak	Start	RT	End	Height	Area	Area %	SNR
1	3.612	3.725	4.030	589953	2836914	100.00	

Sample Spectra

+ Scan (rt: 3.757 min) **C30 H43 N9 O9**

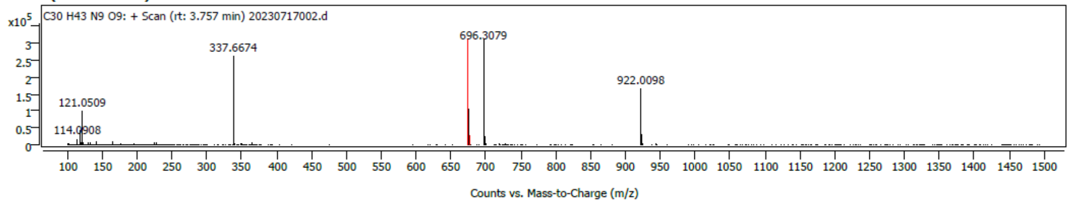

Spectrum Peaks

m/z	Z	Abund	Abund %	m/z (Calc)	Diff (ppm)	Ion Species	Formula	Ion Type
674.3260	1	308560	99.35	674.3257	0.45	(M+H)+	C30 H43 N9 O9	
675.3275	1	106277	34.22	675.3285	-1.43	(M+H)+	C30 H43 N9 O9	
676.3284	1	23286	7.50	676.3310	-3.87	(M+H)+	C30 H43 N9 O9	
114.0908		15700	5.06					
118.0868		43278	13.93					
121.0509		99129	31.92					
337.6674	2	261639	84.24					
338.1689	2	92858	29.90					
338.6693	2	20836	6.71					
696.3079	1	310575	100.00					
697.3101	1	103291	33.26					
698.3088	1	25757	8.29					
922.0098	1	167048	53.79					
923.0111	1	32032	10.31					

Spectrum Identification Table

Best	ID Source	Name	Formula	Species	m/z	Diff (ppm)	CAS	Score	Score (Lib)	Score (DB)	Score (MFG)	Lib/DB
Yes	MFG		C30 H43 N9 O9	(M+H)+	674.3260	-0.25		97.33			97.33	
No	MFG		C31 H49 N2 O14	(M+H)+	674.3260	-0.47		96.31			96.31	
No	MFG		C29 H47 N5 O13	(M+H)+	674.3260	1.63		95.54			95.54	
No	MFG		C32 H45 N6 O10	(M+H)+	674.3260	-2.35		92.71			92.71	
No	MFG		C27 H45 N8 O12	(M+H)+	674.3260	3.73		89.44			89.44	

Figure S56. Q-TOF-HRMS spectrum of **2g**.

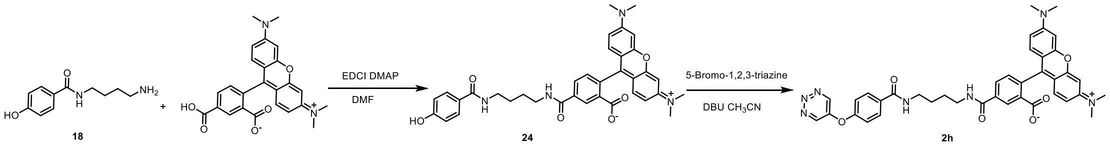

Compound 24

Add EDCI (114 mg, 0.6 mmol, 1.2 eq.) which dissolved in DMF (5 mL) to a stirred solution of 5-carboxytetramethylrhodamine (215 mg, 0.5 mmol), **18** (161 mg, 0.5 mmol) and DMAP (122 mg, 1 mmol, 2 eq.) in DMF (5 mL) at 0 °C. Stir the reaction mixture for 5 min and then at room temperature for 3 h. The solvent was removed in vacuo and the crude product was purified by prep RP-HPLC to obtain red solid **24** (248 mg, 80% yield).

^{1}H NMR (700 MHz, Methanol-*d*$_4$) δ 8.80 (d, *J* = 1.8 Hz, 1H), 8.28 (dd, *J* = 7.9, 1.9 Hz, 1H), 7.73 (d, *J* = 8.7 Hz, 2H), 7.54 (d, *J* = 7.9 Hz, 1H), 7.15 (d, *J* = 9.5 Hz, 2H), 7.08 (dd, *J* = 9.5, 2.5 Hz, 2H), 7.00 (d, *J* = 2.5 Hz, 2H), 6.83 (d, *J* = 8.7 Hz, 2H), 3.54 (t, *J* = 6.6 Hz, 2H), 3.46 (t, *J* = 6.6 Hz, 2H), 3.32 (s, 12H), 1.77 (tddd, *J* = 8.6, 7.1, 4.4, 2.5 Hz, 4H). **^{13}C NMR** (176 MHz, Methanol-*d*$_4$) δ 170.13, 168.17, 167.37, 162.00, 160.72, 159.06, 158.98, 138.06, 137.79, 132.84, 132.30, 131.95, 131.92, 131.29, 130.21, 126.54, 116.04, 115.57, 114.74, 97.45, 40.93, 40.52, 28.17, 27.86. **LRMS** (ESI+) *m/z*: 621.01 $[M + H]^{+}$, (ESI-) *m/z*: 619.62 $[M - H]^{-}$.

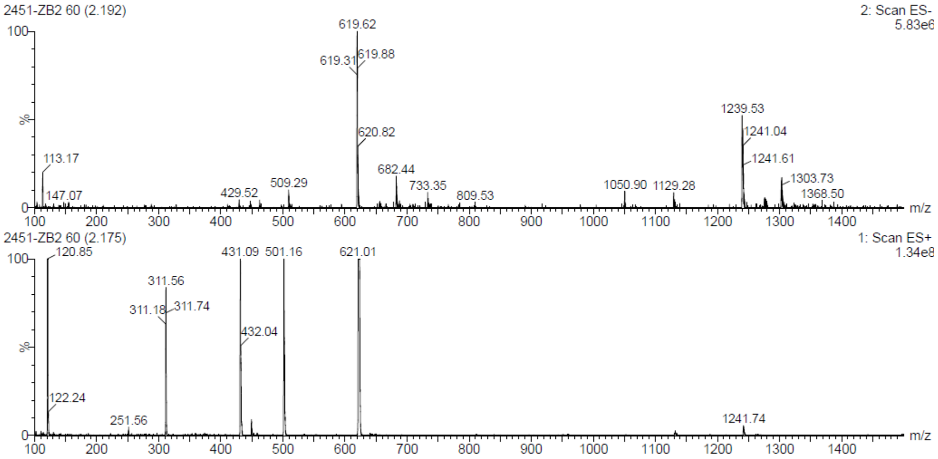

Figure S57. ESI-MS spectrum of **24**.

Compound 2h

24 (0.083 mmol, 51.9 mg) and 5-bromo-1,2,3-triazine (0.083 mmol, 14 mg, 1 eq.) were added to a 10 mL flame-dried round-bottom flask equipped with a stir bar. 3 mL CH_3CN was added to dissolve, then DBU (0.1245 mmol, 20 mg, 1.5 eq.) was added. The reaction system was stirred at room temperature for 1.0 h. After **24** was completely consumed, monitored by LCMS analysis, the solvent was removed in vacuo. The crude residue was directly purified through semi prep RP-HPLC to afford the desired red product **2h** (39.6 mg, 68.3% yield).

^{1}H NMR (700 MHz, Chloroform-*d*) δ 8.80 (s, 1H), 8.72 (s, 2H), 8.43 (t, *J* = 5.7 Hz, 1H), 8.20 (d, *J* = 7.8 Hz, 1H), 8.00 (d, *J* = 8.2 Hz, 2H), 7.85 (t, *J* = 5.9 Hz, 1H), 7.15 (d, *J* = 7.9 Hz, 1H), 7.02 (d, *J* = 8.2 Hz, 2H), 6.78 (d, *J* = 9.0 Hz, 2H), 6.49 (dd, *J* = 9.1, 2.5 Hz, 2H), 6.44 (d, *J* = 2.5 Hz, 2H), 3.43 (q, *J* = 6.8, 6.3 Hz, 2H), 3.27 (q, *J* = 6.2 Hz, 2H), 3.04 (s, 12H), 1.65 (dt, *J* = 36.2, 7.2 Hz, 4H). **^{13}C NMR** (176 MHz, $CDCl_3$) δ 169.50, 166.41, 165.85, 154.78, 154.15, 153.70, 150.55, 139.87, 136.52, 133.56, 132.73, 130.46, 130.04, 130.01, 126.30, 125.90, 120.09, 110.76, 109.22, 97.59, 77.34, 77.16, 76.98, 40.54, 39.65, 39.58, 26.75, 26.26. **LRMS** (ESI+) *m/z*: 700.59 $[M + H]^{+}$.

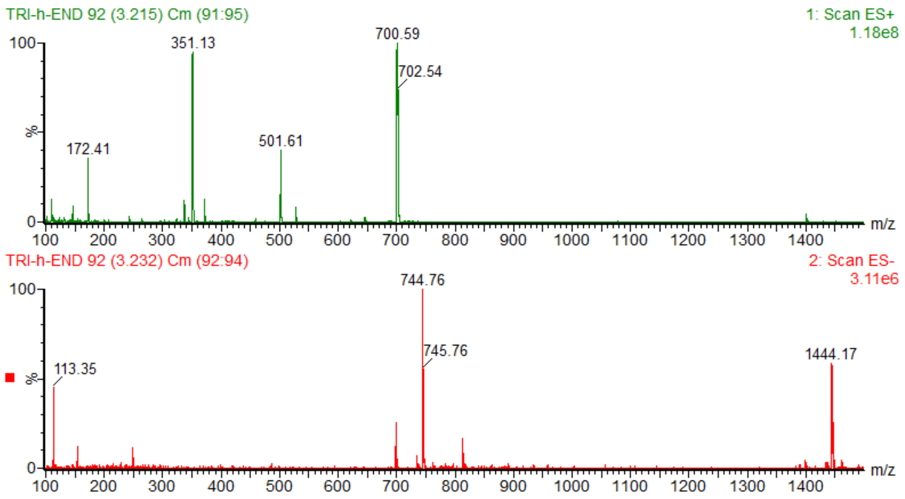

Figure S58. ESI-MS spectrum of **2h**.

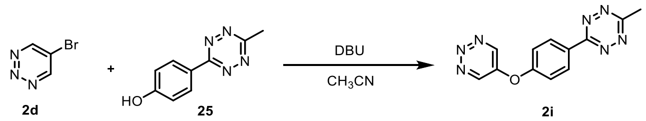

Compound 2i

25 (0.53 mmol, 100 mg) and 5-bromo-1,2,3-triazine (0.53 mmol, 72.1 mg, 1 eq.) were added to a 10 mL flame-dried round-bottom flask equipped with a stir bar. 5 mL CH_3CN was added to dissolve, then DBU (1.06 mmol, 161 mg, 2 eq.) was added. The reaction system was stirred at room temperature for 1.0 h. The solvent was removed in vacuo and the crude residue was directly purified through semi prep RP-HPLC to afford the desired red product **2i** (58 mg, 40.9% yield).

^{1}**H NMR** (700 MHz, Chloroform-*d*) δ 8.87 (s, 2H), 8.77 (d, *J* = 8.7 Hz, 2H), 7.39 (d, *J* = 8.8 Hz, 2H), 3.13 (s, 3H). ^{13}**C NMR** (176 MHz, $CDCl_3$) δ 167.79, 163.16, 155.39, 140.07, 130.85, 130.83, 121.25, 21.38. **LRMS** (ESI+) *m*/*z*: 268.39 $[M + H]^+$.

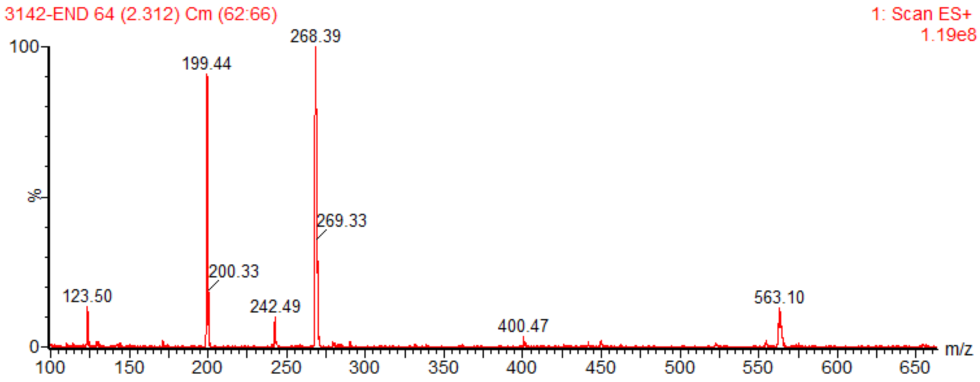

Figure S59. ESI-MS spectrum of **2i**.

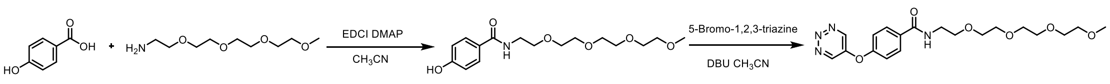

15 26 27 2j

Compound 27

Add EDCI (191 mg, 1.5 mmol, 1.5 eq.) which dissolved in DMF (3 mL) to a stirred solution of 4-hydroxybenzoic acid (217 mg, 1 mmol), 3,6,9,12-tetraoxatridecylamine (310 mg, 1.5 mmol, 1.5 eq.) and DMAP (183 mg, 1.5 mmol, 1.5 eq.) in CH_3CN (5 mL) at 0 °C. Stir the reaction mixture for 5 min and then at room temperature for 3 h. The solvent was removed in vacuo and the crude product was purified by prep RP-HPLC to obtain transparent oily liquid **27** (118.7 mg, 36.3% yield).

^{1}H NMR (700 MHz, Deuterium Oxide) δ 7.57 (d, *J* = 8.8 Hz, 2H), 6.82 (d, *J* = 8.8 Hz, 2H), 3.59 (m, 4H), 3.55 (m, 2H), 3.51 (m, 2H), 3.45 (m, 6H), 3.40 (m, 2H), 3.20 (s, 3H). **^{13}C NMR** (176 MHz, Deuterium Oxide) δ 170.15, 159.14, 129.30, 125.25, 115.28, 70.85, 69.53, 69.50, 69.44, 69.43, 69.31, 68.84, 57.93, 39.39. **LRMS** (ESI+) *m/z*: 327.88 $[M + H]^+$, (ESI-) *m/z*: 326.49 $[M - H]^-$.

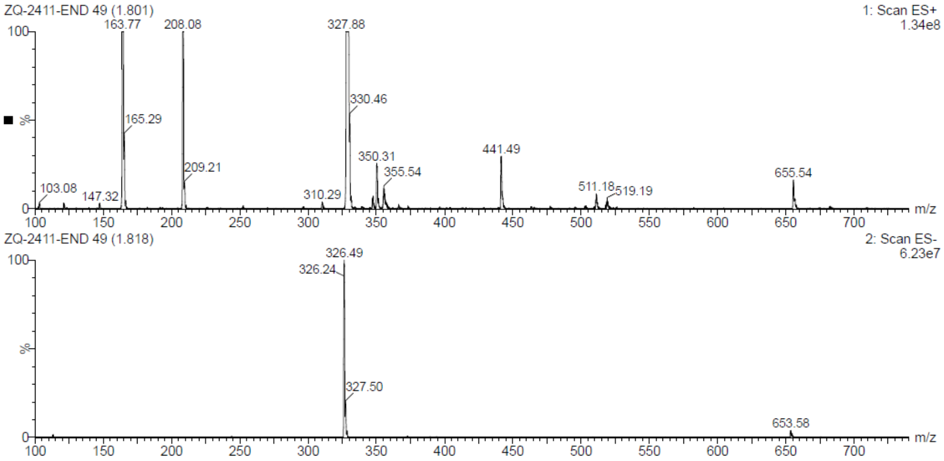

Figure S60. ESI-MS spectrum of **27**.

Compound 2j

27 (0.08 mmol, 26.2 mg) and 5-bromo-1,2,3-triazine (0.08 mmol,12.8 mg, 1 eq.) were added to a 10 mL flame-dried round-bottom flask equipped with a stir bar. 3 mL CH_3CN was added to dissolve, then DBU (0.12 mmol, 18.2 mg, 1.5 eq.) was added. The reaction system was stirred at room temperature for 1.0 h. The solvent was removed in vacuo and the crude residue was directly purified through HPLC to afford the desired product **2j** (9.8 mg, 29.8% yield).

^{1}H NMR (700 MHz, Chloroform-*d*) δ 8.79 (s, 2H), 8.04 (d, *J* = 8.5 Hz, 2H), 7.47 (s, 1H), 7.20 (m, 2H), 3.69 (s, 4H), 3.67 (m, 6H), 3.64 (dd, *J* = 6.3, 3.5 Hz, 2H), 3.60 (m, 2H), 3.52 (m, 2H), 3.31 (s, 3H). **^{13}C NMR** (176 MHz, $CDCl_3$) δ 166.13, 154.10, 150.55, 139.92, 133.80, 130.44, 120.33, 59.02, 40.15. **LRMS** (ESI+) *m/z*: 407.02 $[M + H]^+$, (ESI-) *m/z*: 405.45 $[M - H]^-$.

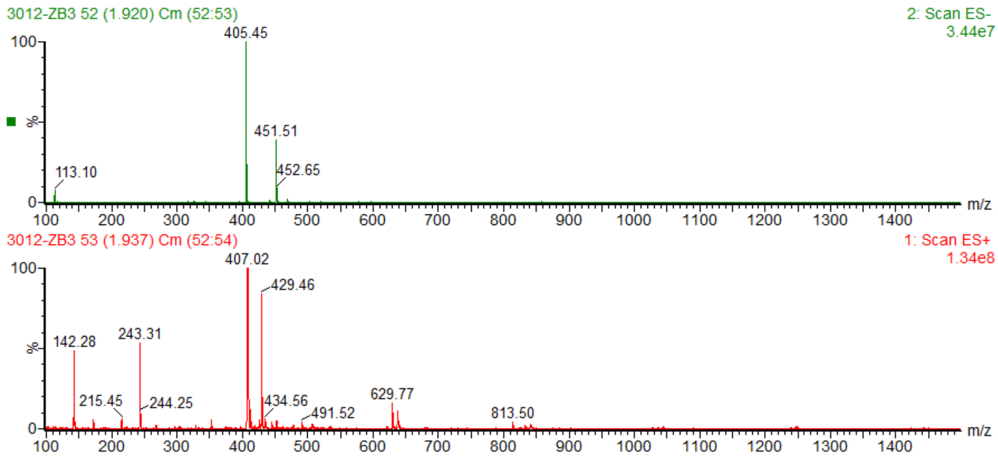

Figure S61. ESI-MS spectrum of **2j**.

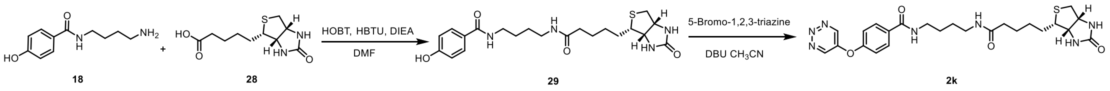

Compound 29

18 (250 mg, 1.2 mmol, 1.2 eq.) was added to a suspension of D-biotin (244 mg, 1 mmol), HBTU (455 mg, 1.2 mmol, 1.2 eq.), HOBT (202 mg, 1.5 mmol, 1.5 eq.) and DIEA (387 mg, 530 uL, 3 mmol, 3 eq.) in DMF (10 mL), and stirred at room temperature for 10 h. Then, the solvent was evaporated to dryness, followed by purification by semi prep RP-HPLC to give the white solid **29** (260 mg, 60.0% yield).

^{1}**H NMR** (700 MHz, Methanol-d_4) δ 7.59 (d, J = 8.7 Hz, 2H), 6.72 (d, J = 8.7 Hz, 2H), 4.36 (dd, J = 7.8, 5.0 Hz, 1H), 4.17 (dd, J = 7.9, 4.4 Hz, 1H), 3.27 (t, J = 6.9 Hz, 2H), 3.12 (td, J = 6.8, 1.7 Hz, 2H), 3.07 (ddd, J = 8.9, 5.8, 4.5 Hz, 1H), 2.79 (dd, J = 12.7, 5.0 Hz, 1H), 2.58 (d, J = 12.7 Hz, 1H), 2.09 (td, J = 7.3, 2.2 Hz, 2H), 1.60 (dddd, J = 29.0, 15.2, 13.6, 7.7 Hz, 2H), 1.50 (m, 6H), 1.32 (p, J = 7.7 Hz, 2H). ^{13}**C NMR** (176 MHz, Methanol-d_4) δ 176.06, 170.05, 166.12, 161.98, 130.20, 126.57, 116.04, 63.36, 61.61, 56.99, 41.02, 40.50, 40.05, 36.81, 29.76, 29.48, 28.04, 27.90, 26.90. **LRMS** (ESI+) m/z: 435.13 $[M + H]^+$, (ESI-) m/z: 433.49 $[M - H]^-$.

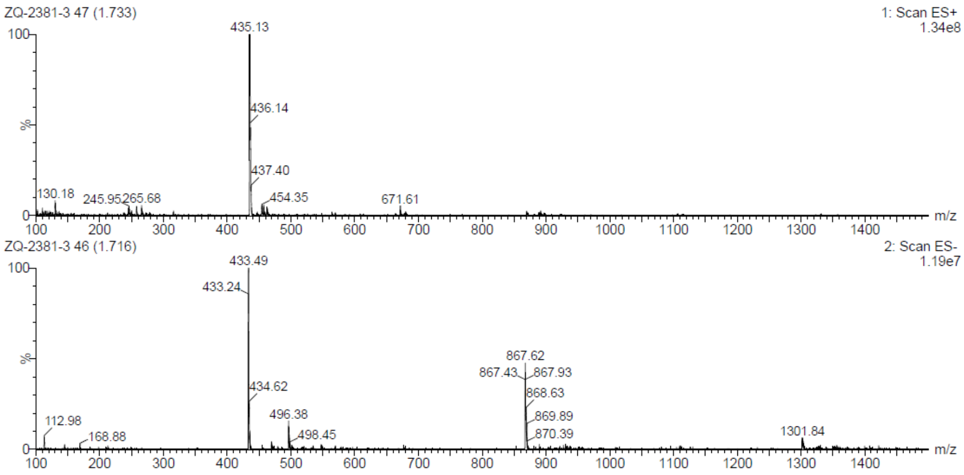

Figure S62. ESI-MS spectrum of **29**.

Compound 2k

29 (0.054 mmol, 23.4 mg) and 5-bromo-1,2,3-triazine (0.06 mmol,9.5 mg, 1.1 eq.) were added to a 10 mL flame-dried round-bottom flask equipped with a stir bar. 3 mL DMF was added to dissolve, then DBU (0.086 mmol, 13.2 mg, 1.5 eq.) was added. The reaction system was stirred at room temperature for 1.0 h. The solvent was removed in vacuo and the crude residue was directly purified through semi prep RP-HPLC to afford the desired product **2k** (22.1 mg, 79.8% yield).

^{1}**H NMR** (700 MHz, DMF-d_7) δ 9.15 (s, 2H), 8.62 (t, *J* = 5.6 Hz, 1H), 8.15 (m, 2H), 7.80 (t, *J* = 5.6 Hz, 1H), 7.52 (m, 2H), 6.39 (s, 1H), 6.31 (s, 1H), 4.46 (ddt, *J* = 7.5, 5.1, 1.1 Hz, 1H), 4.29 (ddd, *J* = 7.8, 4.4, 2.0 Hz, 1H), 3.41 (m, 2H), 3.20 (m, 3H), 2.94 (d, *J* = 5.1 Hz, 1H), 2.71 (d, *J* = 12.4 Hz, 1H), 2.17 (t, *J* = 7.4 Hz, 2H), 1.75 (ddt, *J* = 13.6, 10.2, 6.0 Hz, 1H), 1.61 (m, 7H), 1.42 (m, 2H).^{13}**C NMR** (176 MHz, DMF) δ 173.29, 166.39, 164.03, 155.74, 151.73, 141.58, 134.31, 131.03, 121.41, 62.62, 60.99, 56.94, 41.20, 40.50, 39.61, 36.60, 29.64, 29.47, 28.25, 28.01, 26.77. **LRMS** (ESI+) *m*/*z*: 514.08 [M + H]$^+$, (ESI-) *m*/*z*: 512.63 [M - H]$^-$.

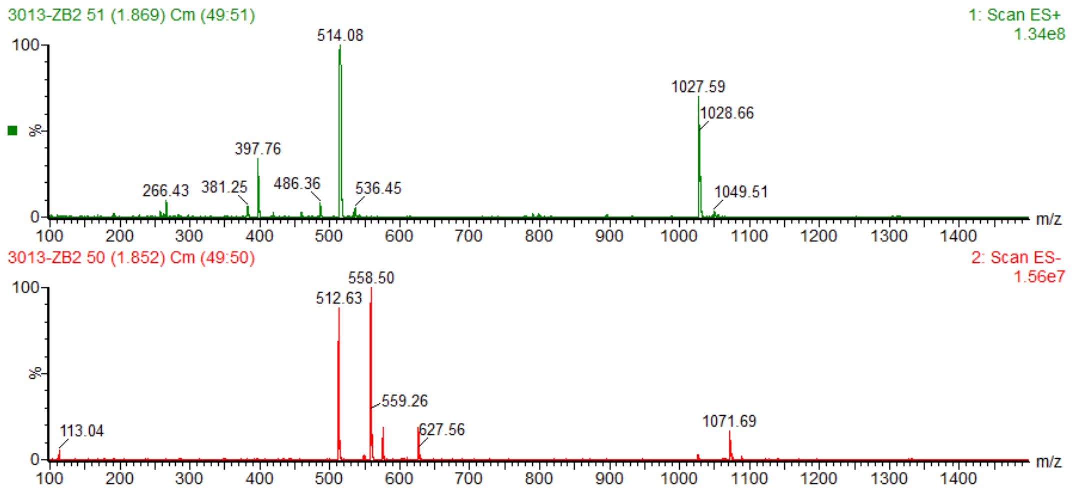

Figure S63. ESI-MS spectrum of **2k**.

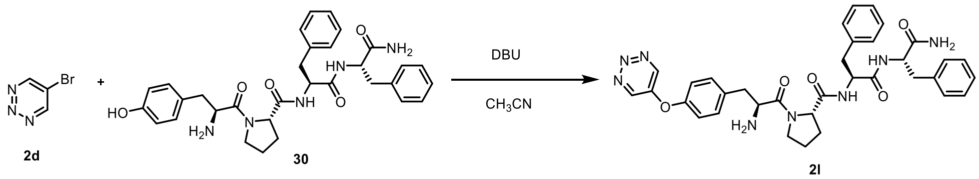

Compound 2l

H-Tyr-Pro-Phe-Phe-NH$_2$ (**30**, 0.087 mmol, 50 mg) and 5-bromo-1,2,3-triazine (0.096 mmol, 15.4 mg, 1.1 eq.) were added to a 10 mL flame-dried round-bottom flask equipped with a stir bar. 3 mL CH_3CN was added to dissolve, then DBU (0.174 mmol, 26.5 mg, 2 eq.) was added. The reaction system was stirred at room temperature for 1.0 h. The solvent was removed in vacuo and the crude residue was directly purified through semi prep RP-HPLC to afford the desired white product **2l** (30 mg, 53.1 % yield).

Analytical **HPLC** using Method D, RT = 11.219 min, the purity is 96.74%. **LRMS** (ESI+) *m*/*z*: 651.76 [M + H]$^+$, (ESI-) *m*/*z*: 649.87 [M - H]$^-$. **HRMS** (ES+) *m*/*z*: [M + H]$^+$ calcd for $C_{35}H_{38}N_8O_5^+$ 651.3043, found 651.3043.

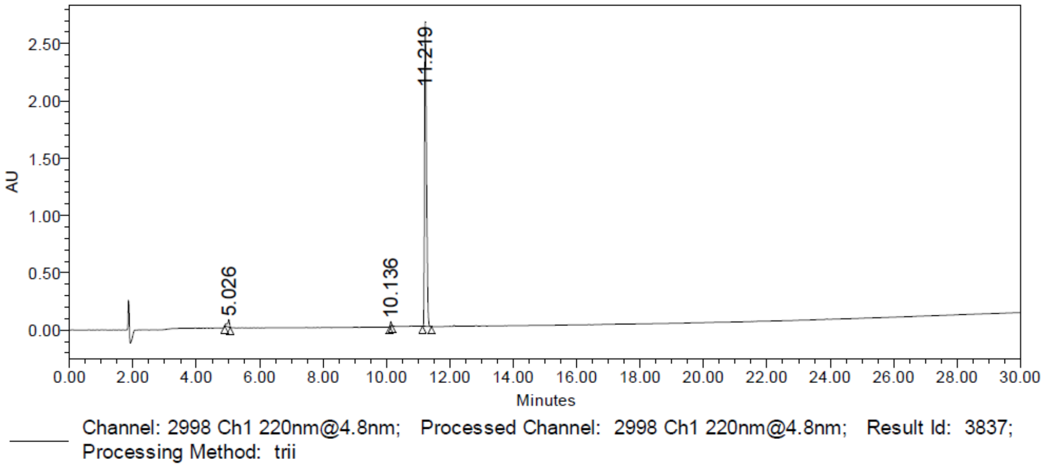

Processed Channel Descr.: 2998 Ch1 220nm@4.8nm

	Processed Channel Descr.	RT	Area	% Area	Height
1	2998 Ch1 220nm@4.8nm	5.026	301477	2.52	64502
2	2998 Ch1 220nm@4.8nm	10.136	88928	0.74	36917
3	2998 Ch1 220nm@4.8nm	11.219	11576406	96.74	2659107

Figure S64. HPLC-UV chromatogram at 220 nm of **2l**.

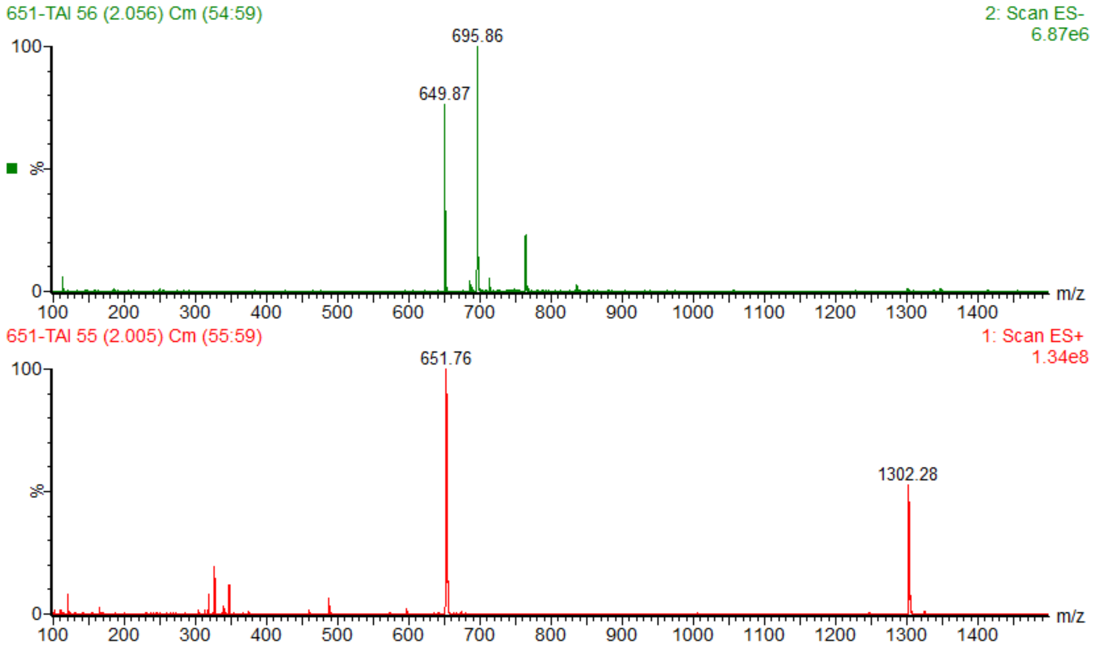

Figure S65. ESI-MS spectrum of **2l**.

Analysis Report

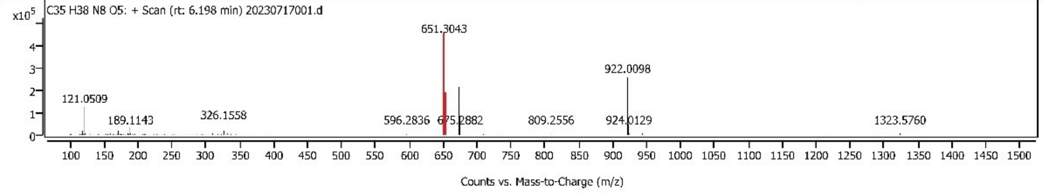

Spectrum Peaks

m/z	z	Abund	Abund %	m/z (Calc)	Diff (ppm)	Ion Species	Formula	Ion Type
651.3043	1	453470	100.00	651.3038	0.77	(M+H)+	C35 H38 N8 O5	
652.3062	1	187970	41.45	652.3067	-0.81	(M+H)+	C35 H38 N8 O5	
653.3072	1	38687	8.53	653.3095	-3.57	(M+H)+	C35 H38 N8 O5	
654.3096	1	7071	1.56	654.3121	-3.85	(M+H)+	C35 H38 N8 O5	
114.0913		5881	1.30					
116.9856		5088	1.12					
118.0865		21028	4.64					
119.0837		5921	1.31					
121.0509	1	124101	27.37					
122.0564	1	8499	1.87					
141.1039		5241	1.16					
151.1061		4983	1.10					
156.1377		21008	4.63					
157.1253		6343	1.40					
158.9651		8747	1.93					
163.0443		5387	1.19					
169.1201		6698	1.48					
171.1453		21141	4.66					
178.1240		4559	1.01					
183.0961		5305	1.17					
185.1508		9084	2.00					
187.1260		34549	7.62					
189.1143		8259	1.82					
195.0951		4887	1.08					
207.1529		5842	1.29					
211.1052		5130	1.13					
224.1777		4885	1.08					
237.1470		6263	1.38					
251.1632		4910	1.08					
277.1299		9938	2.19					
299.1147		7598	1.68					
309.2261	1	48493	10.69					
310.2294	1	8700	1.92					
326.1558	2	52102	11.49					
326.2458		13494	2.98					
326.6574	2	19960	4.40					
327.1664	2	6491	1.43					
331.2083	1	43184	9.52					
332.2110	1	8254	1.82					
337.1502		5532	1.22					
345.1810		5338	1.18					
596.2836		4694	1.04					
673.2853	1	215860	47.60					
674.2873	1	85116	18.77					
675.2882	1	18028	3.98					
689.2573		4937	1.09					
709.3534		5592	1.23					
809.2556		8195	1.81					
922.0098	1	256986	56.67					
923.0107	1	49991	11.02					
924.0129	1	8479	1.87					
943.9860		8565	1.89					
1323.5760	1	8739	1.93					
1324.5785	1	7278	1.60					

Spectrum Identification Table

Best	ID Source	Name	Formula	Species	m/z	Diff (ppm)	CAS	Score	Score (Lib)	Score (DB)	Score (MFG)	Lib/DB
Yes	MFG		C35 H38 N8 O5	(M+H)+	651.3043	0.04		97.75			97.75	
No	MFG		C36 H44 N O10	(M+H)+	651.3043	-0.18		96.00			96.00	
No	MFG		C37 H40 N5 O6	(M+H)+	651.3043	-2.13		94.22			94.22	
No	MFG		C34 H42 N4 O9	(M+H)+	651.3043	1.99		93.94			93.94	
No	MFG		C38 H36 N9 O2	(M+H)+	651.3043	-4.08		87.26			87.26	

Figure S66. Q-TOF-HRMS spectrum of **2l**.

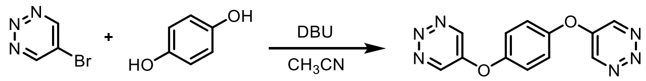

2d **31** **6a**

Compound 6a

Hydroquinone (**31**, 0.3 mmol, 33 mg) and 5-bromo-1,2,3-triazine (0.66 mmol, 159 mg, 2.2 eq.) were added to a 10 mL flame-dried round-bottom flask equipped with a stir bar. 5 mL acetonitrile was added to dissolve, then DBU (1 mmol, 152 mg, 3.3 eq.) was added. The reaction system was stirred at room temperature for 2.0 h. After triazine was completely consumed, monitored by LCMS analysis, the solvent was removed in vacuo. The crude residue was directly purified

through column chromatography on a silica gel with PE/EA (1:2) as the eluent to afford the desired product **6a** (38 mg, 47.3% yield).

^{1}H NMR (400 MHz, Acetone-d_6) δ 9.03 (s, 4H), 7.59 (s, 4H). **^{13}C NMR** (126 MHz, ACETONE-D_6) δ 152.02, 151.44, 140.64, 124.30. **LRMS** (ESI+) m/z: 269.33 [M + H]$^+$.

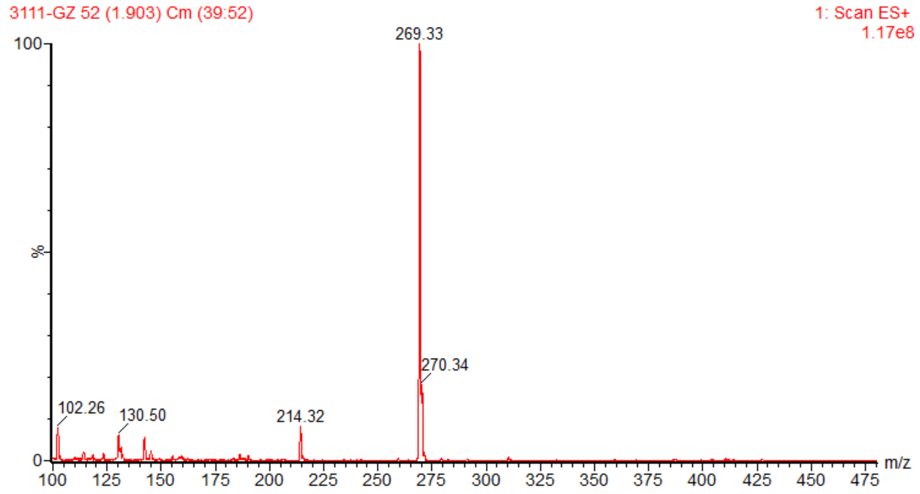

Figure S67. ESI-MS spectrum of **6a**.

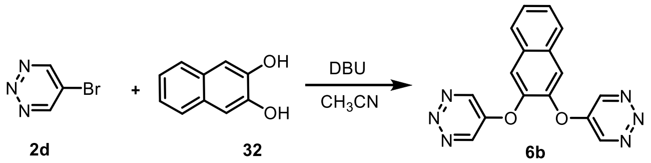

Compound 6b

2,3-Dihydroxynaphthalene (**32**, 0.3 mmol, 48 mg) and 5-bromo-1,2,3-triazine (0.66 mmol, 159 mg, 2.2 eq.) were added to a 10 mL flame-dried round-bottom flask equipped with a stir bar. 5 mL acetonitrile was added to dissolve, then DBU (1 mmol, 152 mg, 3.3 eq.) was added. The reaction system was stirred at room temperature for 2.0 h. After triazine was completely consumed, monitored by LCMS analysis, the solvent was removed in vacuo. The crude residue was directly purified through column chromatography on a silica gel with PE/EA (1:2) as the eluent to afford the white product **6b** (73 mg, 76.5% yield).

^{1}H NMR (700 MHz, Chloroform-d) δ 8.72 (s, 4H), 7.94 (dd, J = 6.2, 3.3 Hz, 2H), 7.87 (s, 2H), 7.69 (dd, J = 6.2, 3.3 Hz, 2H). **^{13}C NMR** (176 MHz, $CDCl_3$) δ 150.40, 141.46, 139.41, 132.47, 128.73, 128.14, 121.78. **LRMS** (ESI+) m/z: 319.43 [M + H]$^+$.

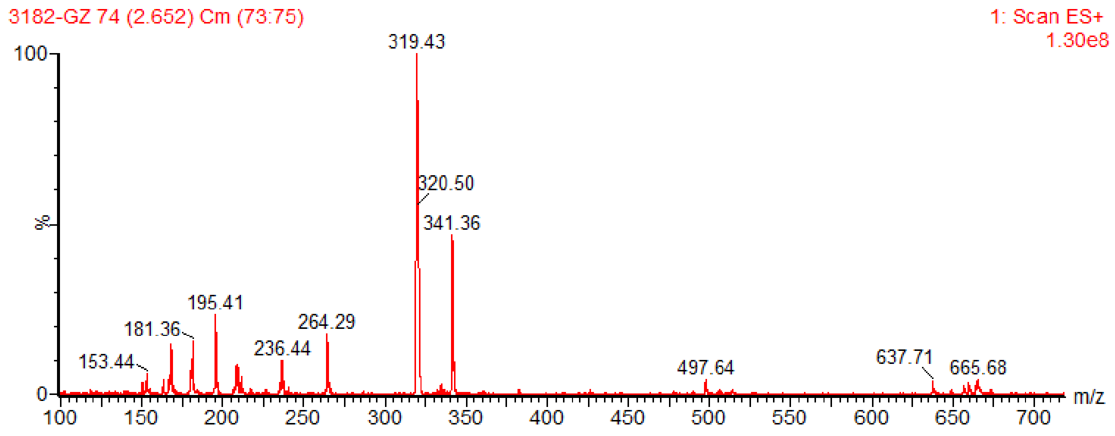

Figure S68. ESI-MS spectrum of **6b**.

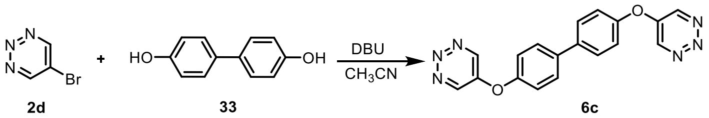

Compound 6c

4,4'-Biphenol (**33**, 0.3 mmol, 55.8 mg) and 5-bromo-1,2,3-triazine (0.66 mmol, 159 mg, 2.2 eq.) were added to a 10 mL flame-dried round-bottom flask equipped with a stir bar. 5 mL acetonitrile was added to dissolve, then DBU (1 mmol, 152 mg, 3.3 eq.) was added. The reaction system was stirred at room temperature for 2.0 h. After triazine was completely consumed, monitored by LCMS analysis, the solvent was removed in vacuo. The crude residue was directly purified through column chromatography on a silica gel with PE/EA (1:2) as the eluent to afford the white product **6c** (68 mg, 65.8% yield).

^{1}H NMR (700 MHz, Chloroform-*d*) δ 8.84 (s, 4H), 7.73 (d, *J* = 8.6 Hz, 4H), 7.28 (d, *J* = 8.5 Hz, 4H). **^{13}C NMR** (176 MHz, $CDCl_3$) δ 151.73, 150.89, 139.86, 138.84, 129.76, 121.18. **LRMS** (ESI+) *m*/*z*: 345.39 $[M + H]^+$.

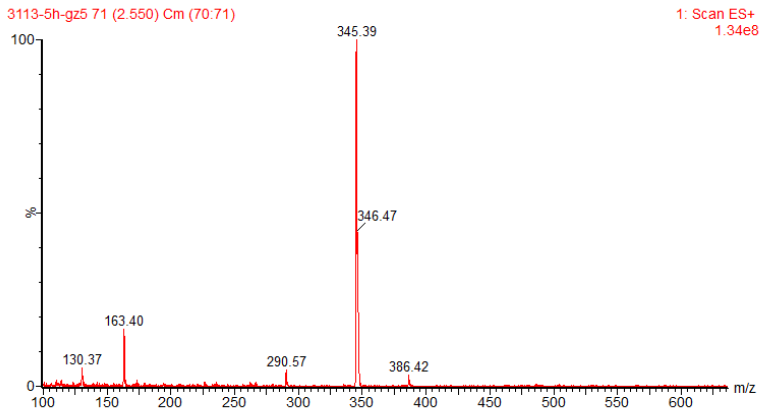

Figure S69. ESI-MS spectrum of **6c**.

5 Reaction optimization

5.1 Modify GSH with Tz-1 and reaction optimization

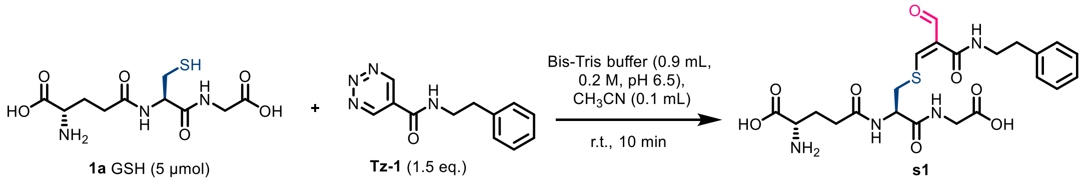

1a and **Tz-1** were used as substrate models. To a solution of GSH (**1a**, 5 µmol) in 200 mM Bis-Tris buffer (pH 6.5, 0.9 mL), **Tz-1** (7.5 µmol, 1.5 eq., dissolved in 0.1 mL CH_3CN) was added and the resulting mixture (final volume: 1 mL, [peptide] = 5 mM) was gently shaken for 10 min. Coumarin (5 µmol, dissolved in 0.1 mL CH_3CN) was added to the reaction mixture as an internal standard. The reaction mixture was analyzed by HPLC (monitored at 285 nm, using method **E**) to obtain corresponding yield (See section **5.2** for the calculation equation).

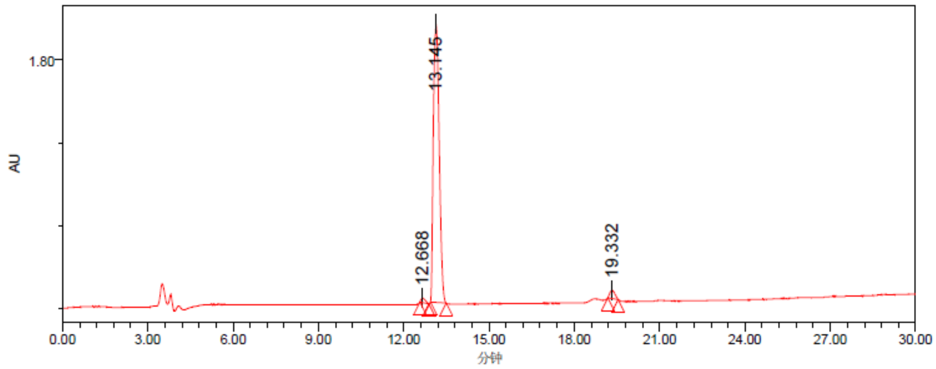

	RT	Area	% Area	Height
1	12.668	342747	1.14	32261
2	13.145	28875942	96.39	2018274
3	19.332	739246	2.47	60494

Figure S70. HPLC-UV chromatogram at 285 nm of **s1**.

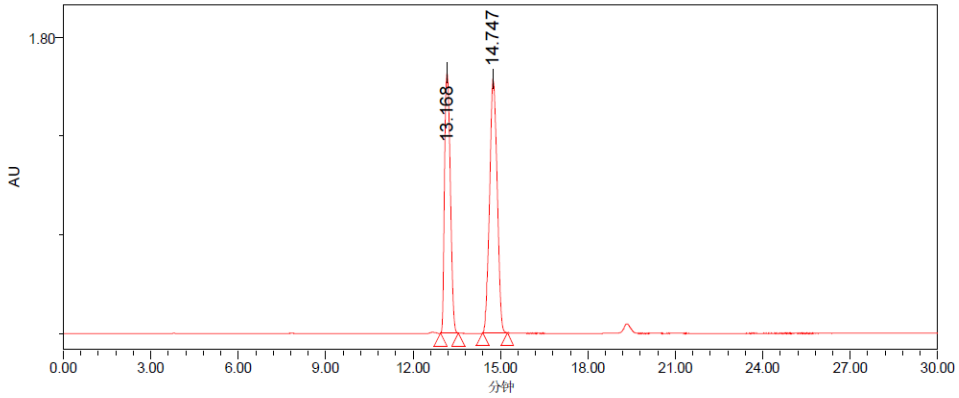

	RT	Area	% Area	Height
1	13.168	21934666	43.95	1582063
2	14.747	27974482	56.05	1542152

Figure S71. HPLC-UV chromatogram at 285 nm of **s1** and coumarin.

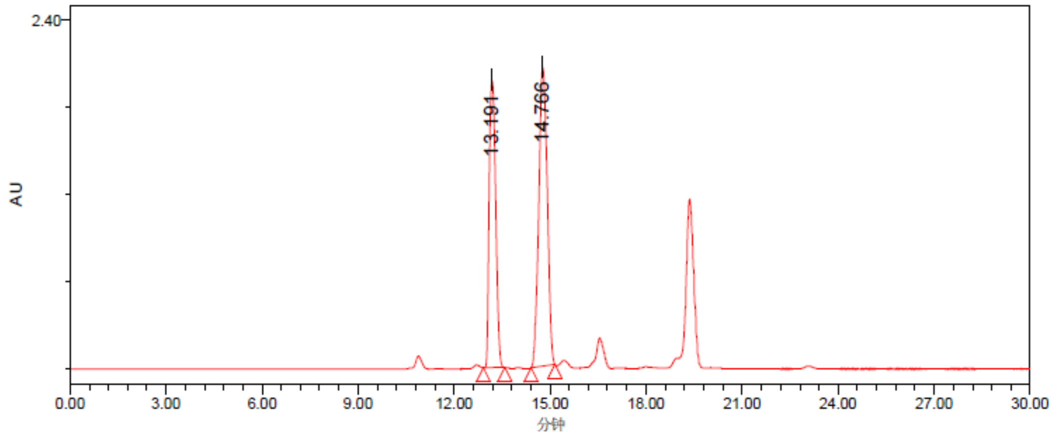

	RT	Area	% Area	Height
1	13.191	27967372	42.52	1974411
2	14.766	37808024	57.48	2053322

Figure S72. HPLC-UV chromatogram at 285 nm of entry 1.

5.2 Modify GSH with 2a and reaction optimization

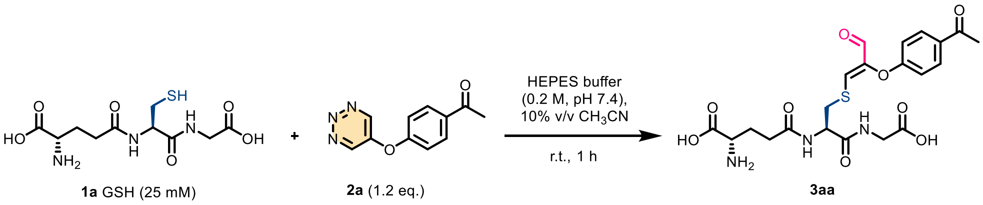

Glutathione (GSH, **1a**) and **2a** were used as substrate models. To a solution of GSH (**1a**, 7.675 mg, 0.025 mmol) in 200 mM HEPES buffer (pH 7.4, 0.9 mL), **2a** (6.45 mg, 0.03 mmol, 1.2 eq., dissolved in 0.1 mL CH_3CN) was added and the resulting mixture (final volume: 1 mL, [peptide] = 25 mM) was gently shaken for 1 h. Coumarin (3.65 mg, 0.025 mmol, dissolved in 0.1 mL CH_3CN) was added to the reaction mixture as an internal standard. The reaction mixture was analyzed by HPLC (monitored at 280 nm, method **D**).

The possible mechanism is shown in **Figure S73**[4].

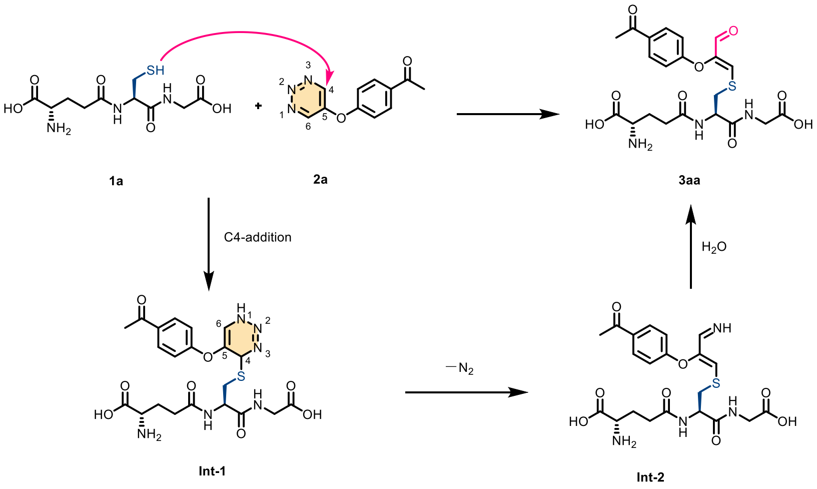

Figure S73. Plausible mechanism.

Coumarin acted as an internal standard containing absorption at 280 nm, a 1:1 mixture of **3aa** (1.25 mM) and coumarin (1.25 mM) was analyzed by HPLC (monitored at 280 nm, method **D**). The injection volumes were 1, 2, 5, 10, and 12 μL, respectively. Taking the HPLC peak area of **3aa** as the abscissa and the HPLC peak area of coumarin as the ordinate, a straight line was obtained with a slope of 0.5528 (R square = 0.9998), as shown in **Figure S34.**

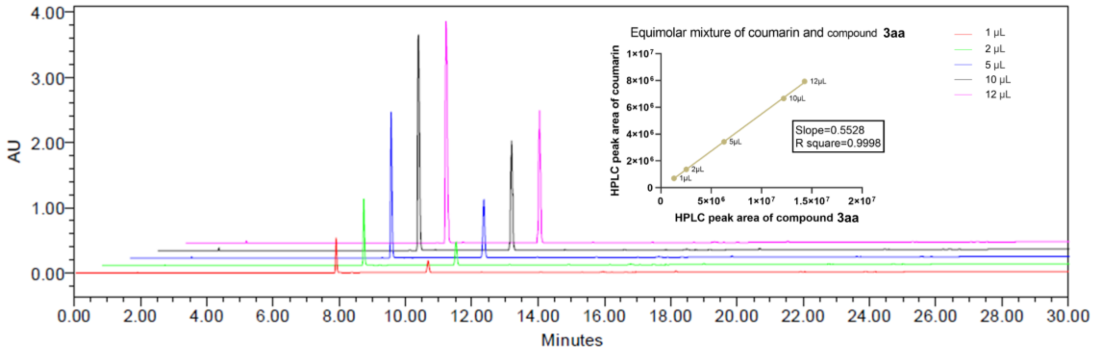

Figure S74. Overlay of HPLC-UV chromatogram at 280 nm.

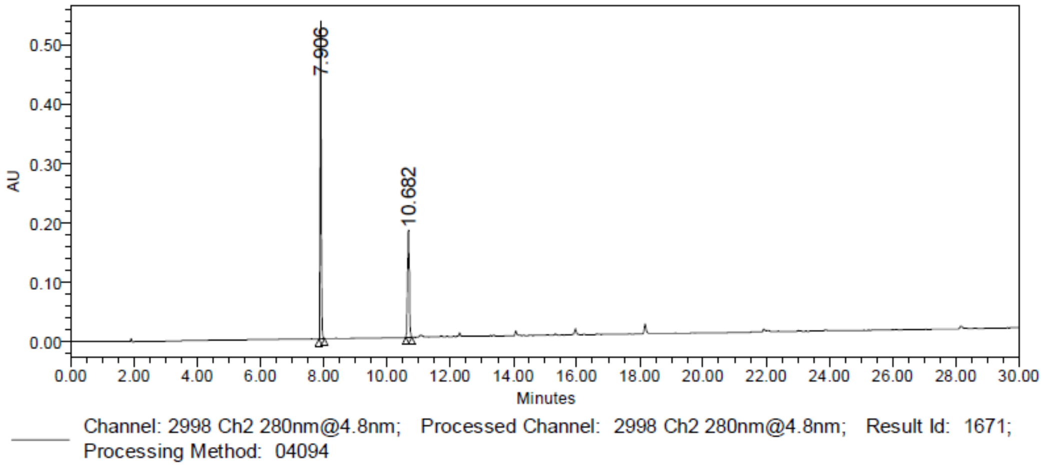

Processed Channel Descr.: 2998 Ch2 280nm@4.8nm

	Processed Channel Descr.	RT	Area	% Area	Height
1	2998 Ch2 280nm@4.8nm	7.906	1289145	64.63	536201
2	2998 Ch2 280nm@4.8nm	10.682	705633	35.37	181017

Figure S75. HPLC-UV chromatogram at 280 nm with an injection volume of 1 μL.

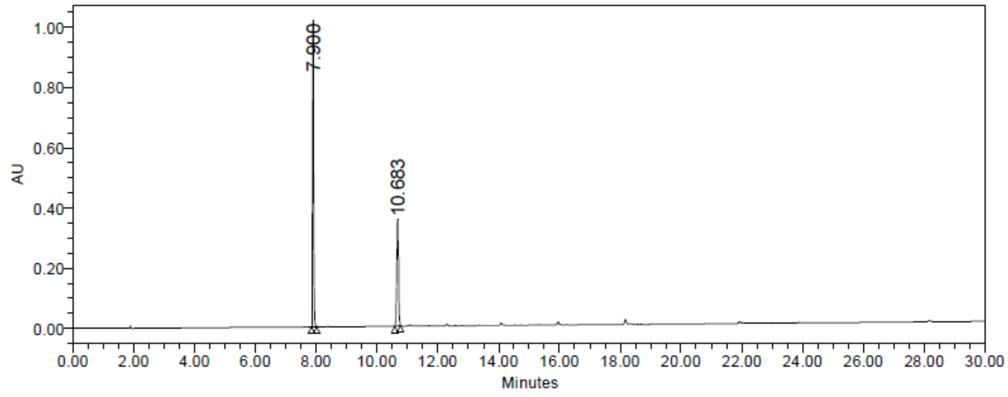

Channel: 2998 Ch2 280nm@4.8nm; Processed Channel: 2998 Ch2 280nm@4.8nm; Result Id: 1673; Processing Method: 04094

Processed Channel Descr.: 2998 Ch2 280nm@4.8nm

	Processed Channel Descr.	RT	Area	% Area	Height
1	2998 Ch2 280nm@4.8nm	7.900	2519643	64.70	1018326
2	2998 Ch2 280nm@4.8nm	10.683	1374829	35.30	353820

Figure S76. HPLC-UV chromatogram at 280 nm with an injection volume of 2 µL.

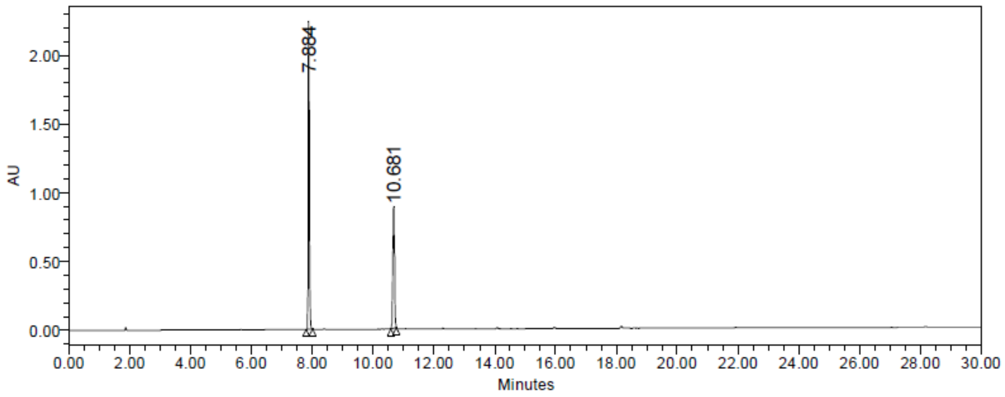

Channel: 2998 Ch2 280nm@4.8nm; Processed Channel: 2998 Ch2 280nm@4.8nm; Result Id: 1675; Processing Method: 04094

Processed Channel Descr.: 2998 Ch2 280nm@4.8nm

	Processed Channel Descr.	RT	Area	% Area	Height
1	2998 Ch2 280nm@4.8nm	7.884	6276830	64.65	2243098
2	2998 Ch2 280nm@4.8nm	10.681	3432719	35.35	887715

Figure S77. HPLC-UV chromatogram at 280 nm with an injection volume of 5 µL.

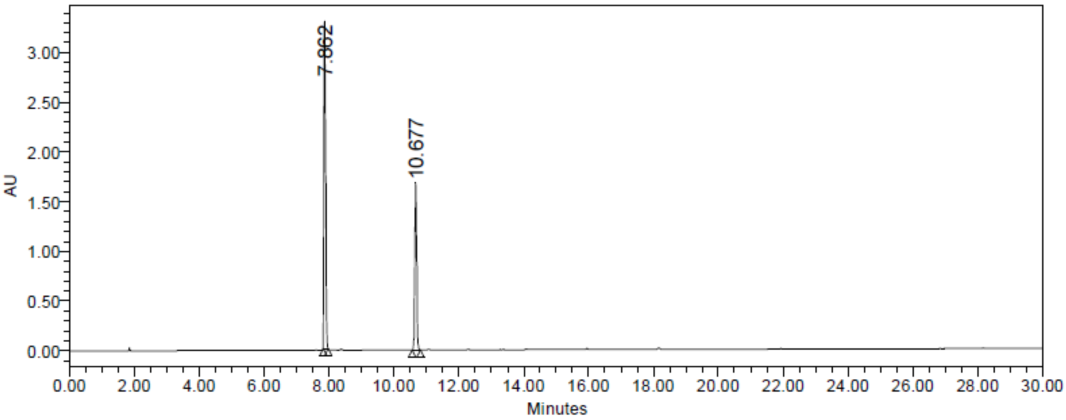

Channel: 2998 Ch2 280nm@4.8nm; Processed Channel: 2998 Ch2 280nm@4.8nm; Result Id: 1697; Processing Method: 04094

Processed Channel Descr.: 2998 Ch2 280nm@4.8nm

	Processed Channel Descr.	RT	Area	% Area	Height
1	2998 Ch2 280nm@4.8nm	7.862	12206626	64.70	3291405
2	2998 Ch2 280nm@4.8nm	10.677	6660514	35.30	1680256

Figure S78. HPLC-UV chromatogram at 280 nm with an injection volume of 10 µL.

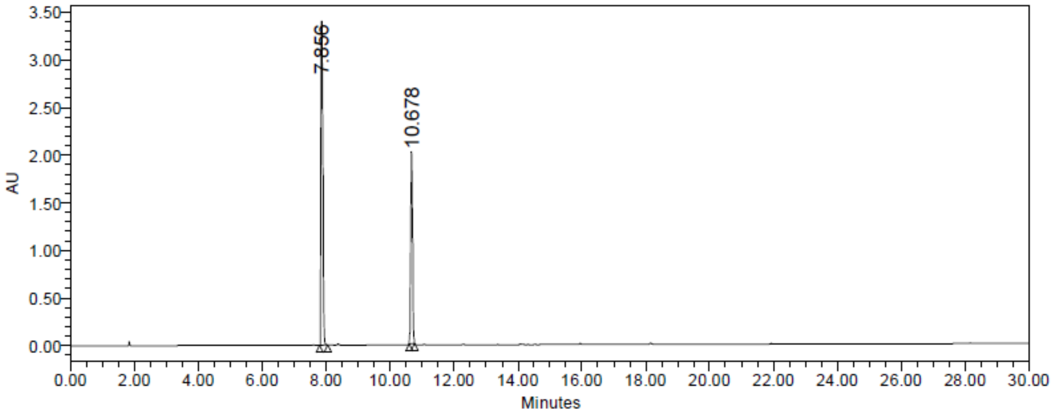

Channel: 2998 Ch2 280nm@4.8nm; Processed Channel: 2998 Ch2 280nm@4.8nm; Result Id: 1762; Processing Method: 04094

Processed Channel Descr.: 2998 Ch2 280nm@4.8nm

	Processed Channel Descr.	RT	Area	% Area	Height
1	2998 Ch2 280nm@4.8nm	7.856	14288664	64.29	3398969
2	2998 Ch2 280nm@4.8nm	10.678	7935221	35.71	2013891

Figure S79. HPLC-UV chromatogram at 280 nm with an injection volume of 12 µL.

Reaction under the optimized conditions as a representative example (Table 1, entry 1):

The yield was determined as 98% using HPLC peak area in comparison with the absorption of the internal standard by the equation below:

$$Yield(\%) = \frac{A1}{A2/k} \times 100$$

A1-A2 refer to the following HPLC peak areas (λ= 280 nm), respectively:

A1: **3aa** in the reaction mixture

A2: coumarin in the reaction mixture

k: slope = 0.5528

An example for yield calculation is shown here.

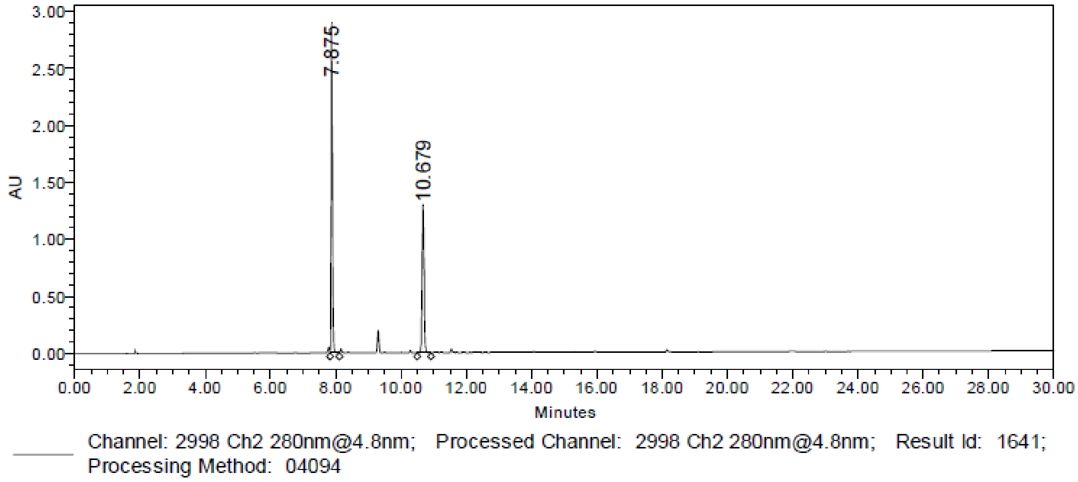

Processed Channel Descr.: 2998 Ch2 280nm@4.8nm

	Processed Channel Descr.	RT	Area	% Area	Height
1	2998 Ch2 280nm@4.8nm	7.875	9070844	63.94	2895828
2	2998 Ch2 280nm@4.8nm	10.679	5115775	36.06	1298833

Figure S80. HPLC-UV chromatogram at 280 nm of entry 1.

The yield was calculated as follows:

$$Yield(\%) = \frac{A1}{A2/k} \times 100 = \frac{9070844}{5115775/0.5528} \times 100 = 98\%$$

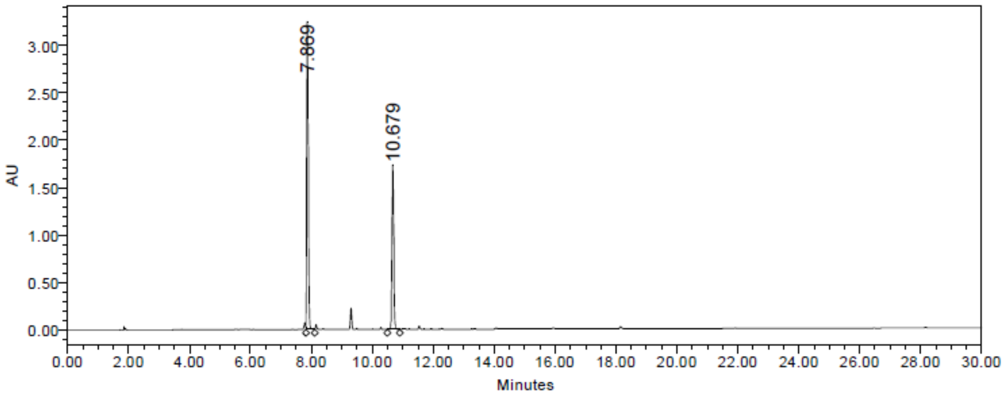

Channel: 2998 Ch2 280nm@4.8nm; Processed Channel: 2998 Ch2 280nm@4.8nm; Result Id: 1643; Processing Method: 04094

Processed Channel Descr.: 2998 Ch2 280nm@4.8nm

	Processed Channel Descr.	RT	Area	% Area	Height
1	2998 Ch2 280nm@4.8nm	7.869	11575235	62.86	3243716
2	2998 Ch2 280nm@4.8nm	10.679	6838983	37.14	1735721

Figure S81. HPLC-UV chromatogram at 280 nm of entry 2.

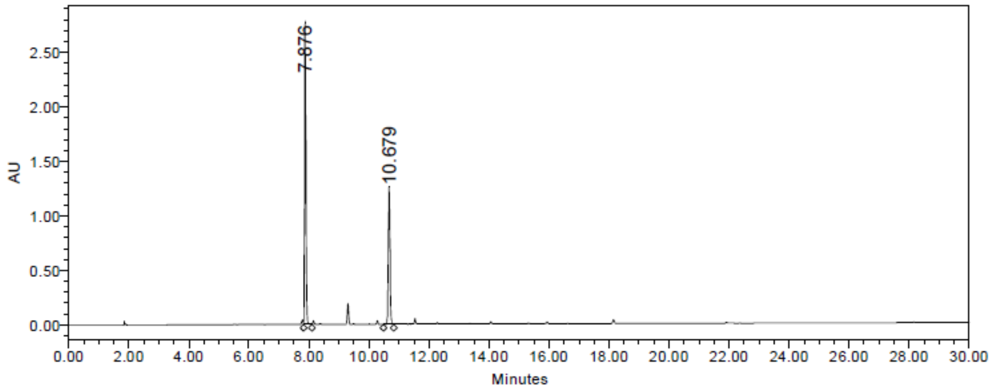

Channel: 2998 Ch2 280nm@4.8nm; Processed Channel: 2998 Ch2 280nm@4.8nm; Result Id: 1645; Processing Method: 04094

Processed Channel Descr.: 2998 Ch2 280nm@4.8nm

	Processed Channel Descr.	RT	Area	% Area	Height
1	2998 Ch2 280nm@4.8nm	7.876	8482525	63.12	2781715
2	2998 Ch2 280nm@4.8nm	10.679	4955778	36.88	1260067

Figure S82. HPLC-UV chromatogram at 280 nm of entry 3.

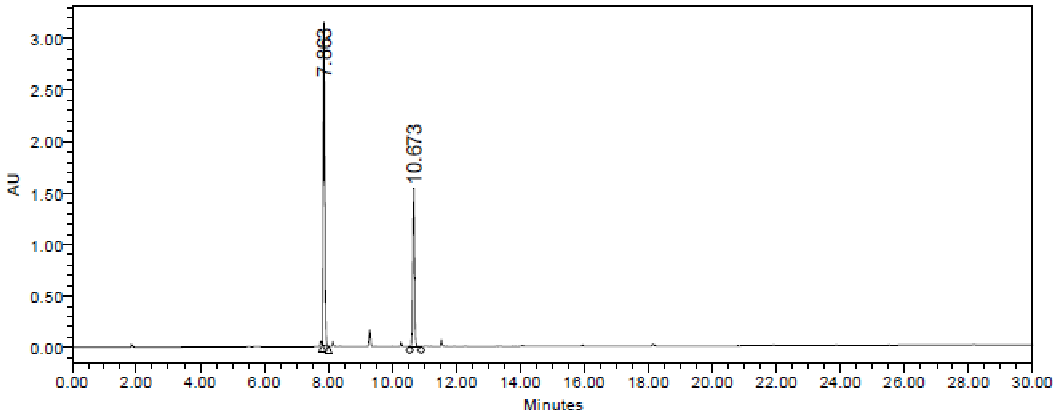

Channel: 2998 Ch2 280nm@4.8nm; Processed Channel: 2998 Ch2 280nm@4.8nm; Result Id: 1647; Processing Method: 04094

Processed Channel Descr.: 2998 Ch2 280nm@4.8nm

	Processed Channel Descr.	RT	Area	% Area	Height
1	2998 Ch2 280nm@4.8nm	7.863	10652104	63.68	3142717
2	2998 Ch2 280nm@4.8nm	10.673	6075848	36.32	1541204

Figure S83. HPLC-UV chromatogram at 280 nm of entry 4.

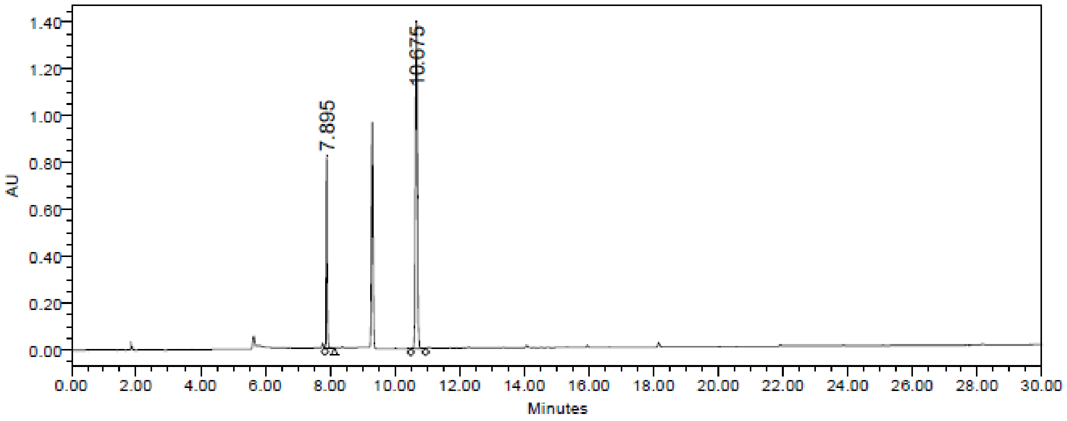

Channel: 2998 Ch2 280nm@4.8nm; Processed Channel: 2998 Ch2 280nm@4.8nm; Result Id: 1655; Processing Method: 04094

Processed Channel Descr.: 2998 Ch2 280nm@4.8nm

	Processed Channel Descr.	RT	Area	% Area	Height
1	2998 Ch2 280nm@4.8nm	7.895	2072708	27.41	825378
2	2998 Ch2 280nm@4.8nm	10.675	5487817	72.59	1396037

Figure S84. HPLC-UV chromatogram at 280 nm of entry 5.

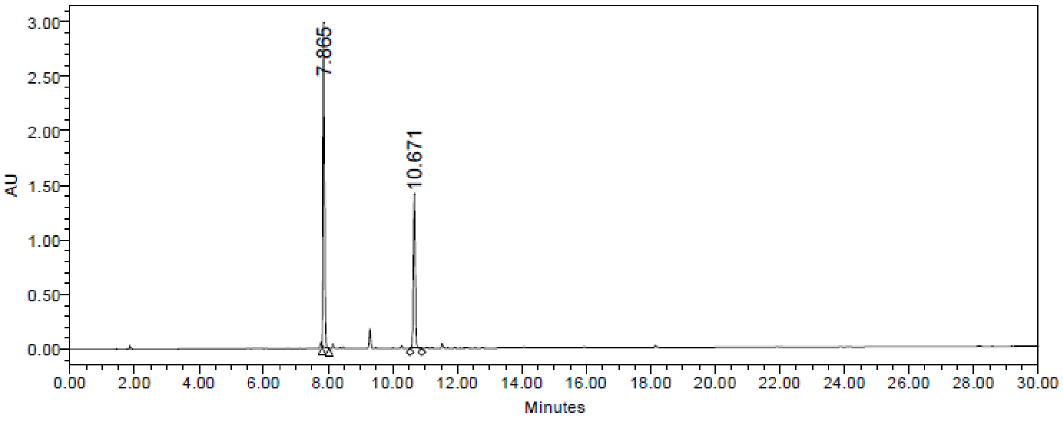

Channel: 2998 Ch2 280nm@4.8nm; Processed Channel: 2998 Ch2 280nm@4.8nm; Result Id: 1657; Processing Method: 04093

Processed Channel Descr.: 2998 Ch2 280nm@4.8nm

	Processed Channel Descr.	RT	Area	% Area	Height
1	2998 Ch2 280nm@4.8nm	7.865	9596960	63.21	2979208
2	2998 Ch2 280nm@4.8nm	10.671	5585635	36.79	1417782

Figure S85. HPLC-UV chromatogram at 280 nm of entry 6.

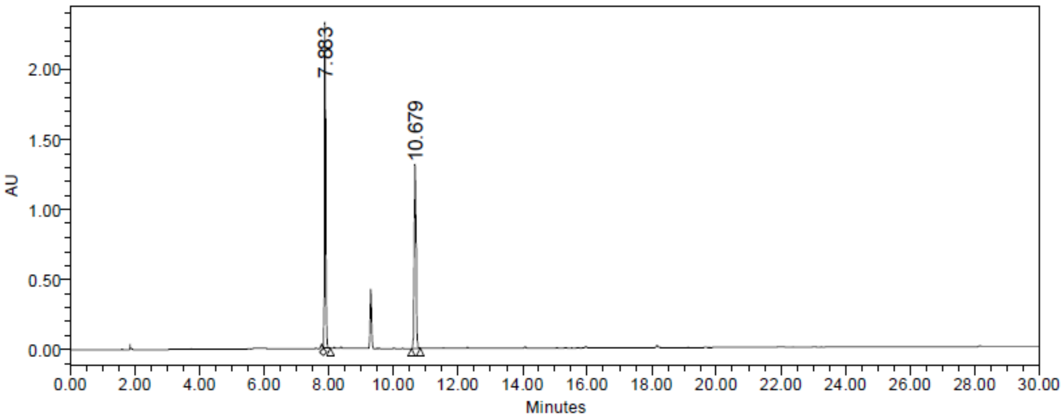

Channel: 2998 Ch2 280nm@4.8nm; Processed Channel: 2998 Ch2 280nm@4.8nm; Result Id: 1736; Processing Method: 04094

Processed Channel Descr.: 2998 Ch2 280nm@4.8nm

	Processed Channel Descr.	RT	Area	% Area	Height
1	2998 Ch2 280nm@4.8nm	7.883	6643641	56.15	2330566
2	2998 Ch2 280nm@4.8nm	10.679	5187282	43.85	1315365

Figure S86. HPLC-UV chromatogram at 280 nm of entry 7.

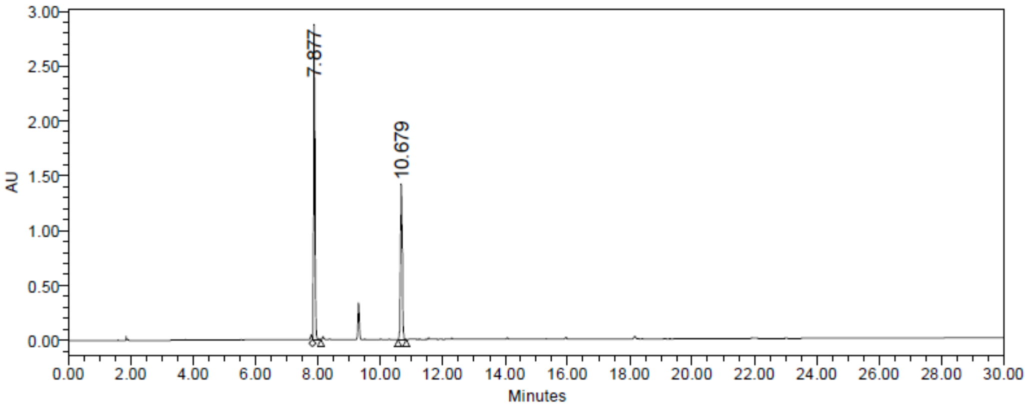

Channel: 2998 Ch2 280nm@4.8nm; Processed Channel: 2998 Ch2 280nm@4.8nm; Result Id: 1724; Processing Method: 04094

Processed Channel Descr.: 2998 Ch2 280nm@4.8nm

	Processed Channel Descr.	RT	Area	% Area	Height
1	2998 Ch2 280nm@4.8nm	7.877	8945646	61.47	2871504
2	2998 Ch2 280nm@4.8nm	10.679	5607782	38.53	1420688

Figure S87. HPLC-UV chromatogram at 280 nm of entry 8.

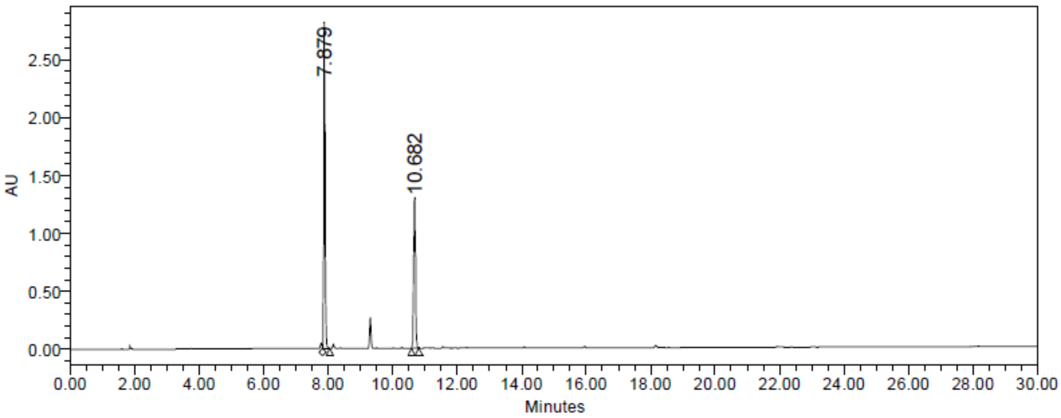

Channel: 2998 Ch2 280nm@4.8nm; Processed Channel: 2998 Ch2 280nm@4.8nm; Result Id: 1758; Processing Method: 04094

Processed Channel Descr.: 2998 Ch2 280nm@4.8nm

	Processed Channel Descr.	RT	Area	% Area	Height
1	2998 Ch2 280nm@4.8nm	7.879	8645697	62.91	2818161
2	2998 Ch2 280nm@4.8nm	10.682	5097119	37.09	1300452

Figure S88. HPLC-UV chromatogram at 280 nm of entry 9.

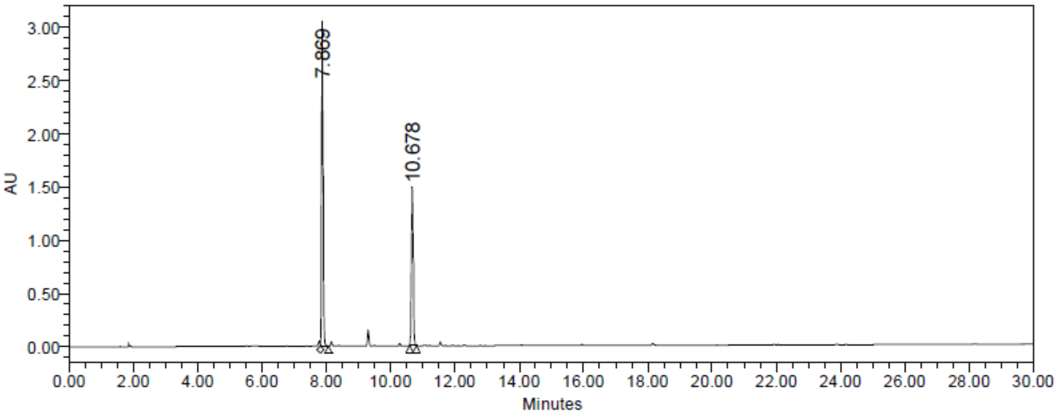

Channel: 2998 Ch2 280nm@4.8nm; Processed Channel: 2998 Ch2 280nm@4.8nm; Result Id: 1760; Processing Method: 04094

Processed Channel Descr.: 2998 Ch2 280nm@4.8nm

	Processed Channel Descr.	RT	Area	% Area	Height
1	2998 Ch2 280nm@4.8nm	7.869	9988399	63.03	3053179
2	2998 Ch2 280nm@4.8nm	10.678	5857886	36.97	1492869

Figure S89. HPLC-UV chromatogram at 280 nm of entry 10.

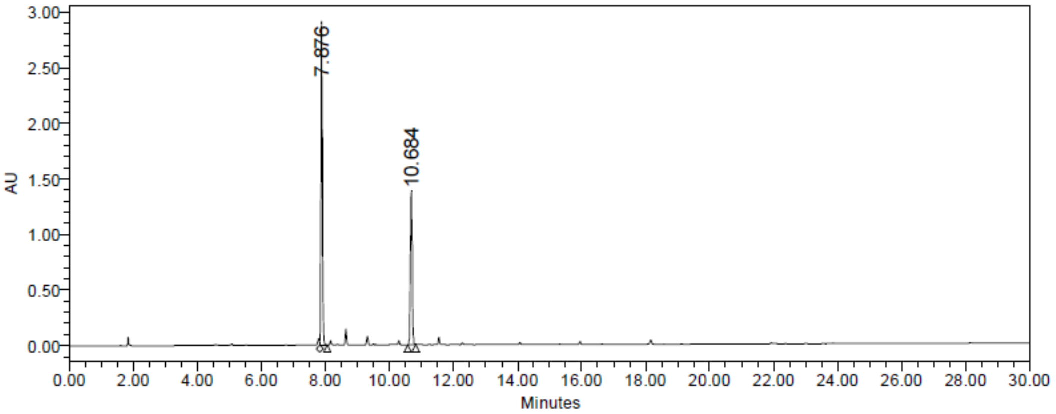

Channel: 2998 Ch2 280nm@4.8nm; Processed Channel: 2998 Ch2 280nm@4.8nm; Result Id: 1784; Processing Method: 04094

Processed Channel Descr.: 2998 Ch2 280nm@4.8nm

	Processed Channel Descr.	RT	Area	% Area	Height
1	2998 Ch2 280nm@4.8nm	7.876	9506662	63.50	2909053
2	2998 Ch2 280nm@4.8nm	10.684	5464641	36.50	1379280

Figure S90. HPLC-UV chromatogram at 280 nm of entry 11.

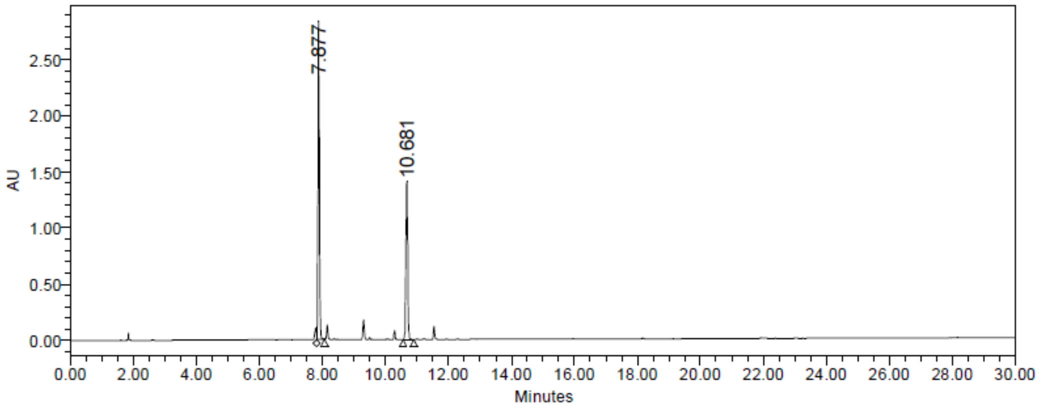

Channel: 2998 Ch2 280nm@4.8nm; Processed Channel: 2998 Ch2 280nm@4.8nm; Result Id: 1789; Processing Method: 04094

Processed Channel Descr.: 2998 Ch2 280nm@4.8nm

	Processed Channel Descr.	RT	Area	% Area	Height
1	2998 Ch2 280nm@4.8nm	7.877	9173700	62.13	2837329
2	2998 Ch2 280nm@4.8nm	10.681	5590816	37.87	1407069

Figure S91. HPLC-UV chromatogram at 280 nm of entry 12.

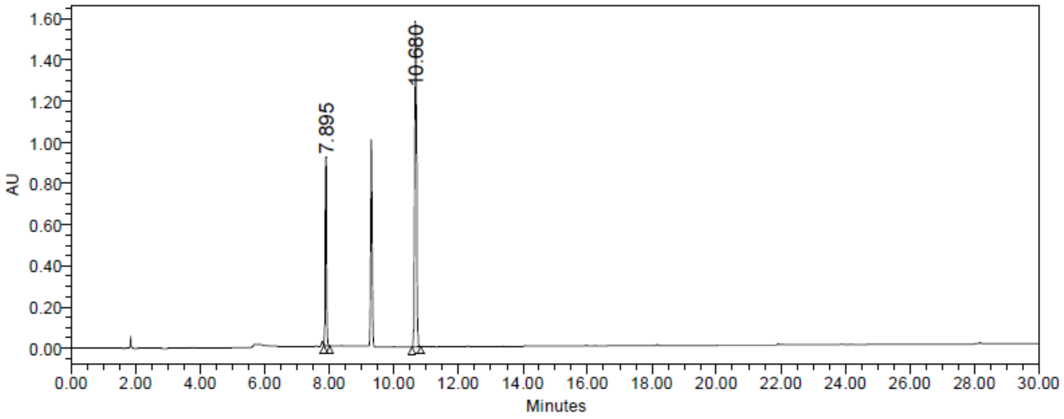

Channel: 2998 Ch2 280nm@4.8nm; Processed Channel: 2998 Ch2 280nm@4.8nm; Result Id: 1810; Processing Method: 04094

Processed Channel Descr.: 2998 Ch2 280nm@4.8nm

	Processed Channel Descr.	RT	Area	% Area	Height
1	2998 Ch2 280nm@4.8nm	7.895	2700264	30.07	918432
2	2998 Ch2 280nm@4.8nm	10.680	6281042	69.93	1579845

Figure S92. HPLC-UV chromatogram at 280 nm of entry 13.

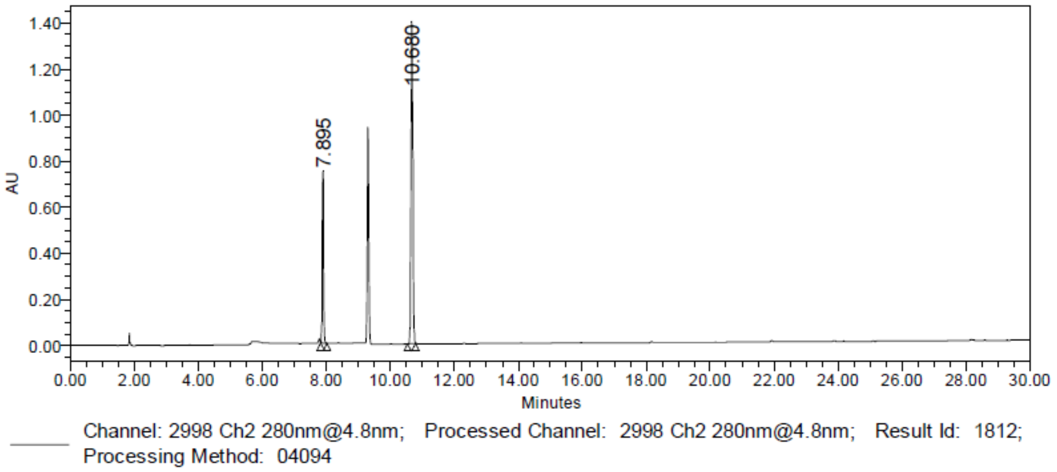

Processed Channel Descr.: 2998 Ch2 280nm@4.8nm

	Processed Channel Descr.	RT	Area	% Area	Height
1	2998 Ch2 280nm@4.8nm	7.895	2202528	28.44	745132
2	2998 Ch2 280nm@4.8nm	10.680	5541856	71.56	1398234

Figure S93. HPLC-UV chromatogram at 280 nm of entry 14.

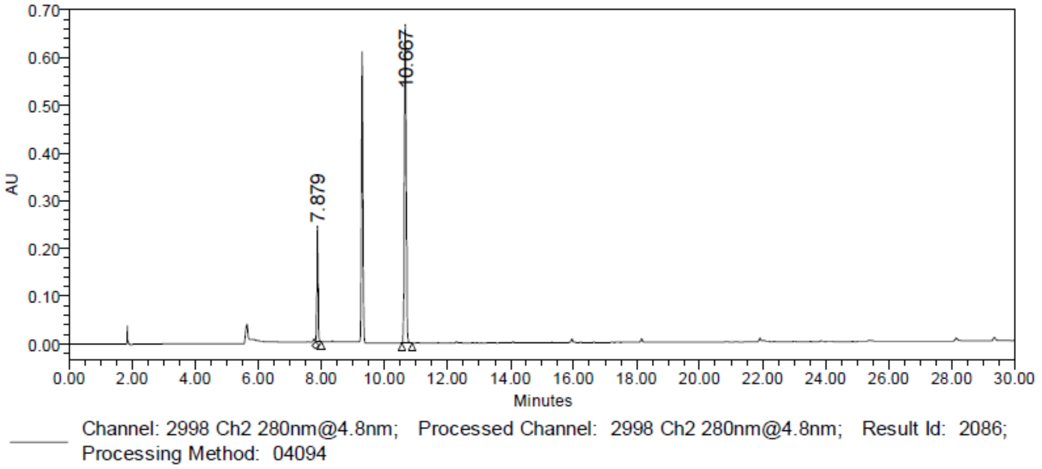

Processed Channel Descr.: 2998 Ch2 280nm@4.8nm

	Processed Channel Descr.	RT	Area	% Area	Height
1	2998 Ch2 280nm@4.8nm	7.879	653639	19.90	242649
2	2998 Ch2 280nm@4.8nm	10.667	2631510	80.10	666919

Figure S94. HPLC-UV chromatogram at 280 nm of entry 15.

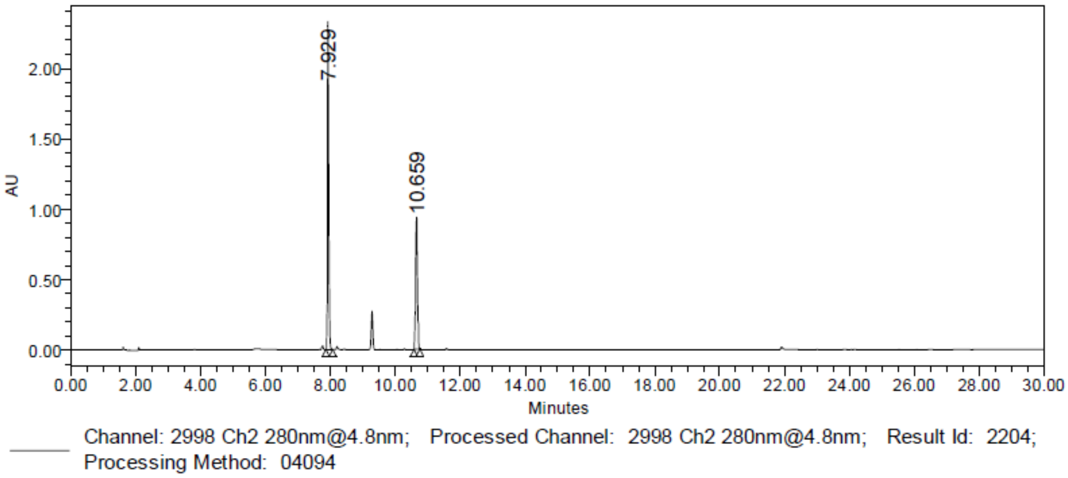

Processed Channel Descr.: 2998 Ch2 280nm@4.8nm

	Processed Channel Descr.	RT	Area	% Area	Height
1	2998 Ch2 280nm@4.8nm	7.929	6635439	64.04	2328251
2	2998 Ch2 280nm@4.8nm	10.659	3725873	35.96	935700

Figure 95. HPLC-UV chromatogram at 280 nm of entry 16.

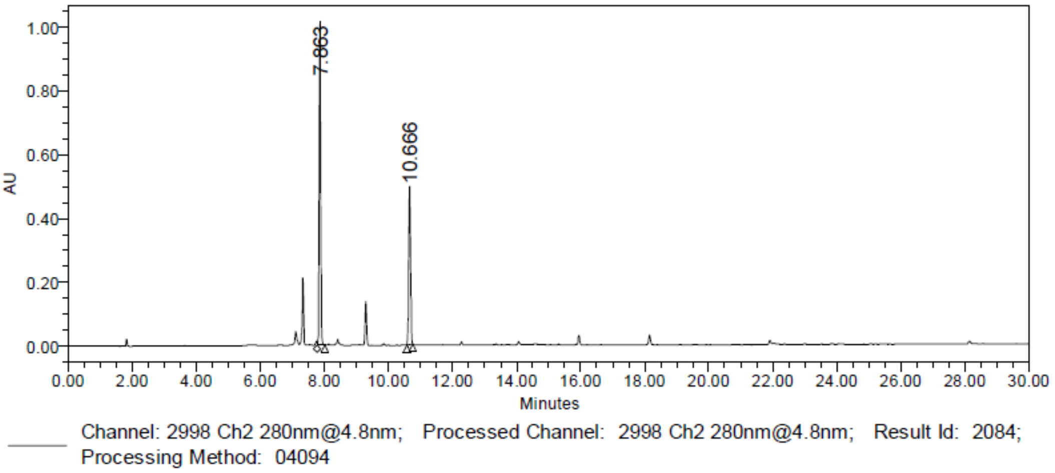

Processed Channel Descr.: 2998 Ch2 280nm@4.8nm

	Processed Channel Descr.	RT	Area	% Area	Height
1	2998 Ch2 280nm@4.8nm	7.863	3177331	61.99	1014665
2	2998 Ch2 280nm@4.8nm	10.666	1948340	38.01	496375

Figure S96. HPLC-UV chromatogram at 280 nm of entry 17.

6 Peptide scope of Cys-selective modification

General procedure A:

The dried peptide (1 equiv.) was dissolved in appropriate amount of HEPES buffer (0.2 M, pH = 7.4) to reach a concentration of 27.78 mM. To this solution, 1,2,3-triazines (1.2 equiv.) dissolved in acetonitrile (1/9 of the volume of buffer) was added. The final concentrations in the reaction were 25 mM peptide, 30 mM 1,2,3-triazines and 10% acetonitrile. The reaction incubated for 1 h at room temperature and the reaction process was detected by LCMS. The solvent was purification by preparative RP-HPLC and to give the product by freeze drying.

Here, **1a** reacted with **2a** to obtain **3aa** as a representative example to illustrate the general procedure A.

1a (0.1 mmol, 30.8 mg, 1 equiv.) was dissolved in HEPES buffer (0.2 M, pH = 7.4, 3.6 mL) to reach a concentration of 27.78 mM. To this solution **2a** (0.12 mmol, 25.8 mg, 1.2 equiv.) dissolved in acetonitrile (0.4 mL) was added. The final concentrations in the reaction were 25 mM **1a**, 30 mM **2a** and 10% acetonitrile. The reaction incubated for 1 h at room temperature and the reaction process was detected by LCMS. The solvent was purification by preparative RP-HPLC and to give the white solid **3aa** (39.9 mg, 80.6% yield) by freeze drying.

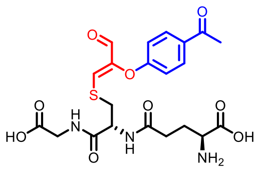

3aa

According to the general procedure A, **3aa** was obtained as a white solid in 80.6% isolated yield.

^{1}H NMR (700 MHz, Deuterium Oxide) δ 9.14 (s, 1H), 7.93 (s, 1H), 7.86 (d, *J* = 8.3 Hz, 2H), 6.92 (d, *J* = 8.3 Hz, 2H), 4.65 (dd, *J* = 8.4, 5.3 Hz, 1H), 3.82 (s, 2H), 3.66 (m, 1H), 3.43 (dd, *J* = 14.3, 5.3 Hz, 1H), 3.21 (dd, *J* = 14.8, 8.4 Hz, 1H), 2.50 (s, 3H), 2.38 (t, *J* = 7.7 Hz, 2H), 2.00 (m, 2H). **^{13}C NMR** (176 MHz, Deuterium Oxide) δ 202.24, 185.83, 174.56, 173.94, 173.54, 171.24, 159.42, 149.10, 145.79, 131.42, 131.20, 114.96, 53.80, 53.34, 41.91, 35.30, 31.25, 26.00, 25.99. **LRMS** (ESI+) *m*/*z*: 496.31 $[M + H]^{+}$, (ESI-) *m*/*z*: 494.49 $[M - H]^{-}$. Analytical **HPLC** using Method D, RT = 7.87 min, the HPLC purity is 98.65%.

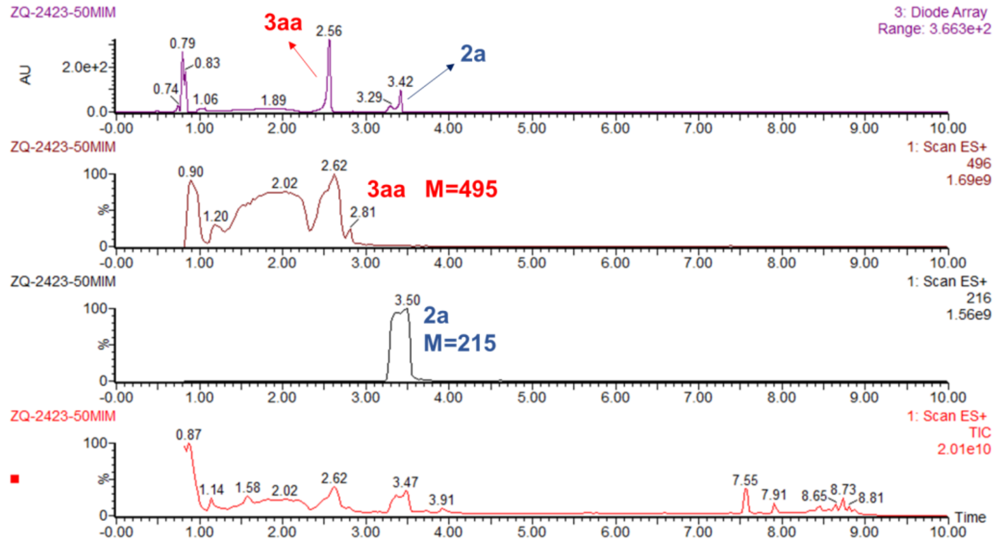

Figure S97. UPLC-MS chromatogram of reaction mixture including TIC and UV curve.

(Extract **2a** and **3aa** mass chromatograms from full scan data).

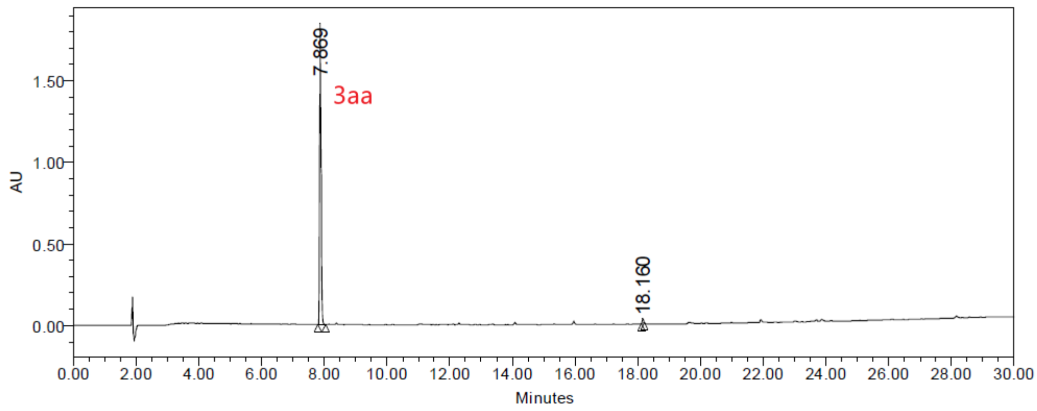

Channel: 2998 Ch1 220nm@4.8nm; Processed Channel: 2998 Ch1 220nm@4.8nm; Result Id: 1627; Processing Method: 010906

Processed Channel Descr.: 2998 Ch1 220nm@4.8nm

	Processed Channel Descr.	RT	Area	% Area	Height
1	2998 Ch1 220nm@4.8nm	7.869	5957534	98.65	1846661
2	2998 Ch1 220nm@4.8nm	18.160	81253	1.35	26635

Figure S98. HPLC-UV chromatogram at 220 nm of **3aa**.

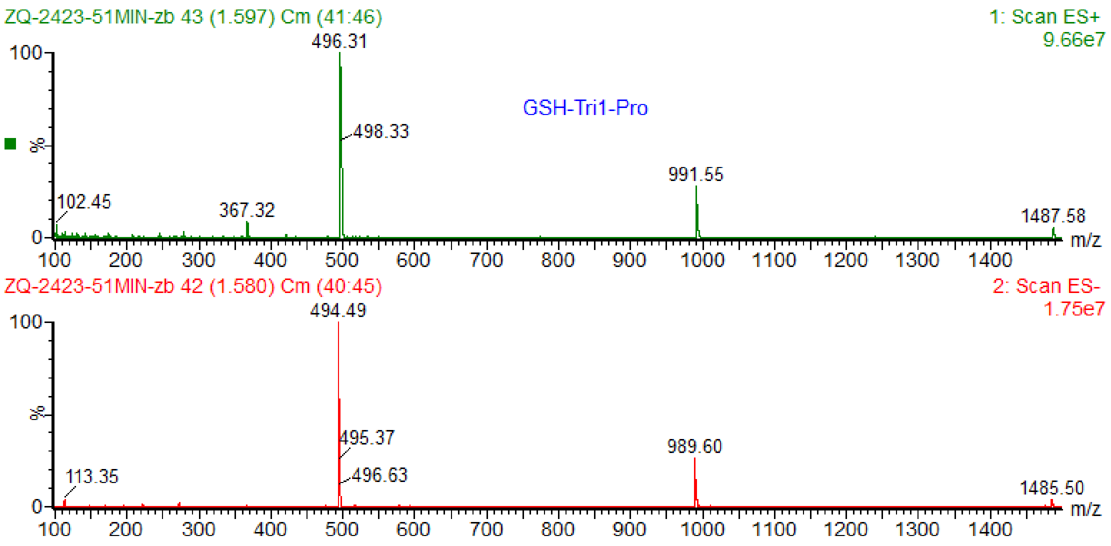

Figure S99. ESI-MS spectrum of **3aa**.

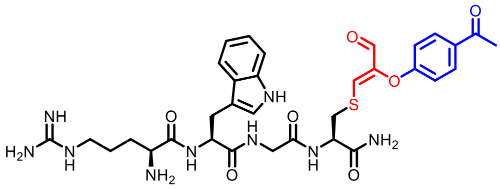

3ba

According to the general procedure A, **1b** (25.6 mg, 0.05 mmol) reacted with **2a** (12.7 mg, 0.06 mmol) to afford the white solid **3ba** (18.2 mg) in 51.5% isolated yield.

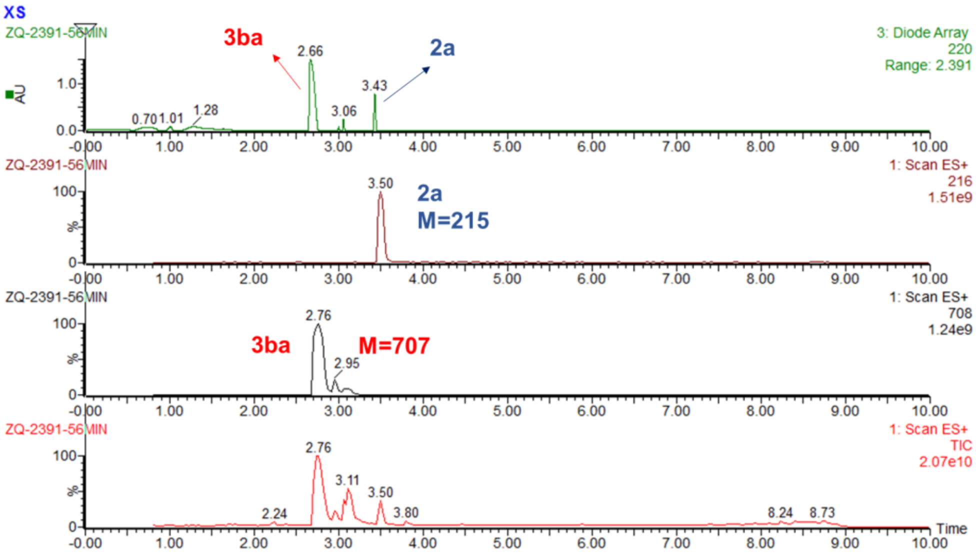

Figure S100. UPLC-MS chromatogram of reaction mixture including TIC and UV curve.

(Extract **2a** and **3ba** mass chromatograms from full scan data).

Analytical **HPLC** using Method D, RT = 9.785 min, the HPLC purity is 98.51%. **LRMS** (ESI+) *m*/*z*: 708.45 [M + H]$^+$, (ESI-) *m*/*z*: 706.70 [M - H]$^-$.

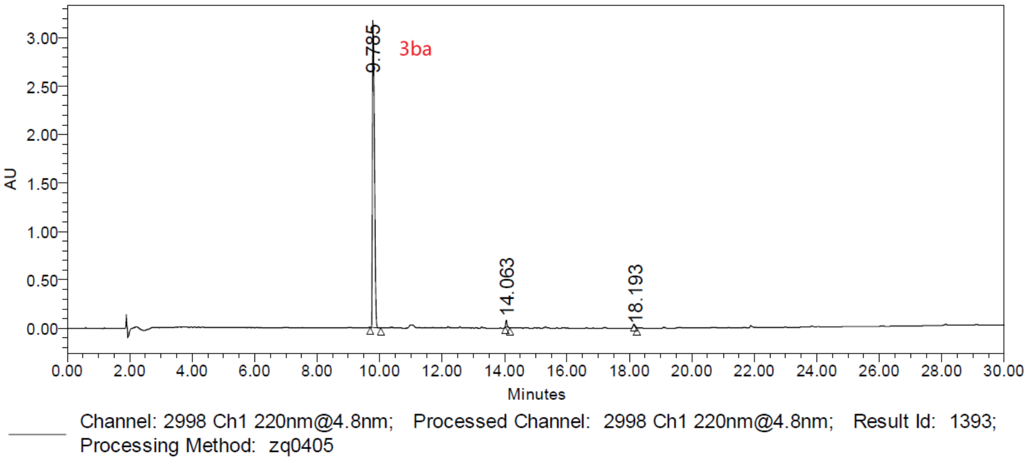

Processed Channel Descr.: 2998 Ch1 220nm@4.8nm

	Processed Channel Descr.	RT	Area	% Area	Height
1	2998 Ch1 220nm@4.8nm	9.785	14641046	98.51	3168189
2	2998 Ch1 220nm@4.8nm	14.063	184181	1.24	67441
3	2998 Ch1 220nm@4.8nm	18.193	37580	0.25	-11364

Figure S101. HPLC-UV chromatogram at 220 nm of **3ba**.

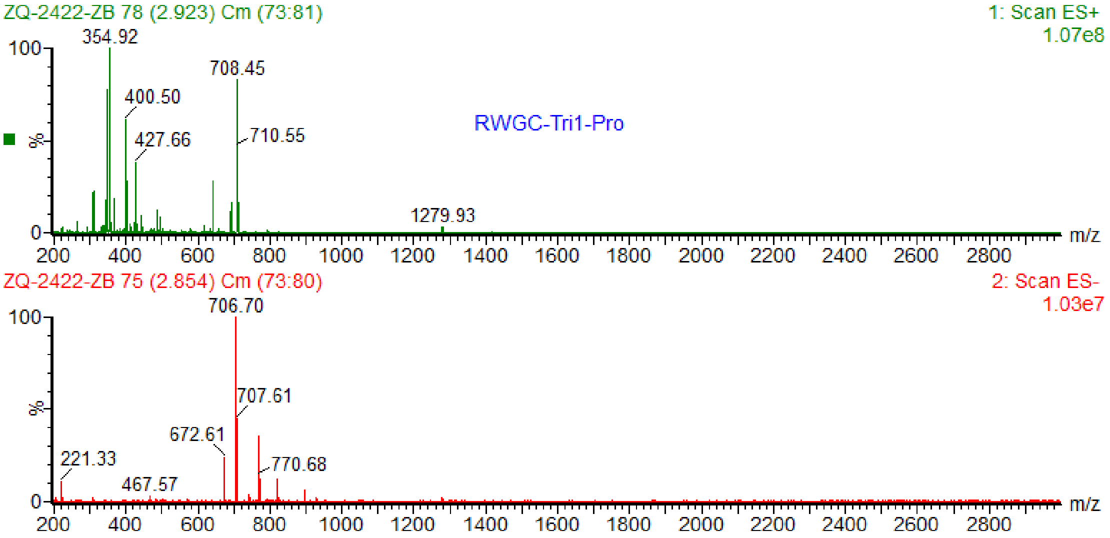

Figure S102. ESI-MS spectrum of **3ba**.

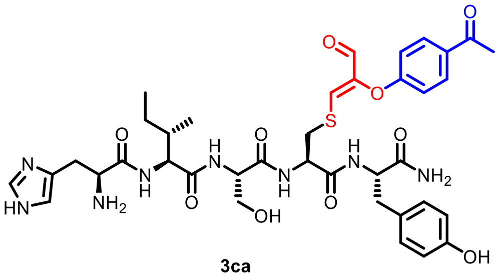

3ca

According to the general procedure A, **1c** (31.0 mg, 0.05 mmol) reacted with **2a** (12.7 mg, 0.06 mmol) to afford the white solid **3ca** (31.5 mg) in 78.0% isolated yield.

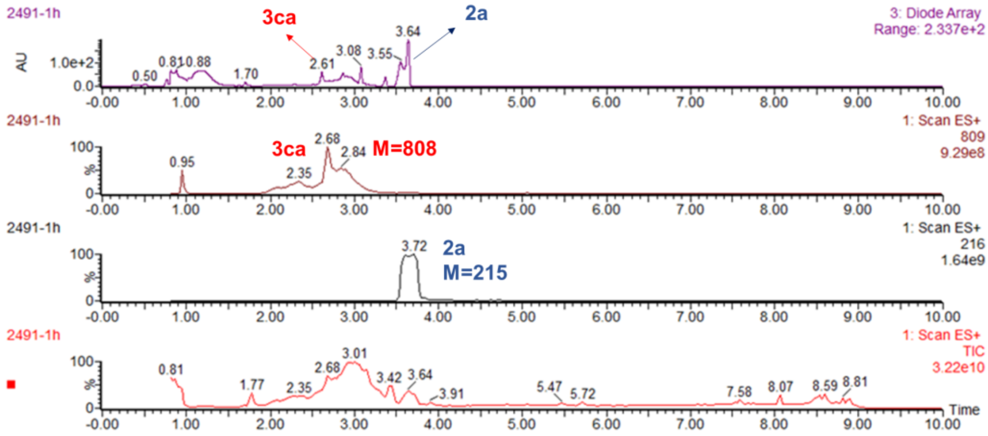

Figure S103. UPLC-MS chromatogram of reaction mixture including TIC and UV curve.

(Extract **2a** and **3ca** mass chromatograms from full scan data).

Analytical **HPLC** using Method D, RT = 9.129 min, the HPLC purity is 96.54%. **LRMS** (ESI+) *m*/*z*: 809.60 [M + H]$^+$, (ESI-) *m*/*z*: 807.64 [M - H]$^-$. **HRMS** (ES+) *m*/*z*: [M + H]$^+$ calcd for $C_{38}H_{48}N_8O_{10}S^+$ 809.3292, found 809.3292.

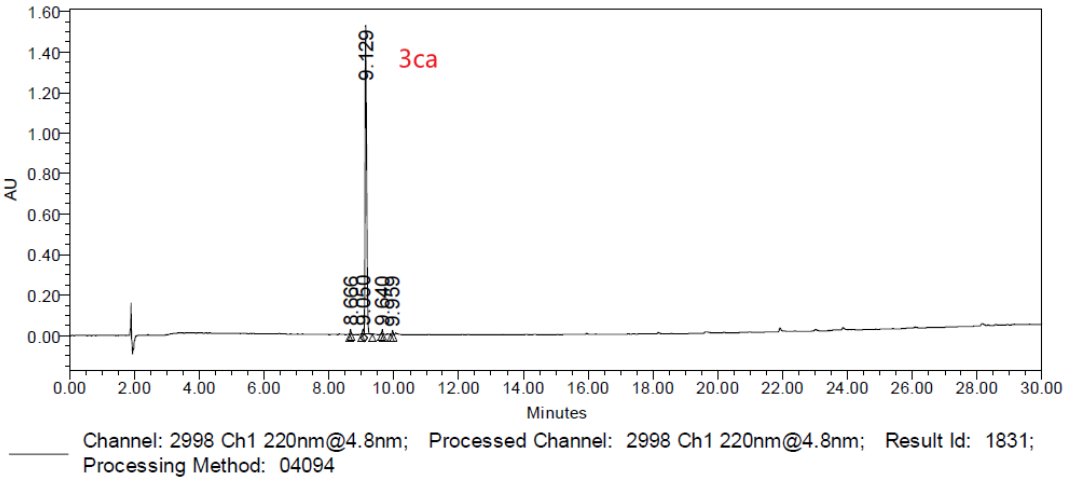

Processed Channel Descr.: 2998 Ch1 220nm@4.8nm

	Processed Channel Descr.	RT	Area	% Area	Height
1	2998 Ch1 220nm@4.8nm	8.666	37798	0.73	20018
2	2998 Ch1 220nm@4.8nm	9.050	66893	1.28	26020
3	2998 Ch1 220nm@4.8nm	9.129	5025845	96.54	1526367
4	2998 Ch1 220nm@4.8nm	9.640	41123	0.79	21195
5	2998 Ch1 220nm@4.8nm	9.959	34330	0.66	17993

Figure S104. HPLC-UV chromatogram at 220 nm of **3ca**.

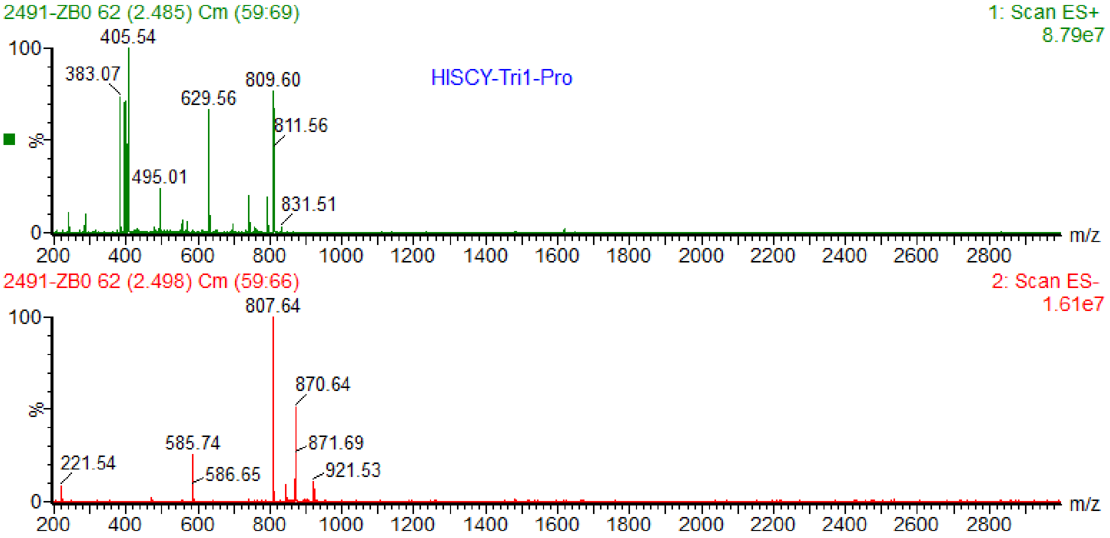

Figure S105. ESI-MS spectrum of **3ca**.

Analysis Report

Agilent Trusted Answers

+ Scan (rt: 4.849 min) Sub (2)   C38 H48 N8 O10 S

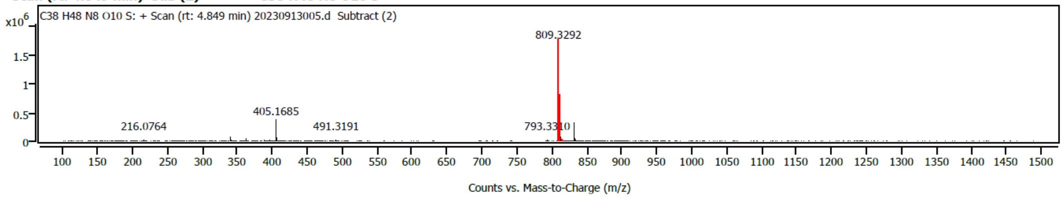

Spectrum Peaks

m/z	Z	Abund	Abund %	m/z (Calc)	Diff (ppm)	Ion Species	Formula	Ion Type
809.3292	1	1757817	100.00	809.3287	0.61	(M+H)+	C38 H48 N8 O10 S	
810.3327	1	820541	46.68	810.3316	1.38	(M+H)+	C38 H48 N8 O10 S	
811.3315	1	313263	17.82	811.3317	-0.15	(M+H)+	C38 H48 N8 O10 S	
812.3314	1	80792	4.60	812.3326	-1.41	(M+H)+	C38 H48 N8 O10 S	
216.0764		24820	1.41					
340.2590		70163	3.99					
362.2410		41368	2.35					
405.1685	2	373919	21.27					
405.6699	2	170576	9.70					
406.1703	2	64809	3.69					
491.3191		19862	1.13					
793.3310		17970	1.02					
831.3101	1	318180	18.10					
832.3129	1	140590	8.00					
833.3126	1	49249	2.80					

Spectrum Identification Table

Best	ID Source	Name	Formula	Species	m/z	Diff (ppm)	CAS	Score	Score (Lib)	Score (DB)	Score (MFG)	Lib/DB
Yes	MFG		C38 H48 N8 O10 S	(M+H)+	809.3292	0.68		99.27			99.27	

Figure S106. Q-TOF-HRMS spectrum of **3ca**.

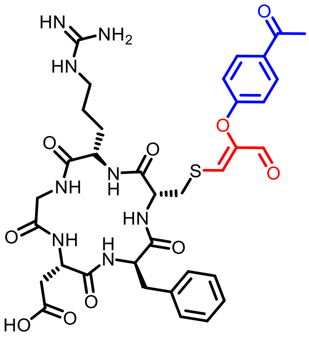

3da

According to the general procedure A, **1d** (28.9 mg, 0.05 mmol) reacted with **2a** (12.7 mg, 0.06 mmol) to afford the white solid **3da** (36.0 mg) in 94.0% isolated yield.

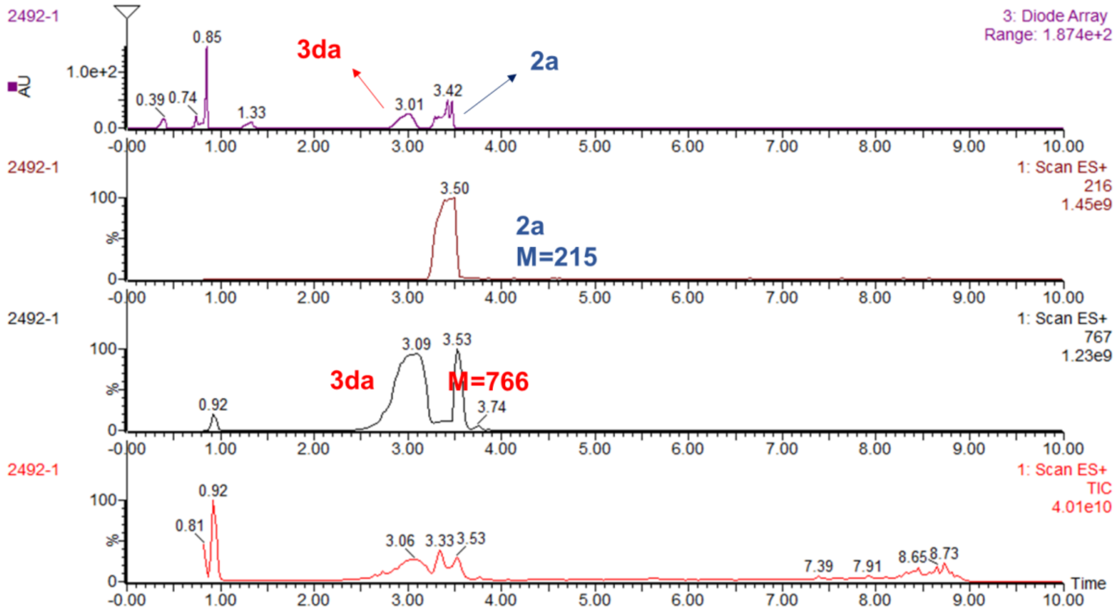

Figure S107. UPLC-MS chromatogram of reaction mixture including TIC and UV curve.

(Extract **2a** and **3da** mass chromatograms from full scan data).

Analytical **HPLC** using Method D, RT = 11.202 min, the HPLC purity is 98.70%. **LRMS** (ESI+) *m*/*z*: 767.46 [M + H]$^+$, (ESI-) *m*/*z*: 765.64 [M - H]$^-$. **HRMS** (ES+) *m*/*z*: [M + H]$^+$ calcd for $C_{35}H_{42}N_8O_{10}S^+$ 767.2822, found 767.2827.

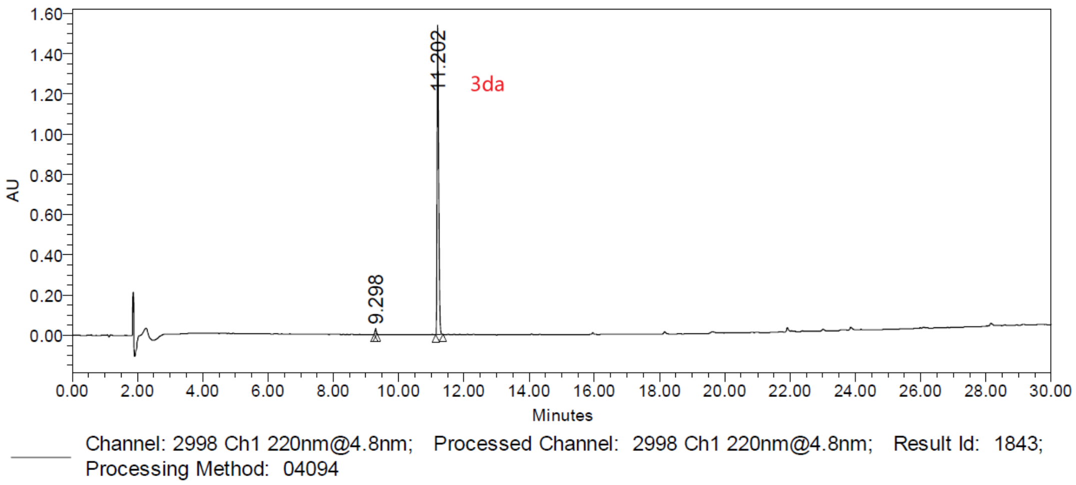

Processed Channel Descr.: 2998 Ch1 220nm@4.8nm

	Processed Channel Descr.	RT	Area	% Area	Height
1	2998 Ch1 220nm@4.8nm	9.298	68343	1.30	26010
2	2998 Ch1 220nm@4.8nm	11.202	5188667	98.70	1536862

Figure S108. HPLC-UV chromatogram at 220 nm of **3da**.

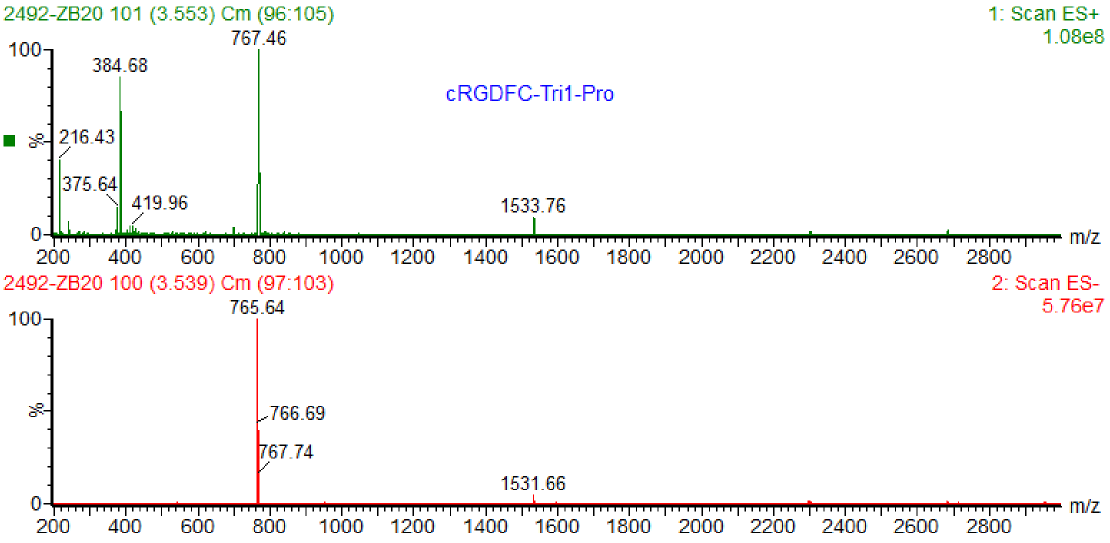

Figure S109. ESI-MS spectrum of **3da**.

Analysis Report

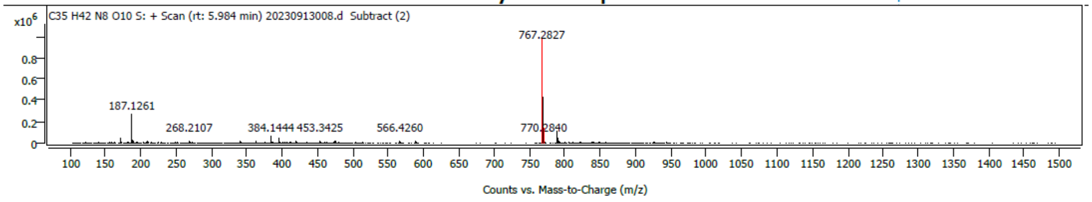

Spectrum Peaks

m/z	z	Abund	Abund %	m/z (Calc)	Diff (ppm)	Ion Species	Formula	Ion Type
767.2827	1	962187	100.00	767.2817	1.31	(M+H)+	C35 H42 N8 O10 S	
768.2848	1	432055	44.90	768.2846	0.19	(M+H)+	C35 H42 N8 O10 S	
769.2839	1	143556	14.92	769.2844	-0.58	(M+H)+	C35 H42 N8 O10 S	
770.2840	1	35427	3.68	770.2853	-1.71	(M+H)+	C35 H42 N8 O10 S	
171.1485		45384	4.72					
187.1261	1	268088	27.86					
188.1286	1	29892	3.11					
189.1215	1	12578	1.31					
209.1091		15854	1.65					
224.1843		10549	1.10					
251.1827		11205	1.16					
268.2107		15084	1.57					
340.2588		19663	2.04					
362.2401		11722	1.22					
384.1444	2	64585	6.71					
384.6464	2	27394	2.85					
385.1460	2	9848	1.02					
395.1359	2	47234	4.91					
395.6364	2	19353	2.01					
411.1042		10840	1.13					
419.6806		14897	1.55					
453.3425		17900	1.86					
474.3281		14747	1.53					
475.3248		16951	1.76					
566.4260		13114	1.36					
588.4085		14128	1.47					
789.2624	1	109918	11.42					
790.2654	1	45806	4.76					
791.2645	1	14780	1.54					
799.3059		10832	1.13					

Spectrum Identification Table

Best	ID Source	Name	Formula	Species	m/z	Diff (ppm)	CAS	Score	Score (Lib)	Score (DB)	Score (MFG)	Lib/DB
Yes	MFG		C35 H42 N8 O10 S	(M+H)+	767.2827	0.76		97.98			97.98	

Figure S110. Q-TOF-HRMS spectrum of **3da**.

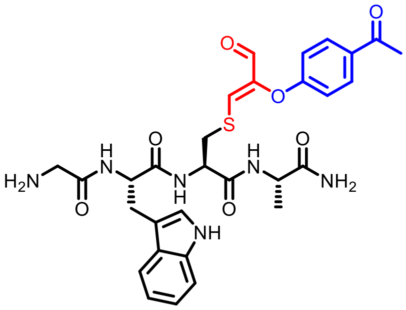

3ea

According to the general procedure A, **1e** (21.7 mg, 0.05 mmol) reacted with **2a** (12.7 mg, 0.06 mmol) to afford the white solid **3ea** (27.2 mg) in 87.5% isolated yield.

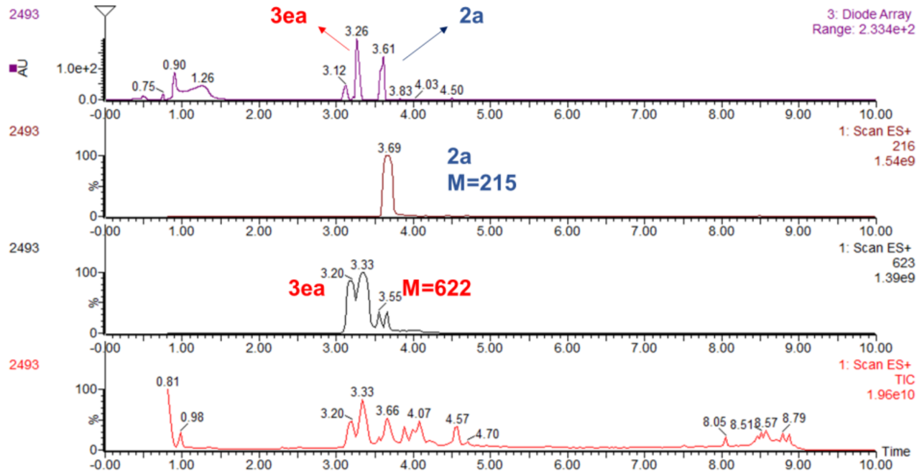

Figure S111. UPLC-MS chromatogram of reaction mixture including TIC and UV curve.

(Extract **2a** and **3ea** mass chromatograms from full scan data).

Analytical **HPLC** using Method D, RT = 10.261 min, the HPLC purity is 96.51%. **LRMS** (ESI+) *m*/*z*: 623.40 [M + H]$^+$, (ESI-) *m*/*z*: 621.65 [M - H]$^-$. **HRMS** (ES+) *m*/*z*: [M + H]$^+$ calcd for $C_{30}H_{34}N_6O_7S^+$ 623.2288, found 623.2284.

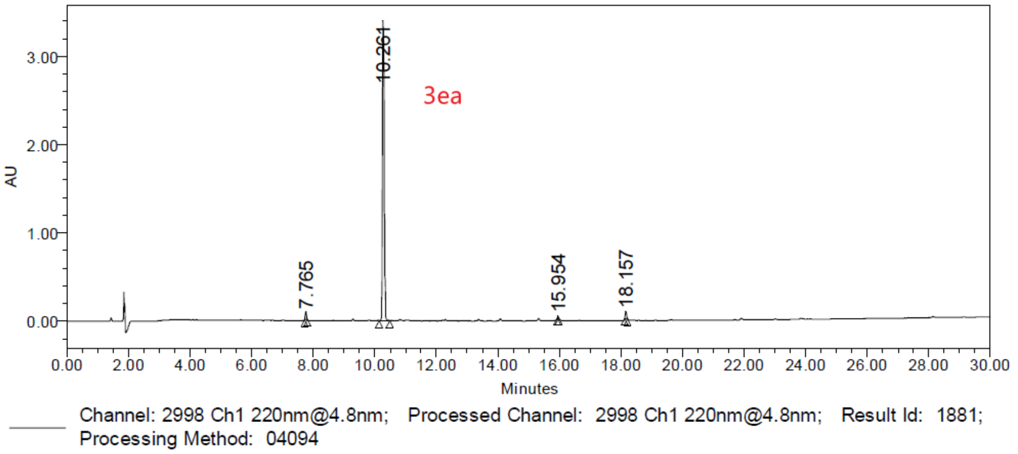

Processed Channel Descr.: 2998 Ch1 220nm@4.8nm

	Processed Channel Descr.	RT	Area	% Area	Height
1	2998 Ch1 220nm@4.8nm	7.765	244318	1.58	84053
2	2998 Ch1 220nm@4.8nm	10.261	14890308	96.51	3398416
3	2998 Ch1 220nm@4.8nm	15.954	41980	0.27	24167
4	2998 Ch1 220nm@4.8nm	18.157	251650	1.63	83760

Figure S112. HPLC-UV chromatogram at 220 nm of **3ea**.

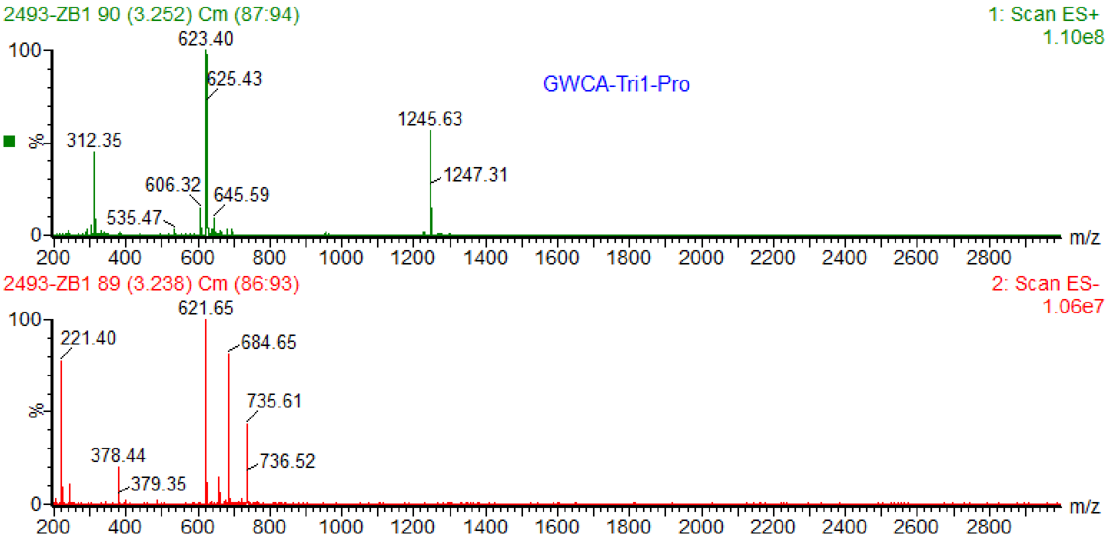

Figure S113. ESI-MS spectrum of **3ea**.

Analysis Report

Agilent Trusted Answers

+ Scan (rt: 5.564 min) Sub (2) **C30 H34 N6 O7 S**

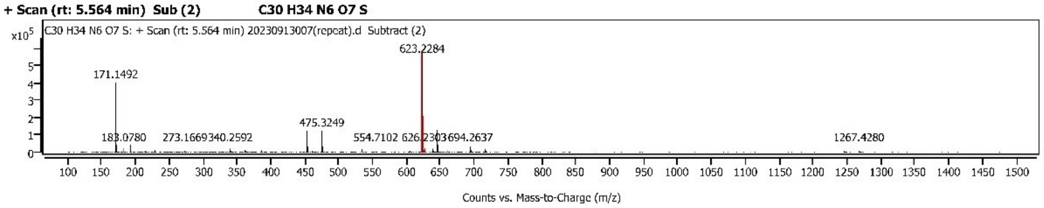

Spectrum Peaks

m/z	z	Abund	Abund %	m/z (Calc)	Diff (ppm)	Ion Species	Formula	Ion Type
623.2284	1	575292	100.00	623.2282	0.32	(M+H)+	C30 H34 N6 O7 S	
624.2311	1	201304	34.99	624.2312	-0.16	(M+H)+	C30 H34 N6 O7 S	
625.2293	1	62935	10.94	625.2302	-1.43	(M+H)+	C30 H34 N6 O7 S	
626.2303	1	13726	2.39	626.2311	-1.35	(M+H)+	C30 H34 N6 O7 S	
171.1492	1	404456	70.30					
172.1523	1	44380	7.71					
183.0780		21370	3.71					
193.0773		18332	3.19					
193.1305		45345	7.88					
215.1271		6737	1.17					
229.2005		11241	1.95					
273.1669		6664	1.16					
312.1184		16084	2.80					
340.2592		20428	3.55					
341.2768		5943	1.03					
362.2405		13853	2.41					
363.2638		6589	1.15					
386.2747		10729	1.87					
446.2948		7579	1.32					
453.3431	1	123442	21.46					
454.3457	1	33834	5.88					
460.3109	1	24537	4.27					
461.3137	1	5973	1.04					
475.3249	1	123689	21.50					
476.3263	1	34124	5.93					
534.3477		17891	3.11					
554.7102		5970	1.04					
605.2157		7249	1.26					
627.2232		7143	1.24					
639.2210	1	20551	3.57					
640.2257	1	7614	1.32					
645.2095	1	130965	22.76					
646.2121	1	45154	7.85					
647.2113	1	14152	2.46					
661.1906		10832	1.88					
694.2637	1	34706	6.03					
695.2670	1	14771	2.57					
716.2459	1	19200	3.34					
717.2491	1	8105	1.41					
1267.4280		7246	1.26					

Spectrum Identification Table

Best	ID Source	Name	Formula	Species	m/z	Diff (ppm)	CAS	Score	Score (Lib)	Score (DB)	Score (MFG)	Lib/DB
Yes	MFG		C30 H34 N6 O7 S	(M+H)+	623.2284	0.05		99.25			99.25	

Figure S114. Q-TOF-HRMS spectrum of **3ea**.

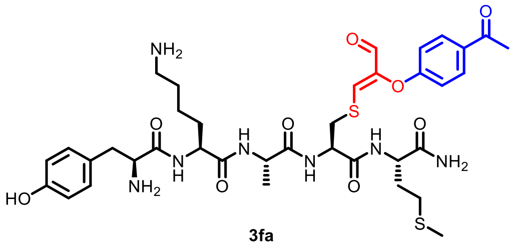

3fa

According to the general procedure A, **1f** (21.0 mg, 0.034 mmol) reacted with **2a** (8.8 mg, 0.041 mmol) to afford the white solid **3fa** (21.9 mg) in 80.5% isolated yield.

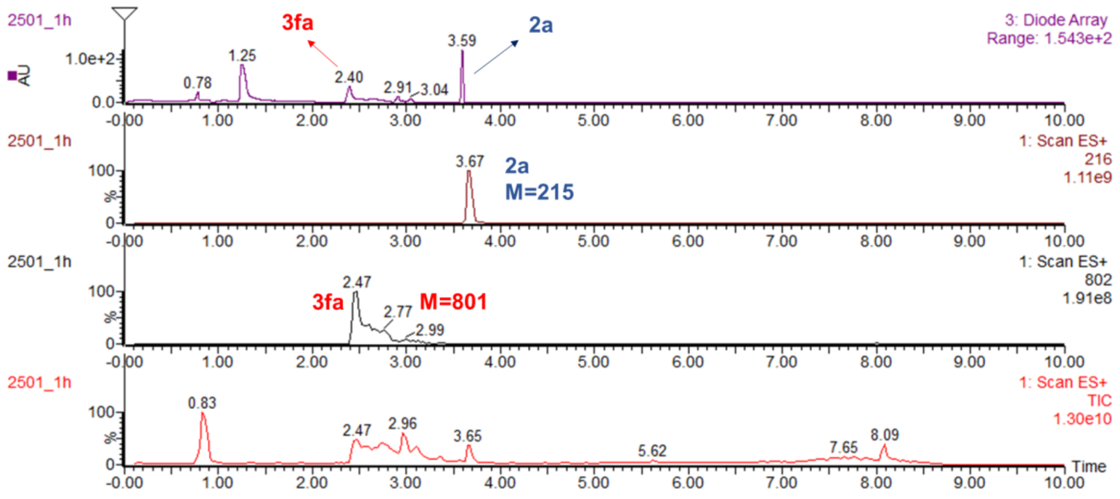

Figure S115. UPLC-MS chromatogram of reaction mixture including TIC and UV curve.

(Extract **2a** and **3fa** mass chromatograms from full scan data).

Analytical **HPLC** using Method D, RT = 8.975 min, the HPLC purity is 98.10%. **LRMS** (ESI+) *m*/*z*: 802.74 [M + H]$^+$, (ESI-) *m*/*z*: 800.71 [M - H]$^-$. **HRMS** (ES+) *m*/*z*: [M + H]$^+$ calcd for $C_{37}H_{51}N_7O_9S_2^+$ 802.3267, found 802.3257.

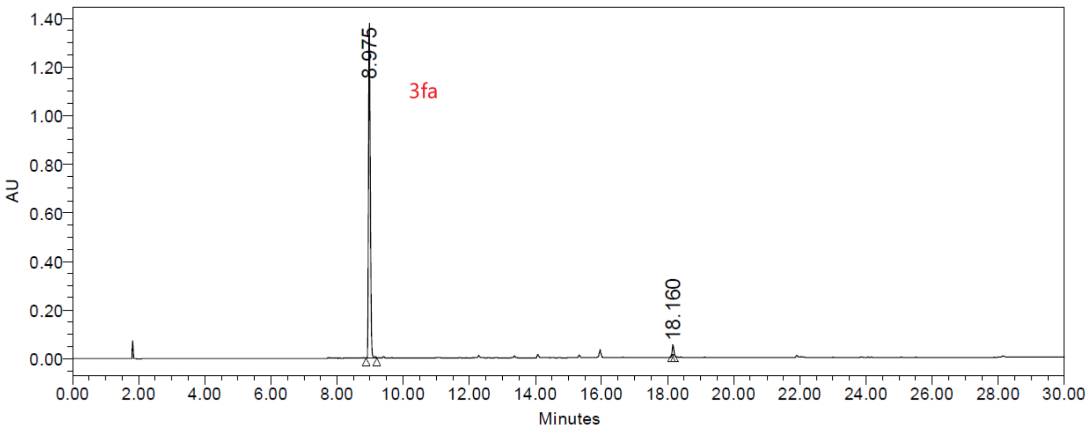

Channel: 2998 Ch2 280nm@4.8nm; Processed Channel: 2998 Ch2 280nm@4.8nm; Result Id: 1925; Processing Method: 04094

Processed Channel Descr.: 2998 Ch2 280nm@4.8nm

	Processed Channel Descr.	RT	Area	% Area	Height
1	2998 Ch2 280nm@4.8nm	8.975	5177109	98.10	1375546
2	2998 Ch2 280nm@4.8nm	18.160	100356	1.90	37049

Figure S116. HPLC-UV chromatogram at 280 nm of **3fa**.

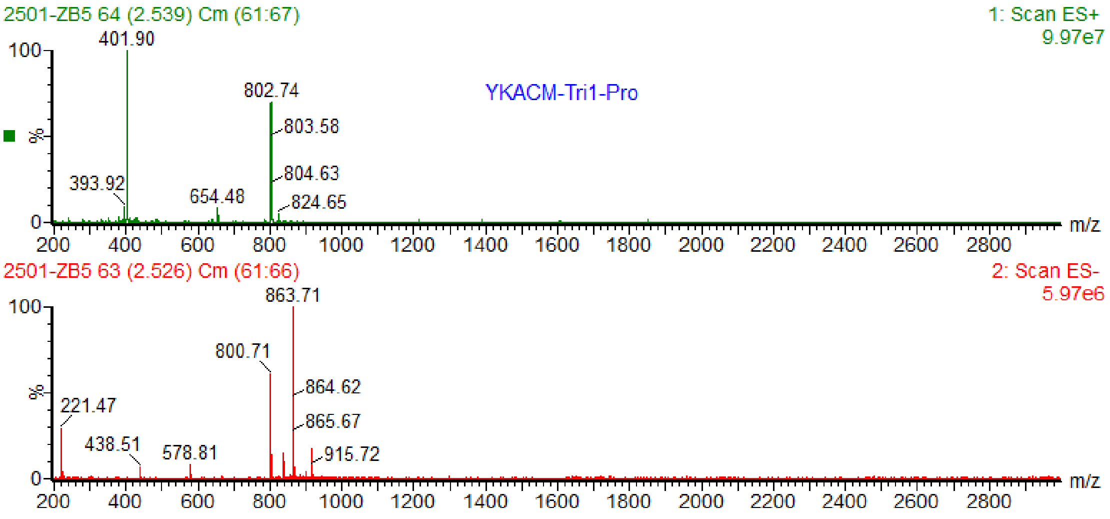

Figure S117. ESI-MS spectrum of **3fa**.

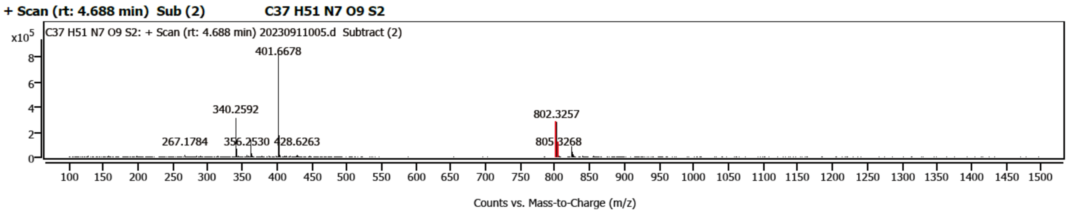

Spectrum Peaks

m/z	Z	Abund	Abund %	m/z (Calc)	Diff (ppm)	Ion Species	Formula	Ion Type
802.3257	1	279407	34.13	802.3262	-0.63	(M+H)+	C37 H51 N7 O9 S2	
803.3278	1	122493	14.96	803.3291	-1.71	(M+H)+	C37 H51 N7 O9 S2	
804.3262	1	54889	6.70	804.3276	-1.67	(M+H)+	C37 H51 N7 O9 S2	
805.3268	1	16380	2.00	805.3284	-1.98	(M+H)+	C37 H51 N7 O9 S2	
267.1784		10717	1.31					
289.1613		8932	1.09					
340.2592	1	312982	38.23					
341.2617	1	61895	7.56					
356.2530		10195	1.25					
362.2409	1	129757	15.85					
363.2436	1	28061	3.43					
401.6678	2	818708	100.00					
402.1687	2	385314	47.06					
402.6680	2	173618	21.21					
403.1683	2	52962	6.47					
403.6680		13901	1.70					
428.6263		15019	1.83					
824.3063	1	83302	10.17					
825.3097	1	36858	4.50					
826.3079	1	17036	2.08					

Spectrum Identification Table

Best	ID Source	Name	Formula	Species	m/z	Diff (ppm)	CAS	Score	Score (Lib)	Score (DB)	Score (MFG)	Lib/DB
Yes	MFG		C37 H51 N7 O9 S2	(M+H)+	802.3257	-1.08		98.18			98.18	

Figure S118. Q-TOF-HRMS spectrum of **3fa**.

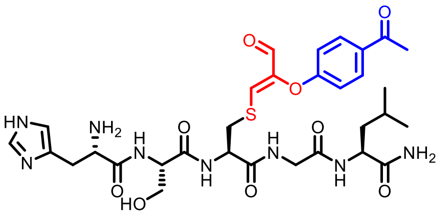

3ga

According to the general procedure A, **1g** (42.1 mg, 0.081 mmol) reacted with **2a** (21 mg, 0.098 mmol) to afford the white solid **3ga** (41.8 mg) in 73.5% isolated yield.

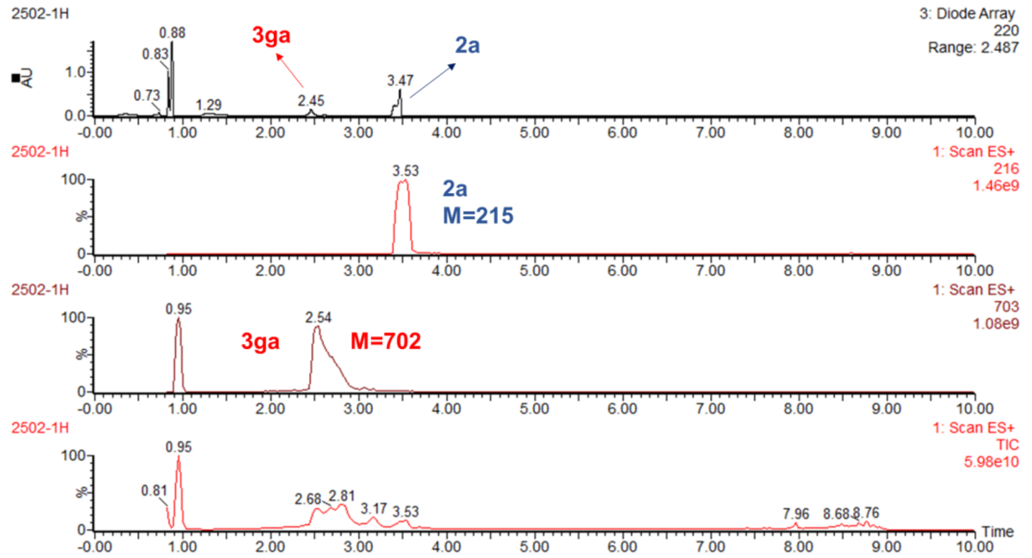

Figure S119. UPLC-MS chromatogram of reaction mixture including TIC and UV curve.

(Extract **2a** and **3ga** mass chromatograms from full scan data).

Analytical **HPLC** using Method D, RT = 9.024 min, the HPLC purity is 98.45%. **LRMS** (ESI+) *m*/*z*: 703.55 $[M + H]^+$, (ESI-) *m*/*z*: 701.66 $[M - H]^-$. **HRMS** (ES+) *m*/*z*: $[M + H]^+$ calcd for $C_{31}H_{42}N_8O_9S^+$ 703.2874, found 703.2870.

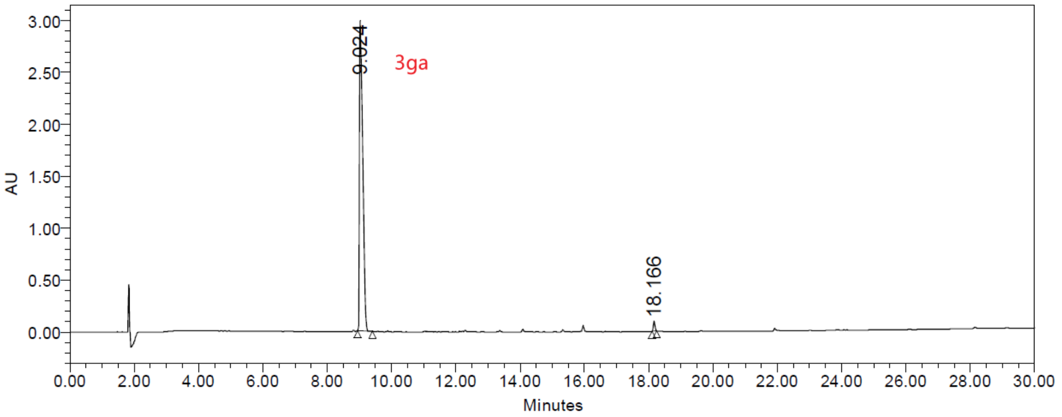

Channel: 2998 Ch1 220nm@4.8nm; Processed Channel: 2998 Ch1 220nm@4.8nm; Result Id: 1923; Processing Method: 04094

Processed Channel Descr.: 2998 Ch1 220nm@4.8nm

	Processed Channel Descr.	RT	Area	% Area	Height
1	2998 Ch1 220nm@4.8nm	9.024	21425228	98.45	2986151
2	2998 Ch1 220nm@4.8nm	18.166	337921	1.55	95236

Figure S120. HPLC-UV chromatogram at 220 nm of **3ga**.

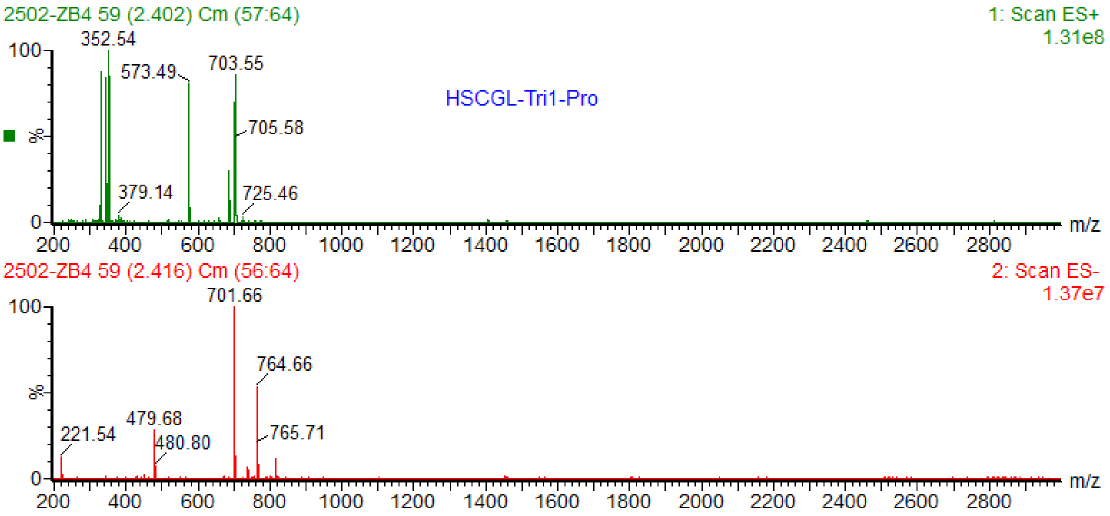

Figure S121. ESI-MS spectrum of **3ga**.

Analysis Report

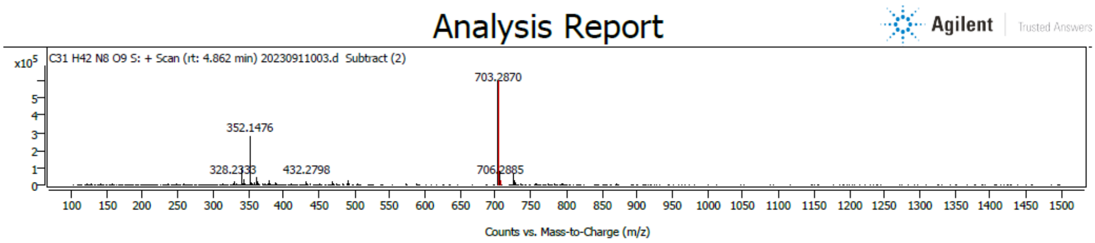

Spectrum Peaks

m/z	Z	Abund	Abund %	m/z (Calc)	Diff (ppm)	Ion Species	Formula	Ion Type
703.2870	1	600066	100.00	703.2868	0.30	(M+H)+	C31 H42 N8 O9 S	
704.2894	1	223214	37.20	704.2897	-0.36	(M+H)+	C31 H42 N8 O9 S	
705.2882	1	76199	12.70	705.2890	-1.16	(M+H)+	C31 H42 N8 O9 S	
706.2885	1	16842	2.81	706.2899	-2.09	(M+H)+	C31 H42 N8 O9 S	
328.2333		6019	1.00					
329.6365	2	17935	2.99					
330.1367	2	6466	1.08					
340.2590	1	99594	16.60					
341.2615	1	20258	3.38					
343.6342	2	30975	5.16					
344.1352	2	10937	1.82					
352.1476	2	280063	46.67					
352.6488	2	108896	18.15					
353.1489	2	35513	5.92					
353.6493	2	9241	1.54					
356.2517		6098	1.02					
358.2430		11006	1.83					
362.2412	1	39264	6.54					
363.1389		15186	2.53					
363.2415	1	9078	1.51					
379.1075	2	25366	4.23					
379.6080	2	10590	1.76					
380.1081	2	7102	1.18					
388.2537		14274	2.38					
432.2798		15613	2.60					
469.3377		15430	2.57					
491.3198	1	24475	4.08					
492.3211	1	7458	1.24					
725.2679	1	63064	10.51					
726.2711	1	23558	3.93					
727.2695	1	8910	1.48					
757.2052		6174	1.03					

Spectrum Identification Table

Best	ID Source	Name	Formula	Species	m/z	Diff (ppm)	CAS	Score	Score (Lib)	Score (DB)	Score (MFG)	Lib/DB
Yes	MFG		C31 H42 N8 O9 S	(M+H)+	703.2870	-0.03		99.51			99.51	

Figure S122. Q-TOF-HRMS spectrum of **3ga**.

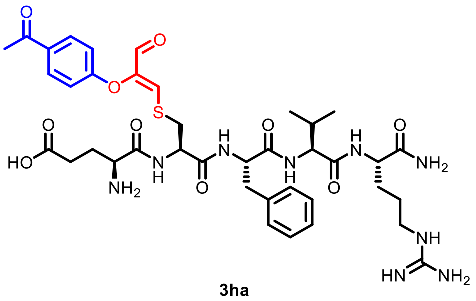

3ha

According to the general procedure A, **1h** (16.2 mg, 0.025 mmol) reacted with **2a** (6.4 mg, 0.03 mmol) to afford the white solid **3ha** (16.9 mg) in 80.5% isolated yield.

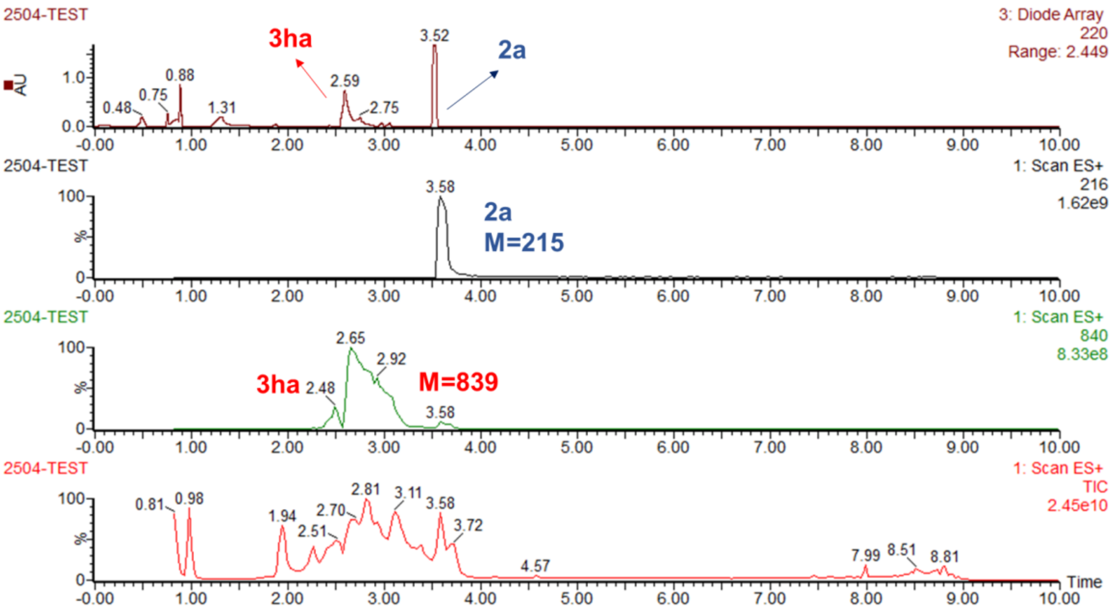

Figure S123. UPLC-MS chromatogram of reaction mixture including TIC and UV curve.

(Extract **2a** and **3ha** mass chromatograms from full scan data).

Analytical **HPLC** using Method D, RT = 9.295 min, the HPLC purity is 95.51%. **LRMS** (ESI+) *m/z*: 840.61 $[M + H]^+$, (ESI-) *m/z*: 838.65 $[M - H]^-$. **HRMS** (ES+) *m/z*: $[M + H]^+$ calcd for $C_{39}H_{53}N_9O_{10}S^+$ 840.3714, found 840.3708.

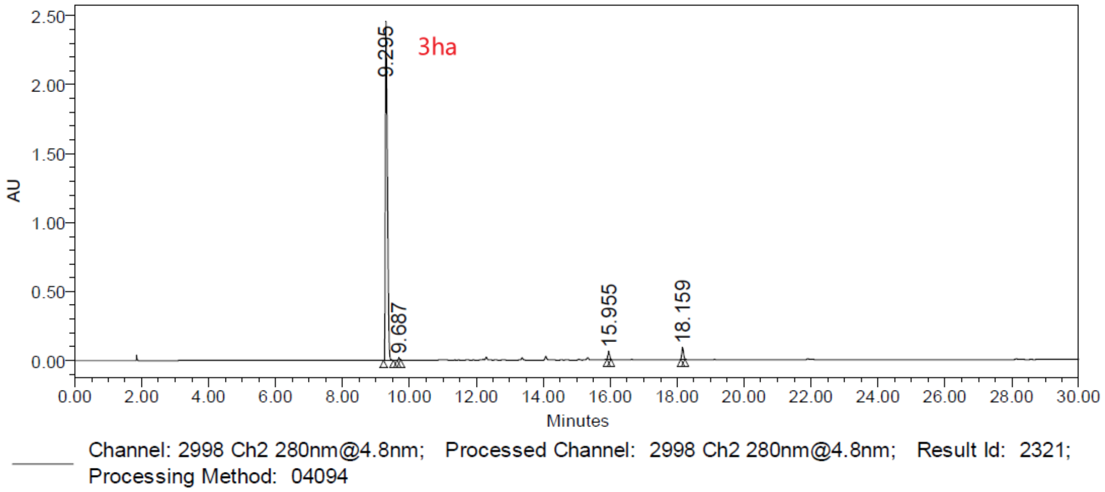

Processed Channel Descr.: 2998 Ch2 280nm@4.8nm

	Processed Channel Descr.	RT	Area	% Area	Height
1	2998 Ch2 280nm@4.8nm	9.295	10405936	95.51	2454652
2	2998 Ch2 280nm@4.8nm	9.687	36443	0.33	15076
3	2998 Ch2 280nm@4.8nm	15.955	175716	1.61	57934
4	2998 Ch2 280nm@4.8nm	18.159	277441	2.55	81435

Figure S124. HPLC-UV chromatogram at 280 nm of **3ha**.

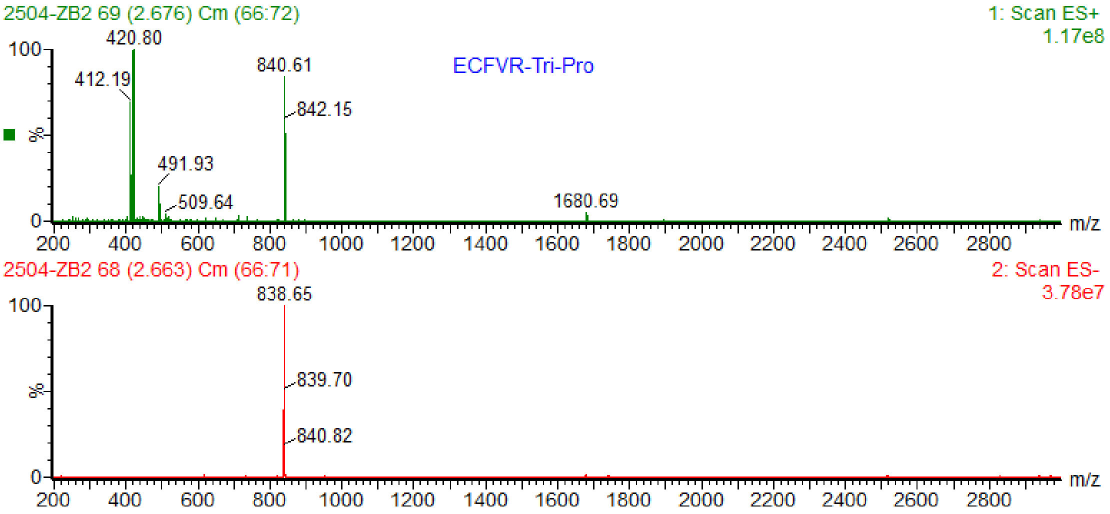

Figure S125. ESI-MS spectrum of **3ha**.

Analysis Report

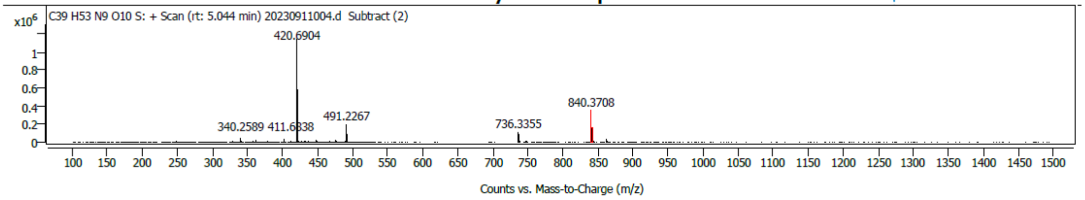

Spectrum Peaks

m/z	Z	Abund	Abund %	m/z (Calc)	Diff (ppm)	Ion Species	Formula	Ion Type
840.3708	1	344176	30.41	840.3709	-0.12	(M+H)+	C39 H53 N9 O10 S	
841.3737	1	156952	13.87	841.3738	-0.10	(M+H)+	C39 H53 N9 O10 S	
842.3733	1	55312	4.89	842.3739	-0.70	(M+H)+	C39 H53 N9 O10 S	
843.3736	1	14551	1.29	843.3748	-1.46	(M+H)+	C39 H53 N9 O10 S	
340.2589		45748	4.04					
362.2405		20481	1.81					
402.2691		27744	2.45					
411.6838		13368	1.18					
420.6904	2	1131904	100.00					
421.1913	2	577102	50.99					
421.6914	2	212154	18.74					
422.1919	2	57311	5.06					
431.6802		16121	1.42					
447.6495		20889	1.85					
467.3229		16386	1.45					
476.3069		17348	1.53					
489.3034		15886	1.40					
491.2267	1	188244	16.63					
491.5608	1	155090	13.70					
491.8946		82243	7.27					
492.2281	1	37512	3.31					
492.5620	1	13636	1.20					
736.3355	2	104232	9.21					
736.8367	2	87784	7.76					
737.3377	2	46450	4.10					
737.8382	2	21199	1.87					
862.3513	1	29423	2.60					
863.3552	1	15949	1.41					

Spectrum Identification Table

Best	ID Source	Name	Formula	Species	m/z	Diff (ppm)	CAS	Score	Score (Lib)	Score (DB)	Score (MFG)	Lib/DB
Yes	MFG		C39 H53 N9 O10 S	(M+H)+	840.3708	-0.20		99.54			99.54	

Figure S126. Q-TOF-HRMS spectrum of **3ha**.

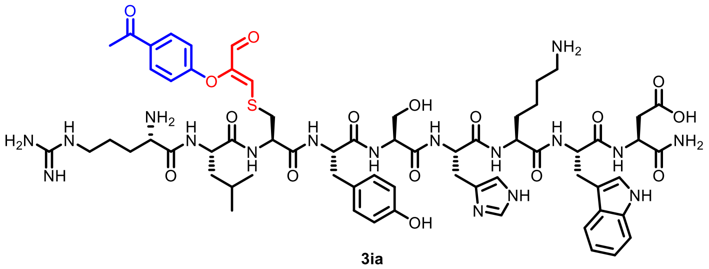

3ia

According to the general procedure A, **1i** (24.1 mg, 0.02 mmol) reacted with **2a** (5.2 mg, 0.024 mmol) to afford the white solid **3ia** (19.8 mg) in 71.4% isolated yield.

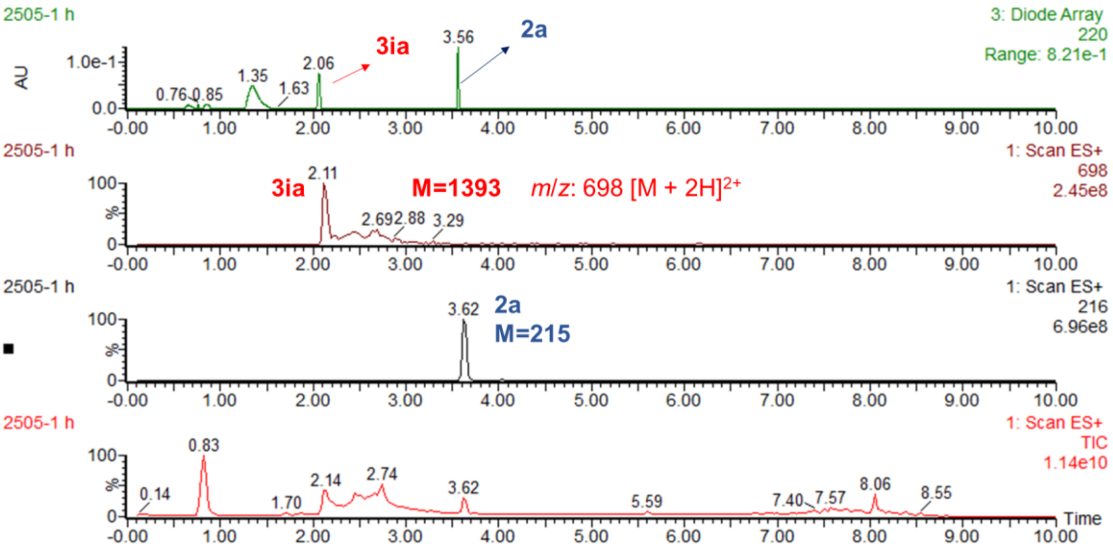

Figure S127. UPLC-MS chromatogram of reaction mixture including TIC and UV curve.

(Extract **2a** and **3ia** mass chromatograms from full scan data).

Analytical **HPLC** using Method D, RT = 9.076 min, the HPLC purity is 99.86%. **LRMS** (ESI+) *m*/*z*: 1394.80 [M + H]$^+$, (ESI-) *m*/*z*: 1392.77 [M - H]$^-$. **HRMS** (*Q-TOF*) *m*/*z*: calcd for $C_{65}H_{87}N_{17}O_{16}S^+$ 1394.6315, found 1394.6333.

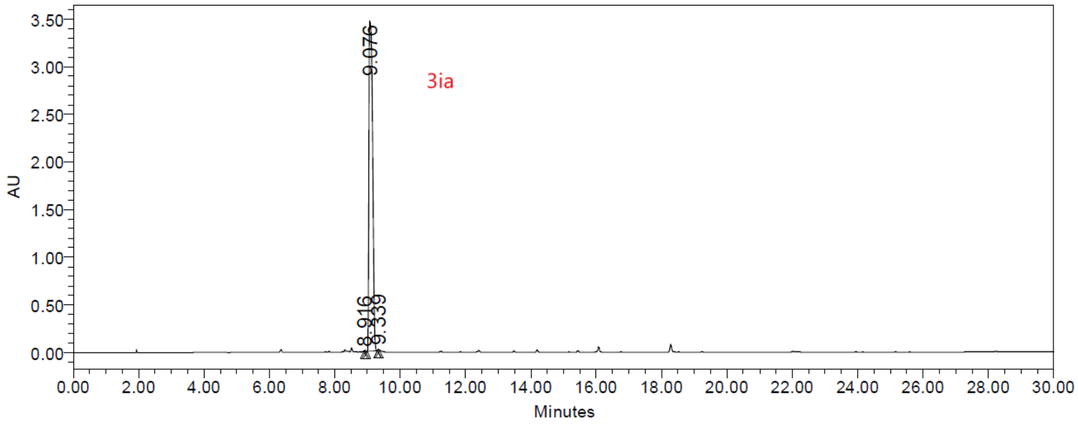

Channel: 2998 Ch2 280nm@4.8nm; Processed Channel: 2998 Ch2 280nm@4.8nm; Result Id: 1958; Processing Method: 04094

Processed Channel Descr.: 2998 Ch2 280nm@4.8nm

	Processed Channel Descr.	RT	Area	% Area	Height
1	2998 Ch2 280nm@4.8nm	8.916	17507	0.06	11415
2	2998 Ch2 280nm@4.8nm	9.076	27171747	99.86	3463782
3	2998 Ch2 280nm@4.8nm	9.339	19926	0.07	11259

Figure S128. HPLC-UV chromatogram at 280 nm of **3ia**.

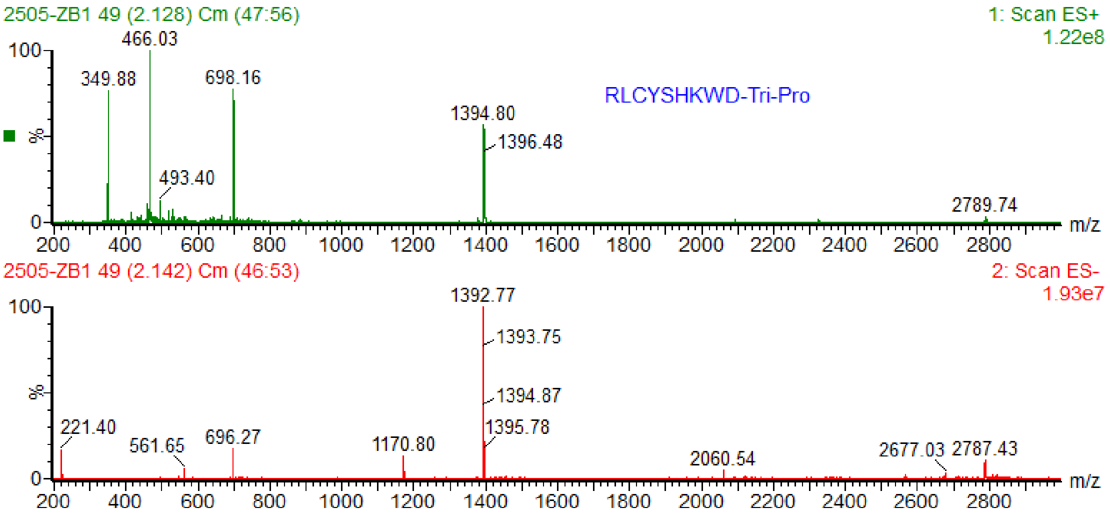

Figure S129. ESI-MS spectrum of **3ia**.

Analysis Report

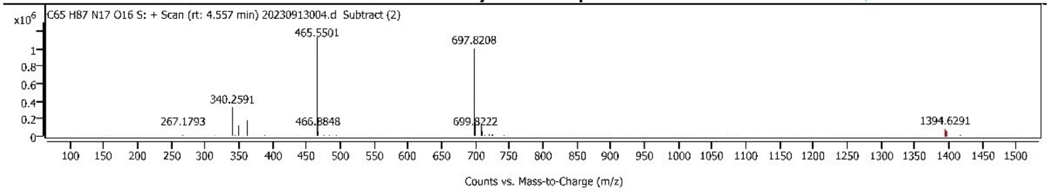

Spectrum Peaks

m/z	Z	Abund	Abund %	m/z (Calc)	Diff (ppm)	Ion Species	Formula	Ion Type
1394.6291	1	82260	7.27	1394.6310	-1.35	(M+H)+	C65 H87 N17 O16 S	
1395.6315	1	65728	5.81	1395.6339	-1.67	(M+H)+	C65 H87 N17 O16 S	
1396.6332	1	29391	2.60	1396.6355	-1.62	(M+H)+	C65 H87 N17 O16 S	
267.1793		12830	1.13					
340.2591	1	327389	28.94					
341.2616	1	64487	5.70					
349.4137	2	120244	10.63					
349.6648	2	91084	8.05					
349.9154	2	46516	4.11					
350.1657	2	17334	1.53					
362.2408	1	178697	15.80					
363.2434	1	35609	3.15					
388.2533		11805	1.04					
465.5501	1	1131256	100.00					
465.8842	1	924666	81.74					
466.2176	1	491219	43.42					
466.5514	1	187470	16.57					
466.8848	1	51868	4.59					
467.2183	1	13435	1.19					
476.2234		11482	1.02					
494.5596		12678	1.12					
697.8208	2	1001620	88.54					
698.3228	2	820772	72.55					
698.8222	2	437363	38.66					
699.3227	2	163136	14.42					
699.8222	2	54403	4.81					
700.3173	2	15434	1.36					
708.8111	2	131405	11.62					
709.3116	2	107858	9.53					
709.8125	2	58422	5.16					
710.3132	2	20543	1.82					
719.8022	2	24820	2.19					
720.3027	2	20089	1.78					
724.2815		12636	1.12					
724.7797	2	32754	2.90					
725.2794	2	23270	2.06					
725.7808	2	15795	1.40					
741.3352		11386	1.01					

Spectrum Identification Table

Best	ID Source	Name	Formula	Species	m/z	Diff (ppm)	CAS	Score	Score (Lib)	Score (DB)	Score (MFG)	Lib/DB
Yes	MFG		C65 H87 N17 O16 S	(M+H)+	1394.6291	-1.71		96.36			96.36	

Figure S130. Q-TOF-HRMS spectrum of **3ia**.

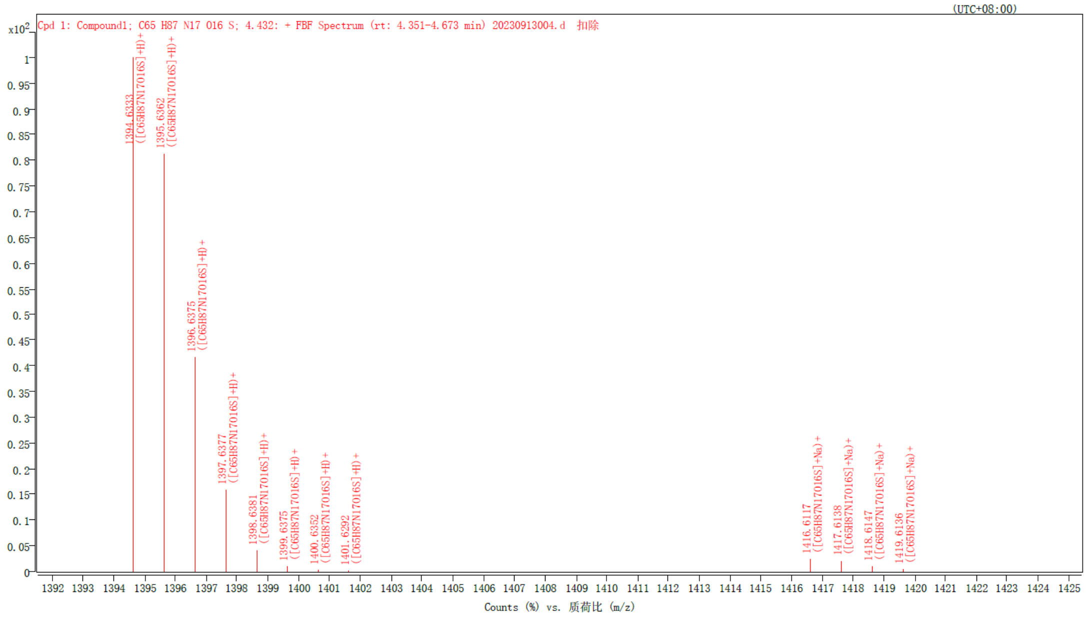

Figure S131. Zoom of Q-TOF-HRMS spectrum of **3ia**.

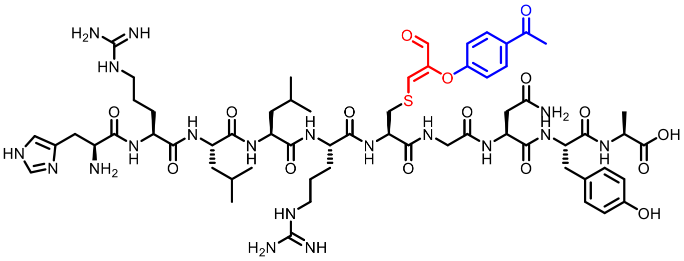

3ja

According to the general procedure A, **1j** (36.1 mg, 0.03 mmol) reacted with **2a** (7.8 mg, 0.036 mmol) to afford the white solid **3ja** (30.6 mg) in 73.4% isolated yield.

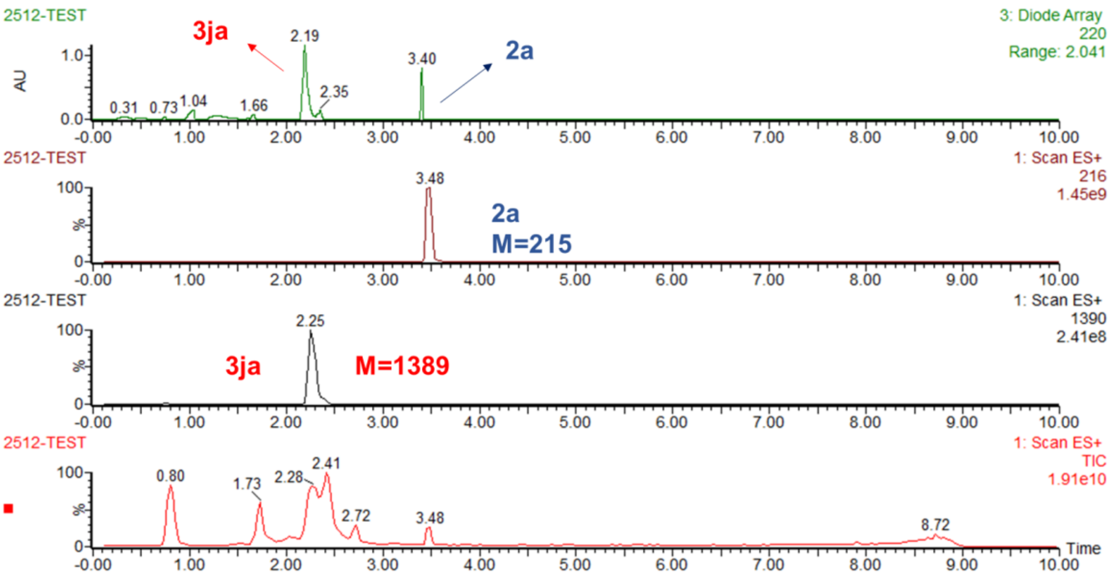

Figure S132. UPLC-MS chromatogram of reaction mixture including TIC and UV curve.

(Extract **2a** and **3ja** mass chromatograms from full scan data).

Analytical **HPLC** using Method D, RT = 9.150 min, the HPLC purity is 97.30%. **LRMS** (ESI+) *m*/*z*: 1390.74 [M + H]$^+$, (ESI-) *m*/*z*: 1388.78 [M - H]$^-$. **HRMS** (ES+) *m*/*z*: [M + H]$^+$ calcd for $C_{62}H_{91}N_{19}O_{16}S^+$ 1390.6689, found 1390.6688.

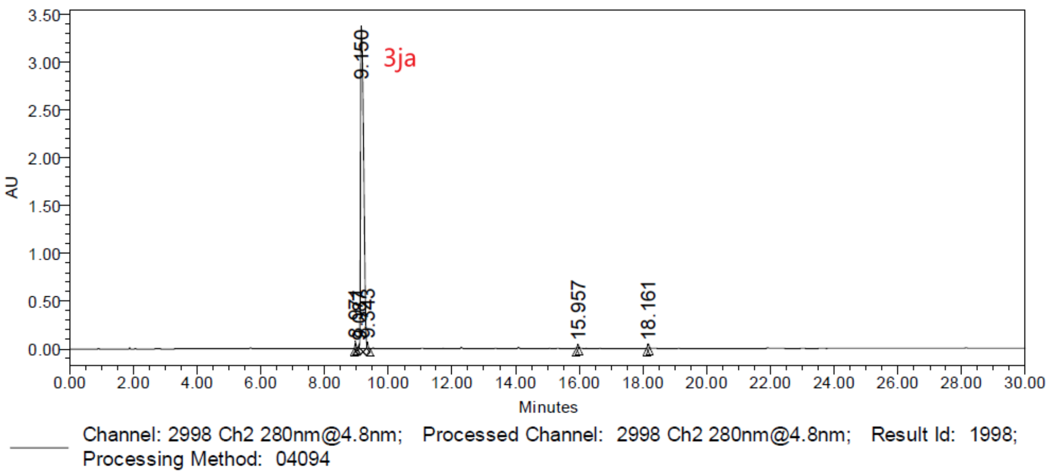

Processed Channel Descr.: 2998 Ch2 280nm@4.8nm

	Processed Channel Descr.	RT	Area	% Area	Height
1	2998 Ch2 280nm@4.8nm	8.971	139103	0.60	66823
2	2998 Ch2 280nm@4.8nm	9.087	93711	0.40	43290
3	2998 Ch2 280nm@4.8nm	9.150	22520670	97.30	3368911
4	2998 Ch2 280nm@4.8nm	9.343	165147	0.71	65796
5	2998 Ch2 280nm@4.8nm	15.957	96900	0.42	35930
6	2998 Ch2 280nm@4.8nm	18.161	129980	0.56	40865

Figure S133. HPLC-UV chromatogram at 280 nm of **3ja**.

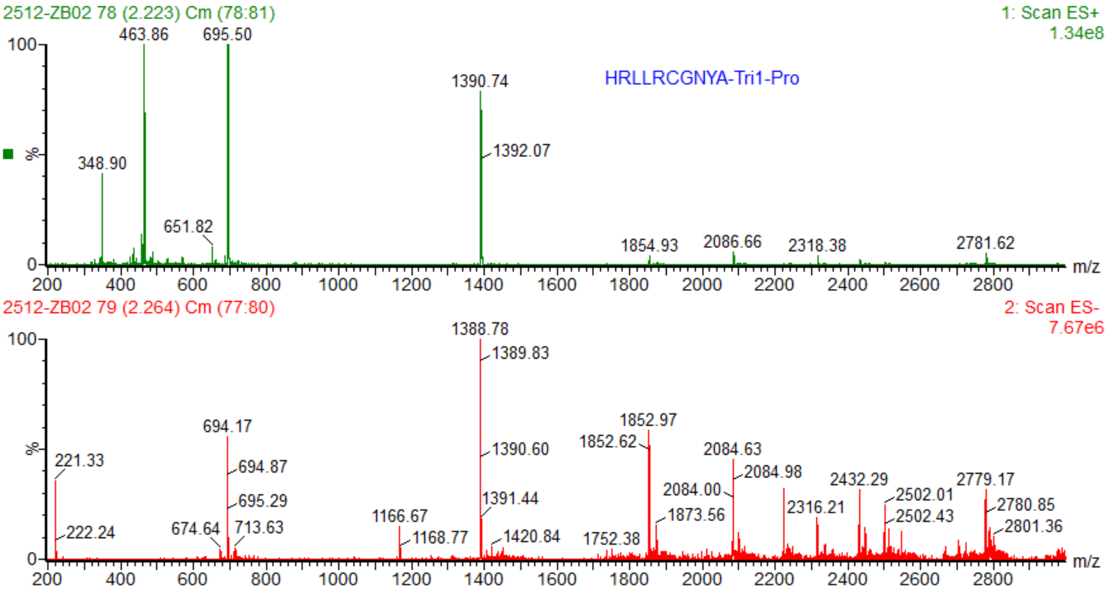

Figure S134. ESI-MS spectrum of **3ja**.

Analysis Report

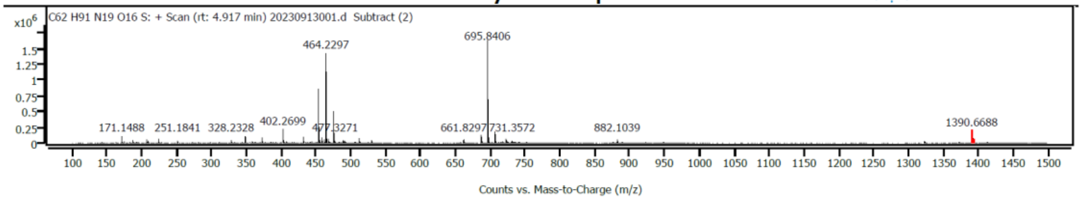

Spectrum Identification Table

Best ID Source	Name	Formula	Species	m/z	Diff (ppm)	CAS	Score	Score (Lib)	Score (DB)	Score (MFG)	Lib/DB
Yes MFG		C62 H91 N19 O16 S	(M+H)+	1390.6688	-0.12		99.42			99.42	

Figure S135. Q-TOF-HRMS spectrum of **3ja**.

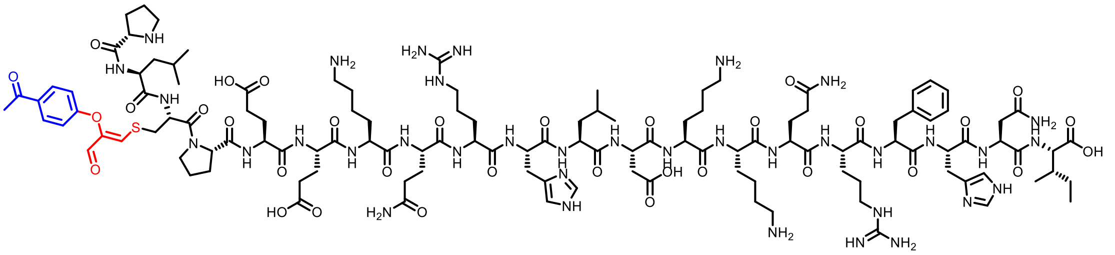

3ka

According to the general procedure A, **1k** (23.2 mg, 0.0092 mmol) reacted with **2a** (2.4 mg, 0.011 mmol) to afford the white solid **3ka** (16.6 mg) in 67.0% isolated yield.

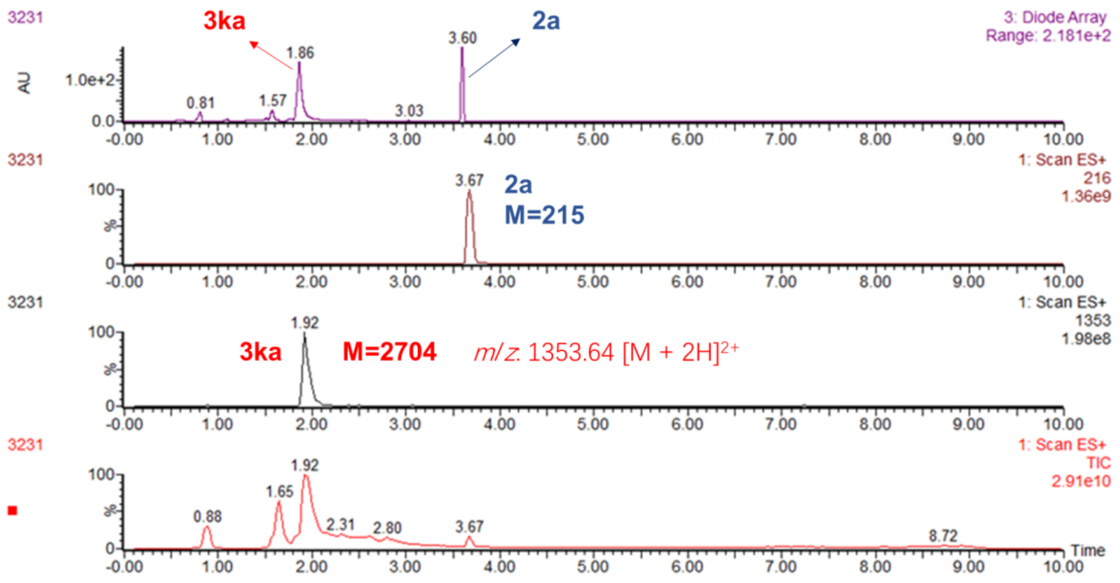

Figure S136. UPLC-MS chromatogram of reaction mixture including TIC and UV curve.

(Extract **2a** and **3ka** mass chromatograms from full scan data).

Analytical **HPLC** using Method D, RT = 8.630 min, the HPLC purity is 100%. **LRMS** (ESI+) *m/z*: 1353.64 $[M + 2H]^{2+}$, (ESI-) *m/z*: 2704.47 $[M - H]^{-}$. **HRMS** (ES+) *m/z*: $[M + 2H]^{2+}$ calcd for $C_{121}H_{186}N_{36}O_{33}S^{+2}$ 1353.1947, found 1353.1966.

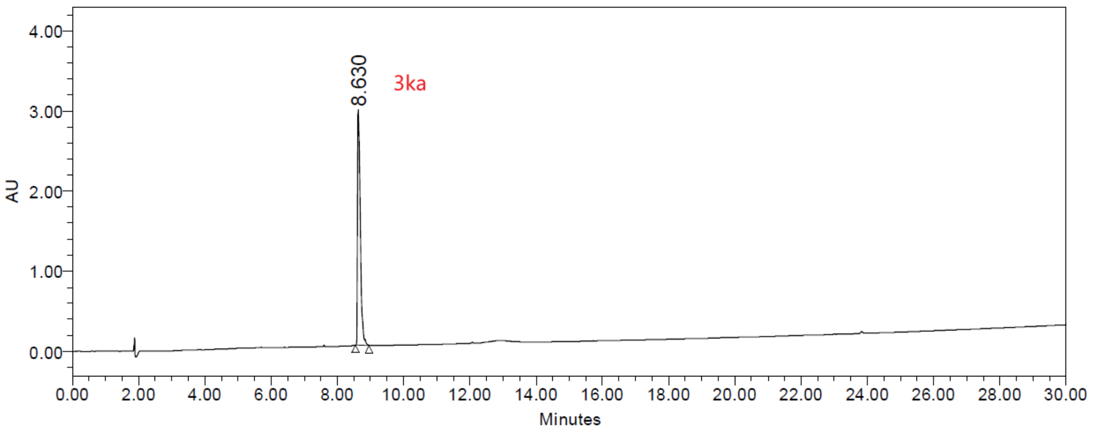

Channel: 2998 Ch1 220nm@4.8nm; Processed Channel: 2998 Ch1 220nm@4.8nm; Result Id: 2590; Processing Method: 04094

Processed Channel Descr.: 2998 Ch1 220nm@4.8nm

	Processed Channel Descr.	RT	Area	% Area	Height
1	2998 Ch1 220nm@4.8nm	8.630	17402628	100.00	2937102

Figure S137. HPLC-UV chromatogram at 220 nm of **3ka**.

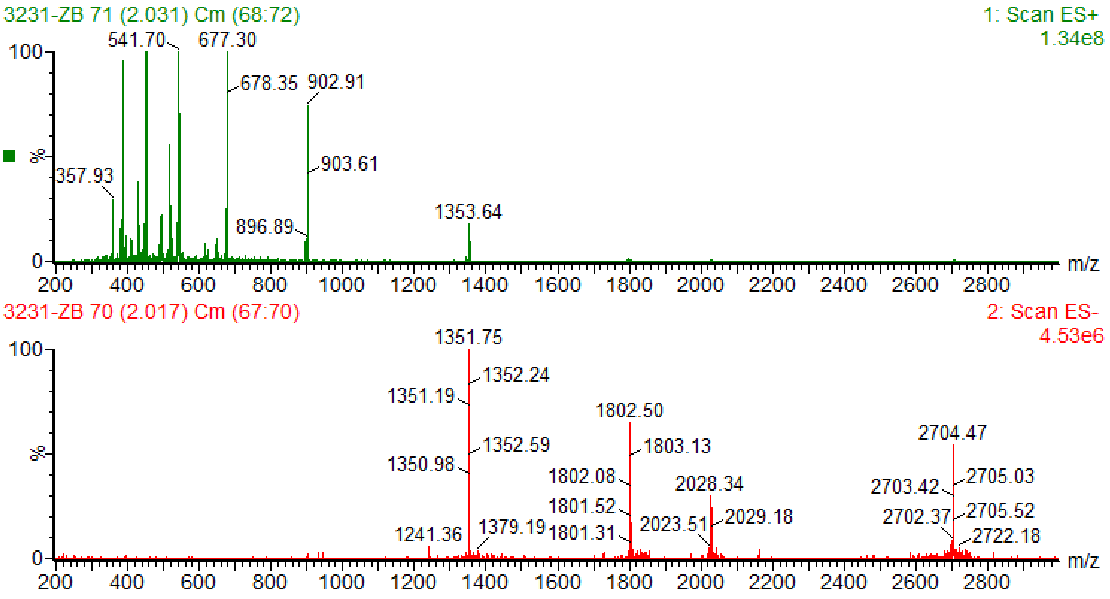

Figure S138. ESI-MS spectrum of **3ka**.

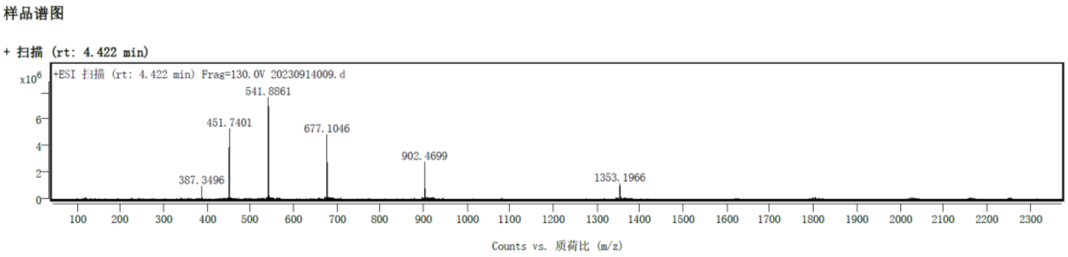

Figure S139. Q-TOF-HRMS spectrum of **3ka**.

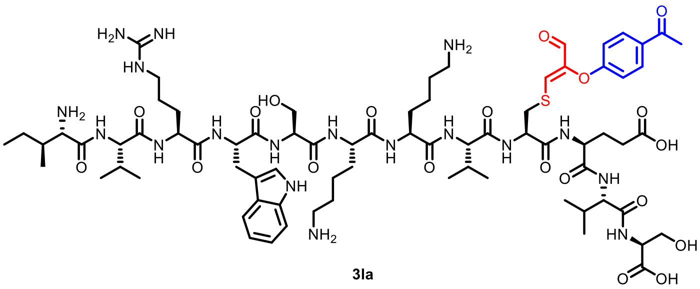

3la

According to the general procedure A, **1l** (43.0 mg, 0.03 mmol) reacted with **2a** (7.7 mg, 0.036 mmol) to afford the white solid **3la** (32.4 mg) in 66.6% isolated yield.

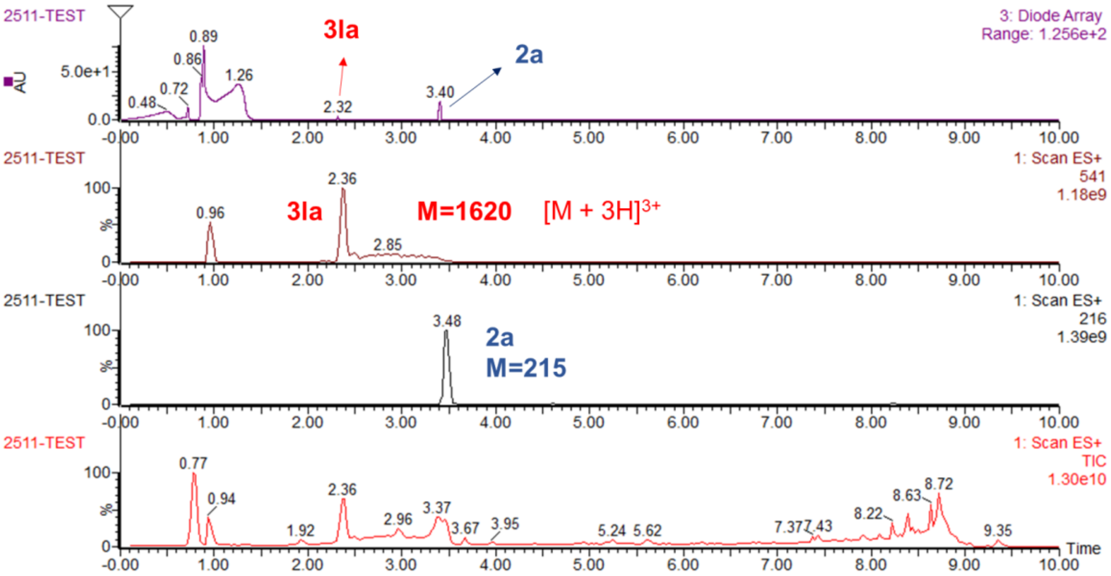

Figure S140. UPLC-MS chromatogram of reaction mixture including TIC and UV curve.

(Extract **2a** and **3la** mass chromatograms from full scan data).

Analytical **HPLC** using Method D, RT = 9.349 min, the HPLC purity is 98.19%. **LRMS** (ESI+) *m/z*: 1621.61 [M + H]$^+$, (ESI-) *m/z*: 1620.00 [M - H]$^-$. **HRMS** (ES+) *m/z*: [M + H]$^+$ calcd for $C_{75}H_{116}N_{18}O_{20}S^+$ 1621.8411, found 1621.8598.

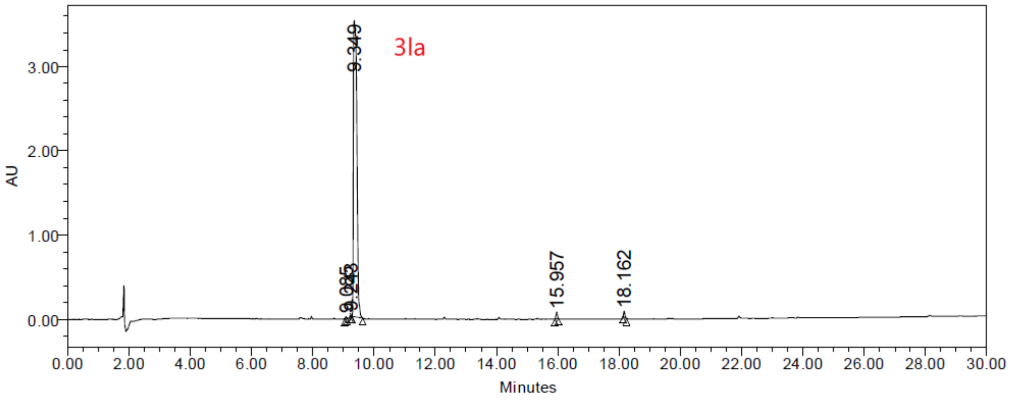

Channel: 2998 Ch1 220nm@4.8nm; Processed Channel: 2998 Ch1 220nm@4.8nm; Result Id: 1982; Processing Method: 04094

Processed Channel Descr.: 2998 Ch1 220nm@4.8nm

	Processed Channel Descr.	RT	Area	% Area	Height
1	2998 Ch1 220nm@4.8nm	9.085	39480	0.14	19795
2	2998 Ch1 220nm@4.8nm	9.243	34778	0.12	23970
3	2998 Ch1 220nm@4.8nm	9.349	27410687	98.19	3510358
4	2998 Ch1 220nm@4.8nm	15.957	229395	0.82	76626
5	2998 Ch1 220nm@4.8nm	18.162	200680	0.72	67174

Figure S141. HPLC-UV chromatogram at 220 nm of **3la**.

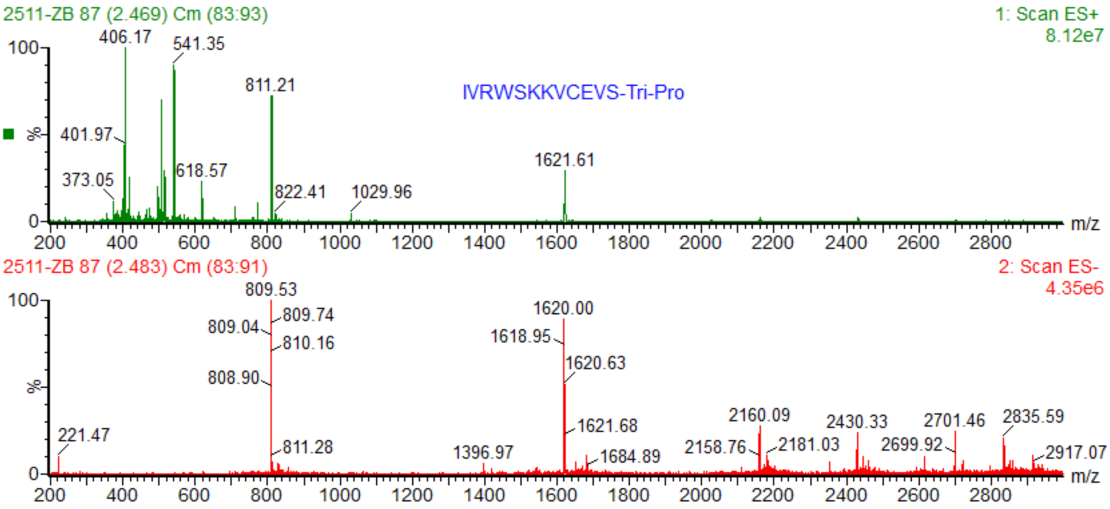

Figure S142. ESI-MS spectrum of **3la**.

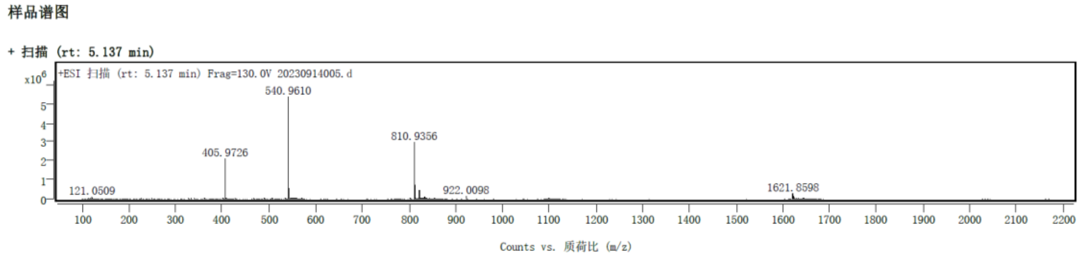

Figure S143. Q-TOF-HRMS spectrum of **3la**.

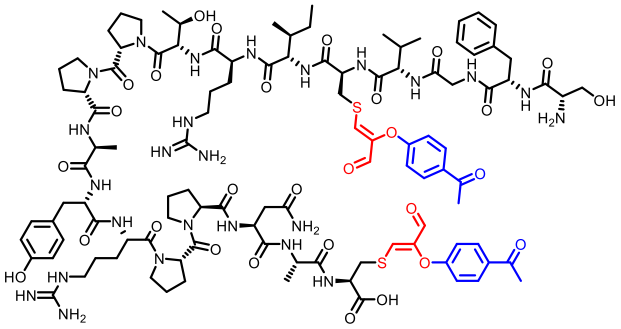

3ma

1m (0.015 mmol, 32.9 mg, 1 equiv.) was dissolved in HEPES buffer (0.2 M, pH = 7.4, 0.9 mL). To this solution **2a** (0.033 mmol, 7.1 mg, 2.2 equiv.) dissolved in acetonitrile (0.1 mL) was added. The reaction incubated for 1 h at room temperature. Then the solvent was purification by preparative RP-HPLC and to give the white solid **3ma** (30.1 mg, 86.3% yield) by freeze drying.

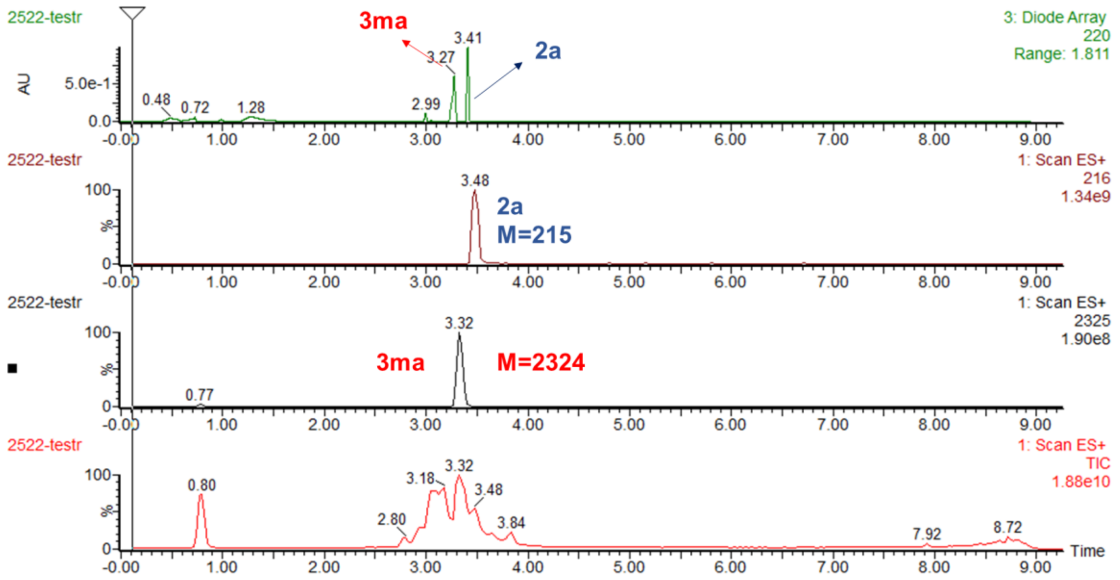

Figure S144. UPLC-MS chromatogram of reaction mixture including TIC and UV curve.

(Extract **2a** and **3ma** mass chromatograms from full scan data).

Analytical **HPLC** using Method D, RT = 11.558 min, the HPLC purity is 97.10%. **LRMS** (ESI+) *m/z*: 2225.87 [M + H]$^+$, (ESI-) *m/z*: 2224.40 [M - H]$^-$. **HRMS** (ES+) *m/z*: [M + H]$^+$ calcd for $C_{108}H_{149}N_{25}O_{29}S_2^+$ 2326.0506, found 2326.0533.

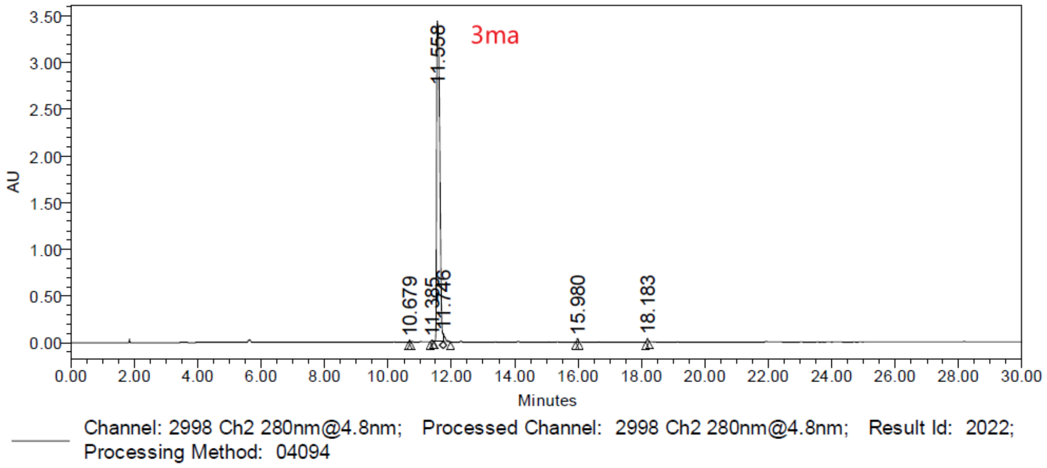

Processed Channel Descr.: 2998 Ch2 280nm@4.8nm

	Processed Channel Descr.	RT	Area	% Area	Height
1	2998 Ch2 280nm@4.8nm	10.679	45151	0.19	18474
2	2998 Ch2 280nm@4.8nm	11.385	35517	0.15	12491
3	2998 Ch2 280nm@4.8nm	11.558	23556309	97.10	3433527
4	2998 Ch2 280nm@4.8nm	11.746	390745	1.61	84180
5	2998 Ch2 280nm@4.8nm	15.980	104333	0.43	35055
6	2998 Ch2 280nm@4.8nm	18.183	127837	0.53	37157

Figure S145. HPLC-UV chromatogram at 280 nm of **3ma**.

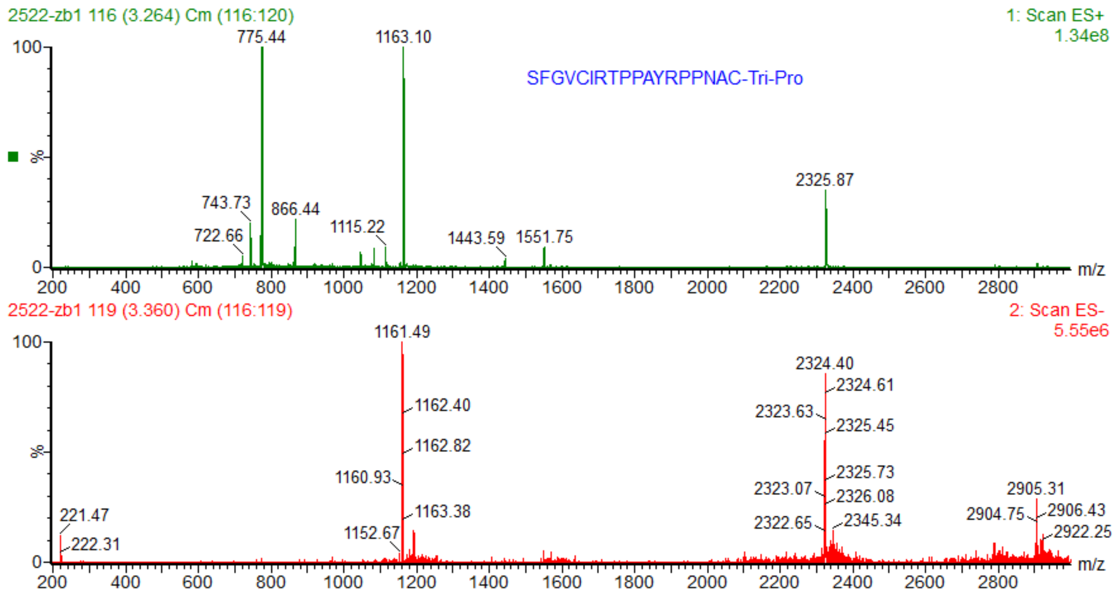

Figure S146. ESI-MS spectrum of **3ma**.

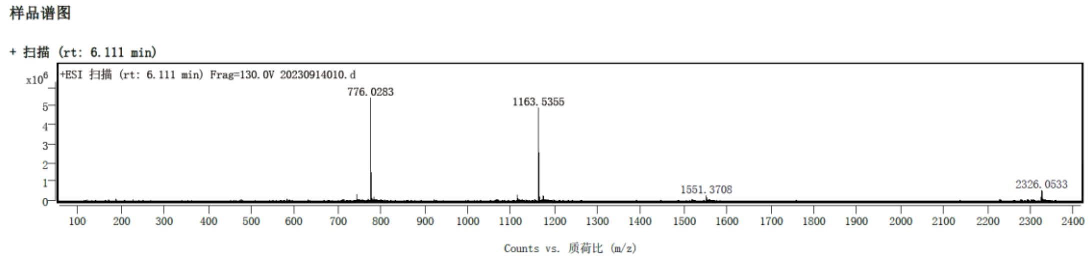

Figure S147. Q-TOF-HRMS spectrum of **3ma**.

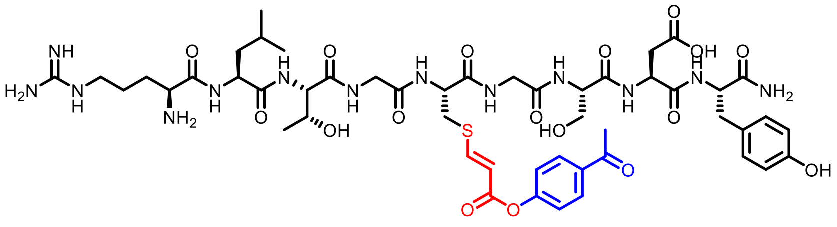

3ra

1r (0.02 mmol, 19.38 mg, 1 equiv.) was dissolved in HEPES buffer (0.2 M, pH = 7.4, 0.9 mL). To this solution **2a** (0.022 mmol, 4.73 mg, 1.1 equiv.) dissolved in acetonitrile (0.1 mL) was added. The reaction incubated for 0.5 h at room temperature and the reaction process was detected by LCMS. Then the solvent was purification by preparative RP-HPLC and to give the white solid **3ra** (15.6 mg, 67.0% yield) by freeze drying.

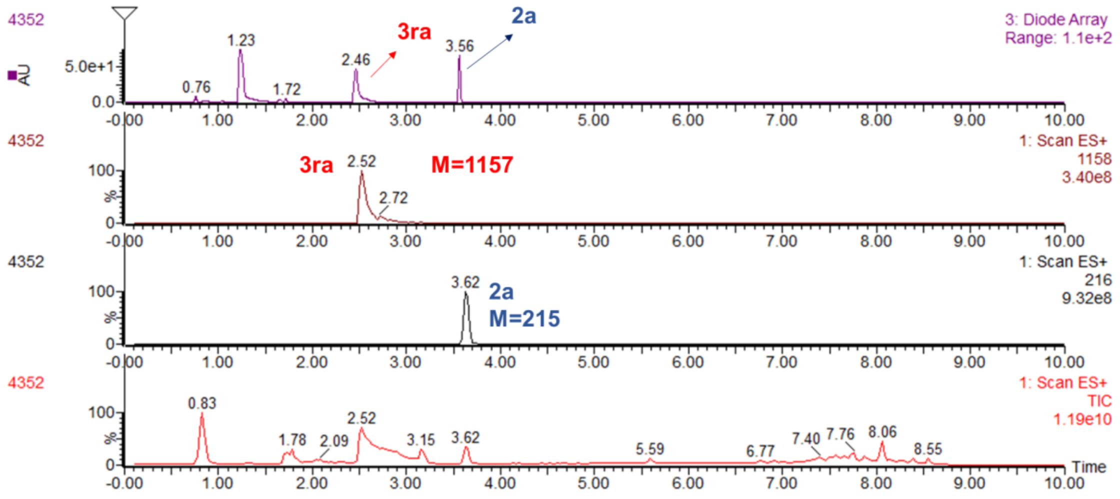

Figure S148. UPLC-MS chromatogram of reaction mixture that reacted for 30 minutes including TIC and UV curve. (Extract **2a** and **3ra** mass chromatograms from full scan data).

Analytical **HPLC** using Method D, RT = 8.403 min, the HPLC purity is 100.0%. **LRMS** (ESI+) *m*/*z*: 1158.60 [M + H]$^+$, (ESI-) *m*/*z*: 1156.78 [M - H]$^-$. **HRMS** (ES+) *m*/*z*: [M + H]$^+$ calcd for $C_{50}H_{71}N_{13}O_{17}S^+$ 1158.4884, found 1158.4876.

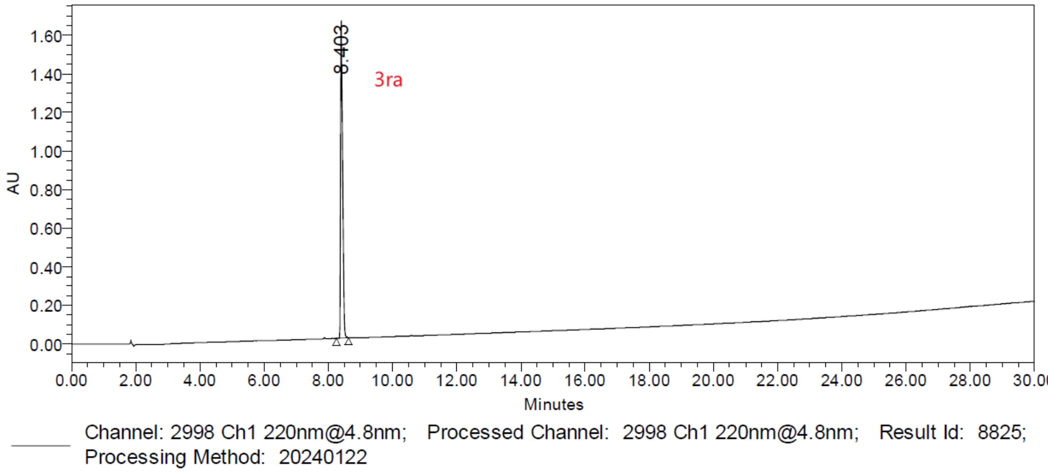

Processed Channel Descr.: 2998 Ch1 220nm@4.8nm

	Processed Channel Descr.	RT	Area	% Area	Height
1	2998 Ch1 220nm@4.8nm	8.403	7328753	100.00	1643780

Figure S149. HPLC-UV chromatogram at 220 nm of **3ra**.

Analysis Report

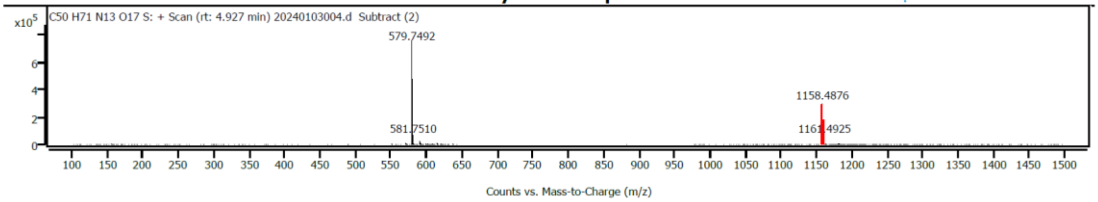

Spectrum Peaks

m/z	z	Abund	Abund %	m/z (Calc)	Diff (ppm)	Ion Species	Formula	Ion Type
1158.4876	1	297195	39.10	1158.4884	-0.69	(M+H)+	C50 H71 N13 O17 S	
1159.4904	1	174424	22.95	1159.4913	-0.76	(M+H)+	C50 H71 N13 O17 S	
1160.4901	1	70766	9.31	1160.4923	-1.89	(M+H)+	C50 H71 N13 O17 S	
1161.4925	1	21752	2.86	1161.4935	-0.84	(M+H)+	C50 H71 N13 O17 S	
579.7492	2	760181	100.00					
580.2510	2	476844	62.73					
580.7511	2	202939	26.70					
581.2517	2	64977	8.55					
581.7510	2	19101	2.51					
590.7390	2	22850	3.01					
591.2395	2	13532	1.78					
591.7353	2	12302	1.62					

Spectrum Identification Table

Best	ID Source	Name	Formula	Species	m/z	Diff (ppm)	CAS	Score	Score (Lib)	Score (DB)	Score (MFG)	Lib/DB
Yes	MFG		C50 H71 N13 O17 S	(M+H)+	1158.4876	-0.90		98.57			98.57	

Figure S150. Q-TOF-HRMS spectrum of **3ra**.

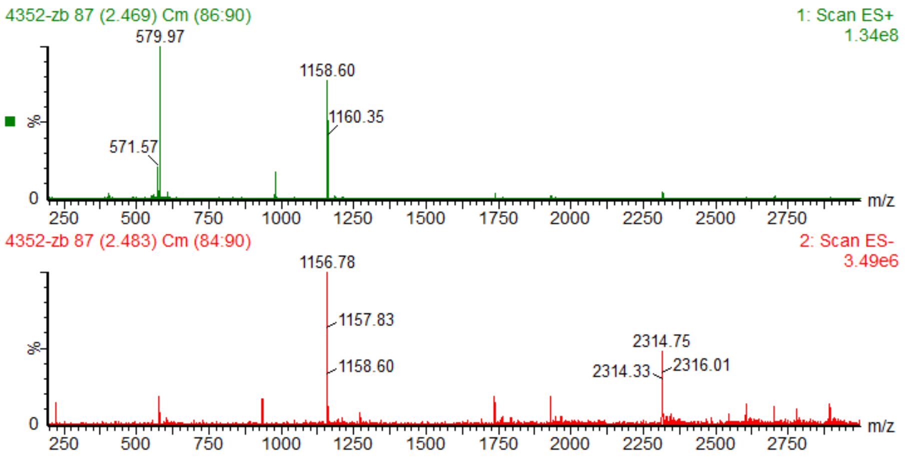

Figure S151. ESI-MS spectrum of **3ra**.

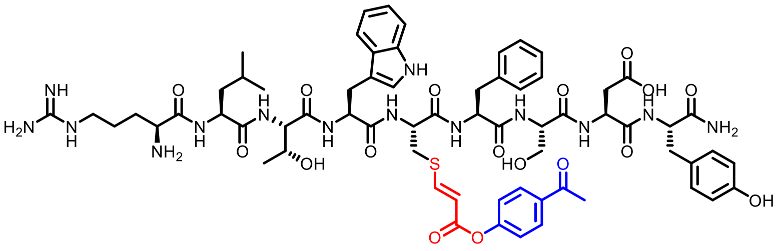

3sa

1s (0.02 mmol, 23.76 mg, 1 equiv.) was dissolved in HEPES buffer (0.2 M, pH = 7.4, 0.9 mL). To this solution **2a** (0.022 mmol, 4.73 mg, 1.1 equiv.) dissolved in acetonitrile (0.1 mL) was added. The reaction incubated at room temperature and the reaction process was detected by LCMS at 0.5 h and 1 h, respectively. Then the solvent was purification by preparative RP-HPLC and to give the white solid **3sa** (17.5 mg, 64.0% yield) by freeze drying.

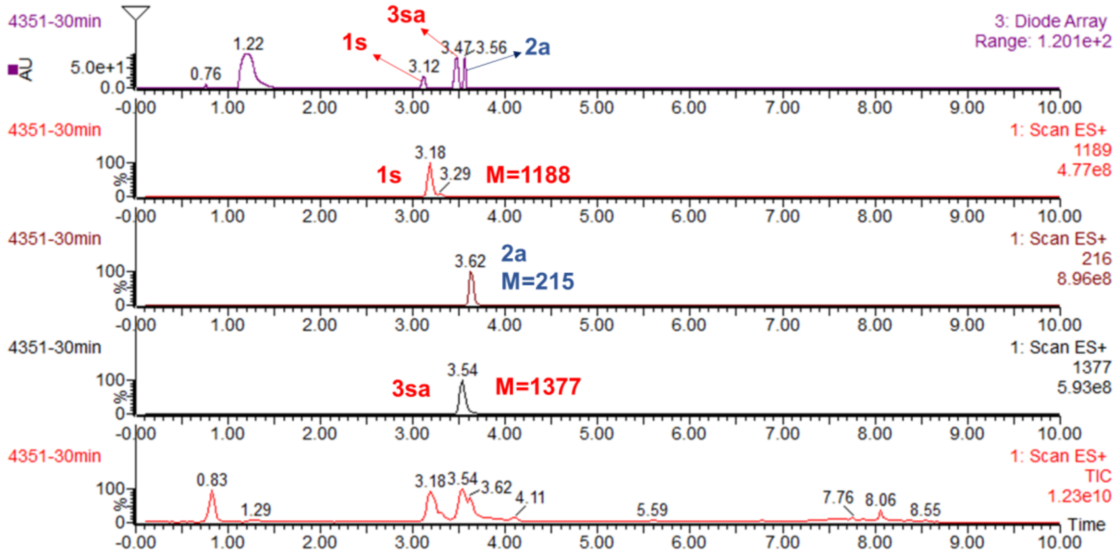

Figure S152. UPLC-MS chromatogram of reaction mixture that reacted for 30 minutes including TIC and UV curve.

(Extract **1s, 2a** and **3sa** mass chromatograms from full scan data).

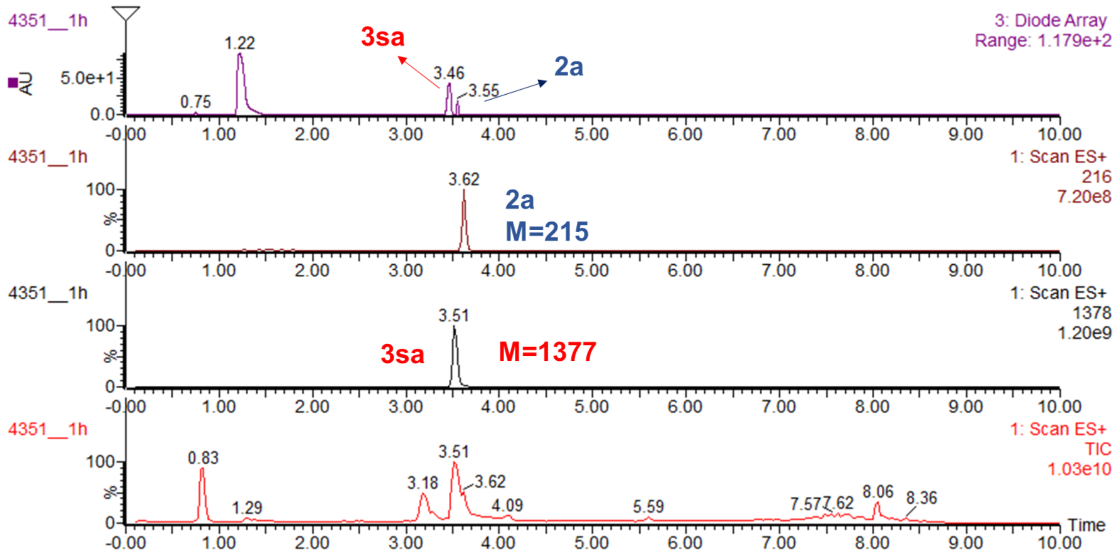

Figure S153. UPLC-MS chromatogram of reaction mixture that reacted for 1 hour including TIC and UV curve.

(Extract **2a** and **3sa** mass chromatograms from full scan data).

Analytical **HPLC** using Method D, RT = 11.749 min, the HPLC purity is 92.99%. **LRMS** (ESI+) *m/z*: 1377.85 $[M + H]^+$, (ESI-) *m/z*: 1375.89 $[M - H]^-$. **HRMS** (ES+) *m/z*: $[M + H]^+$ calcd for $C_{66}H_{84}N_{14}O_{17}S^+$ 1377.5932, found 1377.5924.

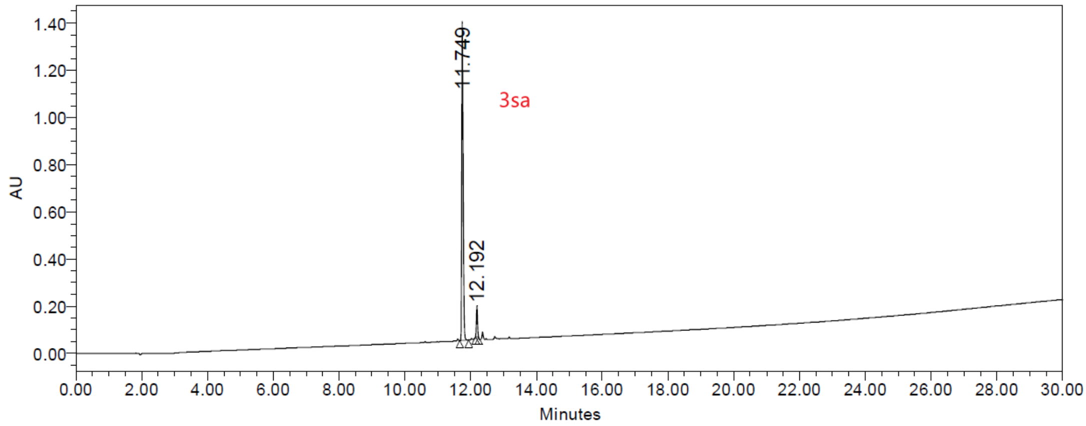

Channel: 2998 Ch1 220nm@4.8nm; Processed Channel: 2998 Ch1 220nm@4.8nm; Result Id: 8827; Processing Method: 20240122

Processed Channel Descr.: 2998 Ch1 220nm@4.8nm

	Processed Channel Descr.	RT	Area	% Area	Height
1	2998 Ch1 220nm@4.8nm	11.749	3997288	92.99	1351565
2	2998 Ch1 220nm@4.8nm	12.192	301104	7.01	131234

Figure S154. HPLC-UV chromatogram at 220 nm of **3sa**.

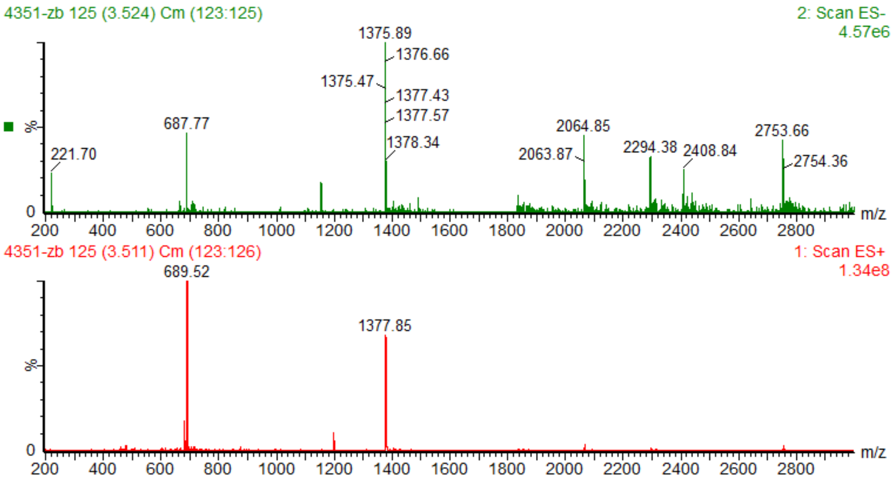

Figure S155. ESI-MS spectrum of **3sa**.

Sample Spectra

+ Scan (rt: 6.224 min) Sub (2) **C66 H84 N14 O17 S**

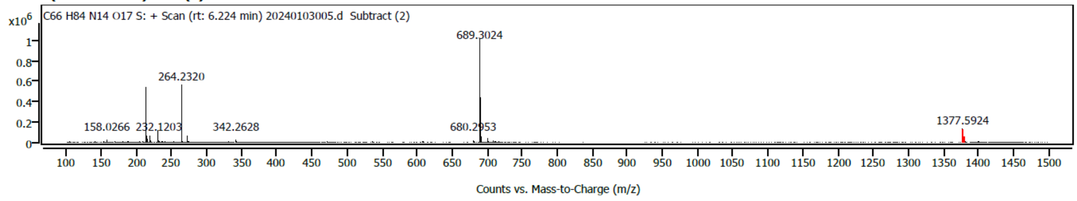

Spectrum Identification Table

Best	ID Source	Name	Formula	Species	m/z	Diff (ppm)	CAS	Score	Score (Lib)	Score (DB)	Score (MFG)	Lib/DB
Yes	MFG		C66 H84 N14 O17 S	(M+H)+	1377.5924	-1.22		97.61			97.61	

Figure S156. Q-TOF-HRMS spectrum of **3sa**.

7 Modification of peptide with functionalized 1,2,3-Triazines

General procedure B:

The dried peptide (1 equiv.) was dissolved in appropriate amount of HEPES buffer (0.2 M, pH = 7.4) to reach a concentration of 27.78 mM. To this solution 1,2,3-triazines (1.2 equiv.) dissolved in acetonitrile or DMF (1/9 of the volume of buffer) was added. The final concentrations in the reaction were 25 mM peptide, 30 mM 1,2,3-triazines and 10% acetonitrile (unless otherwise noted). The reaction incubated for 1-2 h at room temperature and the reaction process was detected by LCMS. The solvent was purification by preparative RP-HPLC and to give the product by freeze drying.

Here, **1b** reacted with **2b** to obtain **4bb** as a representative example to illustrate the general procedure.

1b (0.05 mmol, 26.0 mg, 1 equiv.) was dissolved in HEPES buffer (0.2 M, pH = 7.4, 1.8 mL) to reach a concentration of 27.78 mM. To this solution **2b** (0.06 mmol, 12.2 mg, 1.2 equiv.) dissolved in acetonitrile (0.2 mL) was added. The final concentrations in the reaction were 25 mM **1b**, 30 mM **2b** and 10% acetonitrile. The reaction incubated for 2 h at room temperature and the reaction process was detected by LCMS. The solvent was purification by preparative RP-HPLC and to give the white solid **4bb** (23.3 mg, 67.1% yield) by freeze drying.

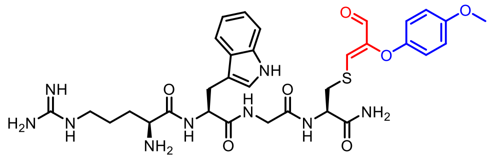

4bb

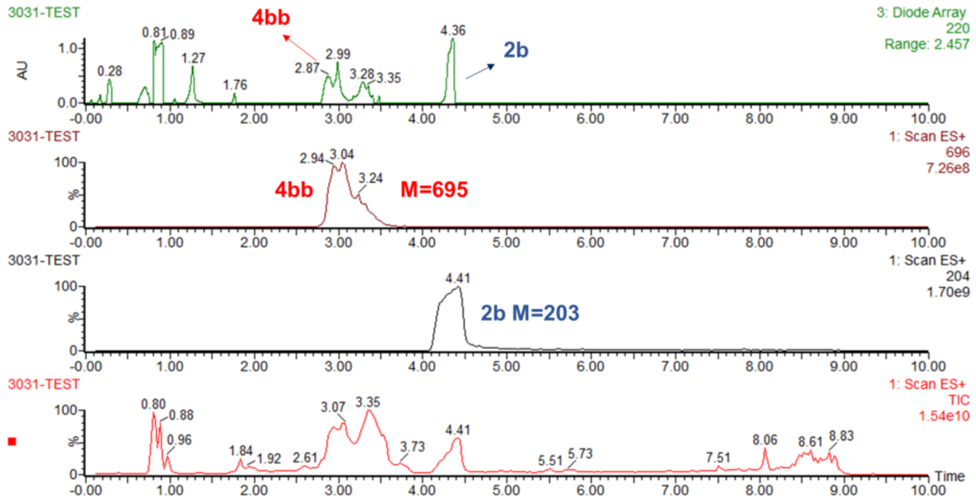

Figure S157. UPLC-MS chromatogram of reaction mixture including TIC and UV curve.

(Extract **2b** and **4bb** mass chromatograms from full scan data).

Analytical **HPLC** using Method D, RT = 9.934 min, the HPLC purity is 97.20%. **LRMS** (ESI+) *m/z*: 696.20 $[M + H]^+$, (ESI-) *m/z*: 694.80 $[M - H]^-$. **HRMS** (ES+) *m/z*: $[M + H]^+$ calcd for $C_{32}H_{41}N_9O_7S^+$ 696.2928, found 696.2935.

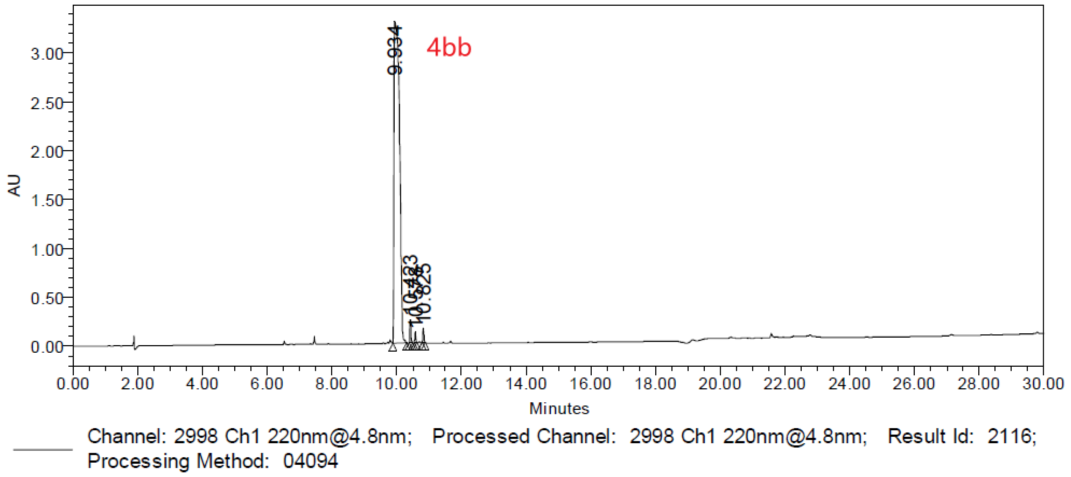

Processed Channel Descr.: 2998 Ch1 220nm@4.8nm

	Processed Channel Descr.	RT	Area	% Area	Height
1	2998 Ch1 220nm@4.8nm	9.934	39215288	97.20	3295729
2	2998 Ch1 220nm@4.8nm	10.423	562420	1.39	238723
3	2998 Ch1 220nm@4.8nm	10.578	234657	0.58	106886
4	2998 Ch1 220nm@4.8nm	10.825	331888	0.82	146852

Figure S158. HPLC-UV chromatogram at 220 nm of **4bb**.

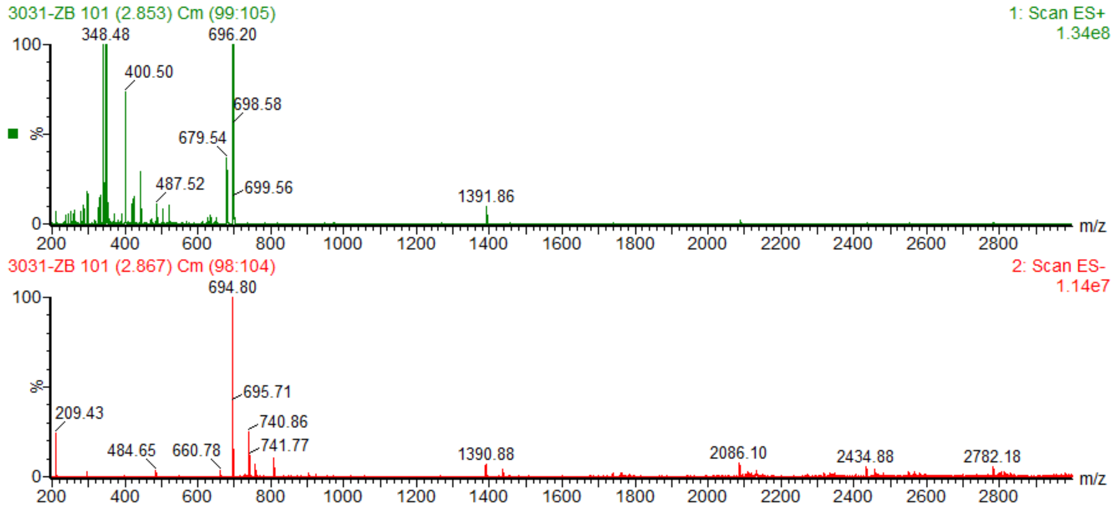

Figure S159. ESI-MS spectrum of **4bb**.

Analysis Report

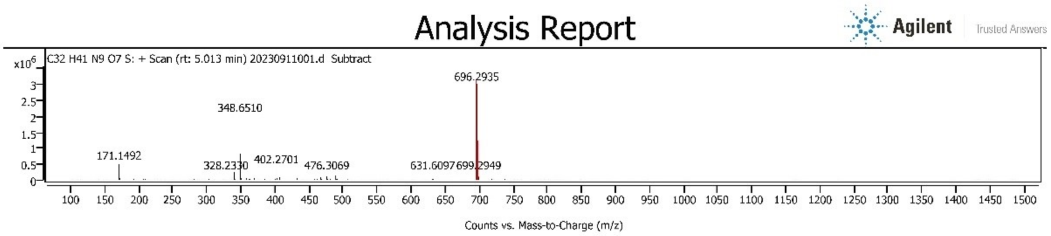

Spectrum Peaks

m/z	Z	Abund	Abund %	m/z (Calc)	Diff (ppm)	Ion Species	Formula	Ion Type
696.2935	1	3031086	100.00	696.2922	1.86	(M+H)+	C32 H41 N9 O7 S	
697.2960	1	1282411	42.31	697.2950	1.41	(M+H)+	C32 H41 N9 O7 S	
698.2951	1	451363	14.89	698.2944	0.98	(M+H)+	C32 H41 N9 O7 S	
699.2949	1	105089	3.47	699.2952	-0.35	(M+H)+	C32 H41 N9 O7 S	
171.1492	1	514668	16.98					
172.1517	1	54251	1.79					
187.1263		114814	3.79					
193.1309		36559	1.21					
328.2330		57929	1.91					
333.1883		31660	1.04					
339.6451	2	353180	11.65					
340.1426	2	243335	8.03					
340.6428	2	88543	2.92					
348.6510	2	2006795	66.21					
349.1525	2	825655	27.24					
349.3191		34220	1.13					
349.6517	2	286888	9.46					
350.1520	2	72124	2.38					
358.2434		64120	2.12					
369.1634	2	142884	4.71					
369.6647	2	59218	1.95					
375.6098	2	36802	1.21					
385.2424		36999	1.22					
402.2701	1	389510	12.85					
403.2729	1	71300	2.35					
407.2250		93936	3.10					
432.2798		66426	2.19					
458.5291		36847	1.22					
462.2901		35319	1.17					
467.3223		87555	2.89					
476.3069	1	113257	3.74					
477.3091	1	33202	1.10					
489.3044	1	150957	4.98					
490.3065	1	43959	1.45					
491.3181	1	46752	1.54					
507.3149		37792	1.25					
631.6097		43814	1.45					
631.9426		44070	1.45					
718.2740		41420	1.37					
737.3184		36609	1.21					

Spectrum Identification Table

Best	ID Source	Name	Formula	Species	m/z	Diff (ppm)	CAS	Score	Score (Lib)	Score (DB)	Score (MFG)	Lib/DB
Yes	MFG		C32 H41 N9 O7 S	(M+H)+	696.2935	1.60		97.39			97.39	

Figure S160. Q-TOF-HRMS spectrum of **4bb**.

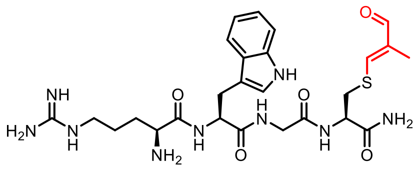

4bc

According to the general procedure B, **1b** (0.05 mmol, 26 mg) reacted with **2c** (0.06 mmol, 5.7 mg) to afford the white solid **4bc** (24.9 mg) in 84.4% isolated yield.

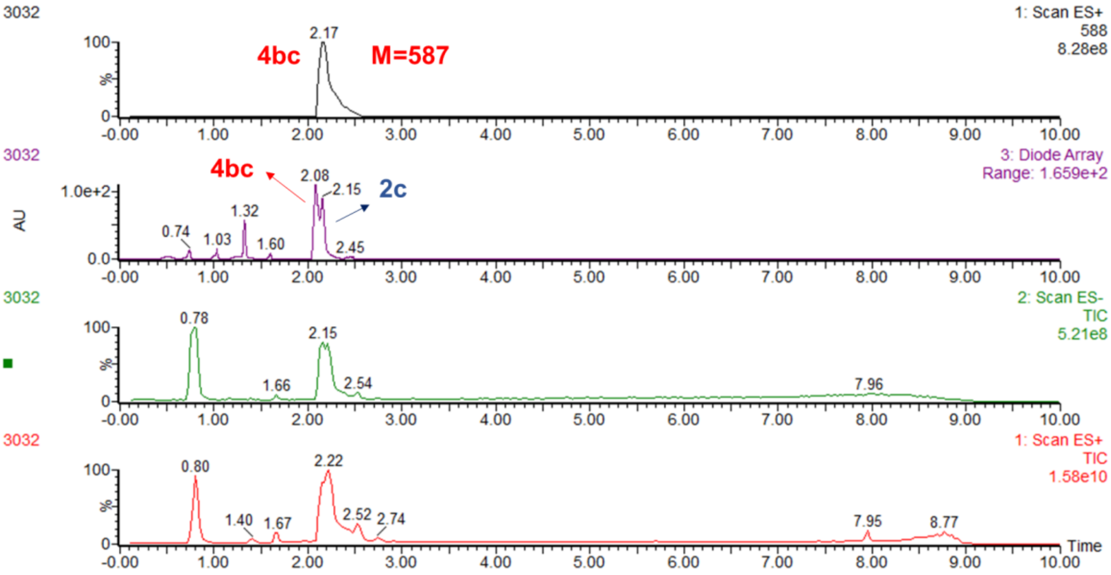

Figure S161. UPLC-MS chromatogram of reaction mixture including TIC and UV curve.

(Extract **4bc** mass chromatogram from full scan data).

Analytical **HPLC** using Method D, RT = 7.768 min, the HPLC purity is 100%. **LRMS** (ESI+) *m*/*z*: 588.54 [M + H]$^+$, (ESI-) *m*/*z*: 586.65 [M - H]$^-$. **HRMS** (ES+) *m*/*z*: [M + H]$^+$ calcd for $C_{26}H_{37}N_9O_5S^+$ 588.2716, found 588.2713.

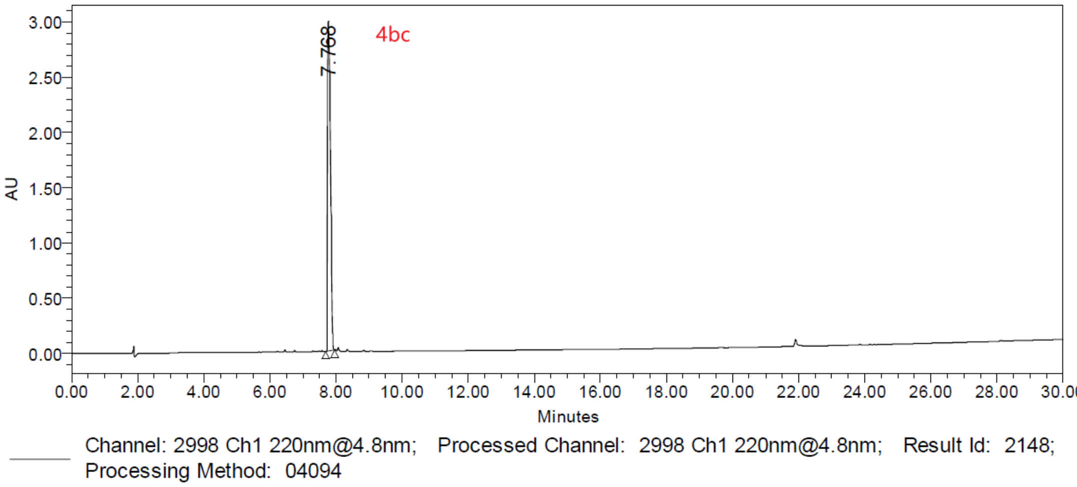

Processed Channel Descr.: 2998 Ch1 220nm@4.8nm

	Processed Channel Descr.	RT	Area	% Area	Height
1	2998 Ch1 220nm@4.8nm	7.768	18491743	100.00	2983684

Figure S162. HPLC-UV chromatogram at 220 nm of **4bc**.

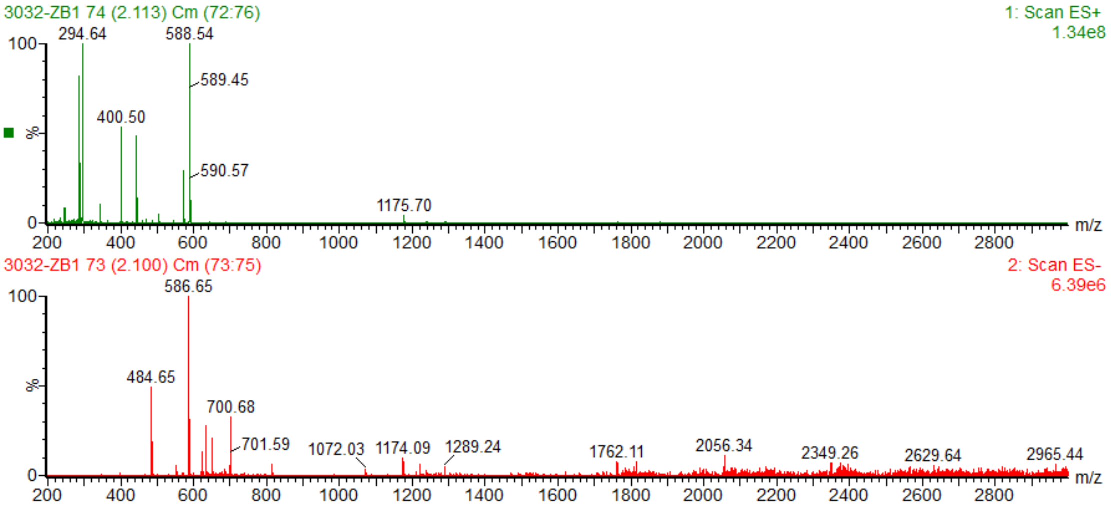

Figure S163. ESI-MS spectrum of **4bc**.

Analysis Report

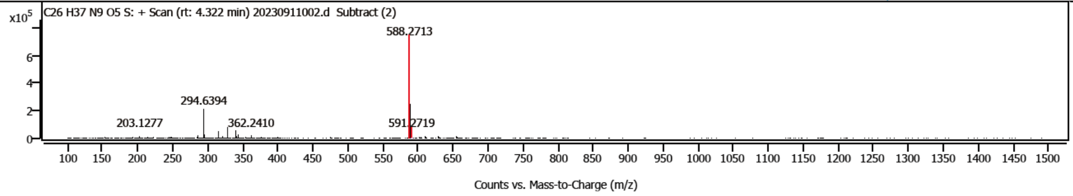

Spectrum Peaks

m/z	Z	Abund	Abund %	m/z (Calc)	Diff (ppm)	Ion Species	Formula	Ion Type
588.2713	1	741430	100.00	588.2711	0.30	(M+H)+	C26 H37 N9 O5 S	
589.2734	1	244020	32.91	589.2738	-0.62	(M+H)+	C26 H37 N9 O5 S	
590.2716	1	75986	10.25	590.2724	-1.30	(M+H)+	C26 H37 N9 O5 S	
591.2719	1	15034	2.03	591.2731	-1.94	(M+H)+	C26 H37 N9 O5 S	
203.1277		11220	1.51					
249.1566		8377	1.13					
286.1268		16782	2.26					
294.6394	2	209477	28.25					
295.1405	2	65088	8.78					
295.6401	2	22201	2.99					
315.1524	2	51235	6.91					
315.6536	2	18998	2.56					
321.6000	2	11418	1.54					
328.1605	2	77331	10.43					
328.6622	2	27478	3.71					
329.1622	2	10081	1.36					
340.2587	1	53544	7.22					
341.2623	1	10225	1.38					
344.2277		24416	3.29					
362.2410		21061	2.84					
610.2527		14971	2.02					
629.2958		11530	1.56					
655.3110		14365	1.94					

Spectrum Identification Table

Best	ID Source	Name	Formula	Species	m/z	Diff (ppm)	CAS	Score	Score (Lib)	Score (DB)	Score (MFG)	Lib/DB
Yes	MFG		C26 H37 N9 O5 S	(M+H)+	588.2713	-0.05		99.41			99.41	

Figure S164. Q-TOF-HRMS spectrum of **4bc**.

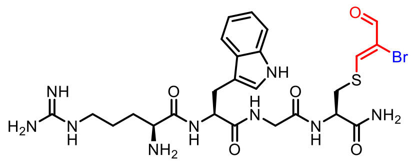

4bd

According to the general procedure B, **1b** (0.087 mmol, 45.2 mg) reacted with **2d** (0.1 mmol, 16 mg) to afford the white solid **4bd** (31.4 mg) in 55.4% isolated yield.

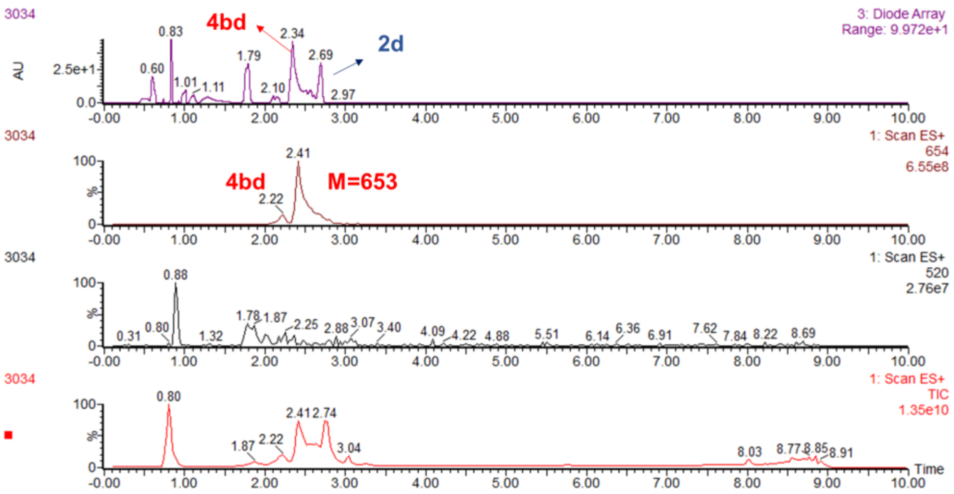

Figure S165. UPLC-MS chromatogram of reaction mixture including TIC and UV curve.

(Extract **2d** and **4bd** mass chromatograms from full scan data).

Analytical **HPLC** using Method D, RT = 8.313 min, the HPLC purity is 95.37%. **LRMS** (ESI+) *m/z*: 654.21 [M + H]$^+$, (ESI-) *m/z*: 652.58 [M - H]$^-$. **HRMS** (ES+) *m/z*: [M + H]$^+$ calcd for $C_{25}H_{34}BrN_9O_5S^+$ 652.1665, found 652.1661.

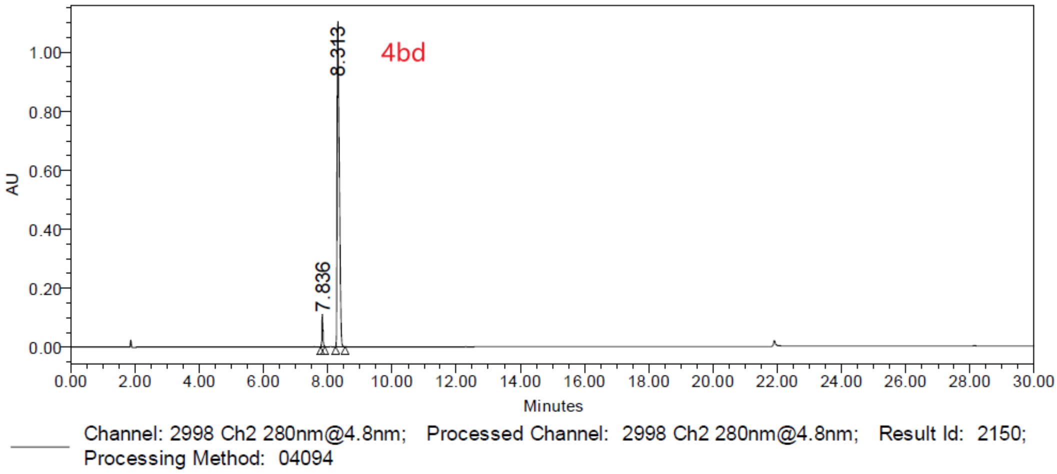

Processed Channel Descr.: 2998 Ch2 280nm@4.8nm

	Processed Channel Descr.	RT	Area	% Area	Height
1	2998 Ch2 280nm@4.8nm	7.836	256141	4.63	108504
2	2998 Ch2 280nm@4.8nm	8.313	5272761	95.37	1102296

Figure S166. HPLC-UV chromatogram at 280 nm of **4bd**.

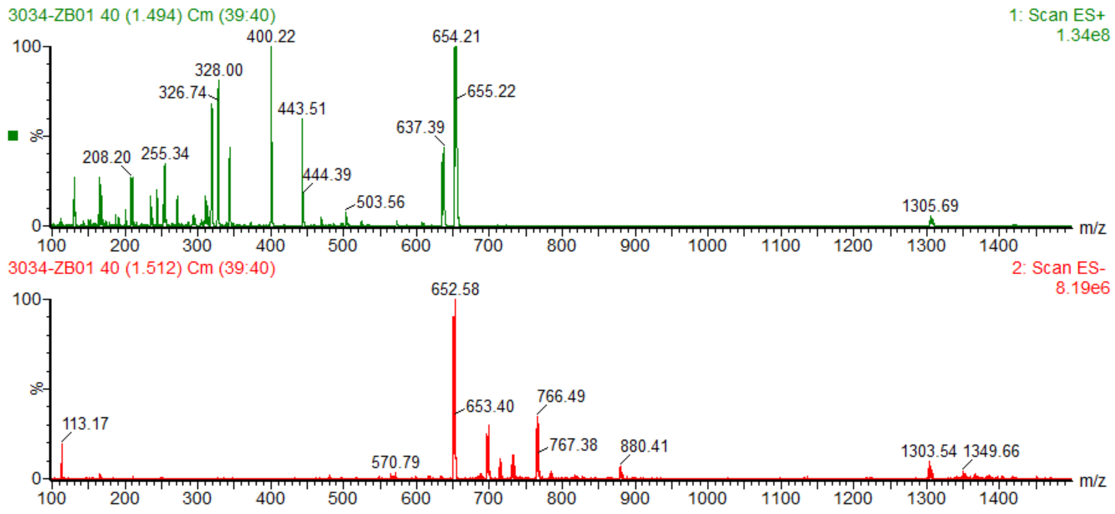

Figure S167. ESI-MS spectrum of **4bd**.

Analysis Report

Agilent Trusted Answers

+ Scan (rt: 4.280 min) Sub (2) C25 H34 Br N9 O5 S

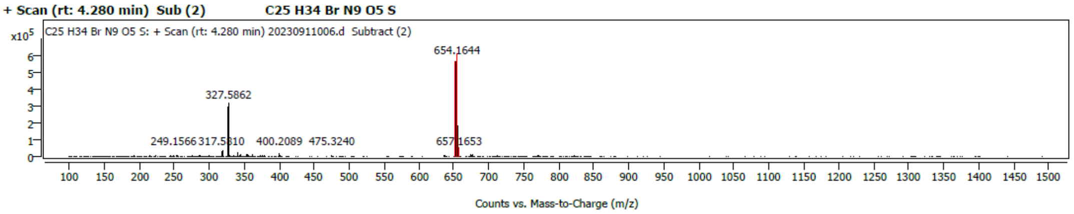

Spectrum Peaks

m/z	Z	Abund	Abund %	m/z (Calc)	Diff (ppm)	Ion Species	Formula	Ion Type
652.1661	1	562214	92.69	652.1660	0.27	(M+H)+	C25 H34 Br N9 O5 S	
653.1680	1	173584	28.62	653.1686	-0.94	(M+H)+	C25 H34 Br N9 O5 S	
654.1644	1	606585	100.00	654.1642	0.33	(M+H)+	C25 H34 Br N9 O5 S	
655.1665	1	182102	30.02	655.1667	-0.27	(M+H)+	C25 H34 Br N9 O5 S	
656.1642	1	53849	8.88	656.1651	-1.40	(M+H)+	C25 H34 Br N9 O5 S	
657.1653	1	10428	1.72	657.1659	-0.80	(M+H)+	C25 H34 Br N9 O5 S	
249.1566		7041	1.16					
317.5810	2	16367	2.70					
318.0758		28621	4.72					
318.5789	2	26096	4.30					
319.0742	2	33967	5.60					
319.5751	2	10845	1.79					
326.5870	2	294485	48.55					
327.0882	2	96310	15.88					
327.5862	2	318707	52.54					
328.0872	2	105423	17.38					
328.5865	2	31058	5.12					
329.0888	2	6122	1.01					
340.2589		23138	3.81					
343.1873		6427	1.06					
344.2268		9612	1.58					
353.5467	2	10562	1.74					
354.2384		6165	1.02					
354.5449	2	11635	1.92					
362.2406		13653	2.25					
376.2202		7399	1.22					
400.2089		17299	2.85					
475.3240		8586	1.42					
674.1470		11142	1.84					
676.1447		13805	2.28					

Spectrum Identification Table

Best	ID Source	Name	Formula	Species	m/z	Diff (ppm)	CAS	Score	Score (Lib)	Score (DB)	Score (MFG)	Lib/DB
Yes	MFG		C25 H34 Br N9 O5 S	(M+H)+	652.1661	0.03		99.51			99.51	

Figure S168. Q-TOF-HRMS spectrum of **4bd**.

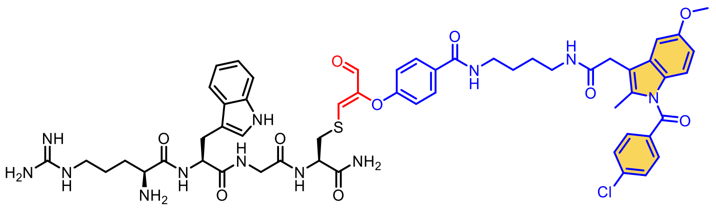

4be

According to the general procedure B, **1b** (0.05 mmol, 26 mg) reacted with **2e** (0.06 mmol, 37 mg, dissolved in DMF) to afford the white solid **4be** (25.3 mg) in 45.3% isolated yield.

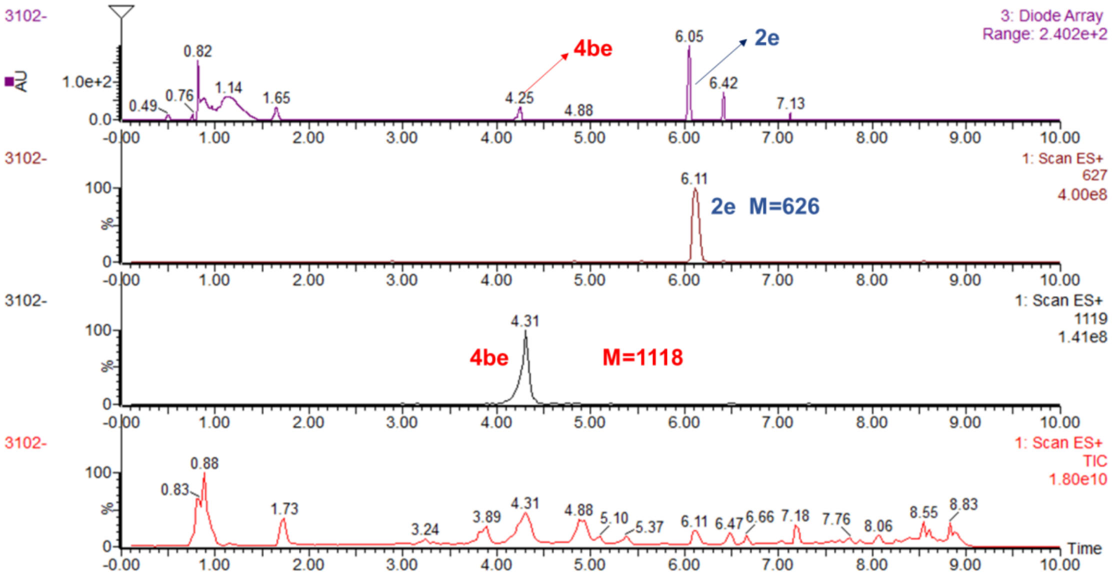

Figure S169. UPLC-MS chromatogram of reaction mixture including TIC and UV curve.

(Extract **2e** and **4be** mass chromatograms from full scan data).

Analytical **HPLC** using Method D, RT = 15.020 min, the HPLC purity is 94.16%. **LRMS** (ESI+) *m/z*: 1119.70 $[M + H]^+$, (ESI-) *m/z*: 1117.81 $[M - H]^-$. **HRMS** (ES+) *m/z*: $[M + H]^+$ calcd for $C_{55}H_{63}ClN_{12}O_{10}S^+$ 1119.4278, found 1119.4273.

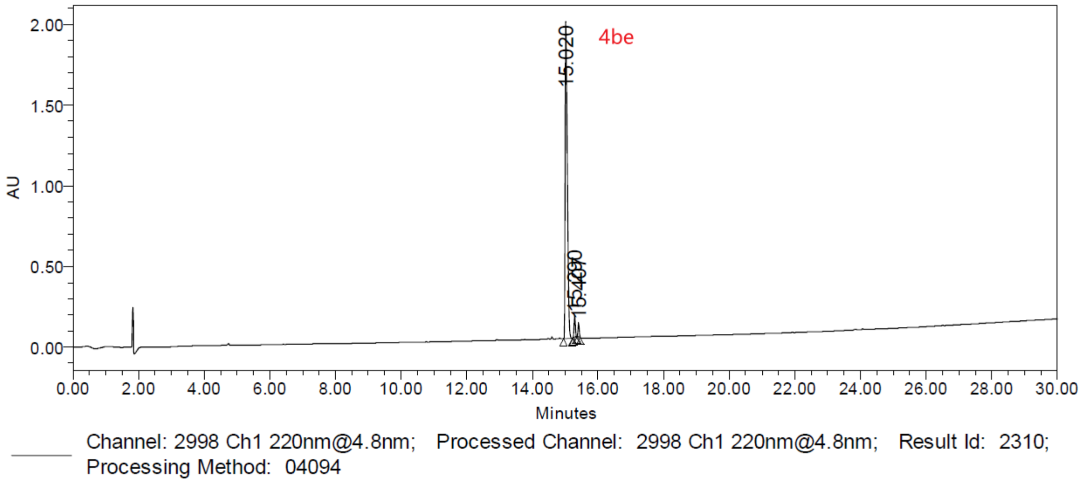

Processed Channel Descr.: 2998 Ch1 220nm@4.8nm

	Processed Channel Descr.	RT	Area	% Area	Height
1	2998 Ch1 220nm@4.8nm	15.020	9103534	94.16	1963861
2	2998 Ch1 220nm@4.8nm	15.290	353315	3.65	133386
3	2998 Ch1 220nm@4.8nm	15.407	210964	2.18	86011

Figure S170. HPLC-UV chromatogram at 220 nm of **4be**.

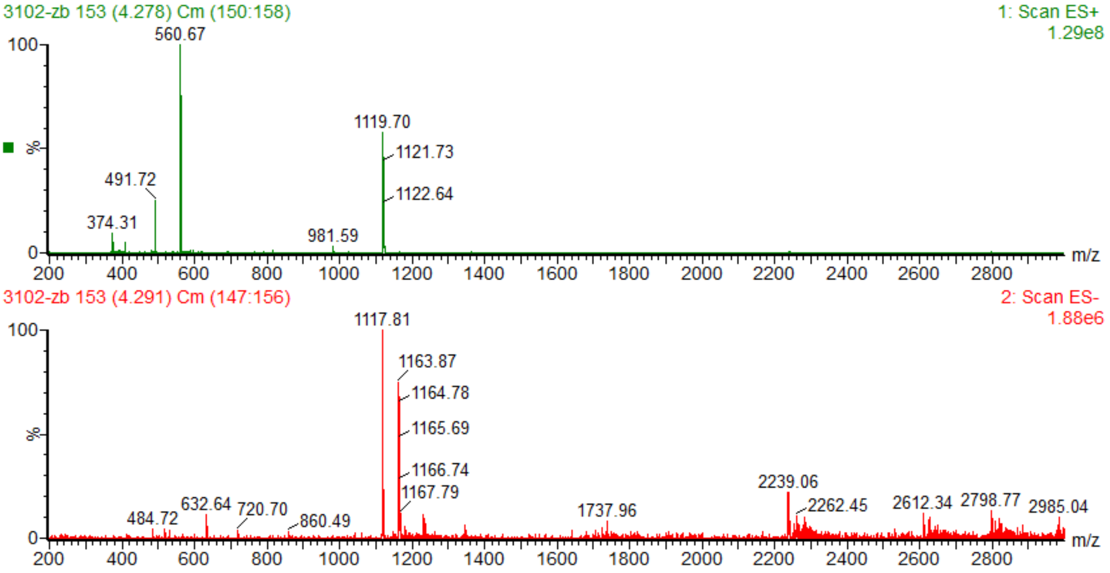

Figure S171. ESI-MS spectrum of **4be**.

Analysis Report

+ Scan (rt: 6.921 min) Sub (2) C55 H63 Cl N12 O10 S

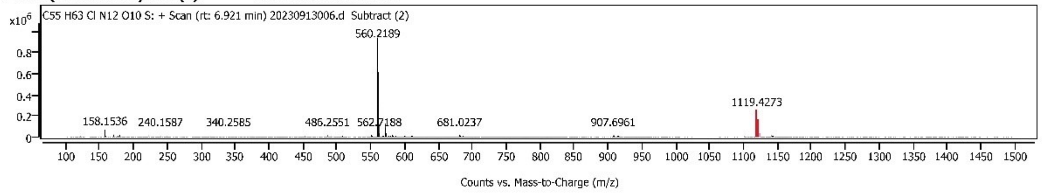

Spectrum Peaks

m/z	Z	Abund	Abund %	m/z (Calc)	Diff (ppm)	Ion Species	Formula	Ion Type
1119.4273	1	253747	27.03	1119.4272	0.09	(M+H)+	C55 H63 Cl N12 O10 S	
1120.4296	1	164715	17.54	1120.4301	-0.47	(M+H)+	C55 H63 Cl N12 O10 S	
1121.4275	1	150071	15.98	1121.4275	-0.06	(M+H)+	C55 H63 Cl N12 O10 S	
1122.4274	1	71844	7.65	1122.4287	-1.22	(M+H)+	C55 H63 Cl N12 O10 S	
158.1536		72216	7.69					
171.1477		23196	2.47					
180.1356		16572	1.77					
223.0954		16995	1.81					
240.1587		10008	1.07					
340.2585		11828	1.26					
362.2402		15645	1.67					
486.2551		17051	1.82					
508.2387		9934	1.06					
551.2123	2	16042	1.71					
551.7139	2	10295	1.10					
560.2189	2	938924	100.00					
560.7202	2	661406	70.44					
561.2188	2	611692	65.15					
561.7191	2	310919	33.11					
562.2189	2	116959	12.46					
562.7188	2	36851	3.92					
567.2142		11805	1.26					
568.2164		13792	1.47					
571.2088	2	126619	13.49					
571.7100	2	83900	8.94					
572.2091	2	73080	7.78					
572.7099	2	36355	3.87					
573.2099		15639	1.67					
576.2258	2	14122	1.50					
576.7268	2	9762	1.04					
582.2011	2	21331	2.27					
582.7021	2	15355	1.64					
583.2009	2	13744	1.46					
681.0237		10042	1.07					
681.2742	2	16867	1.80					
681.5253	2	21565	2.30					
681.7752	2	21906	2.33					
682.0254	2	15816	1.68					
682.2752	2	12686	1.35					
685.7761	2	10500	1.12					
686.0279	2	12986	1.38					
686.2758	2	12197	1.30					
686.5276	2	9573	1.02					
907.6961		10419	1.11					
908.0312	1	15623	1.66					
908.3629	1	22541	2.40					
908.6982		20620	2.20					
909.0314	1	15503	1.65					
909.3652	1	10865	1.16					
914.3634	1	10574	1.13					
915.3554	1	10269	1.09					
1123.4268		26938	2.87					
1141.4089	1	18464	1.97					
1142.4109	1	11389	1.21					
1143.4072	1	11201	1.19					

Spectrum Identification Table

Best ID Source	Name	Formula	Species	m/z	Diff (ppm)	CAS	Score	Score (Lib)	Score (DB)	Score (MFG)	Lib/DB
Yes MFG		C55 H63 Cl N12 O10 S	(M+H)+	1119.4273	-0.24		91.50			91.50	

Figure S172. Q-TOF-HRMS spectrum of **4be**.

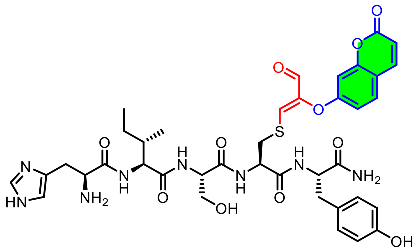

4cf

According to the general procedure B, **1c** (0.07 mmol, 43.4 mg) reacted with **2f** (0.084 mmol, 20.2 mg, dissolved in DMF) to afford the white solid **4cf** (46.1 mg) in 78.9% isolated yield.

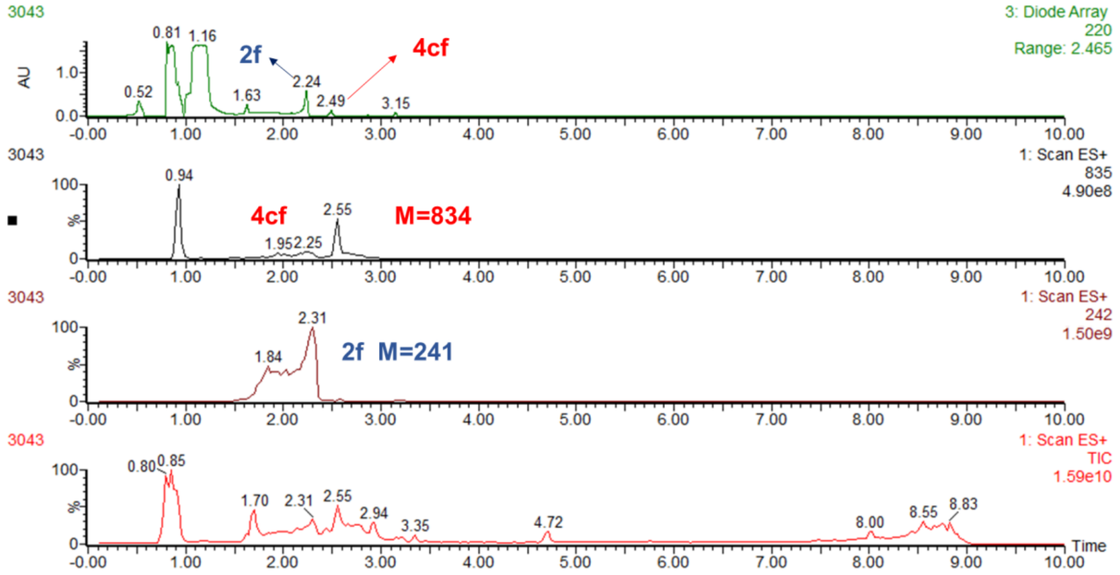

Figure S173. UPLC-MS chromatogram of reaction mixture including TIC and UV curve.

(Extract **2f** and **4cf** mass chromatograms from full scan data).

Analytical **HPLC** using Method D, RT = 9.068 min, the HPLC purity is 100%. **LRMS** (ESI+) *m*/*z*: 835.57 [M + H]$^+$, (ESI-) *m*/*z*: 833.75 [M - H]$^-$. **HRMS** (ES+) *m*/*z*: [M + H]$^+$ calcd for $C_{39}H_{46}N_8O_{11}S^+$ 835.3084, found 835.3081.

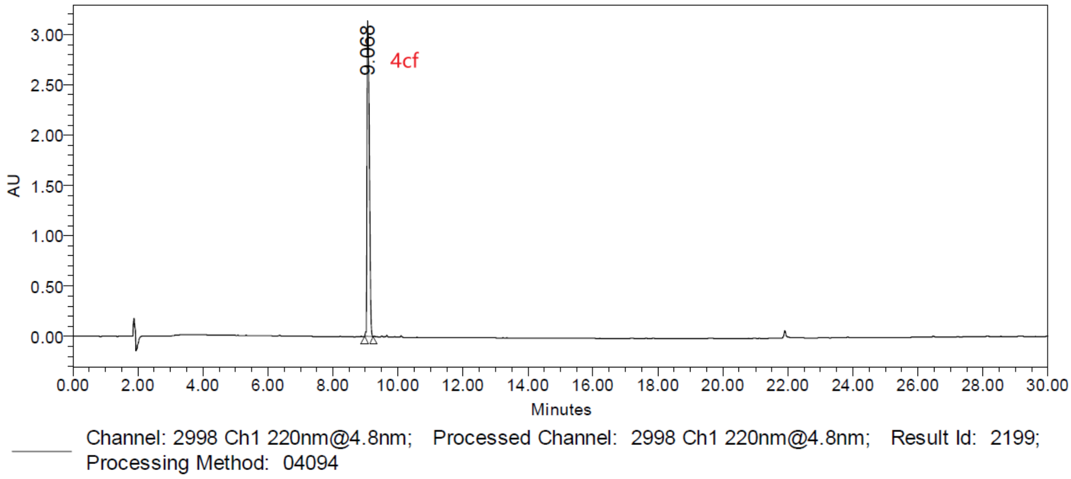

Processed Channel Descr.: 2998 Ch1 220nm@4.8nm

	Processed Channel Descr.	RT	Area	% Area	Height
1	2998 Ch1 220nm@4.8nm	9.068	15849804	100.00	3130449

Figure S174. HPLC-UV chromatogram at 220 nm of **4cf**.

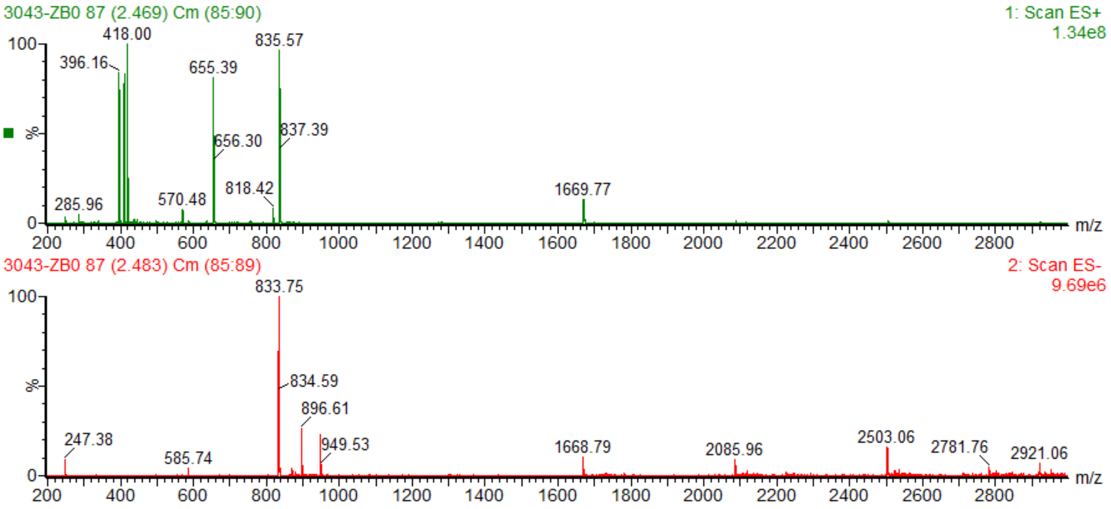

Figure S175. ESI-MS spectrum of **4cf**.

Analysis Report

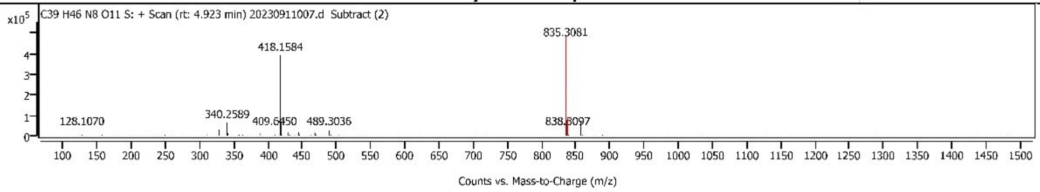

Spectrum Peaks

m/z	Z	Abund	Abund %	m/z (Calc)	Diff (ppm)	Ion Species	Formula	Ion Type
835.3081	1	485309	100.00	835.3080	0.14	(M+H)+	C39 H46 N8 O11 S	
836.3106	1	223228	46.00	836.3109	-0.39	(M+H)+	C39 H46 N8 O11 S	
837.3104	1	78243	16.12	837.3110	-0.74	(M+H)+	C39 H46 N8 O11 S	
838.3097	1	21166	4.36	838.3120	-2.71	(M+H)+	C39 H46 N8 O11 S	
128.1070		4936	1.02					
157.1312		4866	1.00					
311.2071		8315	1.71					
328.2328	1	31922	6.58					
329.2363	1	5016	1.03					
333.1880		12619	2.60					
340.2589	1	66660	13.74					
341.2615	1	14024	2.89					
358.2428		6147	1.27					
362.2408	1	29697	6.12					
363.2409	1	6393	1.32					
388.2529		13646	2.81					
395.6476		6389	1.32					
409.6450	2	10111	2.08					
410.1479	2	5424	1.12					
418.1584	2	393596	81.10					
418.6595	2	190578	39.27					
419.1599	2	70676	14.56					
419.6611	2	18137	3.74					
429.1495	2	18041	3.72					
429.6500	2	9178	1.89					
444.6152		5804	1.20					
445.1178	2	17885	3.69					
445.6200	2	7970	1.64					
446.1191	2	6066	1.25					
467.3223	1	21324	4.39					
468.3264	1	6071	1.25					
469.3366	1	12383	2.55					
489.3036	1	25478	5.25					
490.3061	1	7808	1.61					
491.3182	1	17876	3.68					
492.3217	1	5622	1.16					
857.2884	1	73051	15.05					
858.2919	1	36321	7.48					
859.2921	1	11459	2.36					
889.2281		4977	1.03					

Spectrum Identification Table

Best	ID Source	Name	Formula	Species	m/z	Diff (ppm)	CAS	Score	Score (Lib)	Score (DB)	Score (MFG)	Lib/DB
Yes	MFG		C39 H46 N8 O11 S	(M+H)+	835.3081	-0.18		99.56			99.56	

Figure S176. Q-TOF-HRMS spectrum of **4cf**.

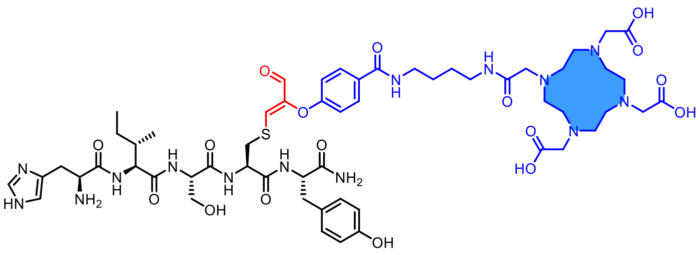

4cg

According to the general procedure B, **1c** (0.06 mmol, 37.2 mg) reacted with **2g** (0.072 mmol, 48.5 mg) to afford the white solid **4cg** (51.2 mg) in 67.4% isolated yield.

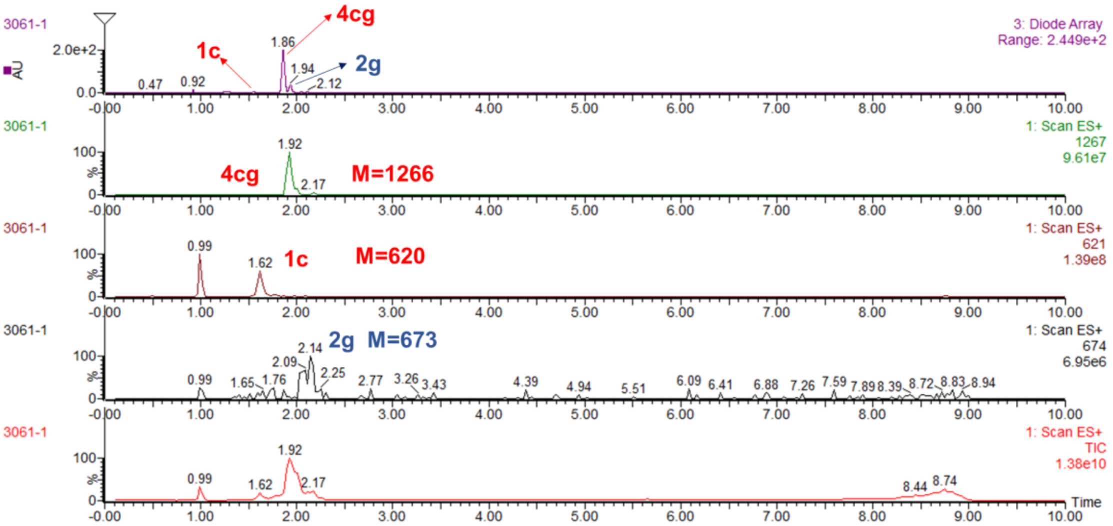

Figure S177. UPLC-MS chromatogram of reaction mixture including TIC and UV curve.

(Extract **1c, 2g** and **4cg** mass chromatograms from full scan data).

Analytical **HPLC** using Method D, RT = 6.858 min, the HPLC purity is 96.47%. **LRMS** (ESI+) *m*/*z*: 1267.82 [M + H]$^+$, (ESI-) *m*/*z*: 1266.00 [M - H]$^-$. **HRMS** (ES+) *m*/*z*: [M + H]$^+$ calcd for $C_{57}H_{82}N_{14}O_{17}S^+$ 1267.5781, found 1267.5802.

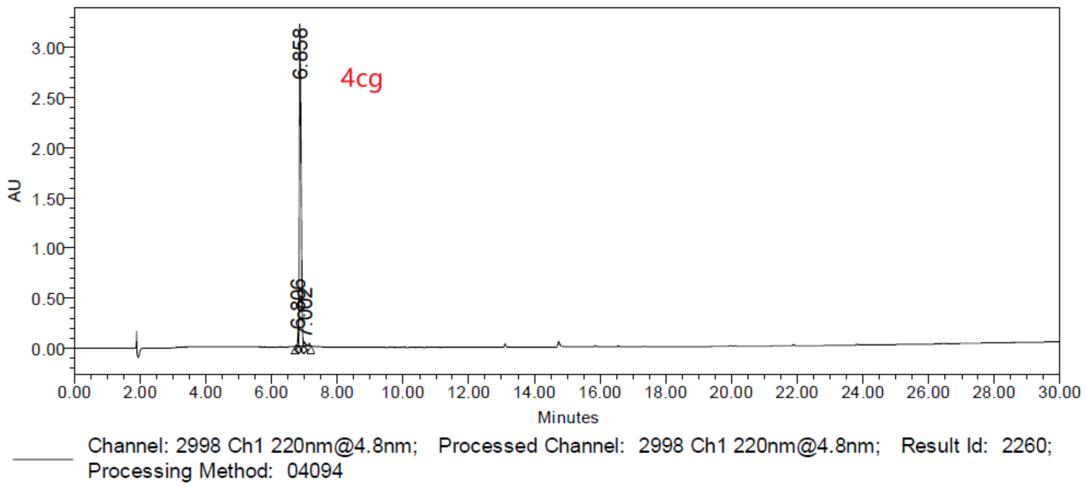

Processed Channel Descr.: 2998 Ch1 220nm@4.8nm

	Processed Channel Descr.	RT	Area	% Area	Height
1	2998 Ch1 220nm@4.8nm	6.806	248791	2.04	128260
2	2998 Ch1 220nm@4.8nm	6.858	11784137	96.47	3209908
3	2998 Ch1 220nm@4.8nm	7.002	182392	1.49	41502

Figure S178. HPLC-UV chromatogram at 220 nm of **4cg.**

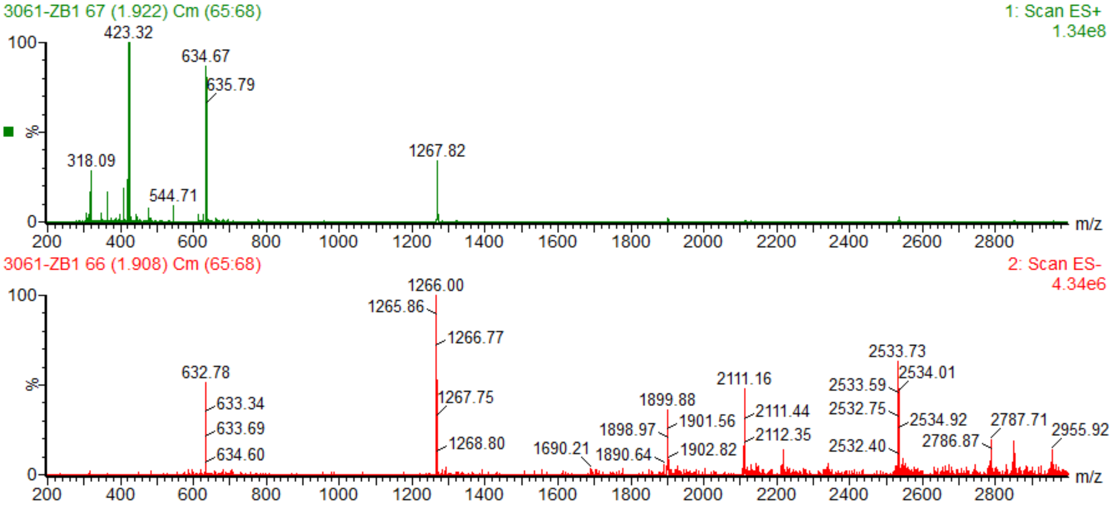

Figure S179. ESI-MS spectrum of **4cg.**

Analysis Report

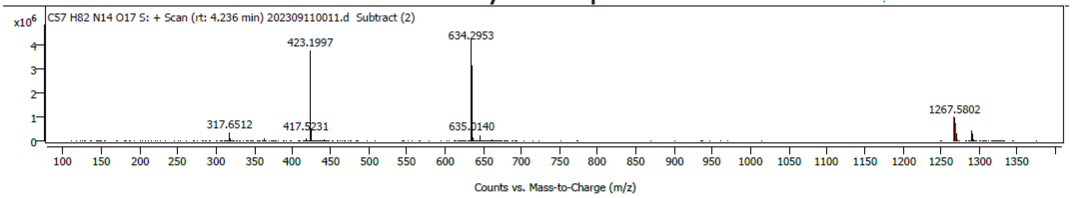

Spectrum Peaks

m/z	Z	Abund	Abund %	m/z (Calc)	Diff (ppm)	Ion Species	Formula	Ion Type
1267.5802	1	953668	22.75	1267.5776	2.09	(M+H)+	C57 H82 N14 O17 S	
1268.5830	1	709326	16.92	1268.5805	1.99	(M+H)+	C57 H82 N14 O17 S	
1269.5838	1	323095	7.71	1269.5818	1.57	(M+H)+	C57 H82 N14 O17 S	
1270.5837	1	105630	2.52	1270.5831	0.53	(M+H)+	C57 H82 N14 O17 S	
317.6512	2	346184	8.26					
317.9018		241817	5.77					
318.1520	2	110554	2.64					
363.1683		99041	2.36					
363.5024	2	58259	1.39					
417.5231	2	89314	2.13					
417.8576		65867	1.57					
423.1997	2	3719800	88.74					
423.3806		210188	5.01					
423.5338	1	2759840	65.84					
423.7144	2	179756	4.29					
423.8674		1347098	32.14					
424.2009	2	514017	12.26					
424.5346	1	131504	3.14					
441.1715		59151	1.41					
634.2953	2	4191813	100.00					
634.7966	2	3095466	73.85					
635.0140	2	187102	4.46					
635.2969	2	1534250	36.60					
635.5158	2	90251	2.15					
635.7972	2	585817	13.98					
636.2971	2	148705	3.55					
645.2848	2	234765	5.60					
645.7861	2	165324	3.94					
646.2864	2	79414	1.89					
661.2534		46448	1.11					
1289.5613	1	412928	9.85					
1290.5640	1	287862	6.87					
1291.5643	1	129651	3.09					
1292.5625	1	42017	1.00					

Spectrum Identification Table

Best ID Source	Name	Formula	Species	m/z	Diff (ppm)	CAS	Score	Score (Lib)	Score (DB)	Score (MFG)	Lib/DB
Yes MFG		C57 H82 N14 O17 S	(M+H)+	1267.5802	1.82		96.12			96.12	

Figure S180. Q-TOF-HRMS spectrum of **4cg.**

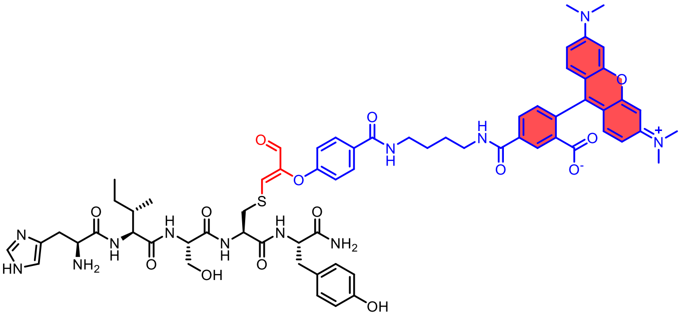

4ch

According to the general procedure B, **1c** (0.04 mmol, 24.8 mg) reacted with **2h** (0.05 mmol, 35.0 mg) to afford the red solid **4ch** (30.6 mg) in 59.2% isolated yield.

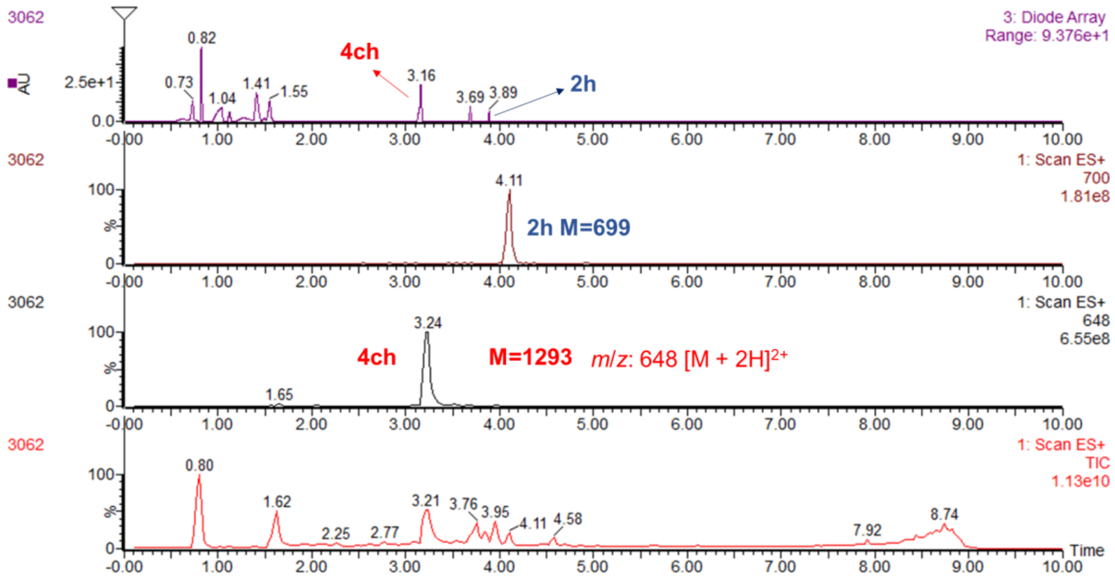

Figure S181. UPLC-MS chromatogram of reaction mixture including TIC and UV curve.

(Extract **2h** and **4ch** mass chromatograms from full scan data).

Analytical **HPLC** using Method D, RT = 11.187 min, the HPLC purity is 100%. **LRMS** (ESI+) *m*/*z*: 1293.79 [M + H]$^+$, (ESI-) *m*/*z*: 1292.04 [M - H]$^-$. **HRMS** (ES+) *m*/*z*: [M + H]$^+$ calcd for $C_{66}H_{76}N_{12}O_{14}S^+$ 1293.5402, found 1293.5378.

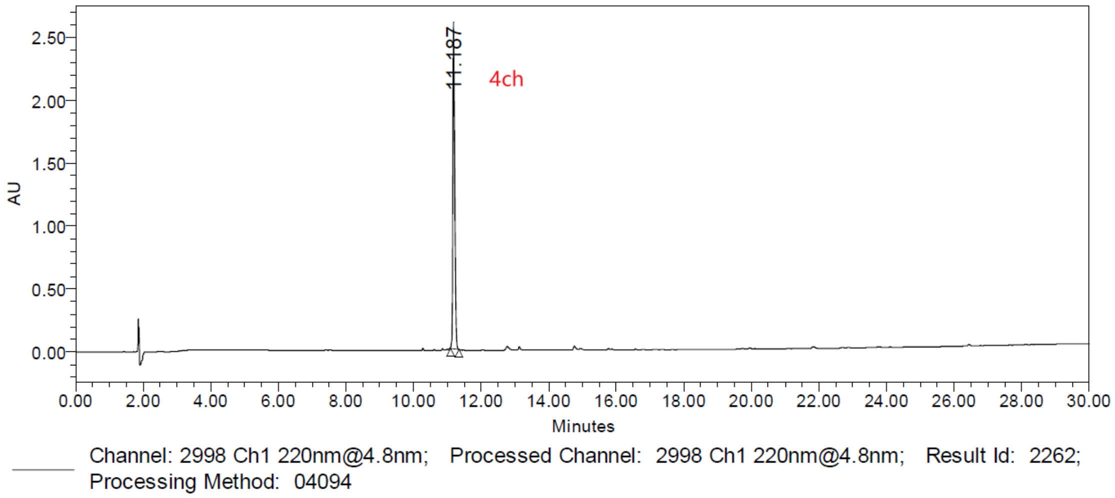

Processed Channel Descr.: 2998 Ch1 220nm@4.8nm

	Processed Channel Descr.	RT	Area	% Area	Height
1	2998 Ch1 220nm@4.8nm	11.187	9989816	100.00	2592015

Figure S182. HPLC-UV chromatogram at 220 nm of **4ch.**

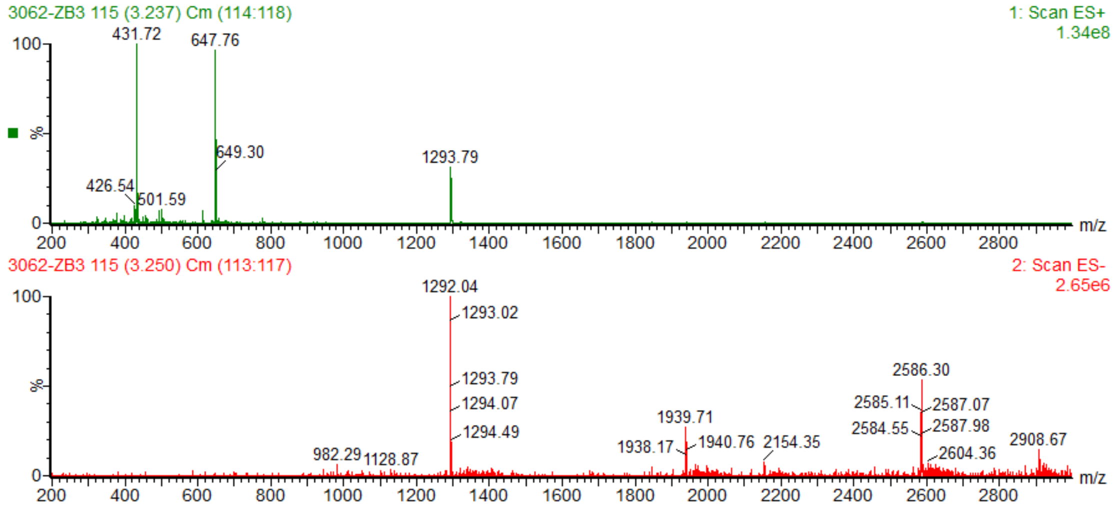

Figure S183. ESI-MS spectrum of **4ch**.

Analysis Report

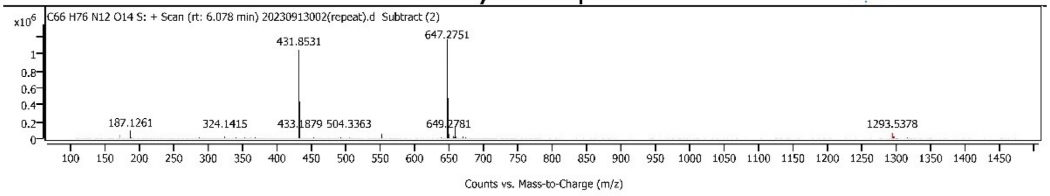

Spectrum Peaks

m/z	Z	Abund	Abund %	m/z (Calc)	Diff (ppm)	Ion Species	Formula	Ion Type
1293.5378	1	59701	5.08	1293.5397	-1.47	(M+H)+	C66 H76 N12 O14 S	
1294.5409	1	48174	4.10	1294.5427	-1.39	(M+H)+	C66 H76 N12 O14 S	
1295.5425	1	22594	1.92	1295.5444	-1.46	(M+H)+	C66 H76 N12 O14 S	
171.1487		46173	3.93					
187.1261		92589	7.88					
324.1415		20313	1.73					
324.3919		14745	1.25					
340.2587		15405	1.31					
362.2401		13877	1.18					
368.1534		12884	1.10					
431.8531	1	1048642	89.23					
432.1878	1	845338	71.93					
432.5211	2	438961	37.35					
432.8542	1	156959	13.36					
433.1879	1	41677	3.55					
453.3423		16242	1.38					
475.3245		18743	1.59					
492.4604		13732	1.17					
504.3363		12453	1.06					
551.7269	2	60211	5.12					
552.2283	2	40090	3.41					
552.7280	2	16495	1.40					
647.2751	2	1175201	100.00					
647.7770	2	915842	77.93					
648.2771	2	475393	40.45					
648.7773	2	172918	14.71					
649.2781	2	51603	4.39					
655.9424	1	20426	1.74					
656.2766		29280	2.49					
656.6099		19372	1.65					
656.9426	1	12274	1.04					
658.2651	2	143987	12.25					
658.7670	2	113416	9.65					
659.2671	2	57201	4.87					
659.7679	2	19335	1.65					
669.2559	2	26002	2.21					
669.7580	2	22750	1.94					
673.6185		14308	1.22					
673.9529		17307	1.47					
674.2790		14106	1.20					

Spectrum Identification Table

Best	ID Source	Name	Formula	Species	m/z	Diff (ppm)	CAS	Score	Score (Lib)	Score (DB)	Score (MFG)	Lib/DB
Yes	MFG		C66 H76 N12 O14 S	(M+H)+	1293.5378	-1.39		97.99			97.99	

Figure S184. Q-TOF-HRMS spectrum of **4ch**.

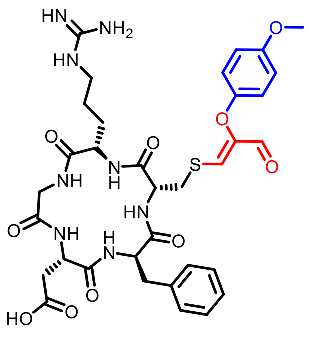

4db

According to the general procedure B, **1d** (0.052 mmol, 30 mg) reacted with **2b** (0.062 mmol, 12.6 mg) to afford the white solid **4db** (24.8 mg) in 63.3% isolated yield.

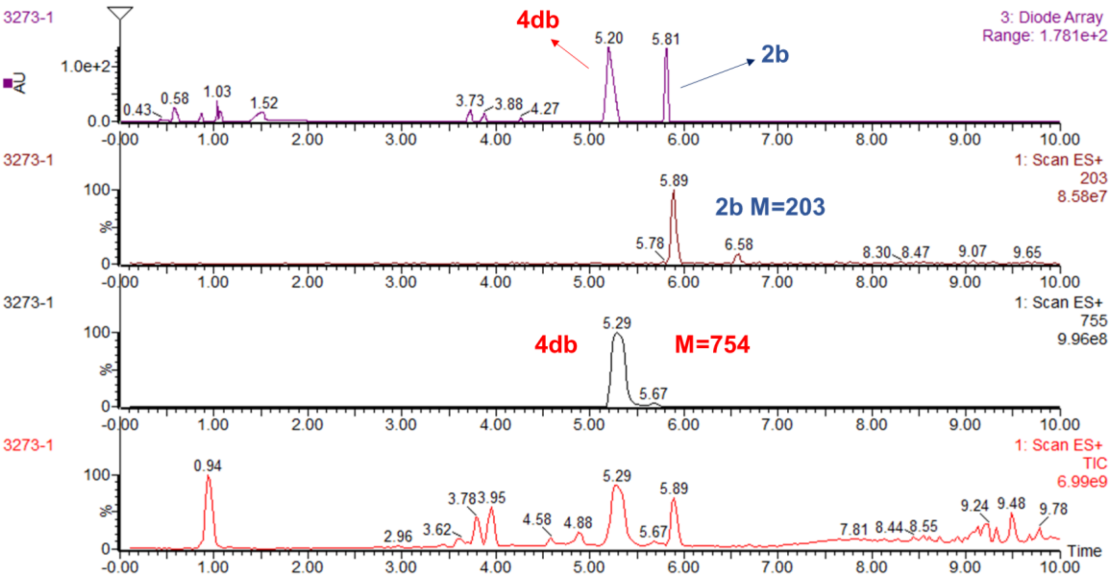

Figure S185. UPLC-MS chromatogram of reaction mixture including TIC and UV curve.

(Extract **2b** and **4bd** mass chromatograms from full scan data).

Analytical **HPLC** using Method D, RT = 12.038 min, the HPLC purity is 97.82%. **LRMS** (ESI+) *m*/*z*: 755.42 [M + H]$^+$, (ESI-) *m*/*z*: 753.67 [M - H]$^-$. **HRMS** (ES+) *m*/*z*: [M + H]$^+$ calcd for $C_{34}H_{42}N_8O_{10}S^+$ 755.2822, found 755.2830.

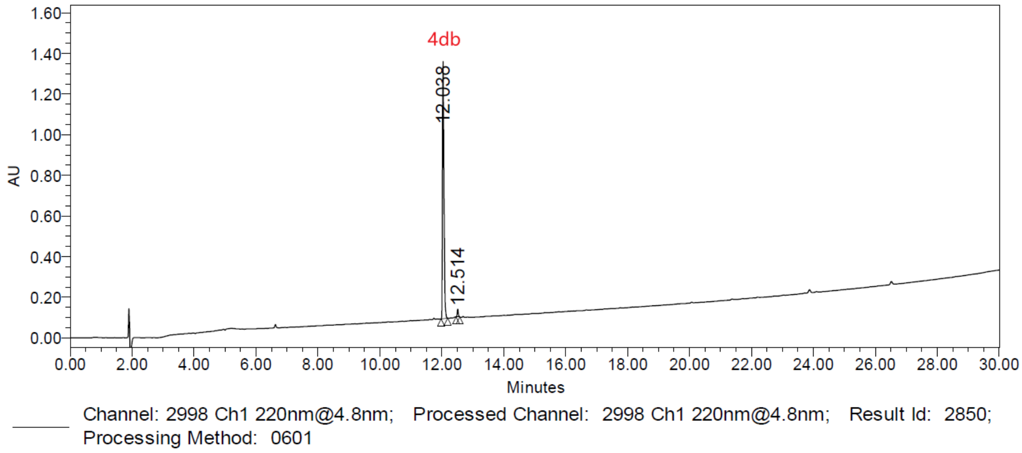

Processed Channel Descr.: 2998 Ch1 220nm@4.8nm

	Processed Channel Descr.	RT	Area	% Area	Height
1	2998 Ch1 220nm@4.8nm	12.038	4164303	97.82	1266374
2	2998 Ch1 220nm@4.8nm	12.514	92745	2.18	38961

Figure S186. HPLC-UV chromatogram at 220 nm of **4db**.

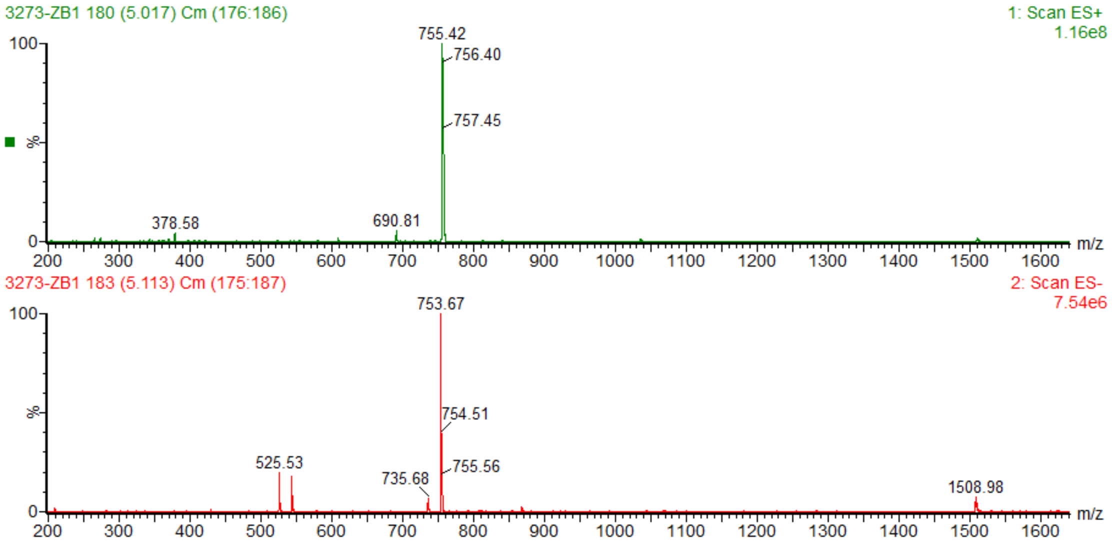

Figure S187. ESI-MS spectrum of **4db**.

Sample Spectra

+ Scan (rt: 6.093 min) Sub **C34 H42 N8 O10 S**

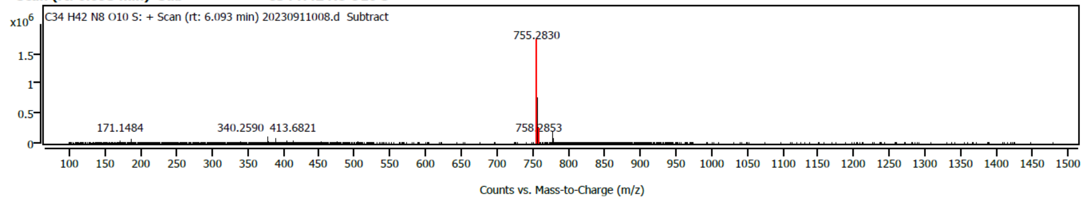

Spectrum Peaks

m/z	Z	Abund	Abund %	m/z (Calc)	Diff (ppm)	Ion Species	Formula	Ion Type
755.2830	1	1698861	100.00	755.2817	1.62	(M+H)+	C34 H42 N8 O10 S	
756.2857	1	758145	44.63	756.2846	1.46	(M+H)+	C34 H42 N8 O10 S	
757.2847	1	266965	15.71	757.2843	0.61	(M+H)+	C34 H42 N8 O10 S	
758.2853	1	63759	3.75	758.2852	0.04	(M+H)+	C34 H42 N8 O10 S	
171.1484		36652	2.16					
187.1261		62213	3.66					
340.2590		19207	1.13					
378.1450	2	99664	5.87					
378.6461	2	40262	2.37					
389.1359	2	73881	4.35					
389.6367	2	33033	1.94					
405.1044		32142	1.89					
413.6821		31594	1.86					
777.2636	1	175030	10.30					
778.2657	1	71366	4.20					
779.2665	1	24657	1.45					

Spectrum Identification Table

Best	ID Source	Name	Formula	Species	m/z	Diff (ppm)	CAS	Score	Score (Lib)	Score (DB)	Score (MFG)	Lib/DB
Yes	MFG		C34 H42 N8 O10 S	(M+H)+	755.2830	1.43		97.49			97.49	

Figure S188. Q-TOF-HRMS spectrum of **4db**.

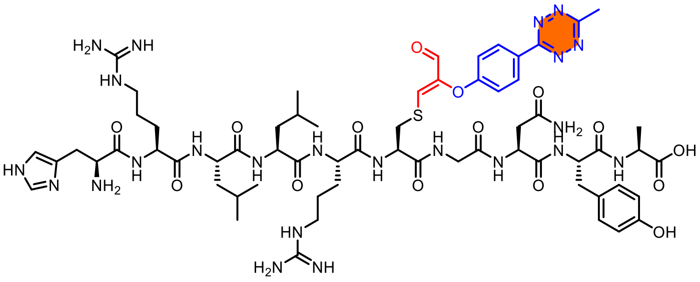

4ji

According to the general procedure B, **1j** (0.02 mmol, 24.0 mg) reacted with **2i** (0.022 mmol, 5.8 mg) to afford the pink solid **4ji** (14.3 mg) in 49.6% isolated yield.

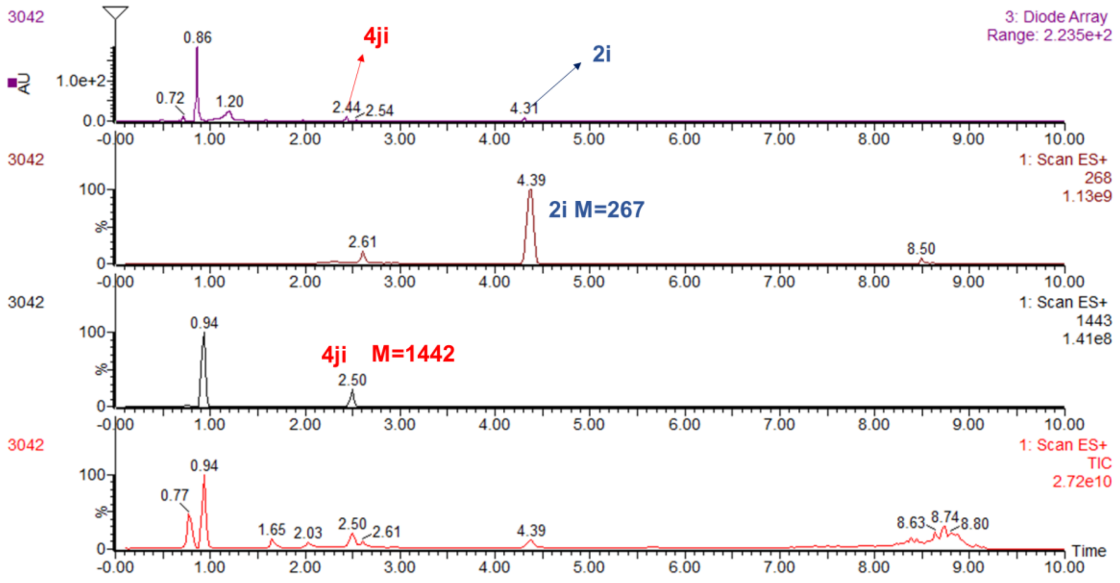

Figure S189. UPLC-MS chromatogram of reaction mixture including TIC and UV curve.

(Extract **2i** and **4ji** mass chromatograms from full scan data).

Analytical **HPLC** using Method D, RT = 10.134 min, the HPLC purity is 99.58%. **LRMS** (ESI+) *m*/*z*: 1442.89 [M + H]$^+$, (ESI-) *m*/*z*: 1441.00 [M - H]$^-$. **HRMS** (ES+) *m*/*z*: [M + H]$^+$ calcd for $C_{63}H_{91}N_{23}O_{15}S^+$ 1442.6863, found 1442.6875.

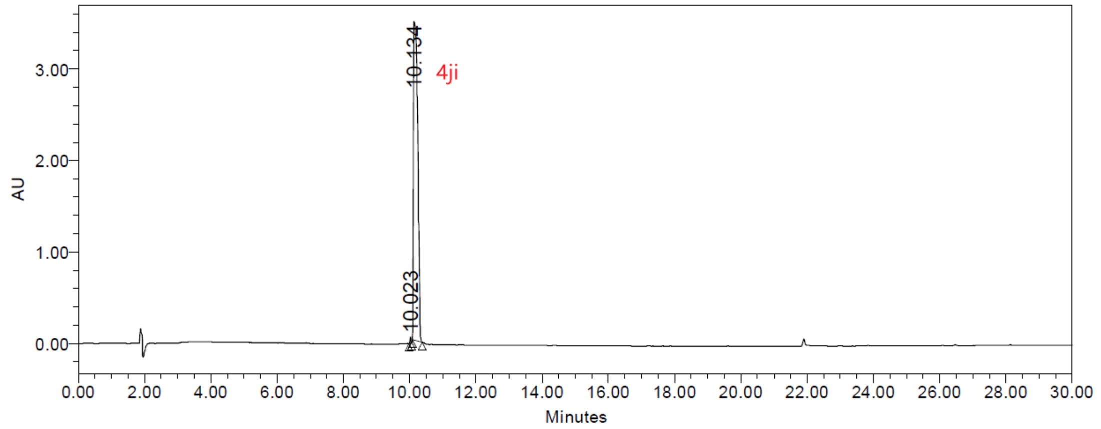

Channel: 2998 Ch1 220nm@4.8nm; Processed Channel: 2998 Ch1 220nm@4.8nm; Result Id: 2201; Processing Method: 04094

Processed Channel Descr.: 2998 Ch1 220nm@4.8nm

	Processed Channel Descr.	RT	Area	% Area	Height
1	2998 Ch1 220nm@4.8nm	10.023	132904	0.42	65817
2	2998 Ch1 220nm@4.8nm	10.134	31445206	99.58	3479405

Figure S190. HPLC-UV chromatogram at 220 nm of **4ji**.

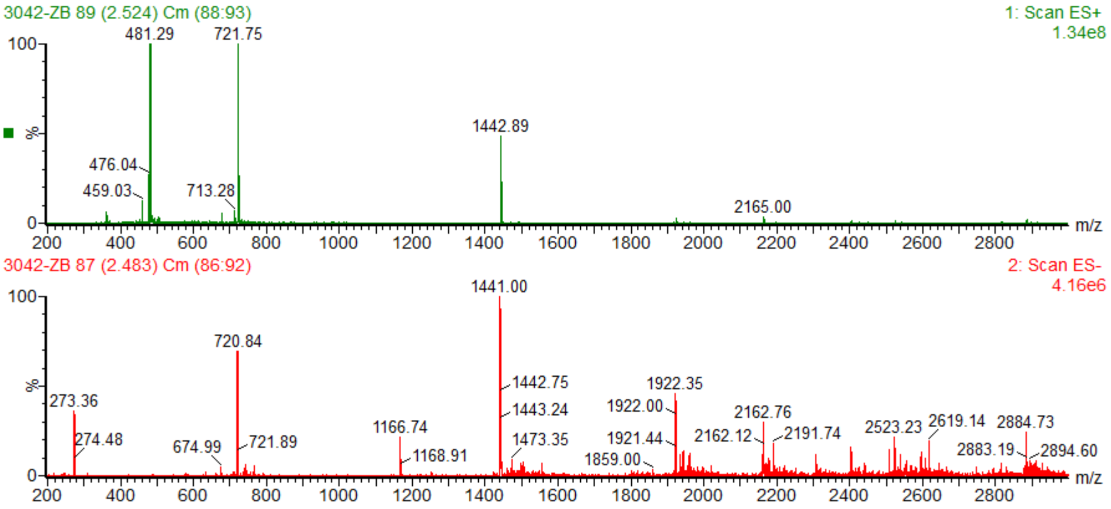

Figure S191. ESI-MS spectrum of **4ji**.

Analysis Report

Agilent Trusted Answers

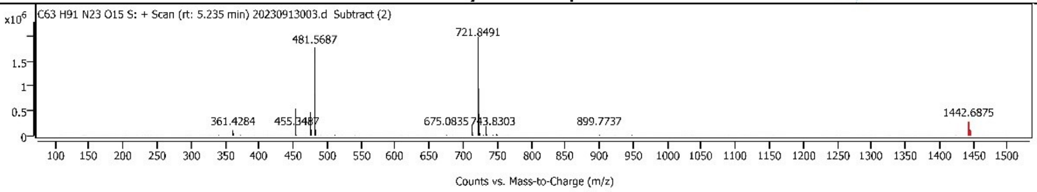

Spectrum Peaks

m/z	Z	Abund	Abund %	m/z (Calc)	Diff (ppm)	Ion Species	Formula	Ion Type
1442.6875	1	289336	14.58	1442.6858	1.15	(M+H)+	C63 H91 N23 O15 S	
1443.6894	1	239583	12.08	1443.6885	0.63	(M+H)+	C63 H91 N23 O15 S	
1444.6910	1	123530	6.23	1444.6900	0.70	(M+H)+	C63 H91 N23 O15 S	
1445.6910	1	48845	2.46	1445.6913	-0.22	(M+H)+	C63 H91 N23 O15 S	
361.4284	2	119617	6.03					
361.6784	2	101675	5.12					
361.9303	2	80333	4.05					
362.1851	2	50723	2.56					
372.2582		22518	1.13					
453.3437	1	553743	27.91					
454.3462	1	146534	7.39					
455.3487	1	23711	1.20					
475.3256	1	474196	23.90					
475.5650	1	147901	7.45					
475.8984		118573	5.98					
476.2330		61427	3.10					
476.3271	1	133521	6.73					
476.5658	1	23124	1.17					
477.3301	1	21863	1.10					
481.5687	1	1760806	88.75					
481.9029	1	1442177	72.69					
482.2369	1	809184	40.79					
482.5708	1	352360	17.76					
482.9049	1	124031	6.25					
483.2387	1	30944	1.56					
675.0835		20096	1.01					
712.8428	2	208538	10.51					
713.3436	2	162645	8.20					
713.8443	2	80817	4.07					
714.3451	2	30765	1.55					
721.8491	2	1983998	100.00					
722.3502	2	1670364	84.19					
722.8515	2	942573	47.51					
723.3524	2	433808	21.87					
723.8504	2	169680	8.55					
724.3476	2	53252	2.68					
732.8391	2	248596	12.53					
733.3405	2	201212	10.14					
733.8411	2	114339	5.76					
734.3414	2	49072	2.47					
743.8303		23310	1.17					
748.8080	2	38585	1.94					
749.3100	2	27940	1.41					
749.8249	2	22156	1.12					
899.7737		20204	1.02					

Spectrum Identification Table

Best	ID Source	Name	Formula	Species	m/z	Diff (ppm)	CAS	Score	Score (Lib)	Score (DB)	Score (MFG)	Lib/DB
Yes	MFG		C63 H91 N23 O15 S	(M+H)+	1442.6875	0.72		98.38			98.38	

Figure S192. Q-TOF-HRMS spectrum of **4ji**.

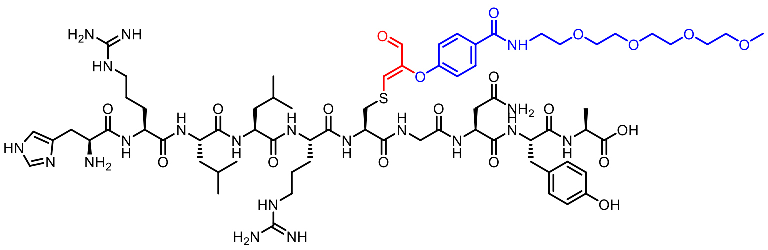

4jj

According to the general procedure B, **1j** (0.015 mmol, 18.0 mg) reacted with **2j** (0.018 mmol, 7.0 mg) to afford the white solid **4jj** (19.9 mg) in 84.0% isolated yield.

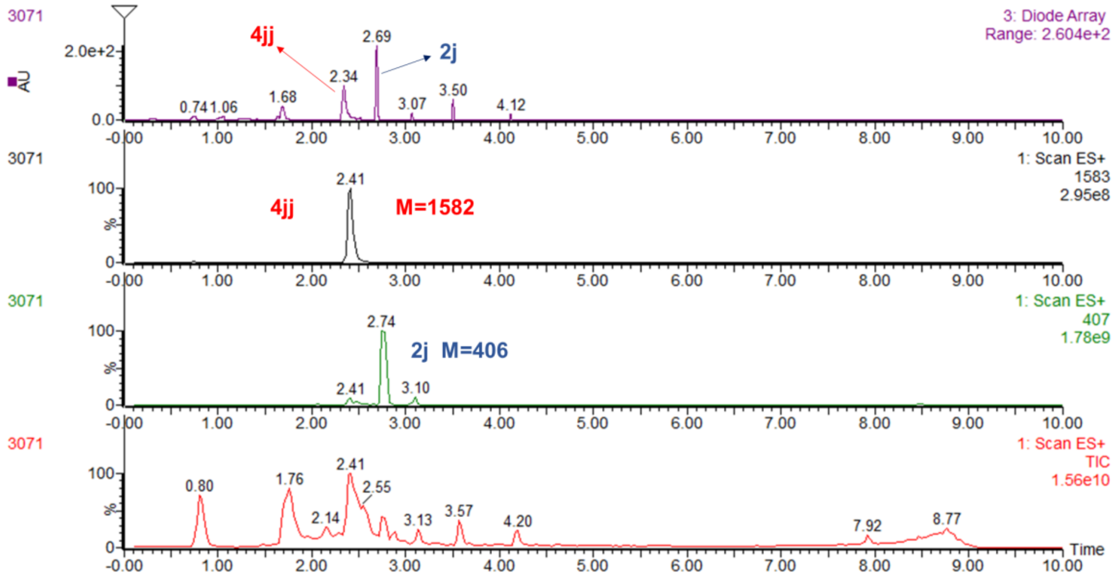

Figure S193. UPLC-MS chromatogram of reaction mixture including TIC and UV curve.

(Extract **2j** and **4jj** mass chromatograms from full scan data).

Analytical **HPLC** using Method D, RT = 6.750 min, the HPLC purity is 96.82%. **LRMS** (ESI+) *m/z*: 1583.11 $[M + H]^+$, (ESI-) *m/z*: 1580.03 $[M - H]^-$. **HRMS** (ES+) *m/z*: $[M + 2H]^{2+}$ calcd for $C_{70}H_{108}N_{20}O_{20}S^{+2}$ 791.3963, found 791.3996.

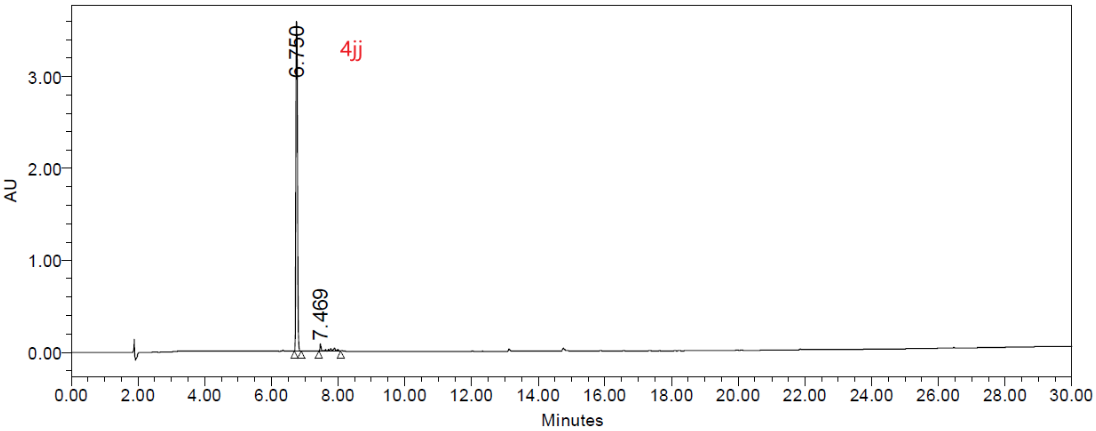

Channel: 2998 Ch1 220nm@4.8nm; Processed Channel: 2998 Ch1 220nm@4.8nm; Result Id: 2283; Processing Method: 04094

Processed Channel Descr.: 2998 Ch1 220nm@4.8nm

	Processed Channel Descr.	RT	Area	% Area	Height
1	2998 Ch1 220nm@4.8nm	6.750	12375241	96.82	3582123
2	2998 Ch1 220nm@4.8nm	7.469	406342	3.18	75097

Figure S194. HPLC-UV chromatogram at 220 nm of **4jj**.

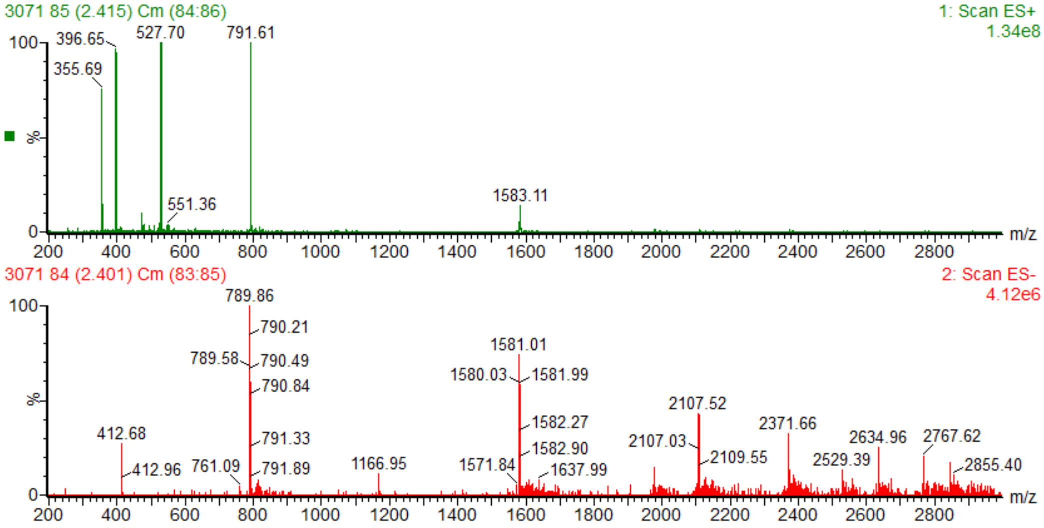

Figure S195. ESI-MS spectrum of **4jj**.

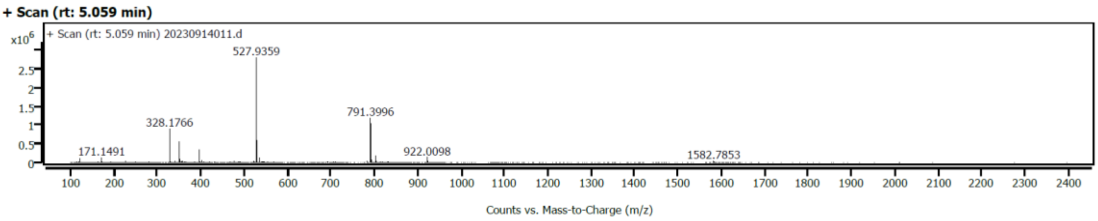

Figure S196. Q-TOF-HRMS spectrum of **4jj**.

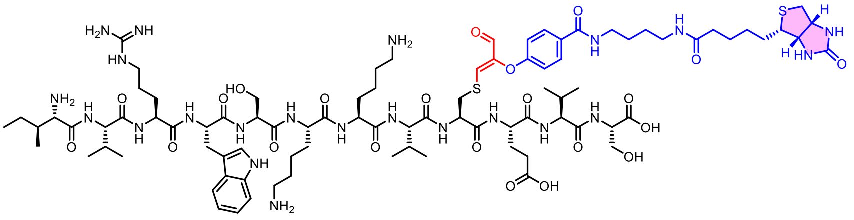

4lk

According to the general procedure B, **1l** (0.015 mmol, 21.5 mg) reacted with **2k** (0.018 mmol, 9.2 mg, dissolved in DMF) to afford the white solid **4lk** (17.7 mg) in 61.5% isolated yield.

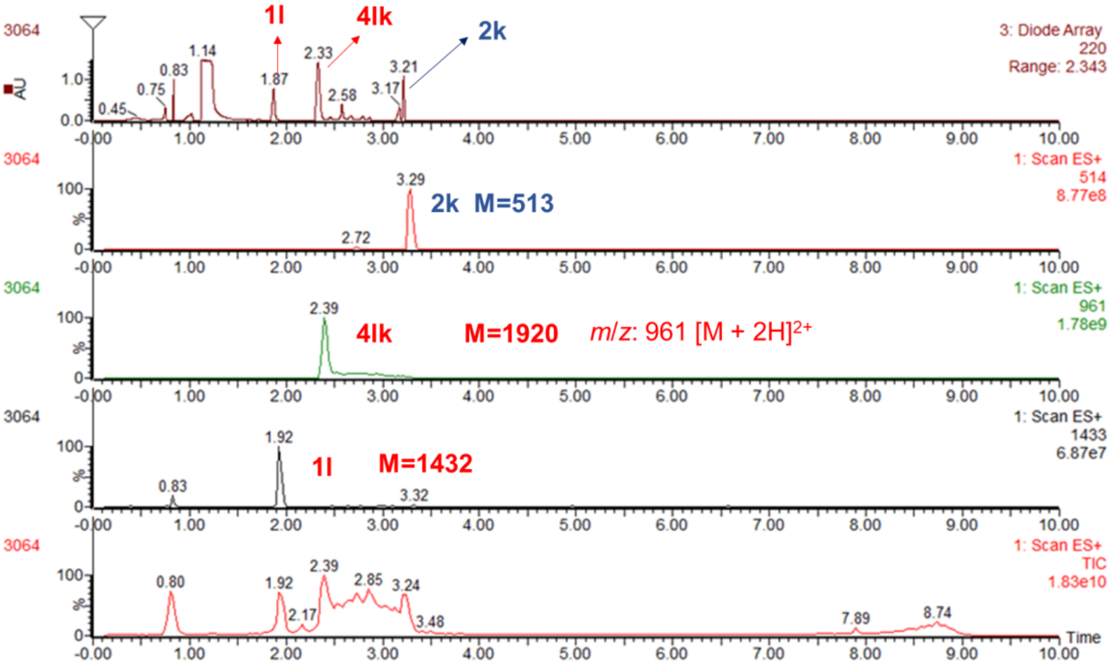

Figure S197. UPLC-MS chromatogram of reaction mixture including TIC and UV curve.

(Extract **1l**, **2k** and **4lk** mass chromatograms from full scan data).

Analytical **HPLC** using Method D, RT = 8.814 min, the HPLC purity is 99.74%. **LRMS** (ESI+) *m/z*: 1920.11 $[M + H]^+$, (ESI-) *m/z*: 1918.43 $[M - H]^-$. **HRMS** (ES+) *m/z*: $[M + 2H]^{2+}$ calcd for $C_{88}H_{138}N_{22}O_{22}S_2^{+2}$ 960.4976, found 960.5095.

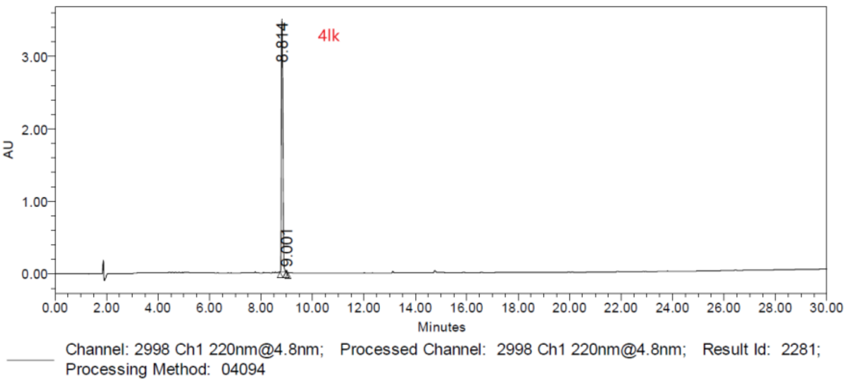

Processed Channel Descr.: 2998 Ch1 220nm@4.8nm

	Processed Channel Descr.	RT	Area	% Area	Height
1	2998 Ch1 220nm@4.8nm	8.814	14316763	99.74	3481693
2	2998 Ch1 220nm@4.8nm	9.001	37927	0.26	21641

Figure S198. HPLC-UV chromatogram at 220 nm of **4lk**.

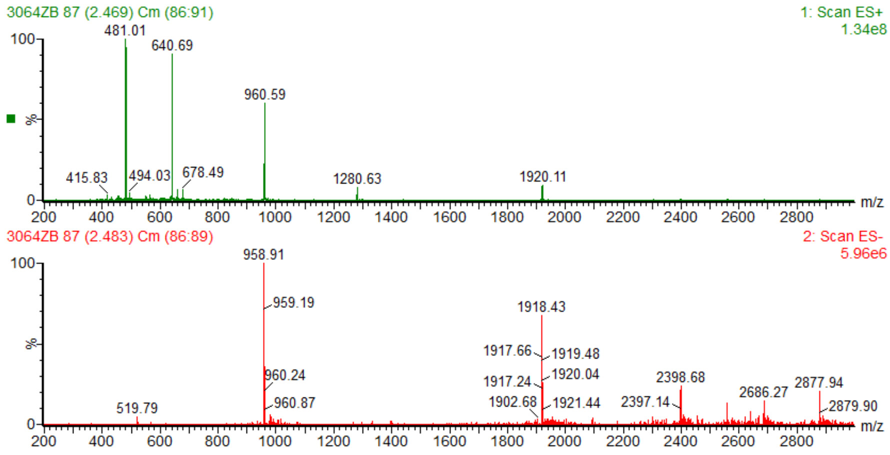

Figure S199. ESI-MS spectrum of **4lk**.

样品谱图

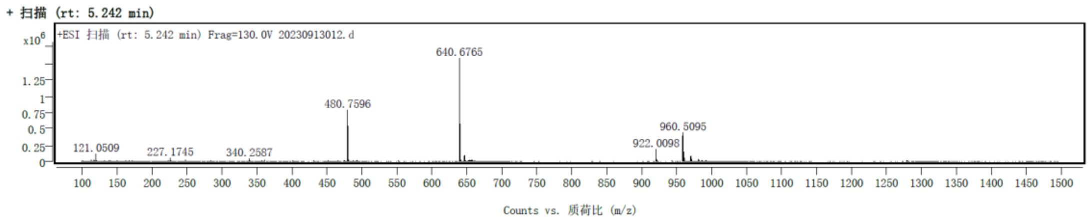

Figure S200. Q-TOF-HRMS spectrum of **4lk**.

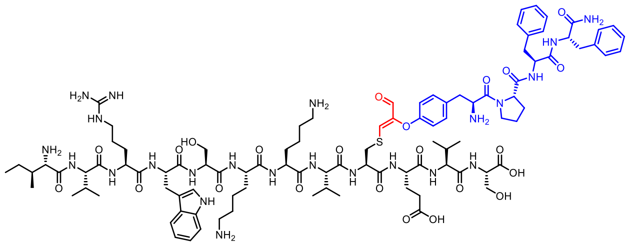

4ll

According to the general procedure B, **1l** (0.025 mmol, 35.8 mg) reacted with **2l** (0.03 mmol, 19.5 mg) to afford the white solid **4ll** (29.7 mg) in 57.7% isolated yield.

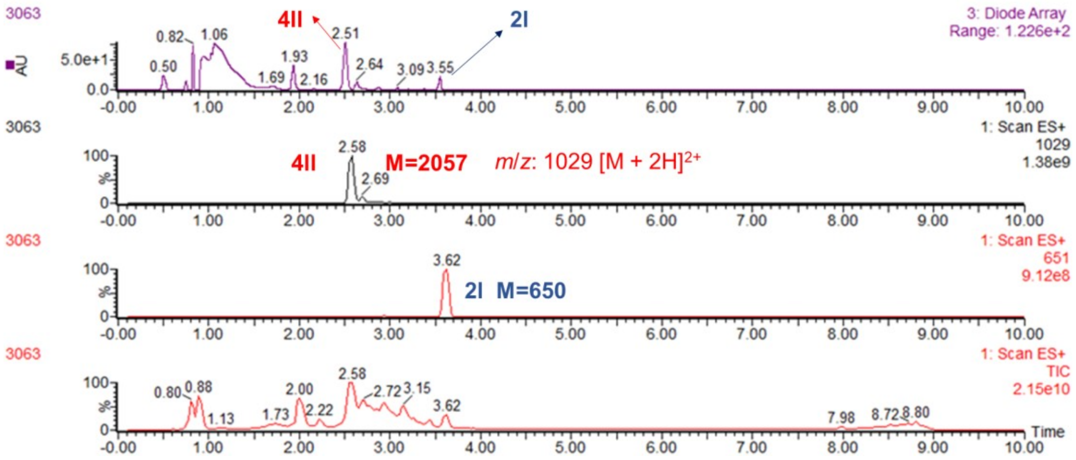

Figure S201. UPLC-MS chromatogram of reaction mixture including TIC and UV curve.

(Extract **2l** and **4ll** mass chromatograms from full scan data).

Analytical **HPLC** using Method D, RT = 9.746 min, the HPLC purity is 95.46%. **LRMS** (ESI+) *m*/*z*: 2057.46 [M + H]$^+$, (ESI-) *m*/*z*: 2055.29 [M - H]$^-$. **HRMS** (ES+) *m*/*z*: [M + H]$^+$ calcd for $C_{99}H_{145}N_{23}O_{23}S^+$ 2057.0682, found 2057.0839.

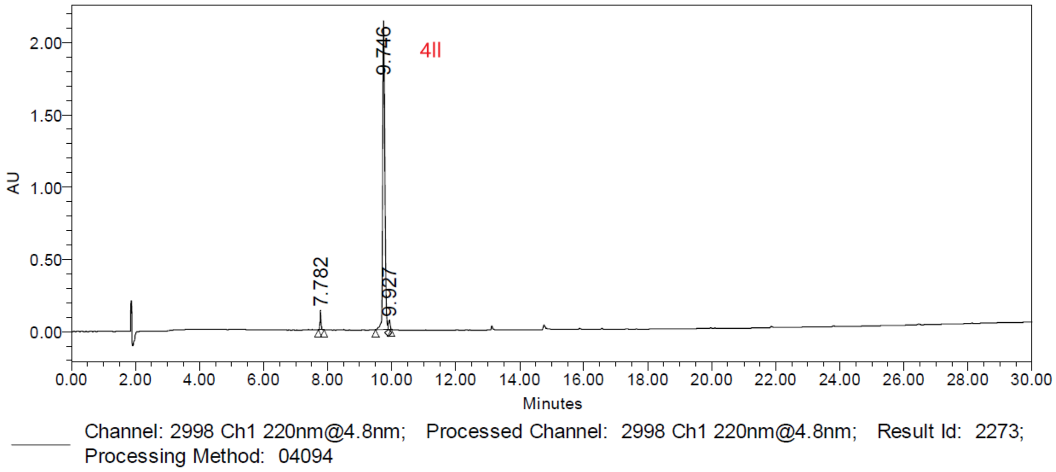

Processed Channel Descr.: 2998 Ch1 220nm@4.8nm

	Processed Channel Descr.	RT	Area	% Area	Height
1	2998 Ch1 220nm@4.8nm	7.782	279260	2.66	133994
2	2998 Ch1 220nm@4.8nm	9.746	10039860	95.46	2134811
3	2998 Ch1 220nm@4.8nm	9.927	198503	1.89	62269

Figure S202. HPLC-UV chromatogram at 220 nm of **4ll.**

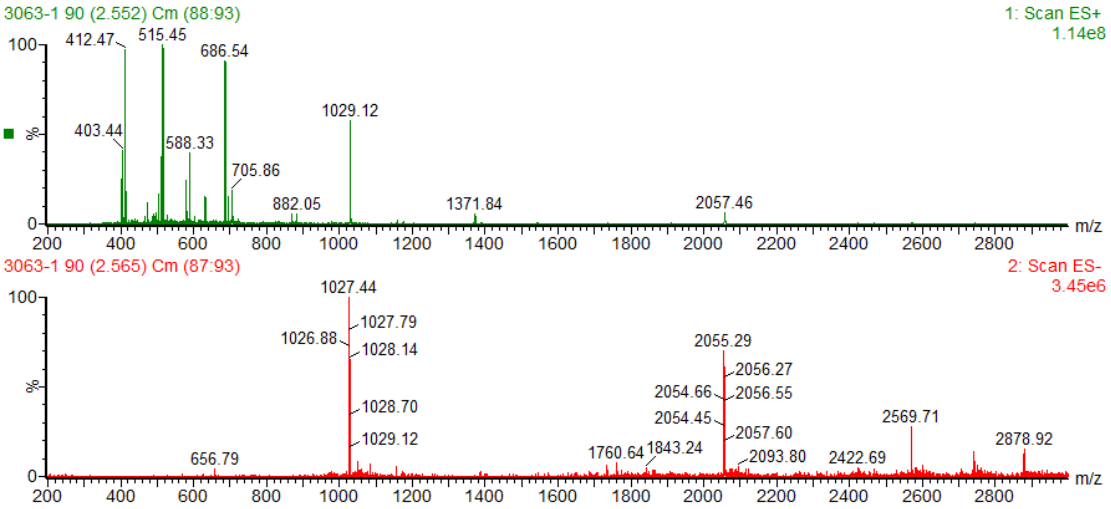

Figure S203. ESI-MS spectrum of **4ll**.

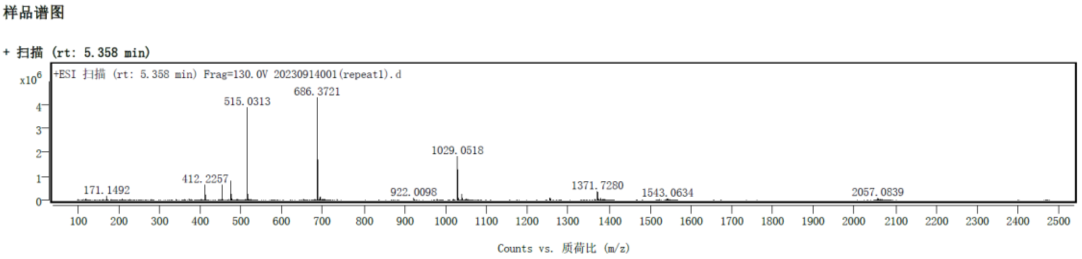

Figure S204. Q-TOF-HRMS spectrum of **4ll**.

8 Secondary labelling and one-pot triple functionalization

8.1 Secondary labelling by ketone-hydroxyl amine condensation

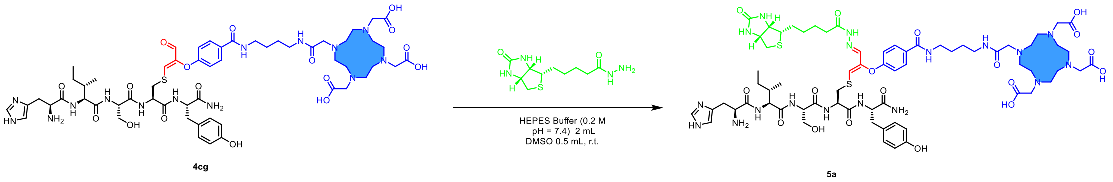

Dissolve compound **4cg** (0.01 mmol, 12.6mg) in 2 mL HEPES buffer (pH = 7.4, 0.2 M), and add 0.5 mL DMSO dissolving (+)-Biotin hydrazide (0.05 mmol, 12.9mg, 5 eq.). Then the reaction process was monitored by LC-MS at 1 h, 6 h, 12 h, and 24 h after the reaction started, respectively. Over time, **4cg** gradually disappeared and the target product **5a** gradually increased. After 24 h, the reaction was stopped and the product **5a** was separated by RP-HPLC in 41.8% isolated yield (6.3 mg).

5a: $C_{67}H_{98}N_{18}O_{18}S_2$, Analytical **HPLC** using Method D, RT = 7.414 min, the HPLC purity is 95.23%. **LRMS** (ESI+) *m/z*: 1507.86 $[M + H]^+$, (ESI-) *m/z*: 1505.97 $[M - H]^-$. **HRMS** (ES+) *m/z*: $[M + H]^+$ calcd for $C_{67}H_{98}N_{18}O_{18}S_2^+$ 1507.6825, found 1507.6843.

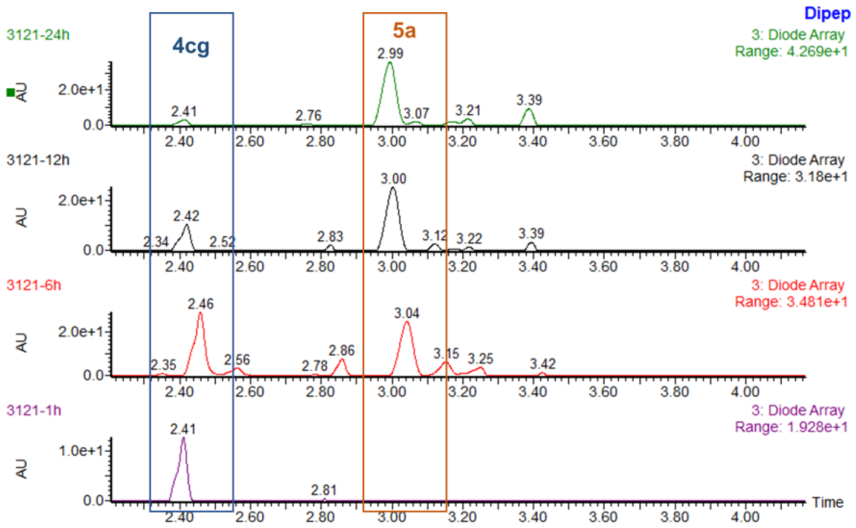

Figure S205. LCMS-UV chromatogram between 190 nm – 450 nm.

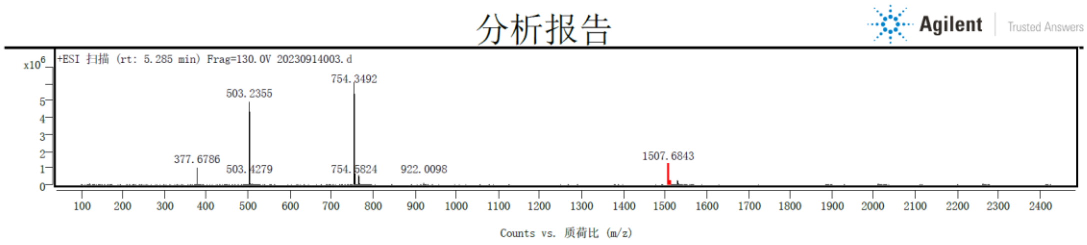

Figure S206. Q-TOF-HRMS spectrum of **5a**.

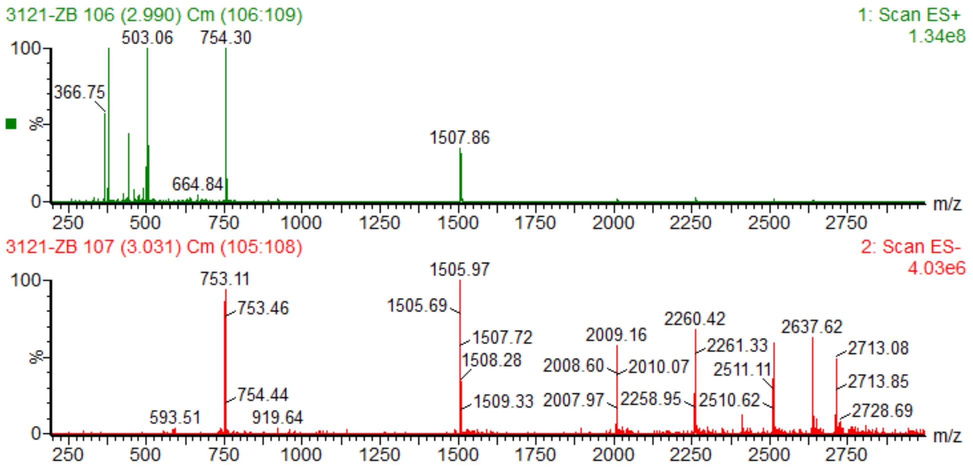

Figure S207. ESI-MS spectrum of **5a**.

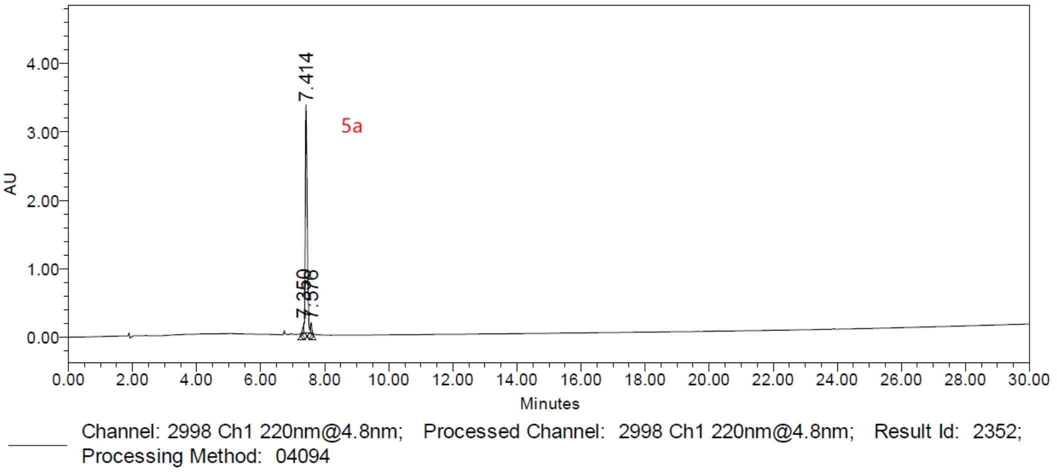

Processed Channel Descr.: 2998 Ch1 220nm@4.8nm

	Processed Channel Descr.	RT	Area	% Area	Height
1	2998 Ch1 220nm@4.8nm	7.350	327498	2.25	146322
2	2998 Ch1 220nm@4.8nm	7.414	13830899	95.23	3330165
3	2998 Ch1 220nm@4.8nm	7.576	365756	2.52	142573

Figure S208. HPLC-UV chromatogram at 220 nm of **5a**.

8.2 Secondary labelling by tetrazine ligation

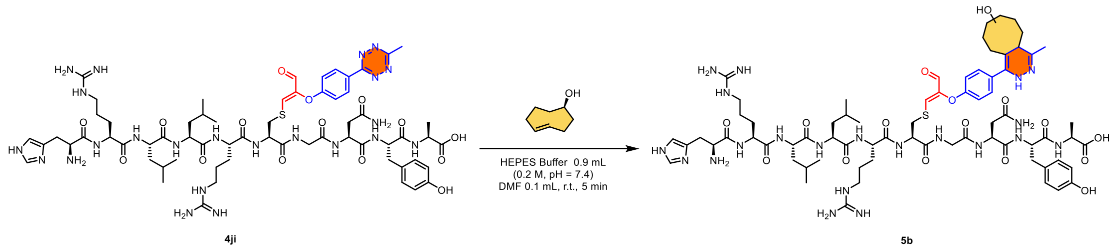

4ji (8.3 µmol, 12.0 mg) was dissolved in 0.9 mL HEPES buffer (0.2 M, pH = 7.4), then add **TCO-OH** (41.5 µmol, 5.3 mg, 5 eq.) dissolved in 0.1 mL DMF. After 5 min, the reaction was detected by LCMS and two isomers (**5b-1, 5b-2**) were generated, which was caused by the inherent reaction between TCO-OH and tetrazine. The product was identified by LCMS, and the ratio of isomers was 8790:7008 = 5:4. Through RP-HPLC, it was separated to obtain **5b-1** (3.8 mg) and **5b-2** (3 mg). The total yield was 53.2 %.

Analytical **HPLC** using Method D, **5b-1** : RT = 9.315 min, the HPLC purity is 93.48%; **5b-2** : RT = 9.780 min, the HPLC purity is 93.61%. **LRMS** (ESI+) *m/z*: 1541.95 [M + H]$^+$, (ESI-) *m/z*: 1539.29 [M - H]$^-$. **HRMS** (ES+) *m/z*: [M + H]$^+$ calcd for $C_{71}H_{105}N_{21}O_{16}S^+$ 1540.7847, found 1539.7715.

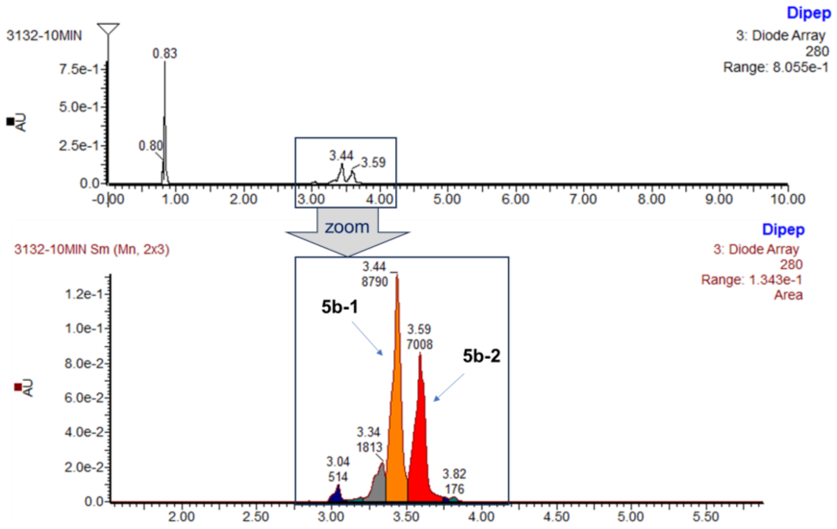

Figure S209. HPLC-UV chromatogram at 280 nm of **4cf**.

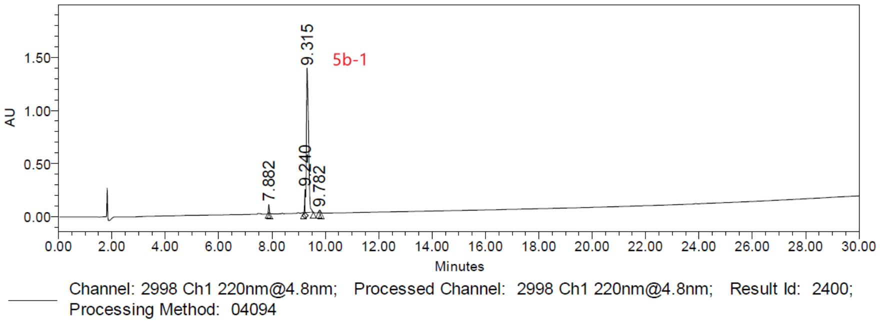

Processed Channel Descr.: 2998 Ch1 220nm@4.8nm

	Processed Channel Descr.	RT	Area	% Area	Height
1	2998 Ch1 220nm@4.8nm	7.882	138749	1.72	74291
2	2998 Ch1 220nm@4.8nm	9.240	320186	3.96	217068
3	2998 Ch1 220nm@4.8nm	9.315	7559552	93.48	1355436
4	2998 Ch1 220nm@4.8nm	9.782	67945	0.84	18776

Figure S210. HPLC-UV chromatogram at 220 nm of **5b-1**.

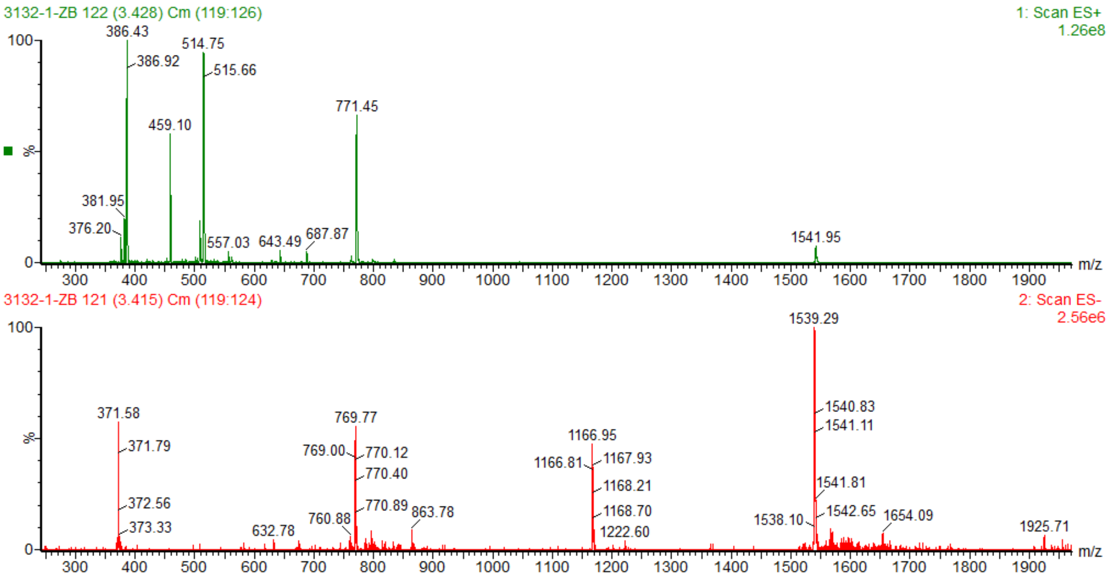

Figure S211. ESI-MS spectrum of **5b-1**.

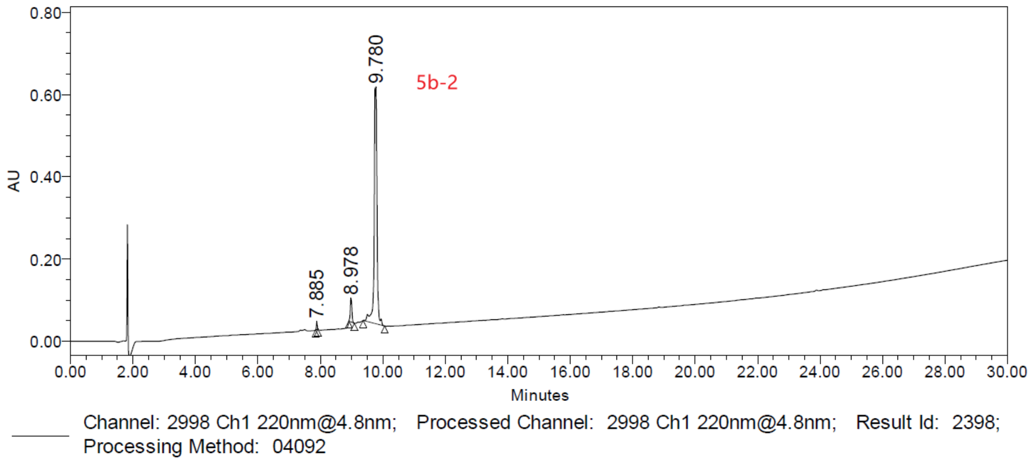

Processed Channel Descr.: 2998 Ch1 220nm@4.8nm

	Processed Channel Descr.	RT	Area	% Area	Height
1	2998 Ch1 220nm@4.8nm	7.885	38013	0.98	19443
2	2998 Ch1 220nm@4.8nm	8.978	209283	5.41	57368
3	2998 Ch1 220nm@4.8nm	9.780	3623240	93.61	576223

Figure S212. HPLC-UV chromatogram at 220 nm of **5b-2**.

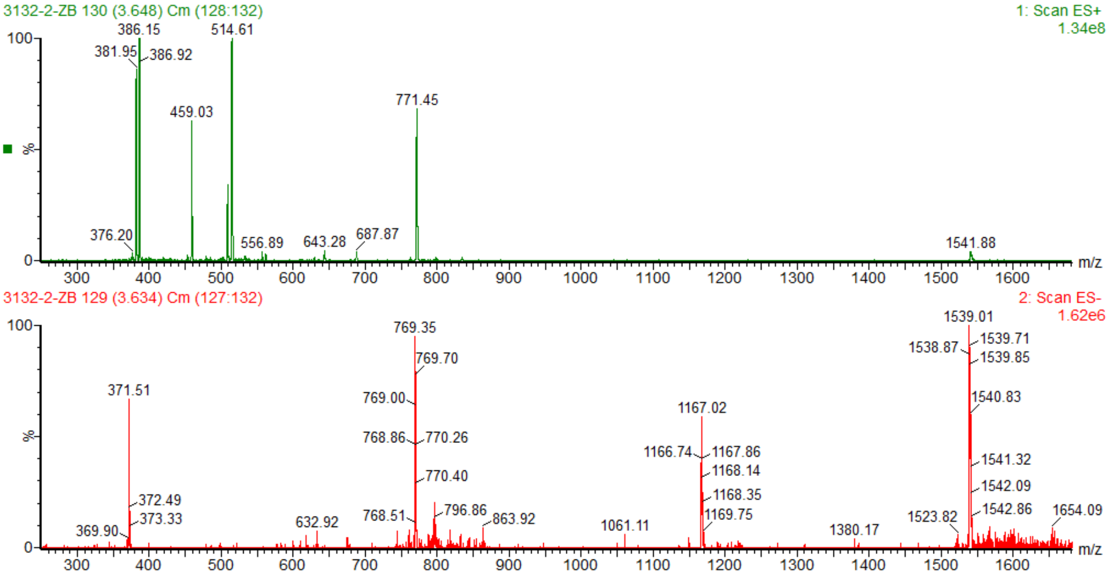

Figure S213. ESI-MS spectrum of **5b-2**.

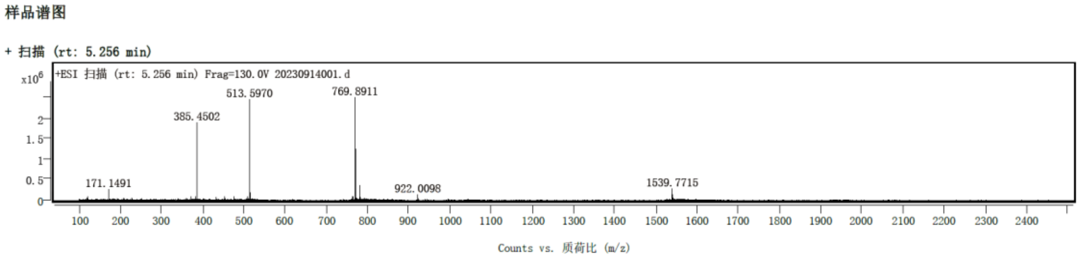

Figure S214. Q-TOF-HRMS spectrum of **5b-2**.

8.3 One-pot triple functionalization

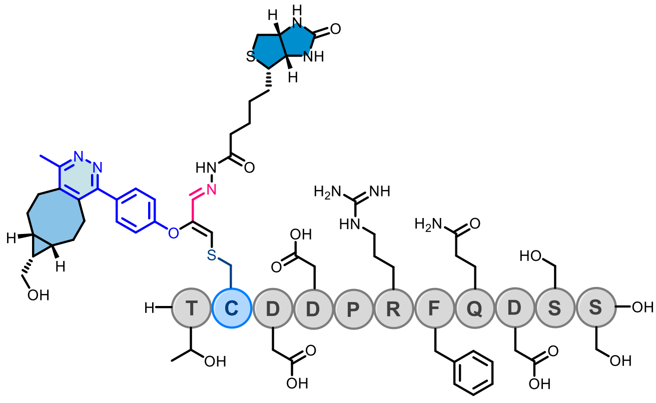

5e

1n (15.8 µmol, 20.0 mg) was dissolved in 0.9 mL HEPES buffer (0.2 M, pH = 7.4), then add **2i** (17.3 µmol, 4.6 mg, 1.1 eq.) dissolved in 0.1 mL CH_3CN. The reaction system was stirred at room temperature for 2.0 h (monitored by LC-MS). A dimer of **1n** was observed (RT = 1.66 min) and the major product was **5c** (RT = 2.33 min). Then, **BCN-OH** (18.6 µmol,

2.8 mg, 1.2 eq., dissolved in 0.1 mL CH_3OH) was added and stir them for 15 min. The LCMS results showed that the main product **5d** was formed (RT = 2.11 min), and **by-product** was also observed (RT = 2.51 min), which is the product of the reaction between **2i** and **BCN-OH**. Next, 0.5 mL of (+)-Biotin hydrazide (79 µmol, 20 mg, 5 eq.) in DMSO solution was added. After 24 h, **5e** generated (RT = 2.18 min), and was isolated by HPLC in 46.0% yield (13.6 mg).

Analytical **HPLC** using Method D, RT = 7.742 min, the HPLC purity is 99.58%. **LRMS** (ESI+) *m/z*: 1874.24 $[M + H]^+$, (ESI-) *m/z*:1872.13 $[M - H]^-$. **HRMS** (ES+) *m/z*: $[M + H]^+$ calcd for $C_{82}H_{113}N_{21}O_{26}S_2^+$ 1873.7764, found1873.7681.

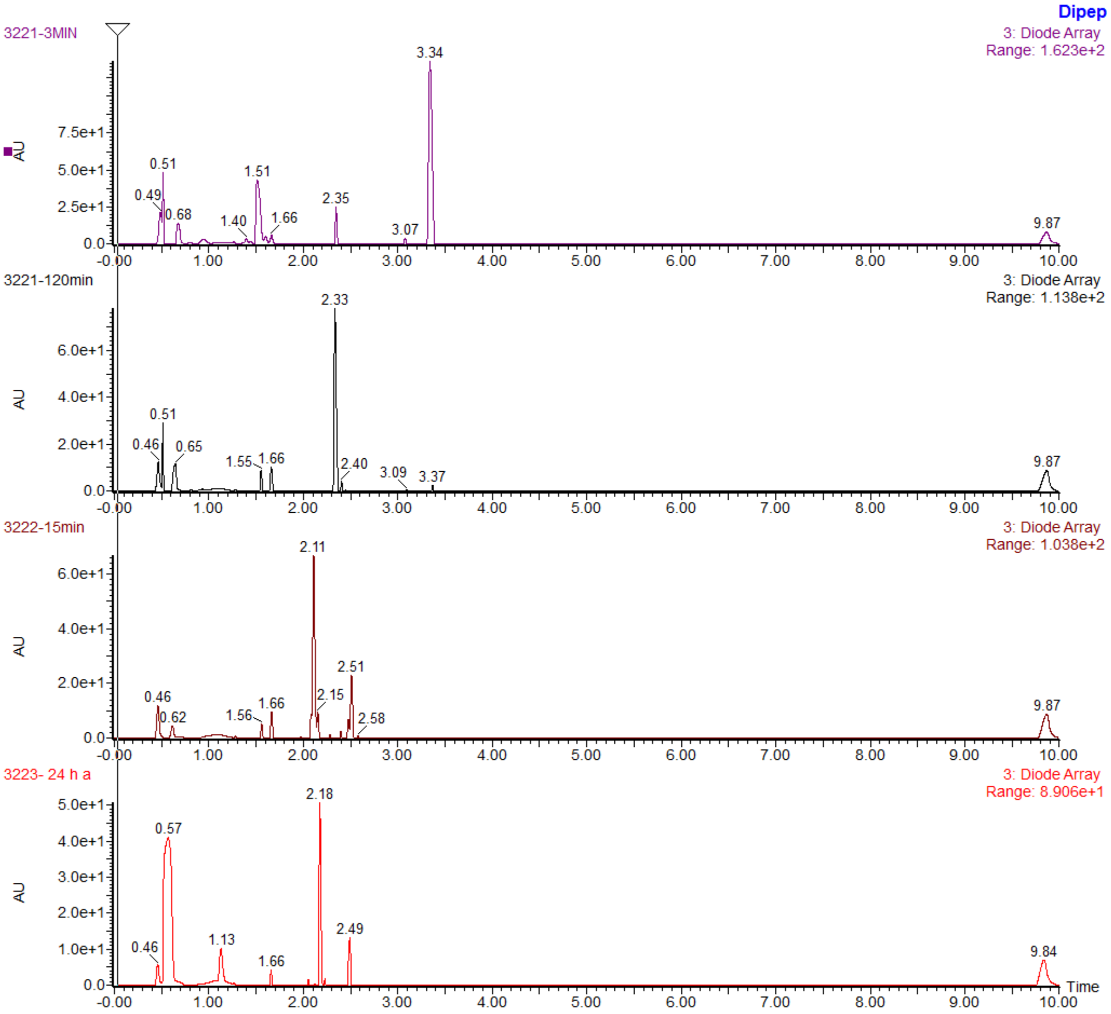

Figure S215. LCMS-UV chromatogram at corresponding time (190 nm – 480 nm) using method C.

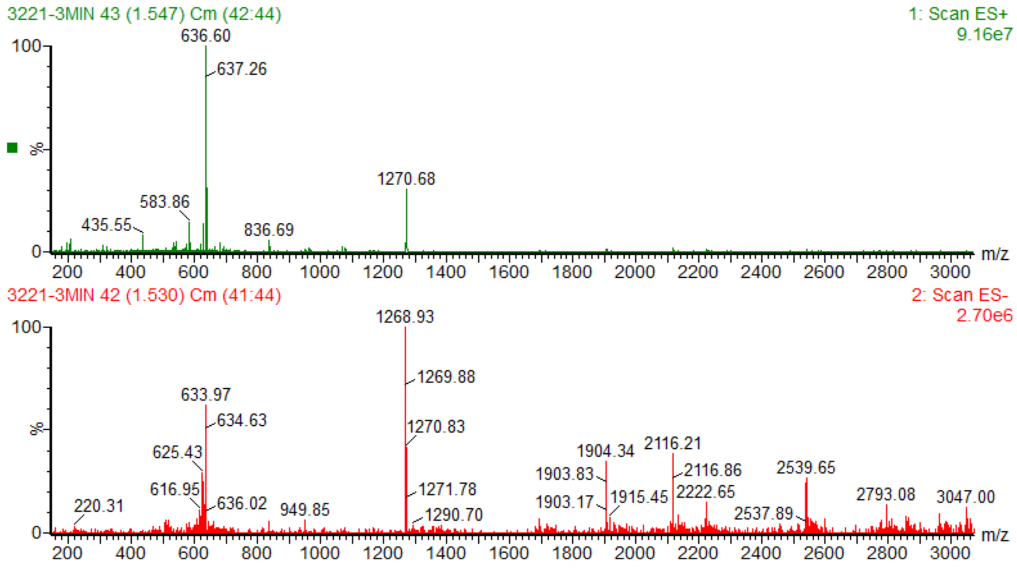

Figure S216. ESI-MS spectrum of **1n**.

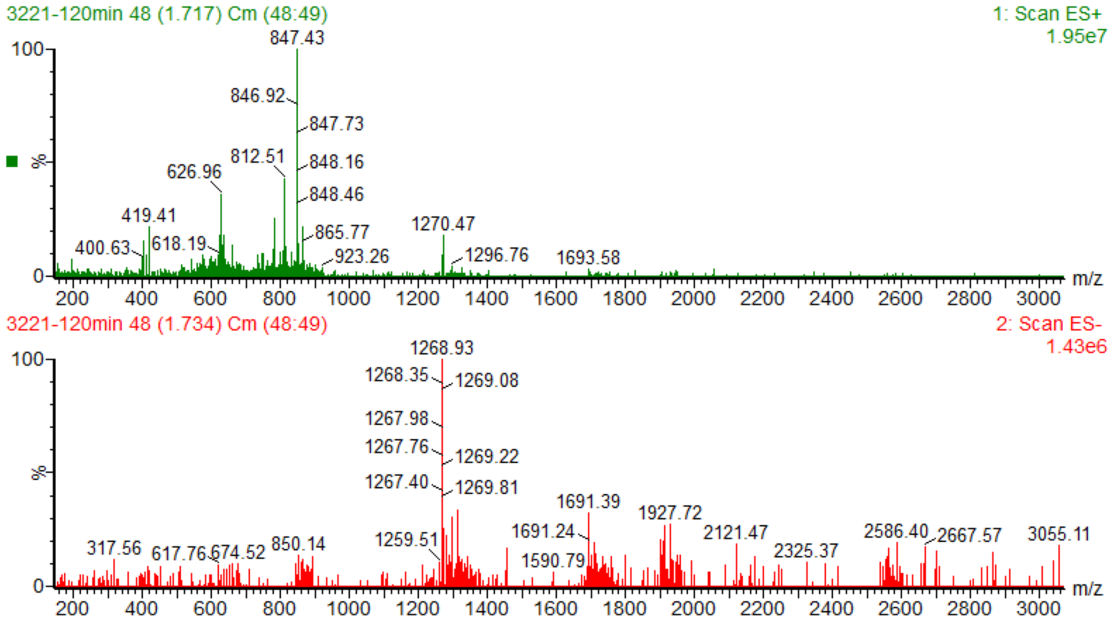

Figure S217. ESI-MS spectrum of **dimer of 1n**

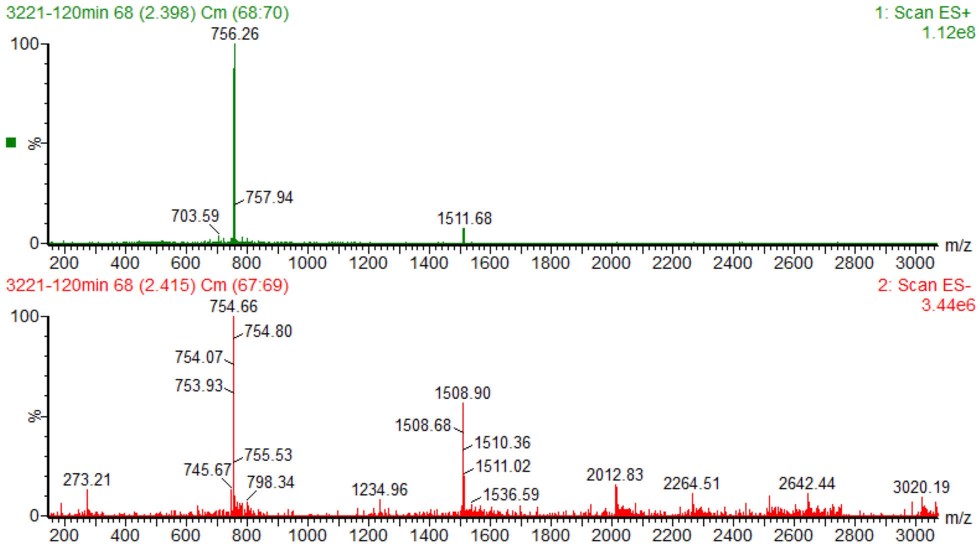

Figure S218. ESI-MS spectrum of **5c**.

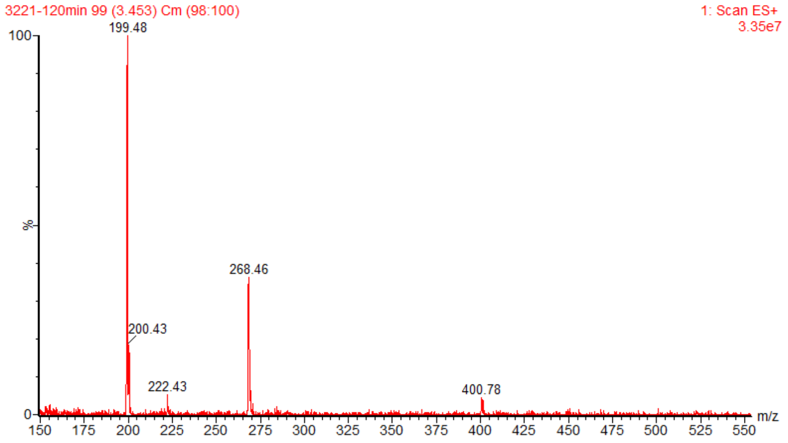

Figure S219. ESI-MS spectrum of **2i**.

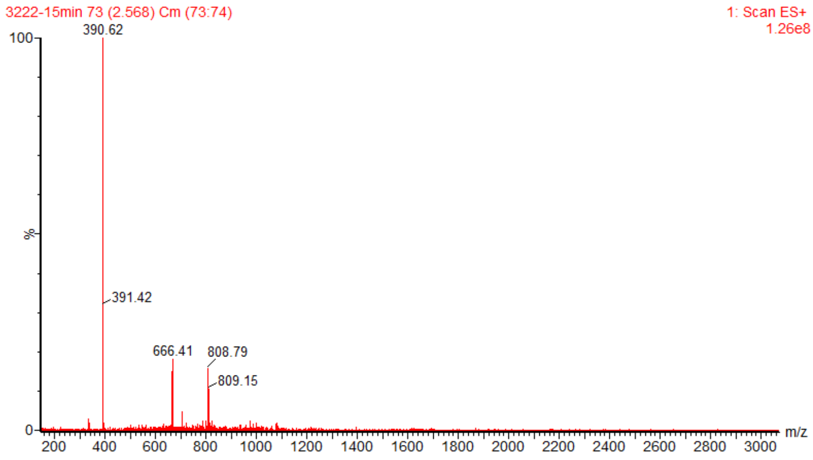

Figure S220. ESI-MS spectrum of **byproduct**.

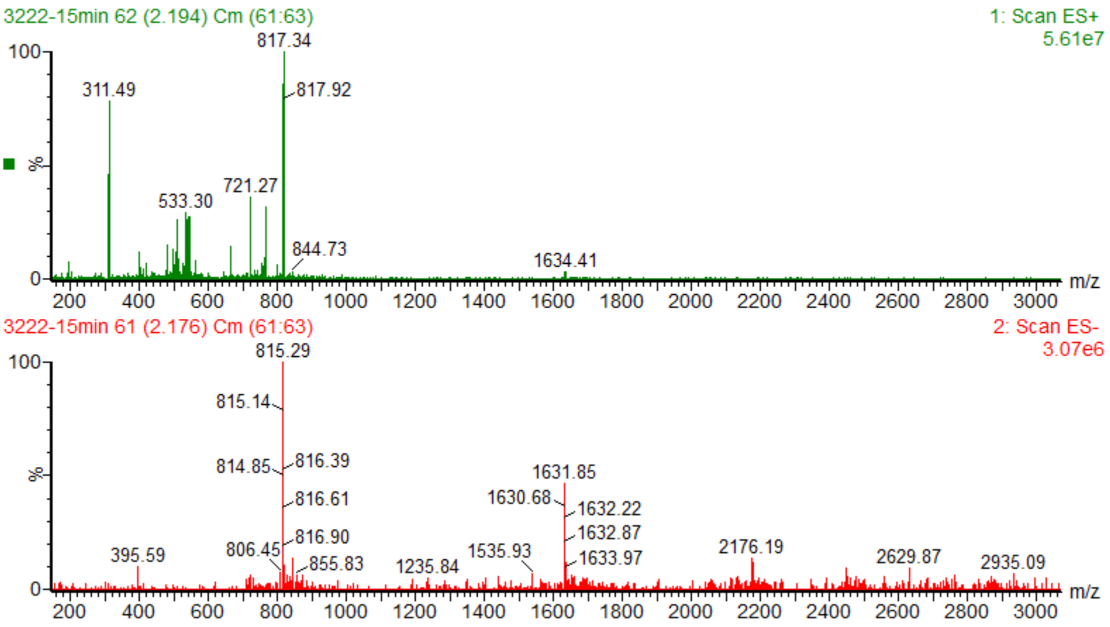

Figure S221. ESI-MS spectrum of **5d**.

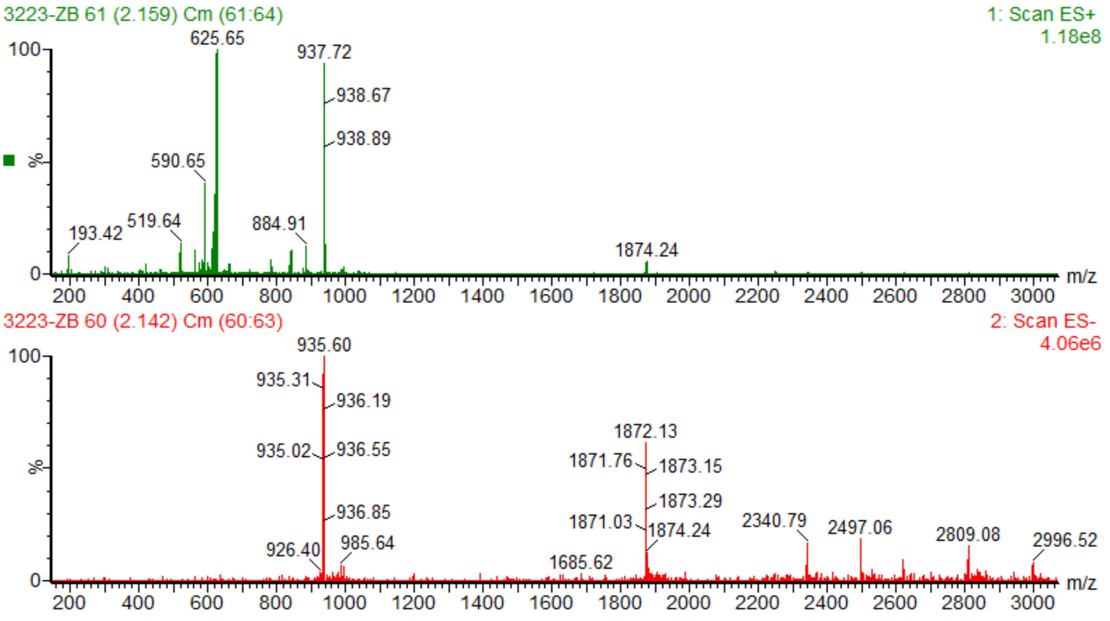

Figure S222. ESI-MS spectrum of **5e**.

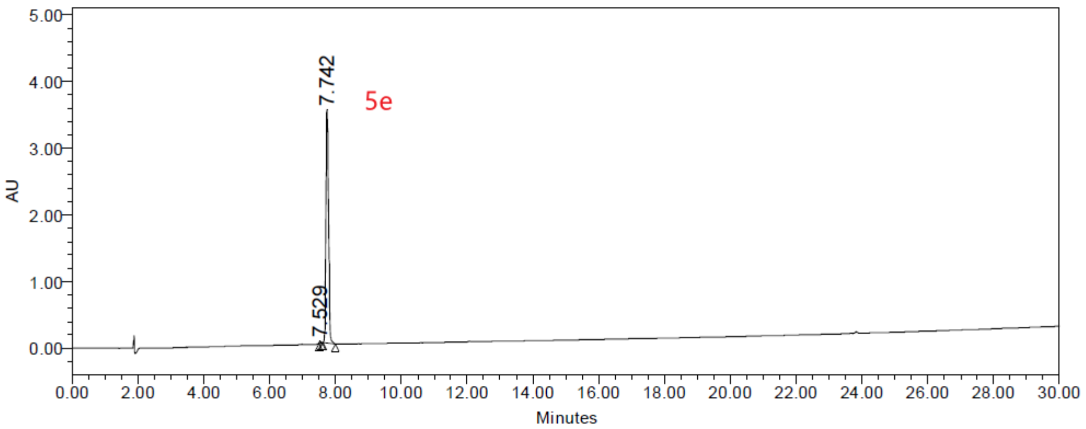

Channel: 2998 Ch1 220nm@4.8nm; Processed Channel: 2998 Ch1 220nm@4.8nm; Result Id: 2601; Processing Method: 0519

Processed Channel Descr.: 2998 Ch1 220nm@4.8nm

	Processed Channel Descr.	RT	Area	% Area	Height
1	2998 Ch1 220nm@4.8nm	7.529	83297	0.42	34647
2	2998 Ch1 220nm@4.8nm	7.742	19546671	99.58	3497168

Figure S223. HPLC-UV chromatogram at 220 nm of **5e**.

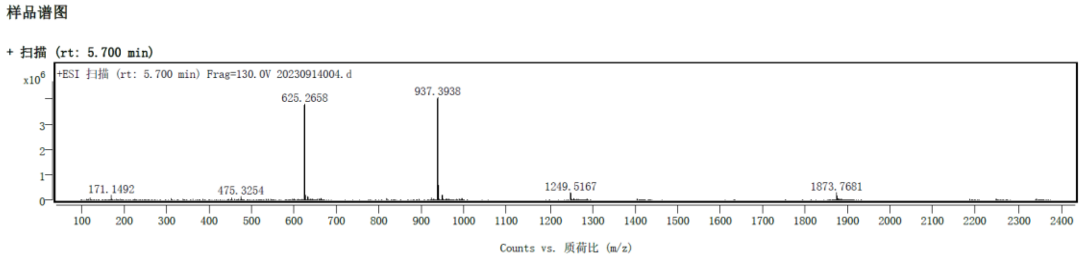

Figure S224. Q-TOF-HRMS spectrum of **5e**.

9 Bi-triazines based peptide cyclization and dimerization

9.1 Intramolecular cyclization using bi-triazines

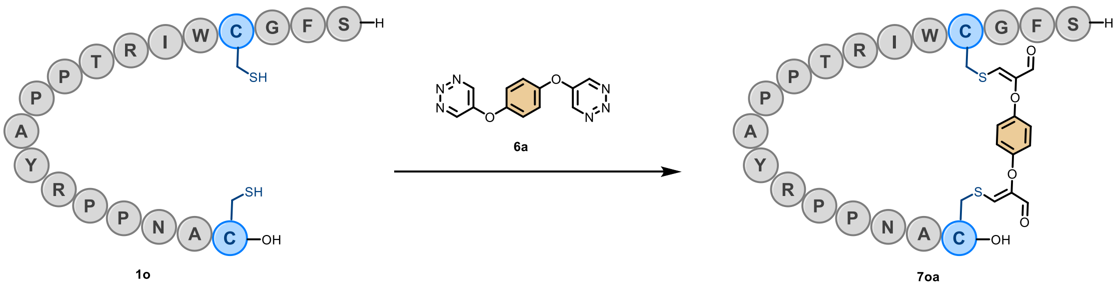

1o (5 µmol, 10 mg) was dissolved in 4.5 mL HEPES buffer (0.2 M, pH = 7.4) to reach a concentration of 1.11 mM. To this solution **6a** (10 µmol, 2.68 mg, 2 eq.) in 0.5 mL acetonitrile was added. The final concentrations in the reaction were 1 mM **1o**, 2 mM **6a**. The reaction incubated for 2 h at room temperature. The white solid **7oa** was isolated in 79.2% isolated yield (8.9 mg).

Analytical **HPLC** using Method D, RT = 10.509 min, the HPLC purity is 94.65%. **LRMS** (ESI+) *m/z*: 2251.31 $[M + H]^+$, (ESI-) *m/z*: 2249.35 $[M - H]^-$. **HRMS** (ES+) *m/z*: $[M + 2H]^{2+}$ calcd for $C_{104}H_{140}N_{26}O_{27}S_2^{+2}$ 1125.4989, found 1126.0022.

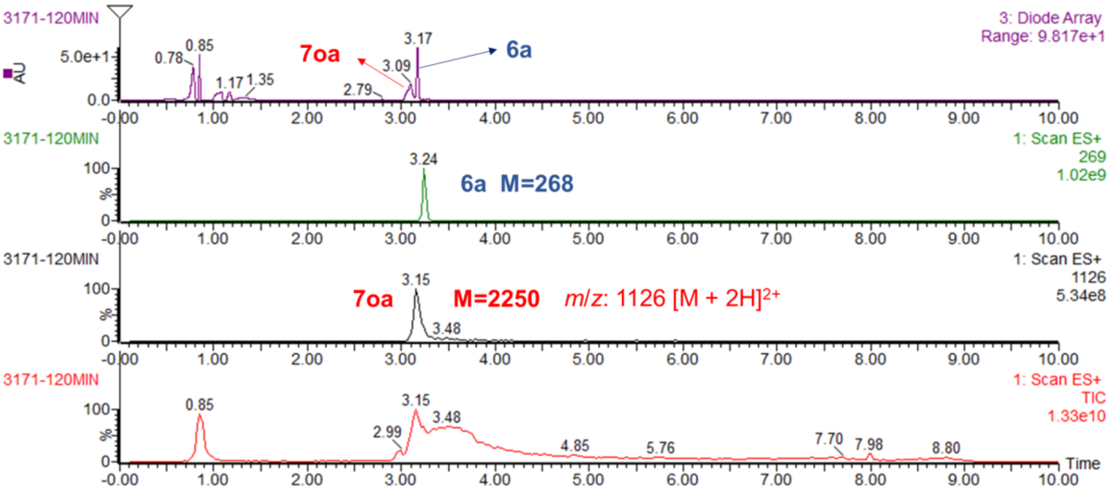

Figure S225. UPLC-MS chromatogram of reaction mixture including TIC and UV curve.

(Extract **6a** and **7oa** mass chromatograms from full scan data).

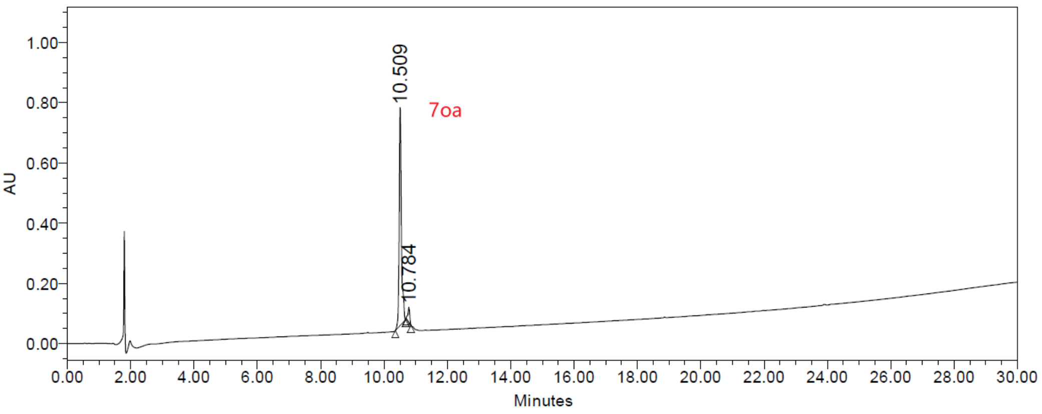

Channel: 2998 Ch1 220nm@4.8nm; Processed Channel: 2998 Ch1 220nm@4.8nm; Result Id: 2459; Processing Method: 04091

Processed Channel Descr.: 2998 Ch1 220nm@4.8nm

	Processed Channel Descr.	RT	Area	% Area	Height
1	2998 Ch1 220nm@4.8nm	10.509	3504513	94.65	724273
2	2998 Ch1 220nm@4.8nm	10.784	198122	5.35	49334

Figure S226. HPLC-UV chromatogram at 220 nm of **7oa**.

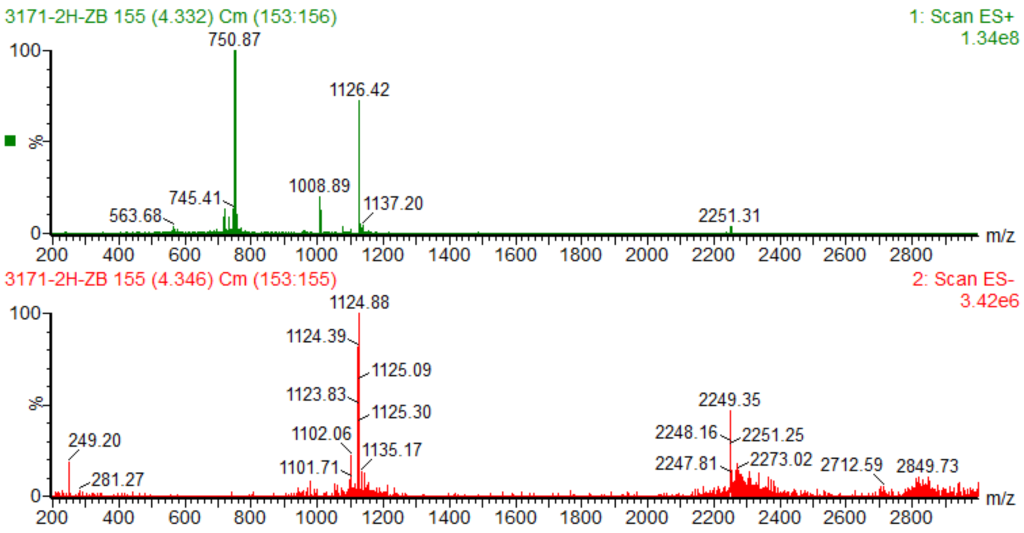

Figure S227. ESI-MS spectrum of **7oa**.

样品谱图

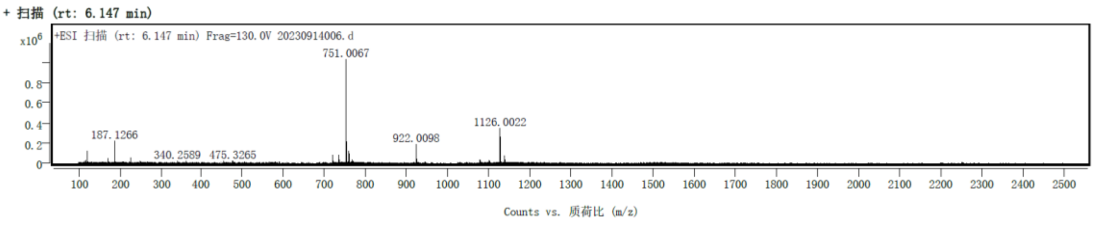

Figure S228. Q-TOF-HRMS spectrum of **7oa**.

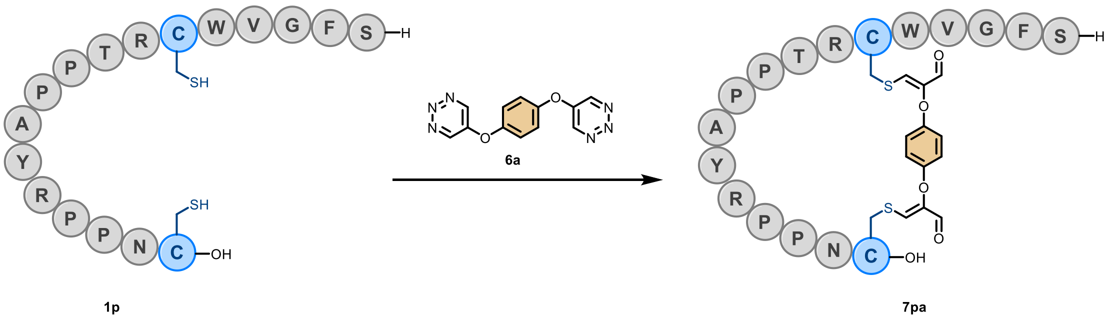

1p (10 µmol, 19.5 mg) was dissolved in 9 mL HEPES buffer (0.2 M, pH = 7.4) to reach a concentration of 1.11 mM. To this solution **6a** (20 µmol, 5.36 mg, 2 eq.) in 1 mL acetonitrile was added. The final concentrations in the reaction were 1 mM **1p**, 2 mM **6a**. The reaction incubated for 4 h at room temperature. The white solid **7pa** was isolated in 39.8% isolated yield (8.6 mg).

Analytical **HPLC** using Method D, RT = 10.300 min, the HPLC purity is 91.60%. **LRMS** (ESI+) *m/z*: 2166.33 $[M + H]^+$, (ESI-) *m/z*: 2164.44 $[M - H]^-$. **HRMS** (ES+) *m/z*: $[M + 2H]^{2+}$ calcd for $C_{100}H_{133}N_{25}O_{26}S_2^{+2}$ 1083.4742, found 1083.4761.

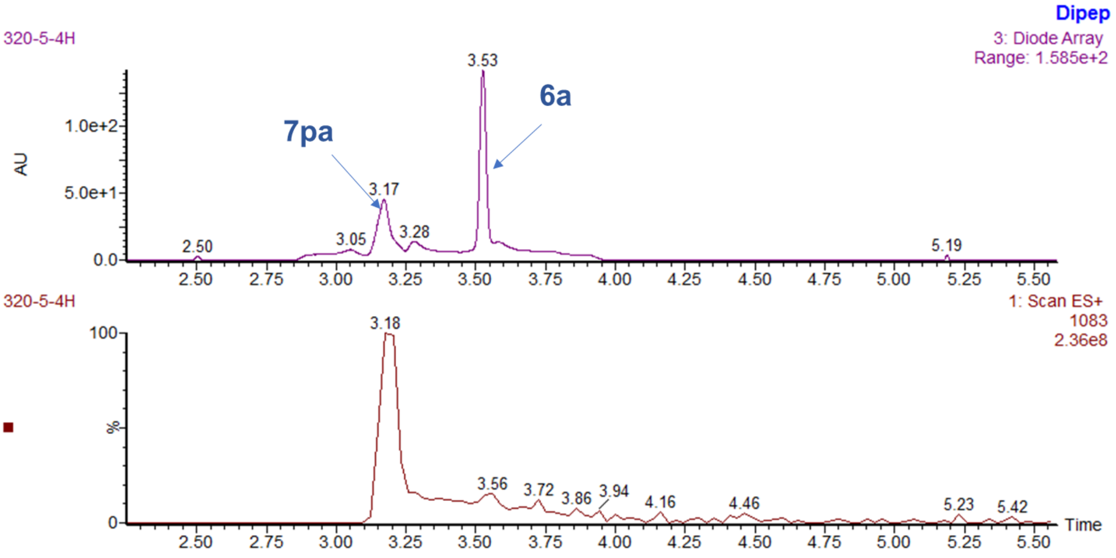

Figure S229. LCMS-UV chromatogram of the reaction system of **6a** and **1p.**

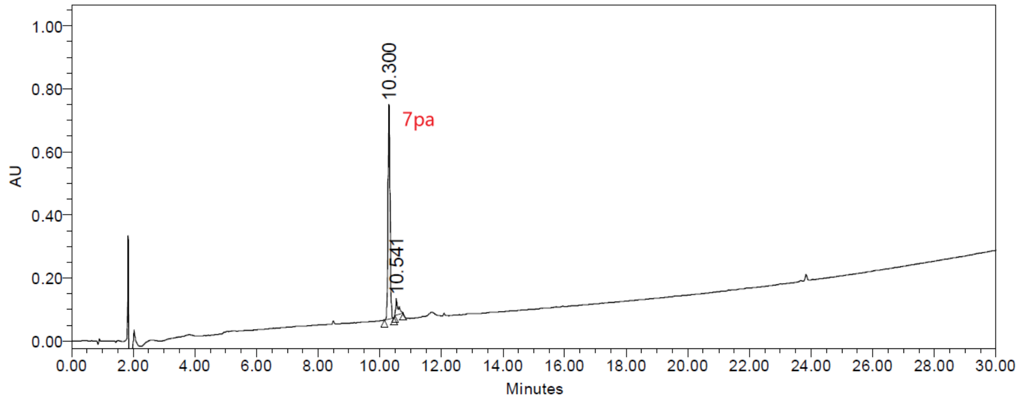

Channel: 2998 Ch1 220nm@4.8nm; Processed Channel: 2998 Ch1 220nm@4.8nm; Result Id: 2554; Processing Method: 04093

Processed Channel Descr.: 2998 Ch1 220nm@4.8nm

	Processed Channel Descr.	RT	Area	% Area	Height
1	2998 Ch1 220nm@4.8nm	10.300	3209676	91.60	681363
2	2998 Ch1 220nm@4.8nm	10.541	294148	8.40	52615

Figure S230. HPLC-UV chromatogram at 220 nm of **7pa**.

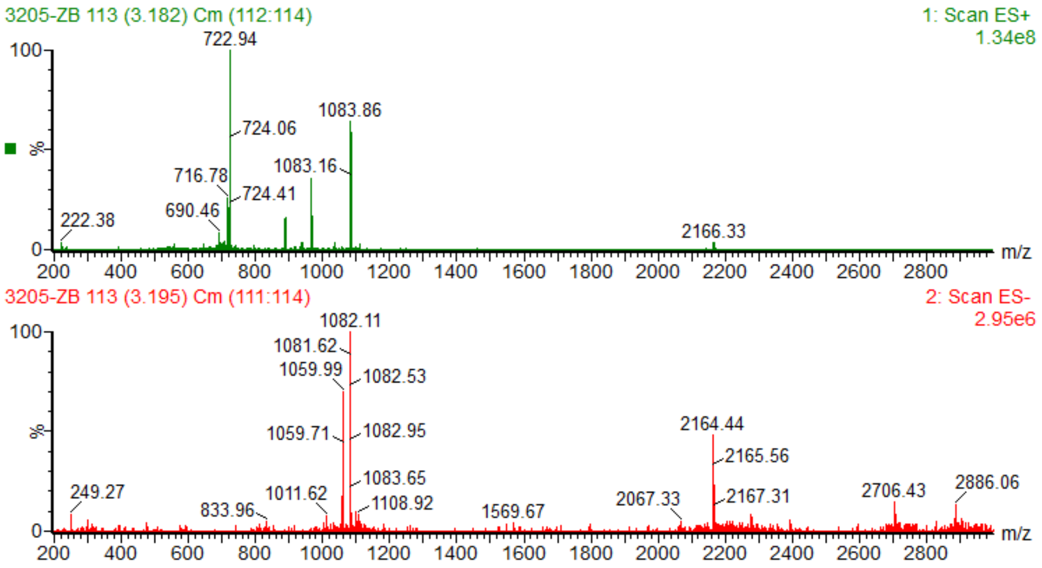

Figure S231. ESI-MS spectrum of **7pa**.

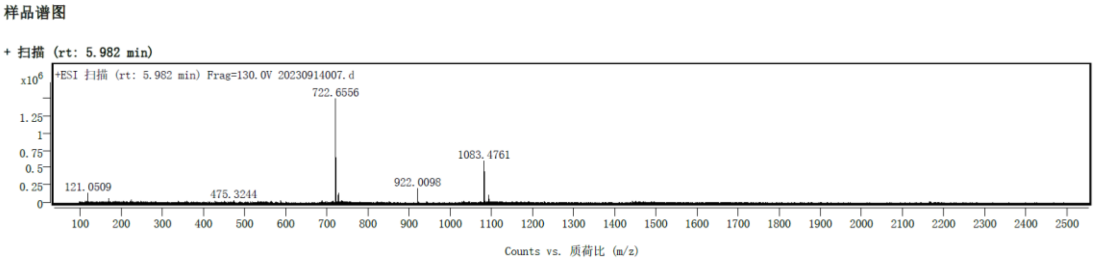

Figure S232. Q-TOF-HRMS spectrum of **7pa**.

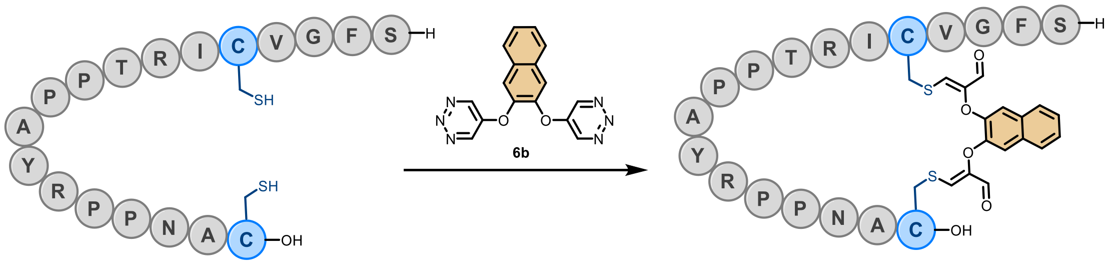

1m (4.1 µmol, 8 mg) was dissolved in 3.6 mL HEPES buffer (0.2 M, pH = 7.4) to reach a concentration of 1.1 mM. To this solution **6b** (8.2 µmol, 2.6 mg, 2 eq.) in 0.5 mL acetonitrile was added. The final concentrations in the reaction were 1 mM **1m**, 2 mM **6b**. The reaction incubated for 4 h at room temperature. The white solid **7mb** was isolated in 70.4% isolated yield (6.4 mg).

Analytical **HPLC** using Method D, RT = 10.943 min, the HPLC purity is 95.17%. **LRMS** (ESI+) *m/z*: 2214.28 $[M + H]^+$, (ESI-) *m/z*: 2212.46 $[M - H]^-$. **HRMS** (ES+) *m/z*: $[M + H]^+$ calcd for $C_{102}H_{141}N_{25}O_{27}S_2^+$ 2213.9982, found 2213.9967.

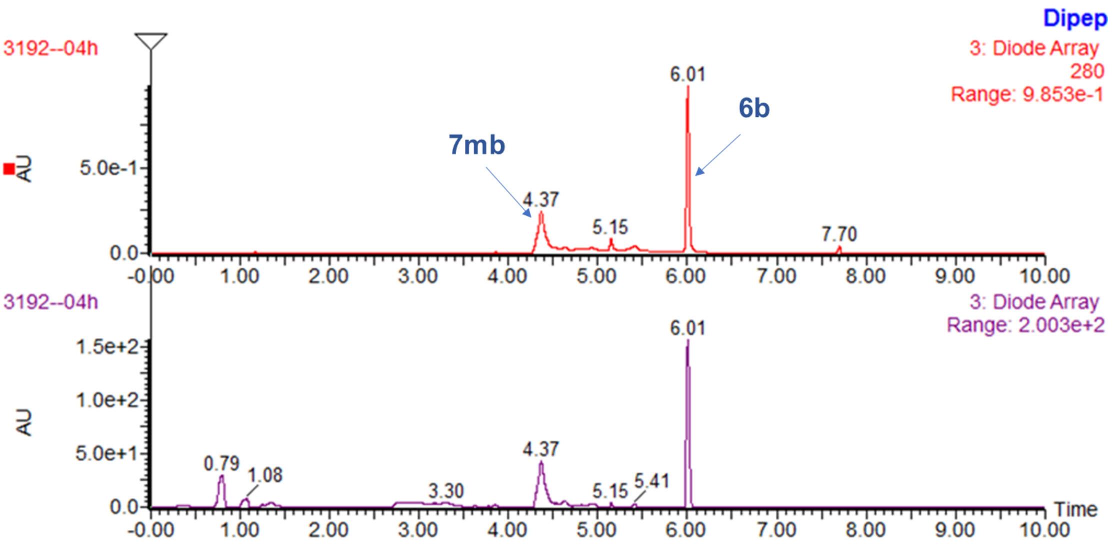

Figure S233. LCMS-UV chromatogram of the reaction system of **6b** and **1m.**

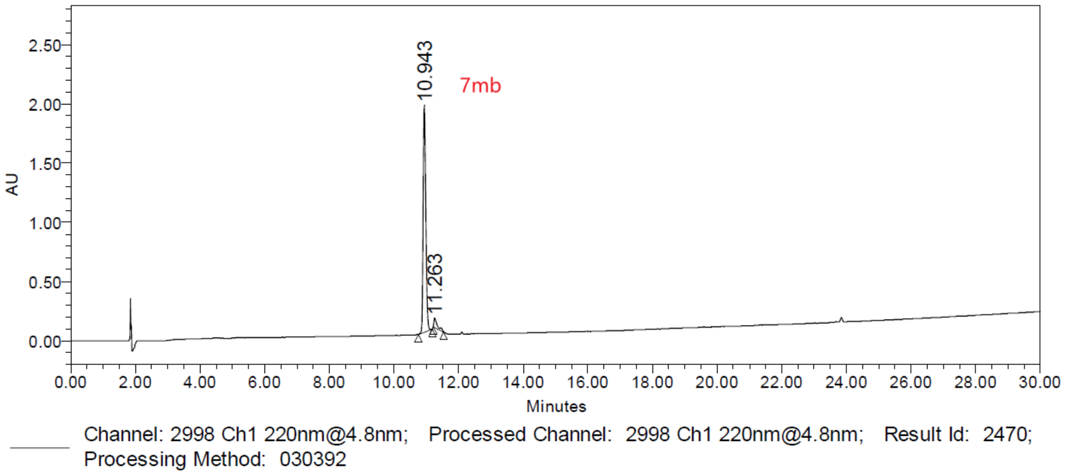

Processed Channel Descr.: 2998 Ch1 220nm@4.8nm

	Processed Channel Descr.	RT	Area	% Area	Height
1	2998 Ch1 220nm@4.8nm	10.943	10890986	95.17	1914919
2	2998 Ch1 220nm@4.8nm	11.263	552820	4.83	78631

Figure S234. HPLC-UV chromatogram at 220 nm of **7mb**.

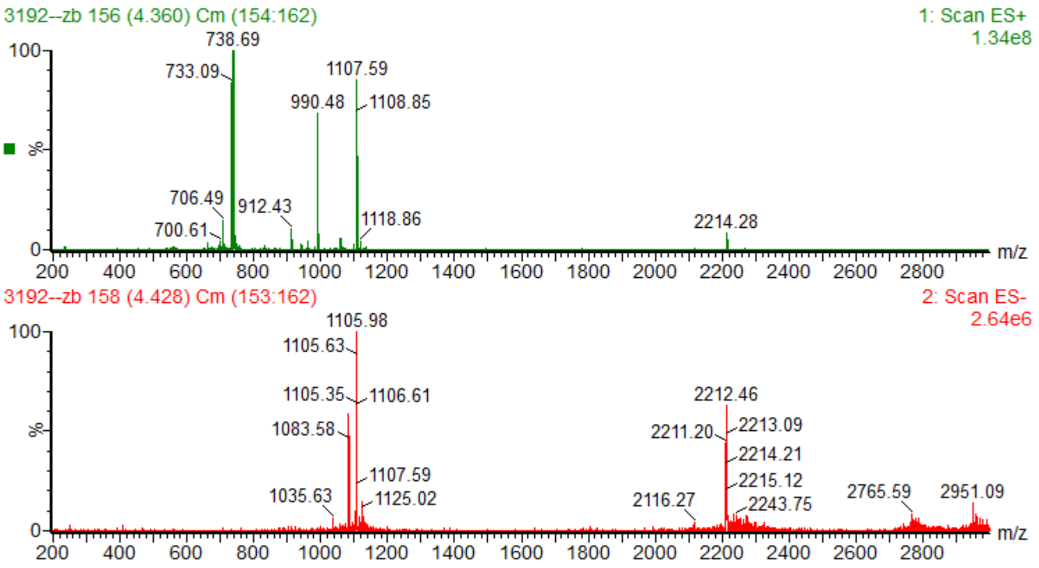

Figure S235. ESI-MS spectrum of **7mb**.

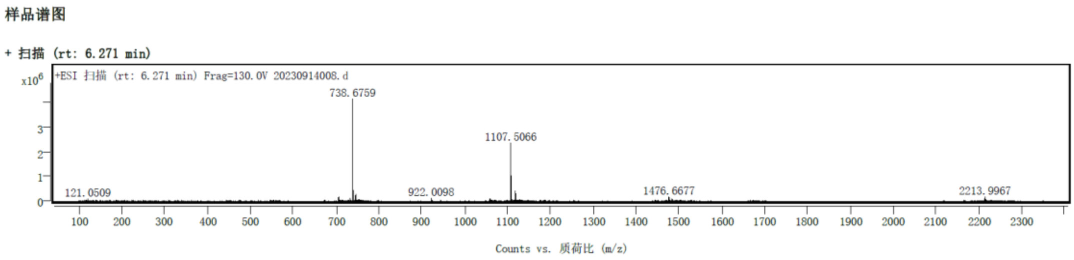

Figure S236. Q-TOF-HRMS spectrum of **7mb**.

9.2 Intermolecular coupling between peptides by bi-triazines

General procedure C :

The dried peptide (1 equiv.) was dissolved in appropriate amount of HEPES buffer (0.2 M, pH = 7.4) to reach a concentration of 27.78 mM. To this solution bi-triazine (0.5 eq.) in acetonitrile (1/9 of the volume of buffer) was added. The final concentrations in the reaction were 25 mM peptide, 12.5 mM bi-triazine. The reaction incubated for 2 – 12 h at room temperature and the reaction process was detected by LCMS. The solvent was purification by preparative RP-HPLC and to give the product by freeze drying.

Here, **1b** reacted with **6b** to obtain **8bb** as a representative example to illustrate the general procedure C.

1b (0.06 mmol, 32.7 mg, 1 equiv.) was dissolved in HEPES buffer (0.2 M, pH = 7.4, 2.16 mL) to reach a concentration of 27.78 mM. To this solution **6b** (0.03 mmol, 9.7 mg, 0.5 equiv.) dissolved in acetonitrile (0.24 mL) was added. The final concentrations in the reaction were 25 mM **1b**, 12.5 mM **6b** and 10% acetonitrile. The reaction incubated for 2 h at room temperature and the reaction process was detected by LCMS. The solvent was purification by preparative RP-HPLC and to give the white solid **8bb** (21.4 mg, 54.8% yield) by freeze drying.

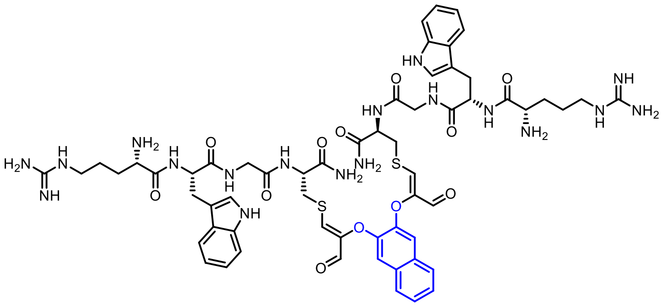

8bb

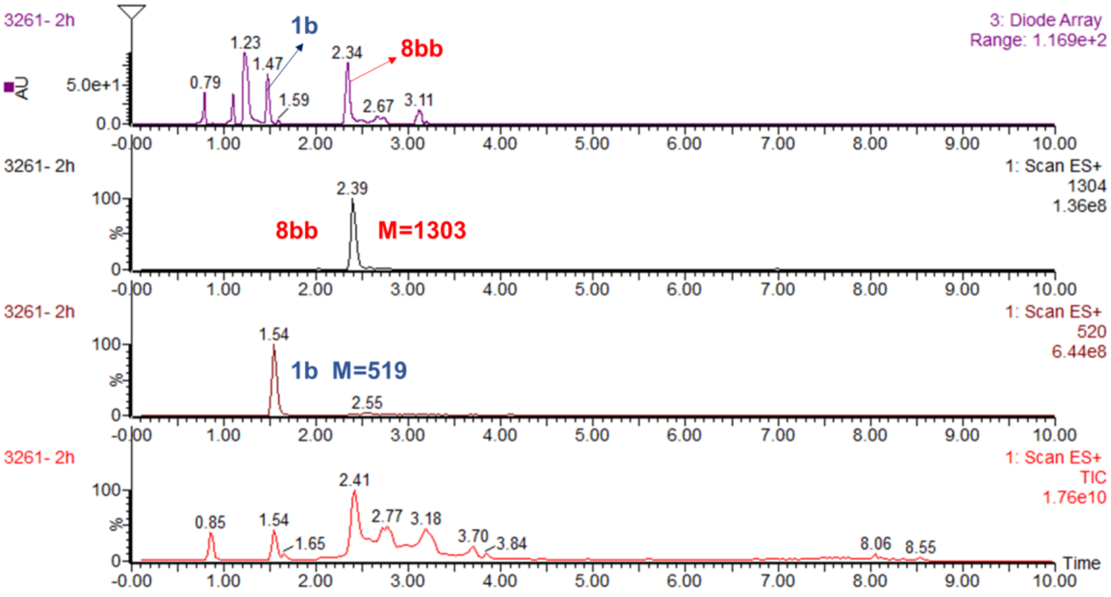

Figure S237. UPLC-MS chromatogram of reaction mixture including TIC and UV curve.

(Extract **1b** and **8bb** mass chromatograms from full scan data).

Analytical **HPLC** using Method D, RT = 9.019 min, the HPLC purity is 95.20%. **LRMS** (ESI+) *m/z*: 1303.87 [M + H]$^+$, (ESI-) *m/z*: 1301.84 [M - H]$^-$. **HRMS** (ES+) *m/z*: [M + H]$^+$ calcd for $C_{60}H_{74}N_{18}O_{12}S_2^+$ 1303.5253, found 1303.5237.

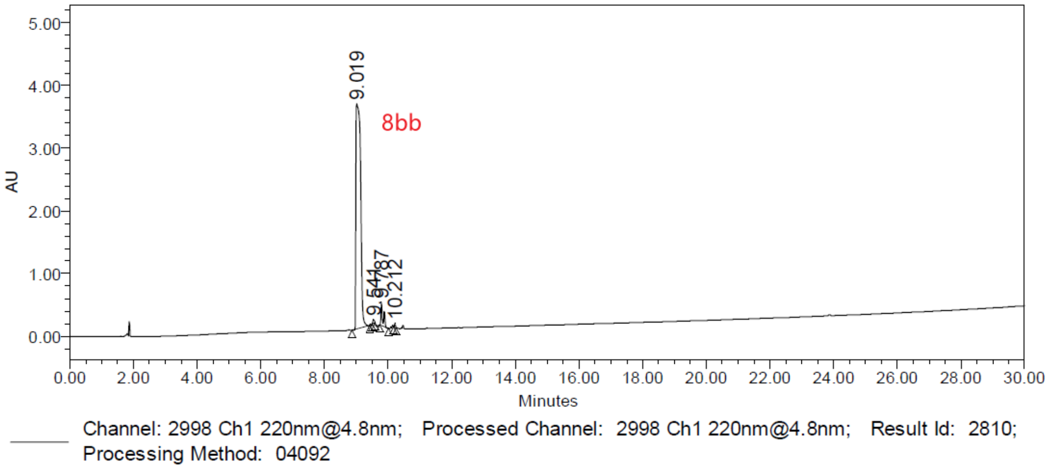

Processed Channel Descr.: 2998 Ch1 220nm@4.8nm

	Processed Channel Descr.	RT	Area	% Area	Height
1	2998 Ch1 220nm@4.8nm	9.019	36615332	95.20	3582599
2	2998 Ch1 220nm@4.8nm	9.541	196526	0.51	58260
3	2998 Ch1 220nm@4.8nm	9.787	1504605	3.91	362222
4	2998 Ch1 220nm@4.8nm	10.212	146700	0.38	69192

Figure S238. HPLC-UV chromatogram at 220 nm of **8bb**.

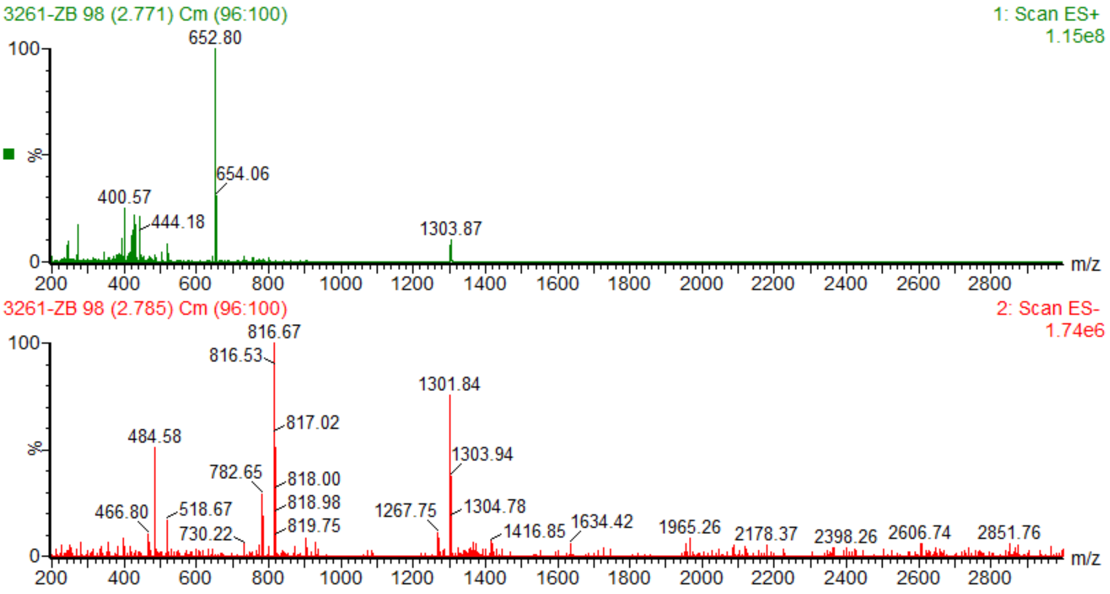

Figure S239. ESI-MS spectrum of **8bb**.

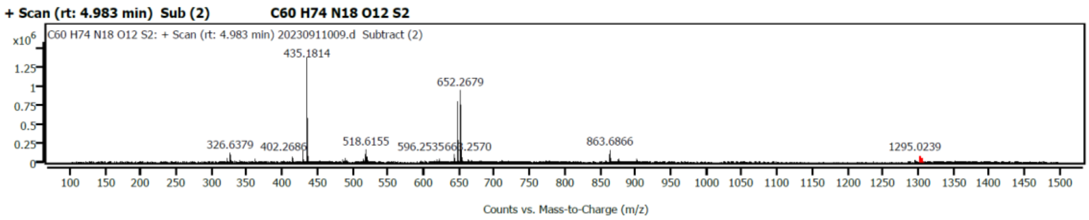

Figure S240. Q-TOF-HRMS spectrum of **8bb**.

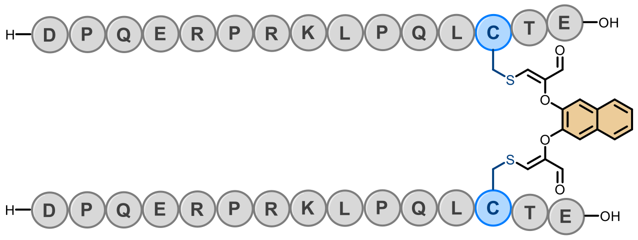

8qb

According to the general procedure C, **1q** (10 μmol, 18.1 mg) reacted with **6b** (5 μmol, 1.6 mg) for 12 h to afford the white solid **8qb** (7.8 mg) in 40.2% isolated yield.

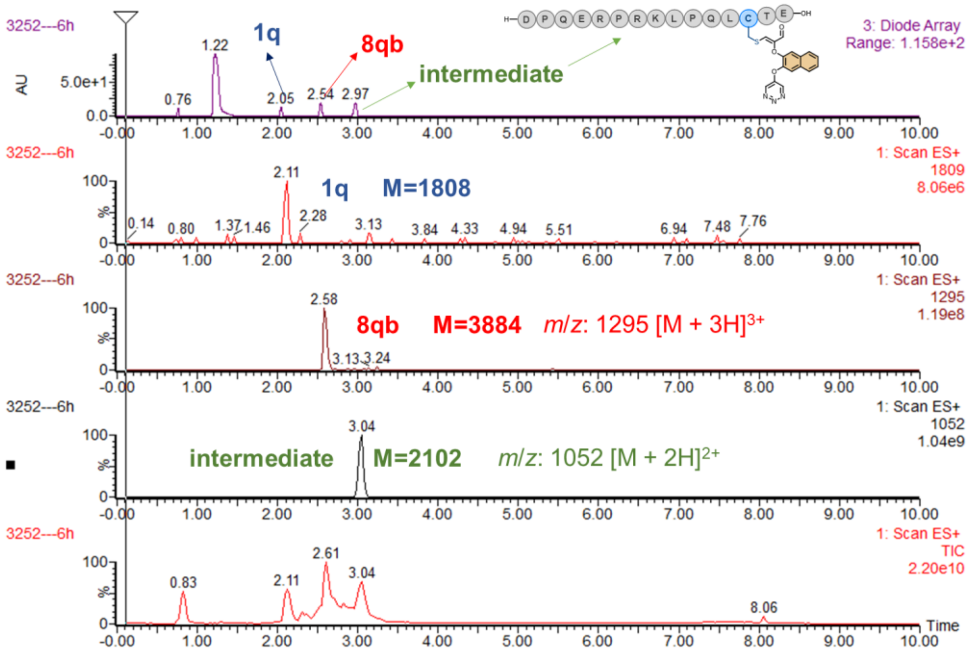

Figure S241. UPLC-MS chromatogram of reaction mixture that reacted for 6 hours including TIC and UV curve. (Extract **1q, 8qb** and their intermediate mass chromatograms from full scan data).

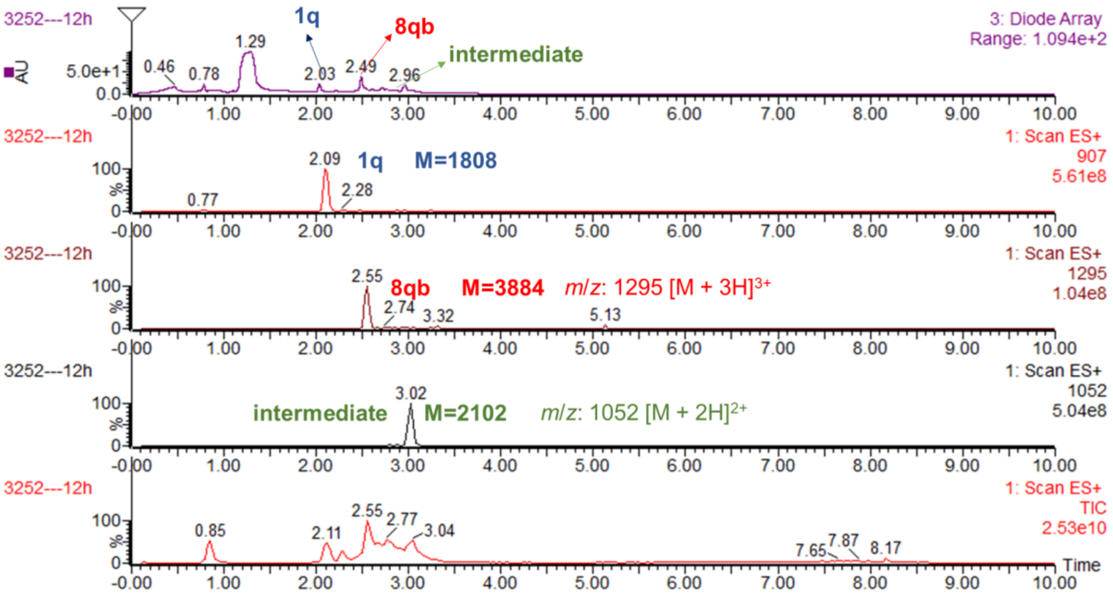

Figure S242. UPLC-MS chromatogram of reaction mixture that reacted for 12 hours including TIC and UV curve. (Extract **1q, 8qb** and their intermediate mass chromatograms from full scan data).

Analytical **HPLC** using Method D, RT = 8.556 min, the HPLC purity is 100%. **LRMS** (ESI+) *m/z*: 1941.74 $[M + 2H]^{2+}$, (ESI-) *m/z*: 972.35 $[M - 4H]^{4-}$. **HRMS** (*MALDI-TOF*) *m/z*: calcd for $C_{168}H_{264}N_{48}O_{54}S_2$ 3884.3620 (molecular weight), found 3883.8318.

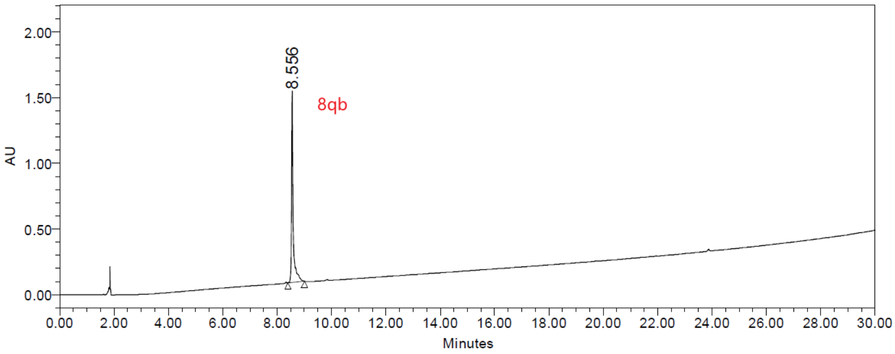

Channel: 2998 Ch1 220nm@4.8nm; Processed Channel: 2998 Ch1 220nm@4.8nm; Result Id: 2831;
Processing Method: 030392

Processed Channel Descr.: 2998 Ch1 220nm@4.8nm

	Processed Channel Descr.	RT	Area	% Area	Height
1	2998 Ch1 220nm@4.8nm	8.556	5935575	100.00	1456475

Figure S243. HPLC-UV chromatogram at 220 nm of **8qb**.

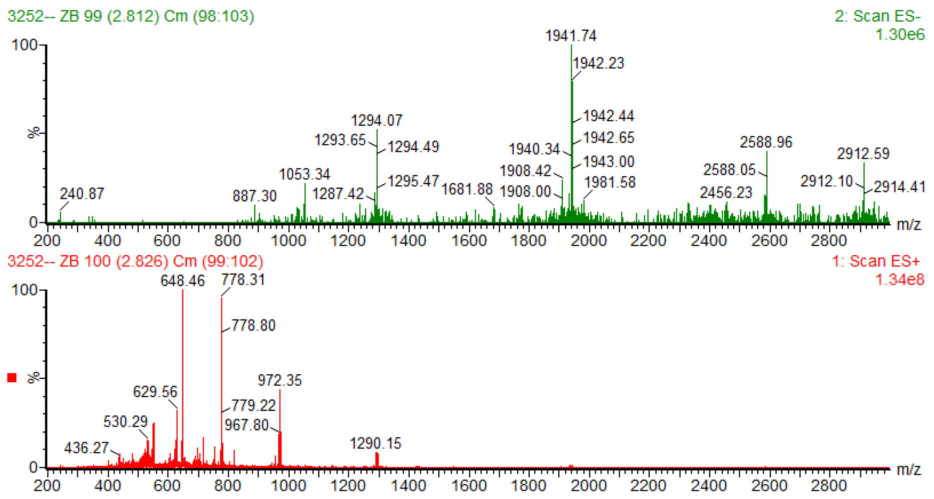

Figure S244. ESI-MS spectrum of **8qb**.

9.3 Heterologous dimerization using bi-triazines

General procedure D:

Dissolve the peptide (**1d**) in the HEPES buffer (0.2 M, pH = 7.4) to reach a concentration of 27.78 mM, and add it in batches to the **6a** (1 equiv) solution in DMF (1/9 of volume of the buffer) within 1 h. Then, add the other payload (**1a** or maytansine, 1.1 equiv) and the reaction incubated at room temperature for 1 h. The reaction process was detected by LCMS. The solvent was purification by preparative RP-HPLC to give the product followed by freeze drying.

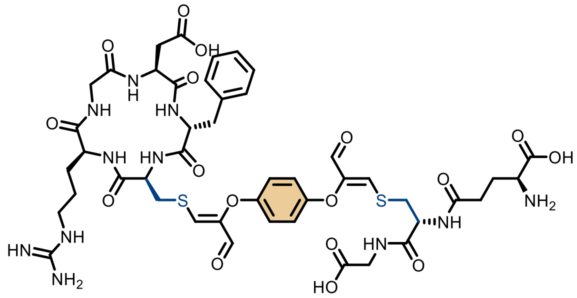

9a

According to the general procedure D, **1d** (0.034 mmol, 20 mg) and **1a** (0.037 mmol, 11.5 mg) reacted with **6a** (0.034 mmol, 9.1 mg) to afford the white solid **9a** (13.4 mg) in 35.8% isolated yield.

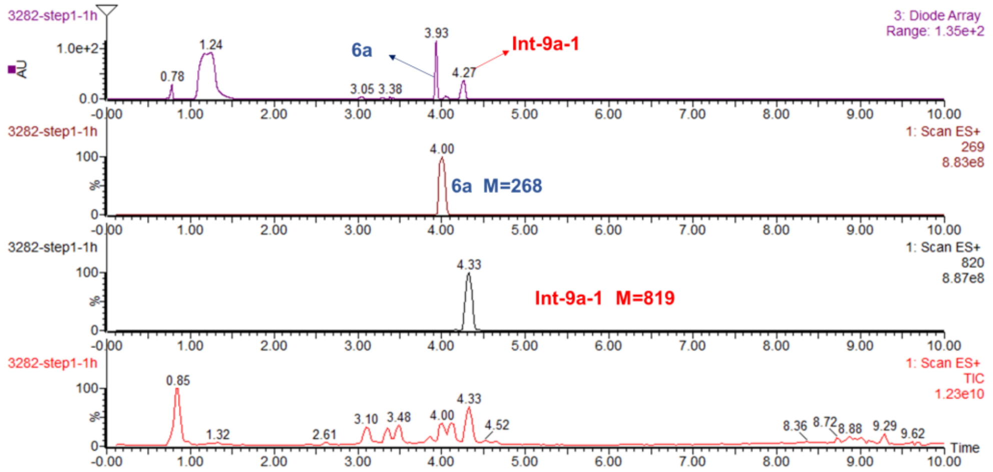

Figure S245. UPLC-MS chromatogram of reaction mixture of step 1 including TIC and UV curve.

(Extract **6a** and **Int-9a-1** mass chromatograms from full scan data).

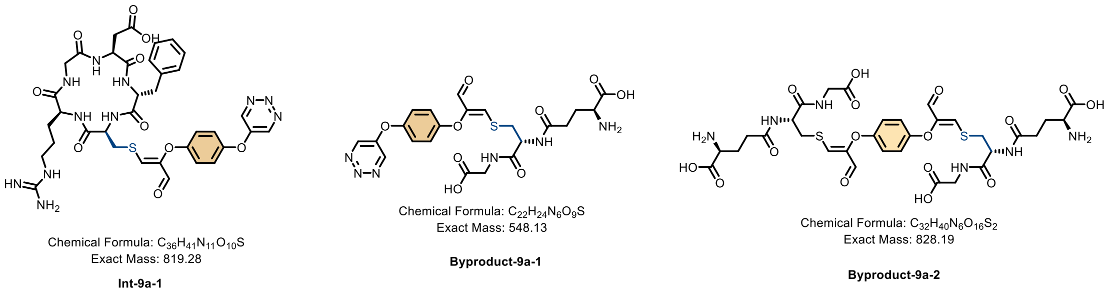

Figure S246. Intermediates and by-products that occur during the reaction process.

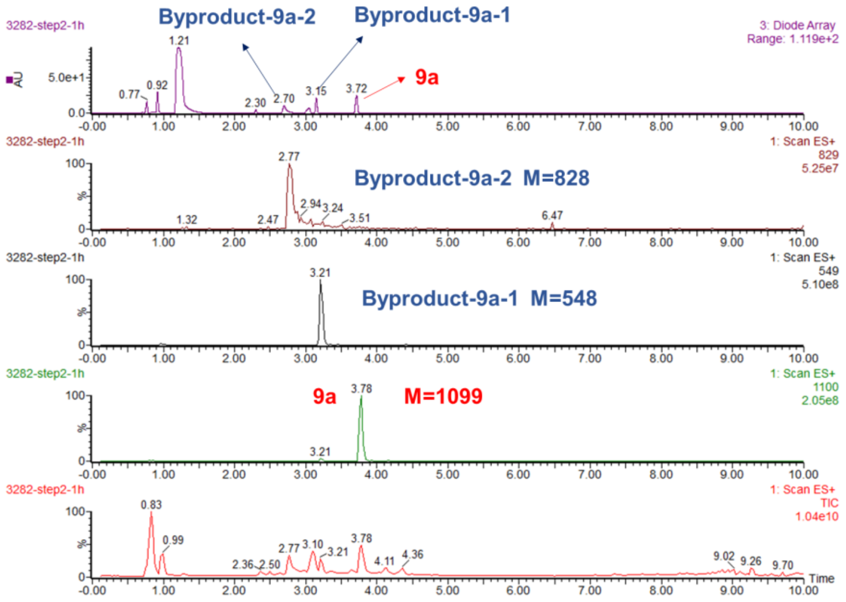

Figure S247. UPLC-MS chromatogram of reaction mixture of step 2 including TIC and UV curve.

(Extract **9a, byproduct-9a-1** and **byproduct-9a-2** mass chromatograms from full scan data).

Analytical **HPLC** using Method D, RT = 9.132 min, the HPLC purity is 100%. **LRMS** (ESI+) *m/z*: 1100.52 $[M + H]^+$, (ESI-) *m/z*: 1098.70 $[M - H]^-$. **HRMS** (ES+) *m/z*: $[M + H]^+$ calcd for $C_{46}H_{57}N_{11}O_{17}S_2^+$ 1100.3453, found 1100.3430.

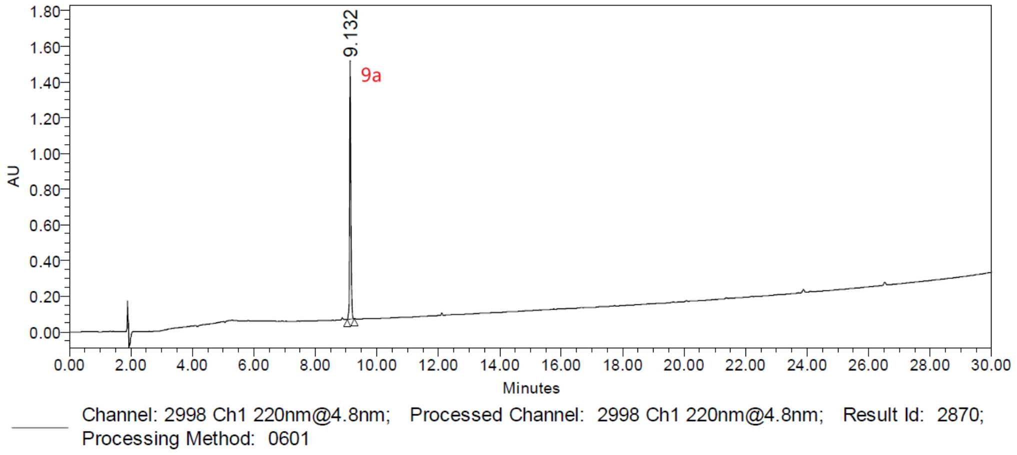

Processed Channel Descr.: 2998 Ch1 220nm@4.8nm

	Processed Channel Descr.	RT	Area	% Area	Height
1	2998 Ch1 220nm@4.8nm	9.132	4020420	100.00	1450083

Figure S248. HPLC-UV chromatogram at 220 nm of **9a**.

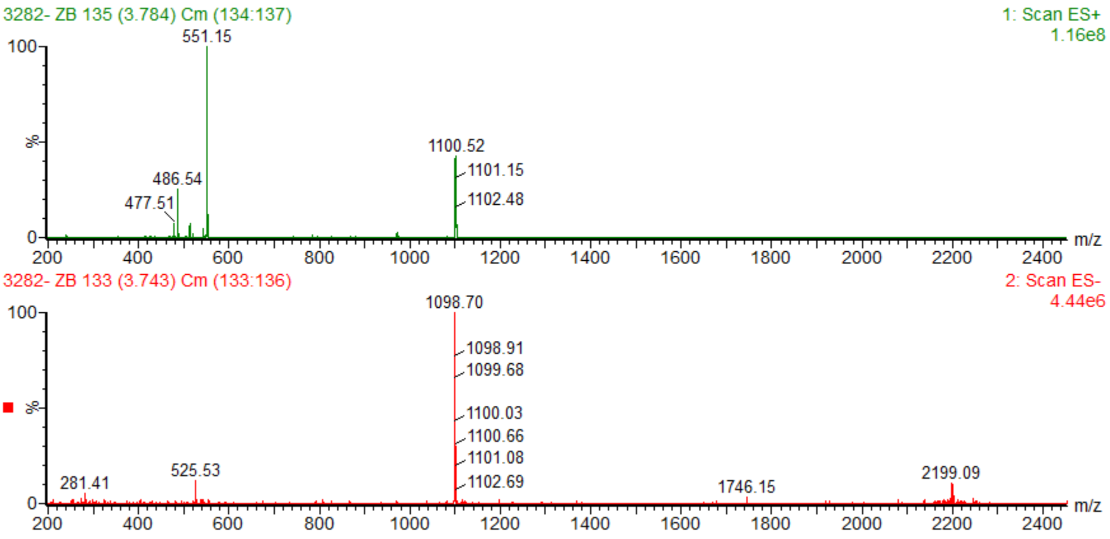

Figure S249. ESI-MS spectrum of **9a**.

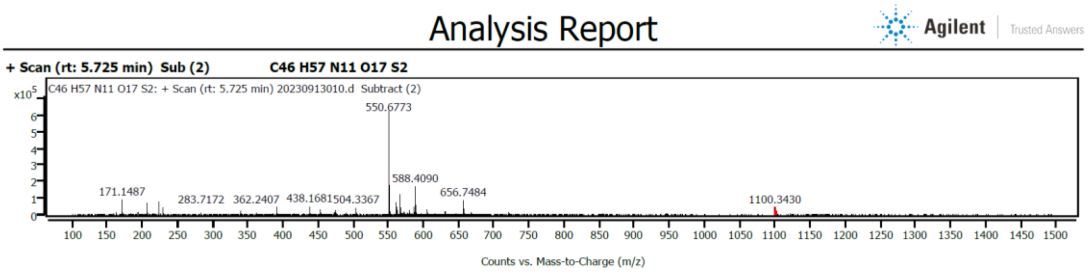

Spectrum Identification Table

Best	ID Source	Name	Formula	Species	m/z	Diff (ppm)	CAS	Score	Score (Lib)	Score (DB)	Score (MFG)	Lib/DB
Yes	MFG		C46 H57 N11 O17 S2	(M+H)+	1100.3430	-1.79		95.85			95.85	

Figure S250. Q-TOF-HRMS spectrum of **9a**.

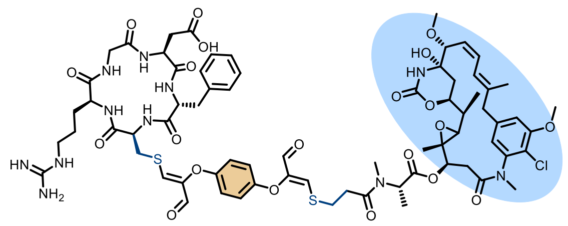

9b

According to the general procedure D, **1d** (0.052 mmol, 30 mg) and maytansine (0.057 mmol, 42.2 mg) reacted with **6a** (0.052 mmol, 13.9 mg) to afford the white solid **9b** (13.0 mg) in 16.4% isolated yield.

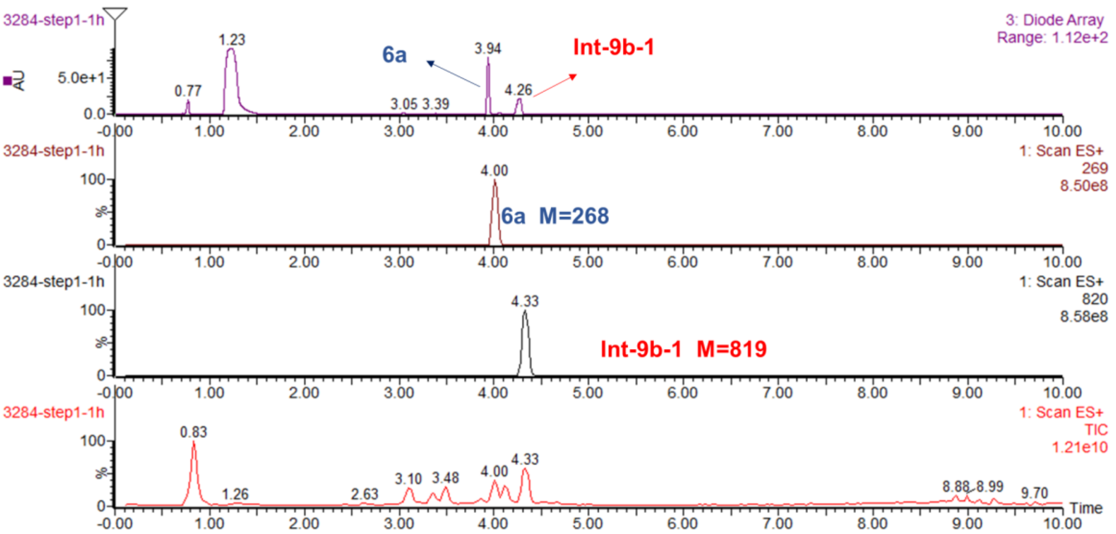

Figure S251. UPLC-MS chromatogram of reaction mixture of step 1 including TIC and UV curve.

(Extract **6a** and **Int-9b-1** mass chromatograms from full scan data).

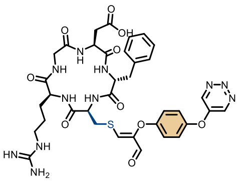

Chemical Formula: $C_{36}H_{41}N_{11}O_{10}S$
Exact Mass: 819.28

Int-9b-1

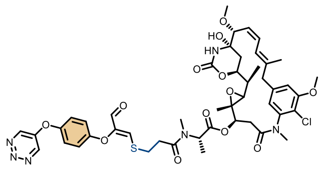

Chemical Formula: $C_{47}H_{55}ClN_6O_{13}S$
Exact Mass: 978.32

Byproduct-9b-1

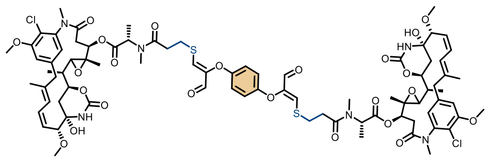

Chemical Formula: $C_{82}H_{102}Cl_2N_6O_{24}S_2$
Exact Mass: 1688.58

Byproduct-9b-2

Figure S252. Intermediates and by-products that occur during the reaction process.

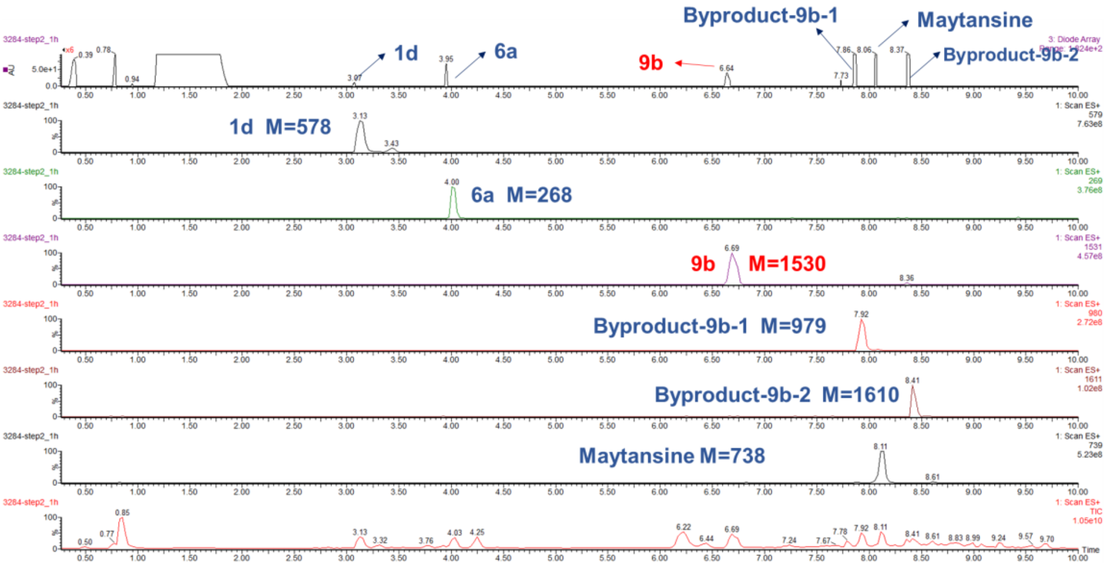

Figure S253. UPLC-MS chromatogram of reaction mixture of step 2 including TIC and UV curve. (Extract **6a, 1d, 9b, maytansine, byproduct-9b-1** and **byproduct-9b-2** mass chromatograms from full scan data).

Analytical **HPLC** using Method D, RT = 15.419 min, the HPLC purity is 100%. **LRMS** (ESI+) *m/z*: 1530.75 [M + H]$^+$, (ESI-) *m/z*: 1528.86 [M - H]$^-$. **HRMS** (ES+) *m/z*: [M + H]$^+$ calcd for $C_{71}H_{88}ClN_{11}O_{21}S_2^+$ 1530.5364, found 1530.5363.

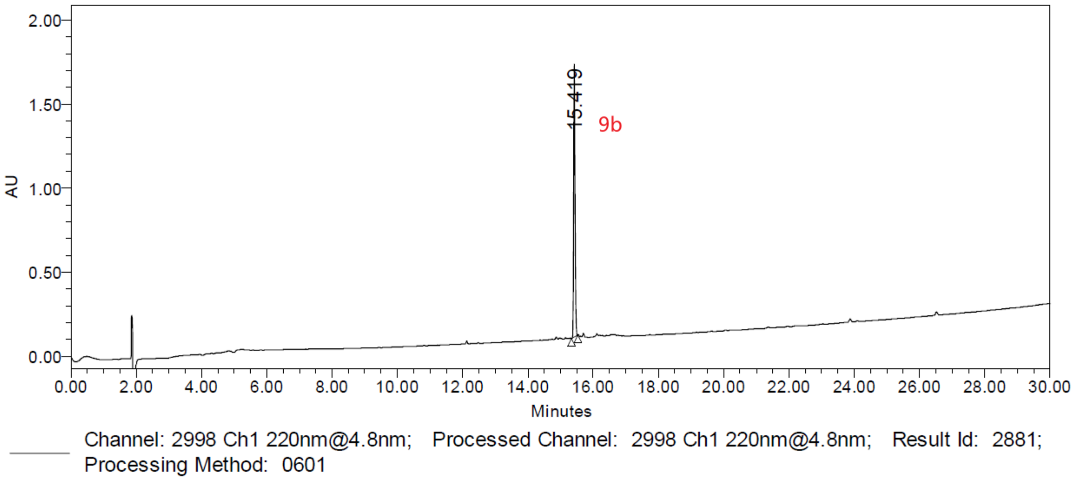

Processed Channel Descr.: 2998 Ch1 220nm@4.8nm

	Processed Channel Descr.	RT	Area	% Area	Height
1	2998 Ch1 220nm@4.8nm	15.419	4623006	100.00	1622186

Figure S254. HPLC-UV chromatogram at 220 nm of **9b**.

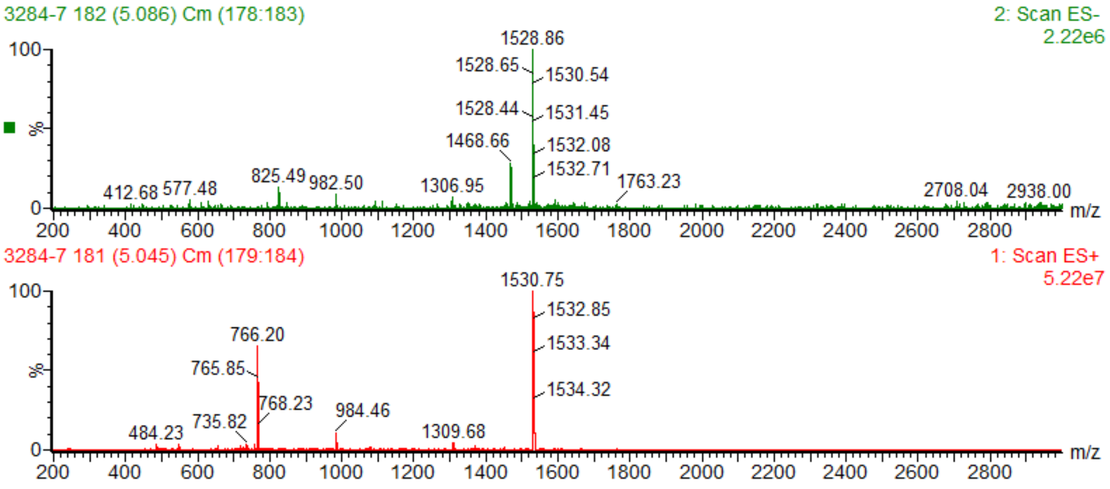

Figure S255. ESI-MS spectrum of **9b**.

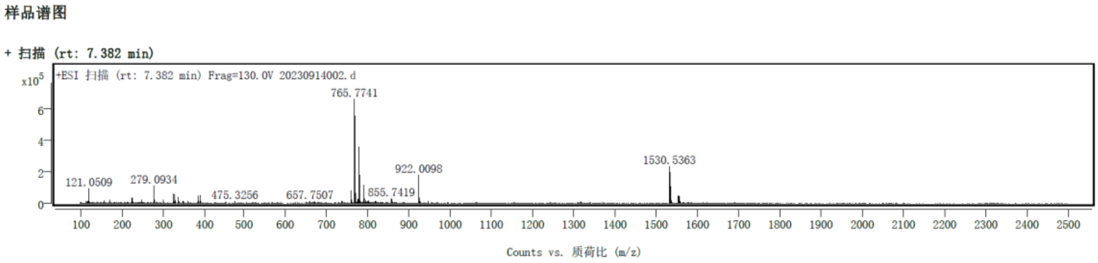

Figure S256. Q-TOF-HRMS spectrum of **9b**.

10 Stability study of the peptide conjugates

Peptide conjugate **4db** (10 µmol, 7.54 mg) was pre-dissolved in water (10 mL, 10% v/v CH_3CN) to afford the 1 mM stock solution. Hydrogen peroxide (30%, 10.22 µL) were dissolved in PBS buffer (0.2 M, pH = 7.4) to reach a 10 mL volume and to afford 10 mM H_2O_2 solutions. GSH (1 mmol, 308 mg) was dissolved in PBS buffer (0.2 M, pH = 7.4, 5 mL) to afford external thiol test solutions at a concentration of 200 mM.

Neutral conditions (pH 7.4)

The stock solution of **4db** and PBS buffer (0.5 M, pH = 7.4) were mixed at 1:1 to afford the 0.5 mM peptide solutions. Then these samples left at room temperature and analyzed by HPLC at corresponding time.

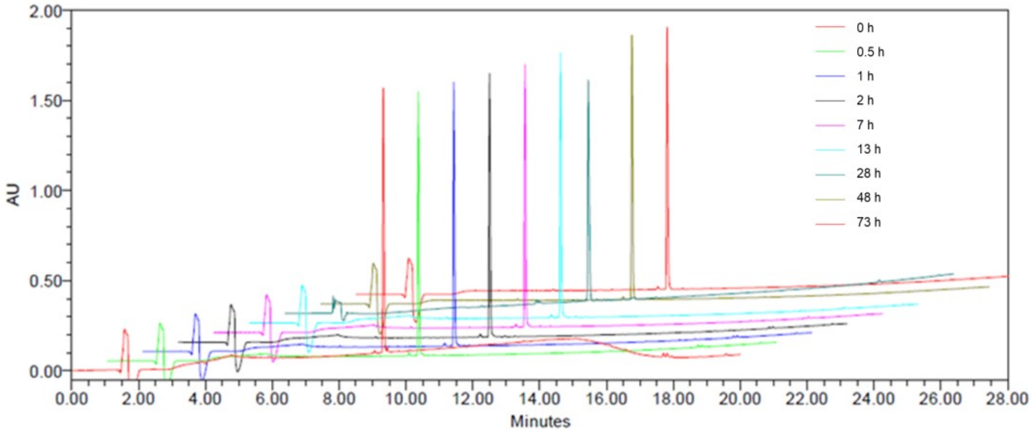

Figure S257. Overlay of HPLC-UV chromatogram at 220 nm of **4db** with neutral condition (pH = 7.4).

Acidic conditions (pH 3.0, 5.0)

pH = 3.0

1 mL of the stock solution of **4db** and 1 mL citrate buffer (0.5 M, pH = 3) were mixed to afford the 0.5 mM peptide solutions. Then these samples left at room temperature and analyzed by HPLC at corresponding time.

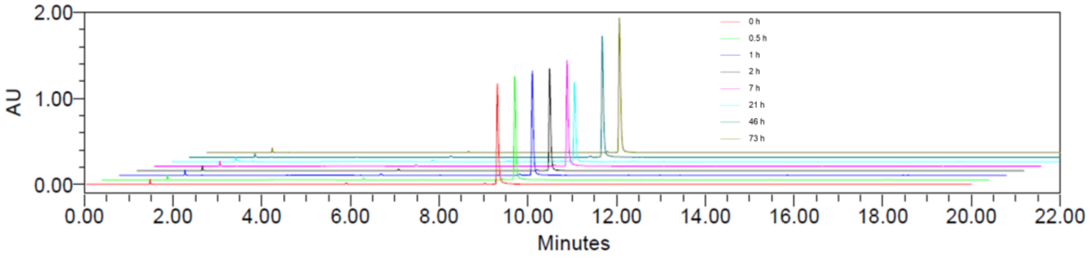

Figure S258. Overlay of HPLC-UV chromatogram at 280 nm of **4db** with acidic condition (pH = 3.0).

pH = 5.0

1 mL of the stock solution of **4db** and 1 mL citrate buffer (0.5 M, pH = 5) were mixed to afford the 0.5 mM peptide solutions. Then these samples left at room temperature and analyzed by HPLC at corresponding time.

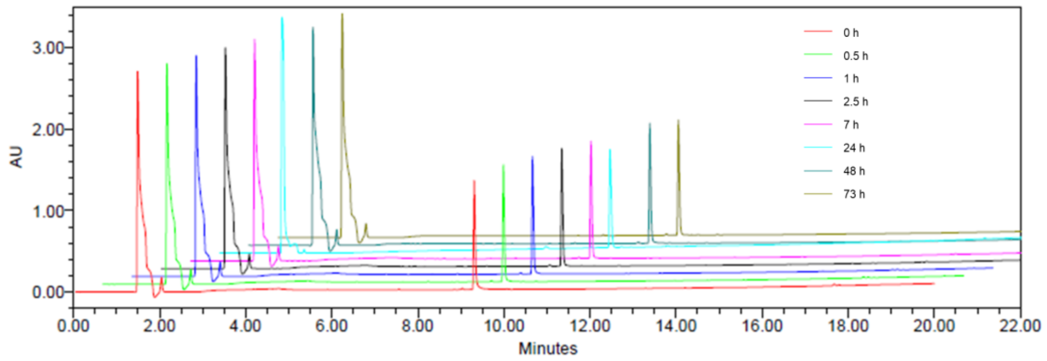

Figure S259. Overlay of HPLC-UV chromatogram at 220 nm of **4db** with acidic condition (pH = 5.0).

Basic conditions (pH 9.0, 11, 12.5)

pH = 9.0

1 mL of the stock solution of **4db** and 1 mL PBS buffer (0.5 M, pH = 9) were mixed to afford the 0.5 mM peptide solutions. Then these samples left at room temperature and analyzed by HPLC at corresponding time.

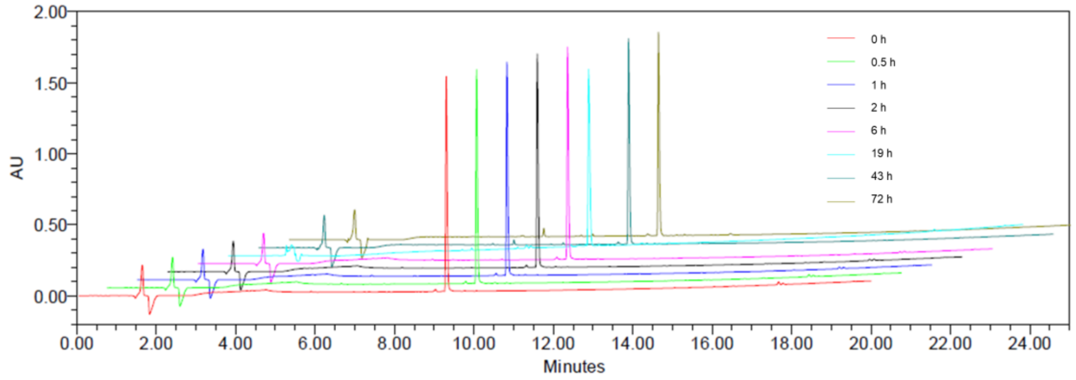

Figure S260. Overlay of HPLC-UV chromatogram at 220 nm of **4db** with basic condition (pH = 9.0).

pH = 11

1 mL of the stock solution of **4db** and 1 mL CAPS buffer (0.5 M, pH = 11) were mixed to afford the 0.5 mM peptide solutions. Then these samples left at room temperature and analyzed by HPLC at corresponding time.

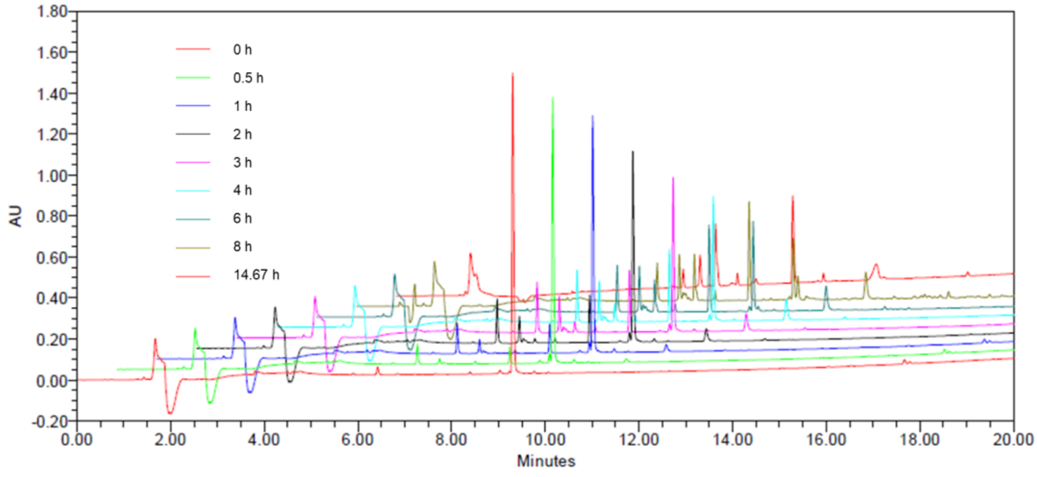

Figure S261. Overlay of HPLC-UV chromatogram at 220 nm of **4db** with basic condition (pH = 11.0).

pH = 12.5

The stock solution of **4db** and $CaOH_2$ buffer (pH = 12.5) were mixed at 1:1 to afford the 0.5 mM peptide solutions. Then these samples left at room temperature and analyzed by HPLC at corresponding time.

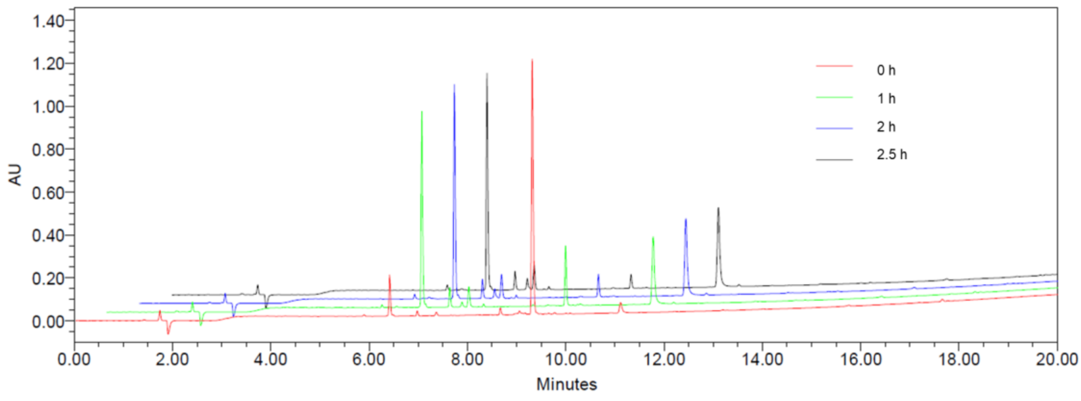

Figure S262. Overlay of HPLC-UV chromatogram at 220 nm of **4db** with basic condition (pH = 12.5).

Oxidation conditions (5 mM H_2O_2)

The stock solution of **4db** (1 mM; 500 µL) and H_2O_2 solutions (10 mM; 500 µL) were combined in a plastic Eppendorf and the final concentrations were 0.5 mM **4db**, 5 mM H_2O_2. Left it at room temperature and analyzed by HPLC at corresponding time.

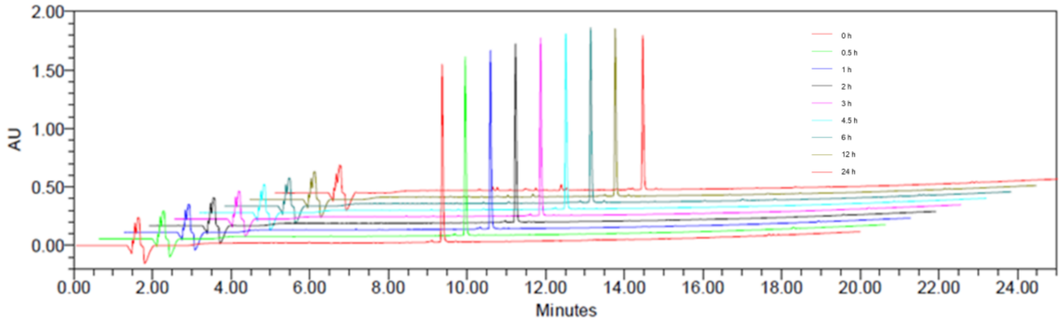

Figure S263. Overlay of HPLC-UV chromatogram at 220 nm of **4db** with Oxidation conditions (5 mM H_2O_2)

External thiol nucleophiles (100 mM GSH)

The stock solution of **4db** (1 mM; 500 µL) and GSH solutions (200 mM; 500 µL) were combined in a plastic Eppendorf and the final concentrations were 0.5 mM **4db**, 100 mM GSH. Left it at room temperature and analyzed by HPLC and LCMS at corresponding time.

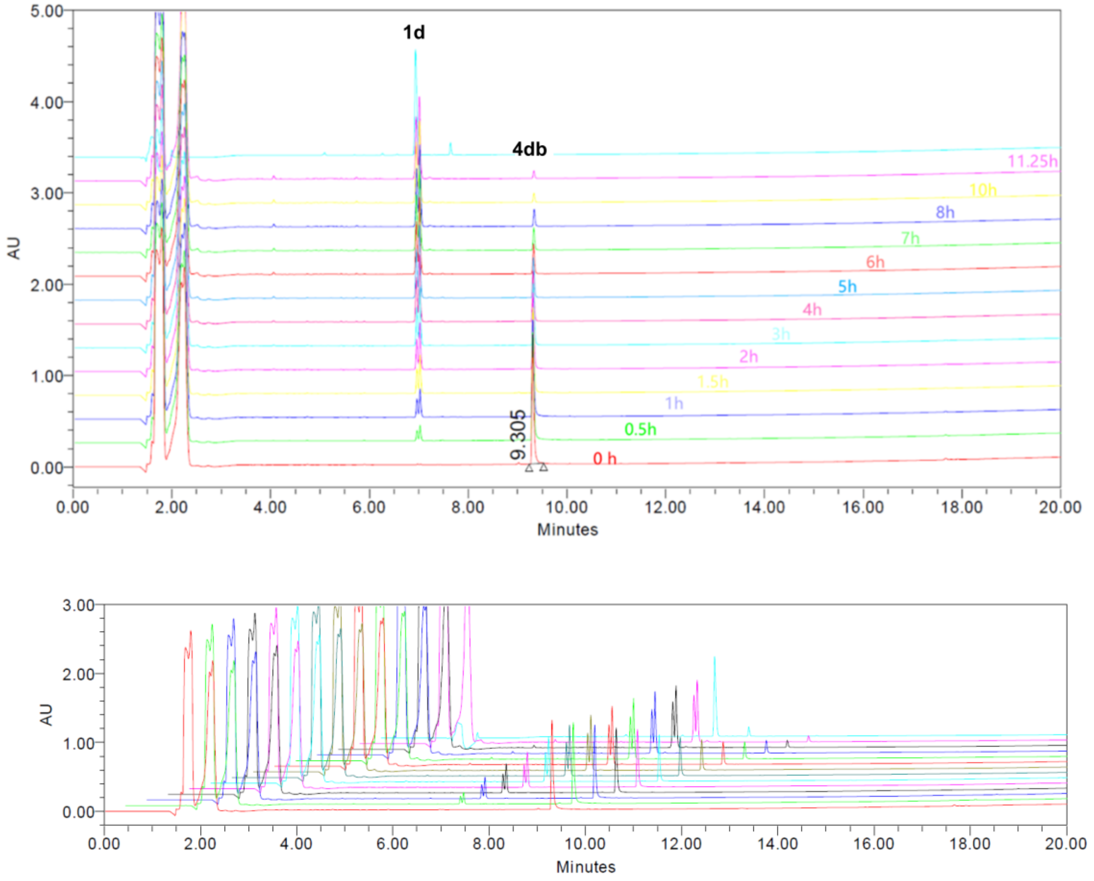

Figure S264. Overlay of HPLC-UV chromatogram at 220 nm of **4db** with external thiol nucleophiles (100 mM GSH)

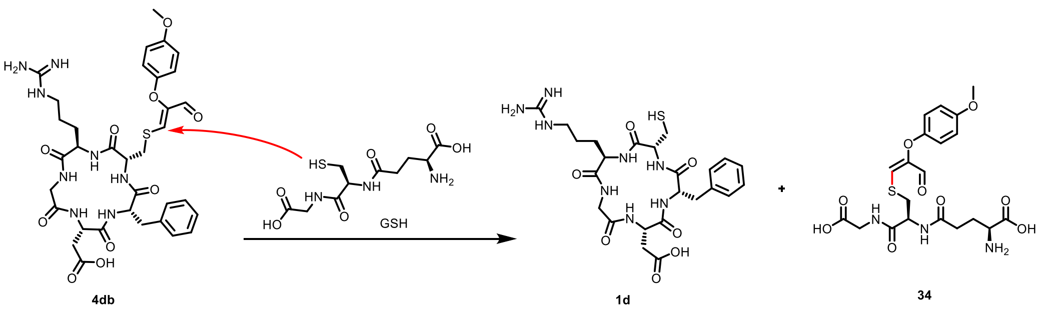

Figure S265. The mechanism of **1d** regeneration and **34** generation.

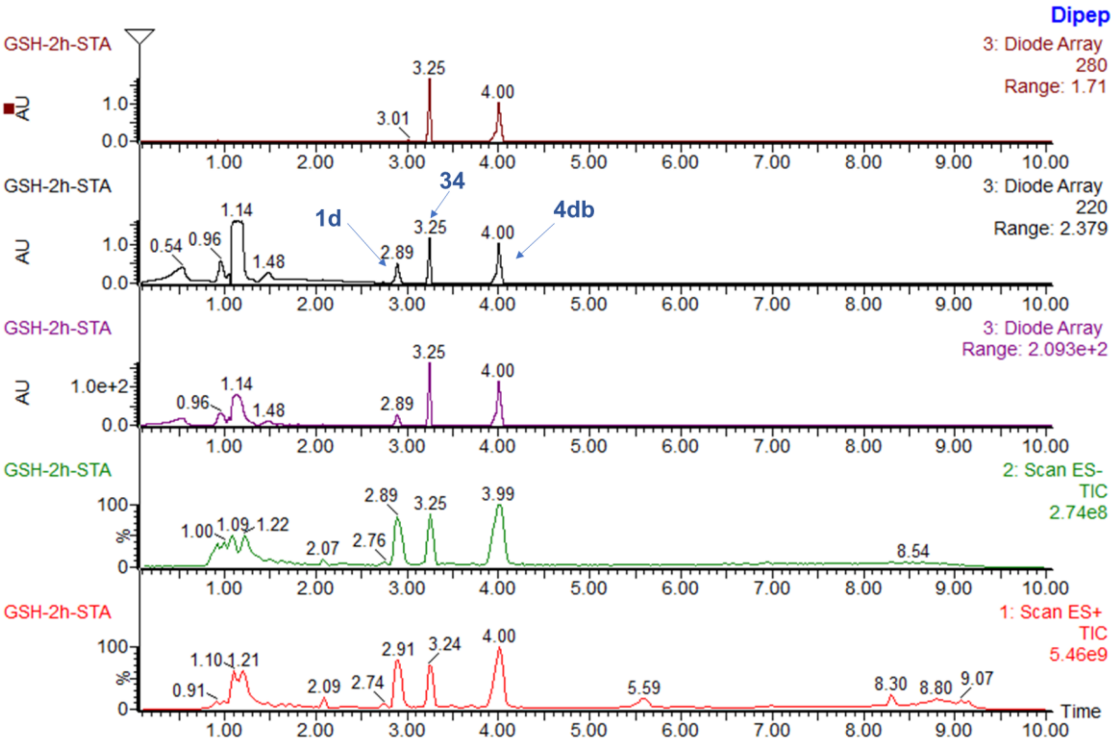

Figure S266. LCMS-UV chromatogram and MS chromatogram of **4db** with 100 mM GSH

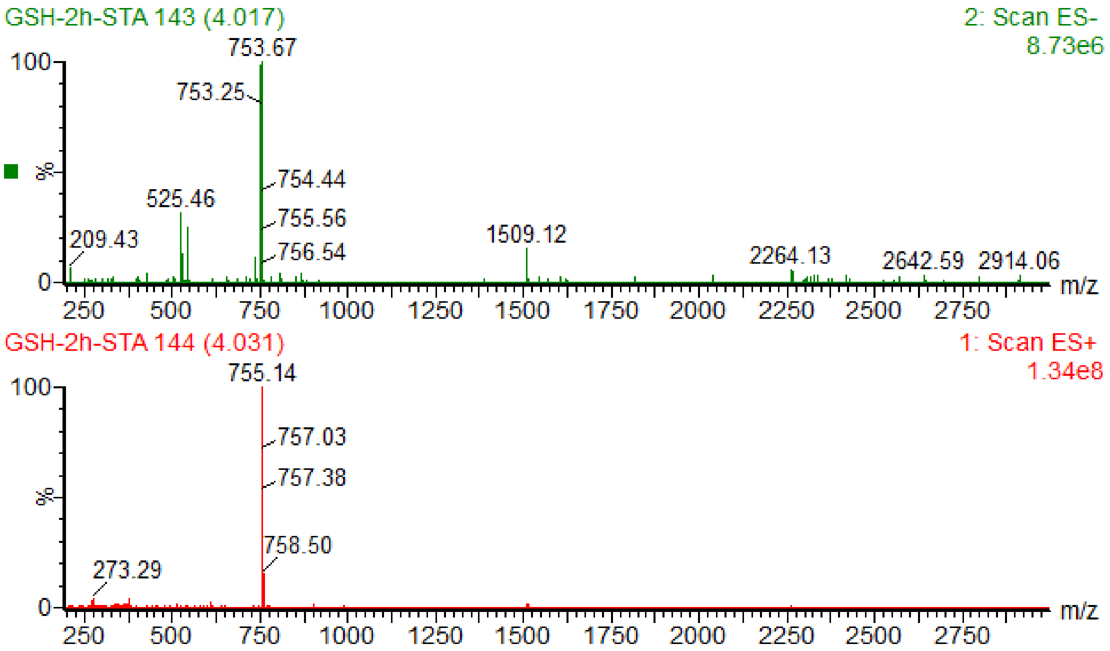

Figure S267. ESI-MS spectrum of **4db**.

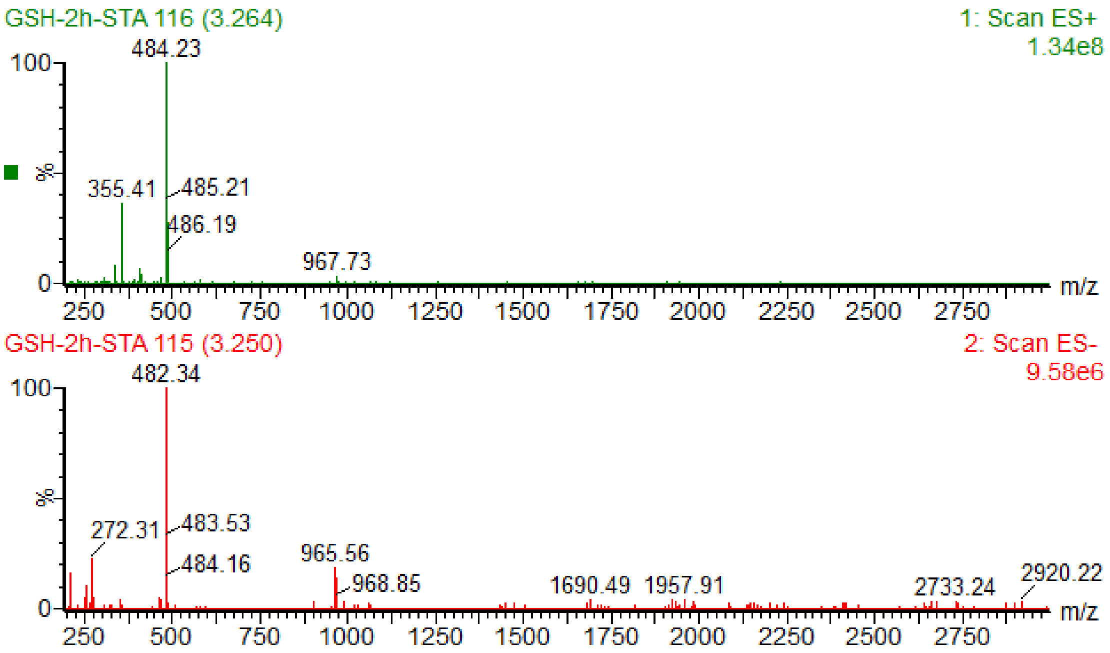

Figure S268. ESI-MS spectrum of **34**.

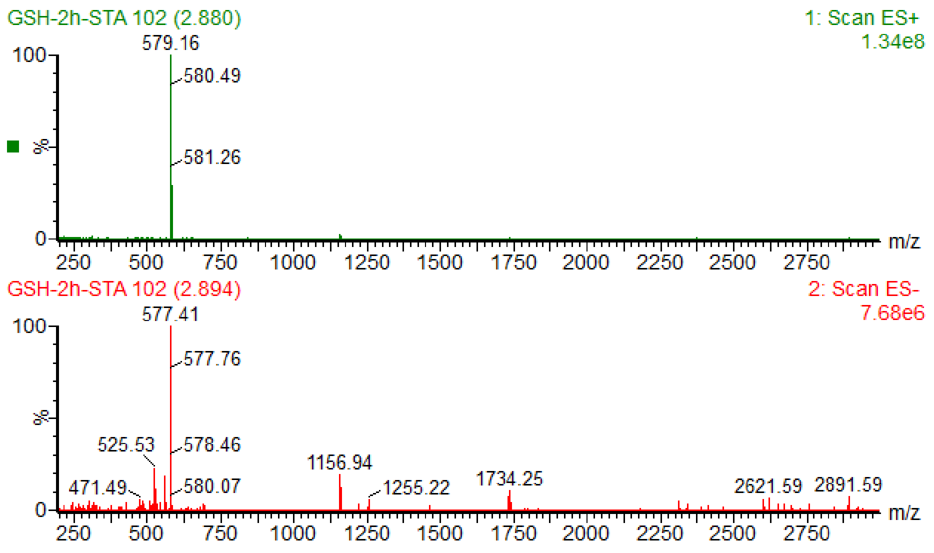

Figure S269. ESI-MS spectrum of **1d**.

Stability of 5a under external thiol nucleophiles (100 mM GSH)

The stock solution of **5a** (1 mM; 500 µL) and GSH solutions (200 mM; 500 µL) were combined in a plastic Eppendorf and the final concentrations were 0.5 mM **5a**, 100 mM GSH. Left it at room temperature and analyzed by HPLC and LCMS at corresponding time in 48 hours. All new compounds generated were characterized by LCMS.

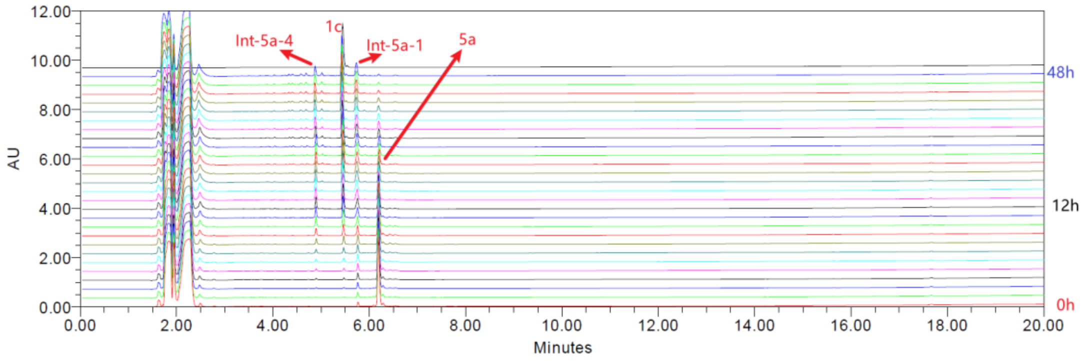

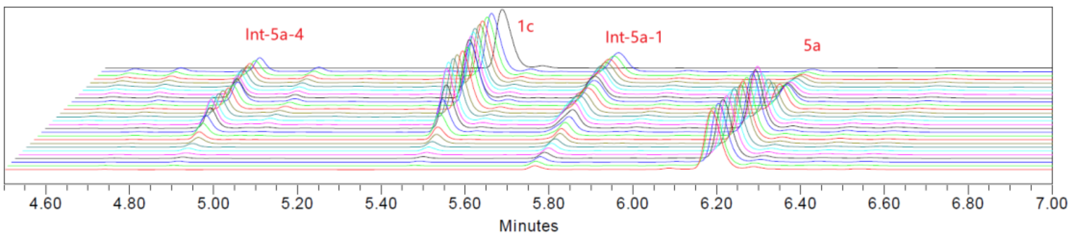

Figure S270. Overlay of HPLC-UV chromatogram at 220 nm of **5a** with external thiol nucleophiles (100 mM GSH)

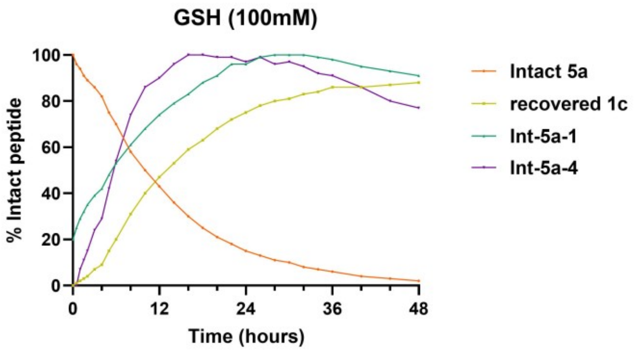

Figure S271. Stability of **5a** under external thiol nucleophiles. All data had been normalized.

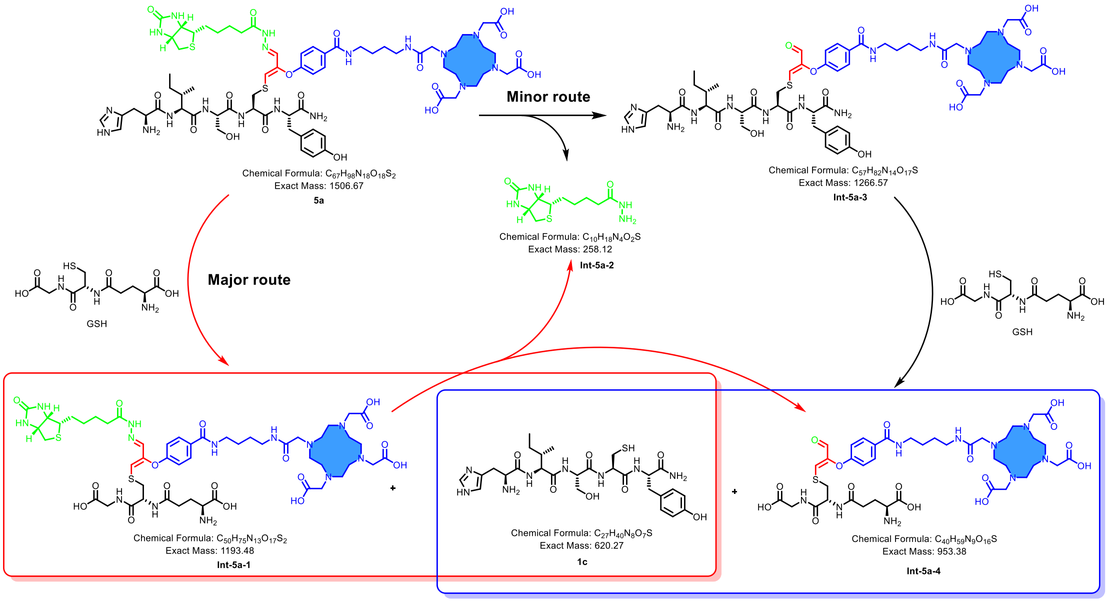

Figure S272. The mechanism of **1c** regeneration and **Int-5a-1, Int-5a-2, Int-5a-3, Int-5a-4** generation. Through LCMS and HPLC, it can be found that regeneration is mainly achieved through the main route, because very few **Int-5a-3** has been observed.

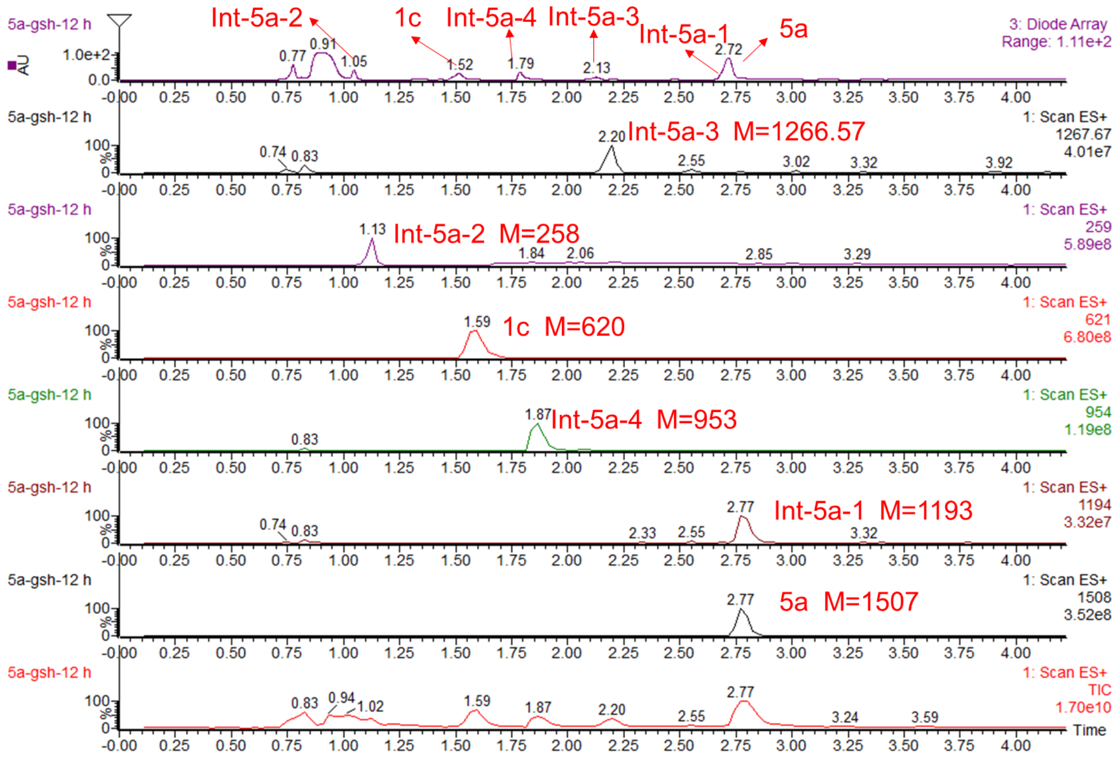

Figure S273. UPLC-MS chromatogram of reaction mixture after 12 hours including TIC and UV curve.

(Extract **1c, 5a, Int-5a-1, Int-5a-2, Int-5a-3** and **Int-5a-4** mass chromatograms from full scan data).

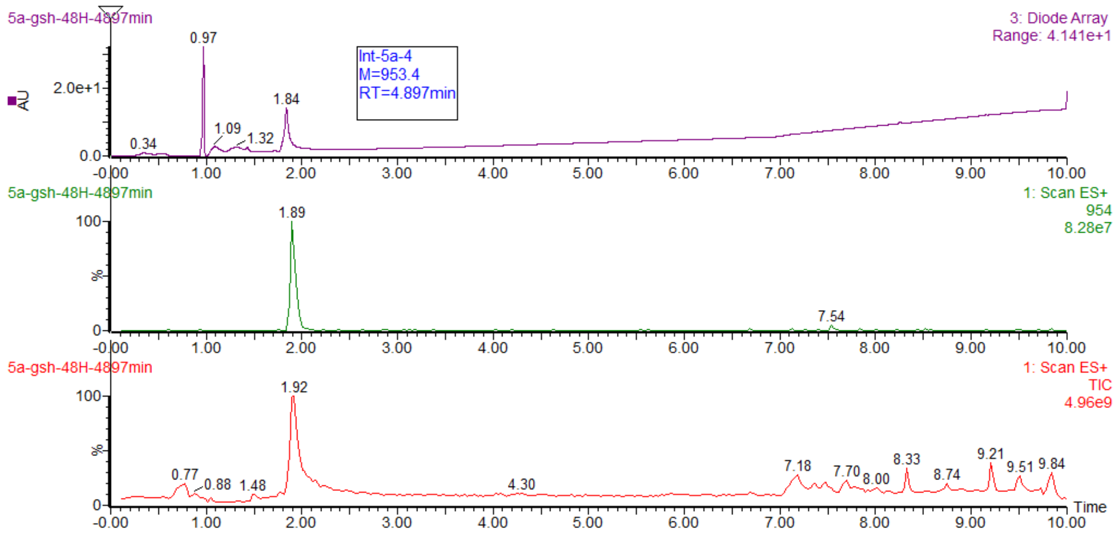

Figure S274. UPLC-MS chromatogram of material with a retention time of 4.897 minutes on HPLC including TIC and UV curve.

(Extract **Int-5a-4** mass chromatogram from full scan data).

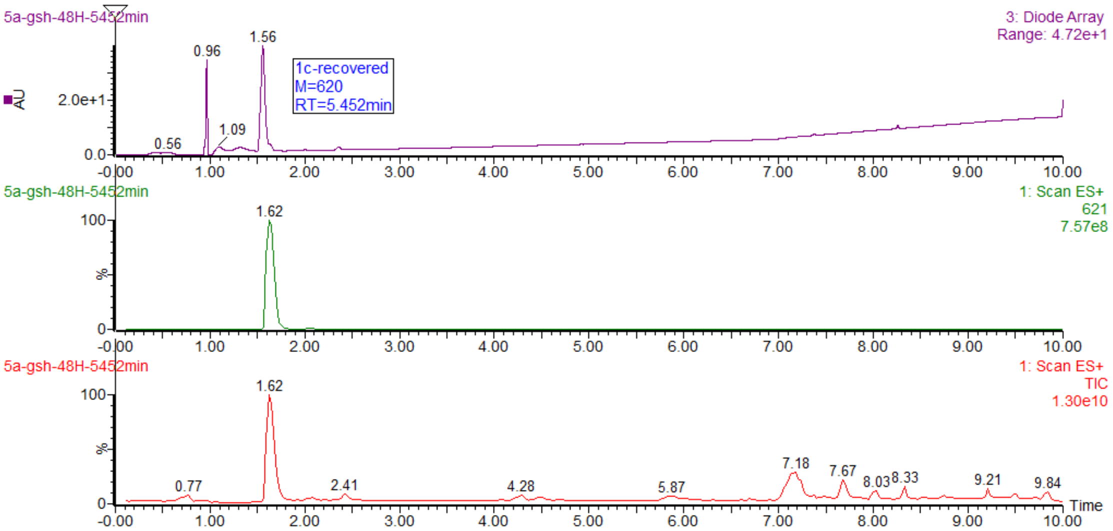

Figure S275. UPLC-MS chromatogram of material with a retention time of 5.452 minutes on HPLC including TIC and UV curve.

(Extract **1c-recovered** mass chromatogram from full scan data).

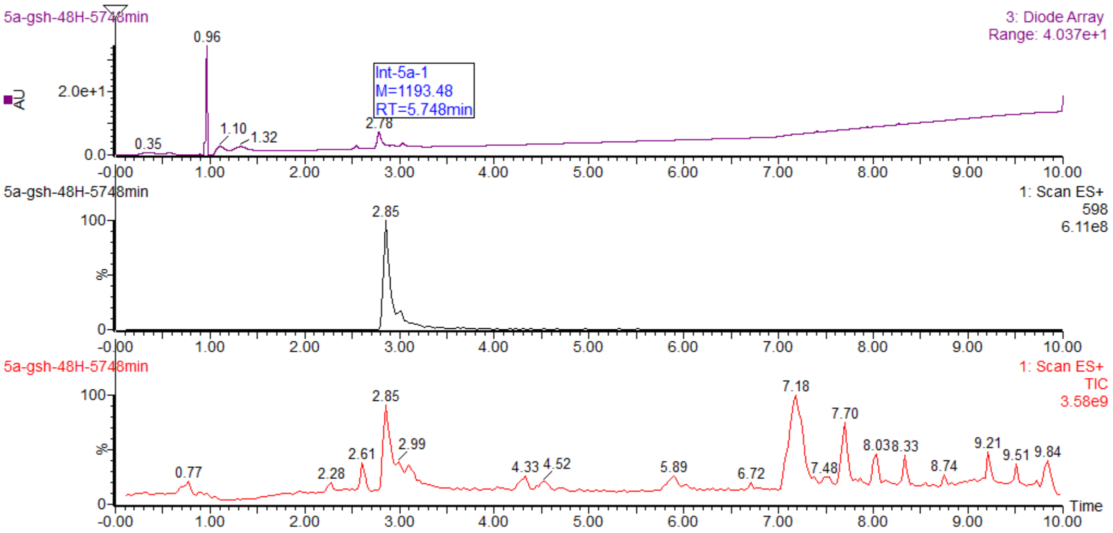

Figure S276. UPLC-MS chromatogram of material with a retention time of 5.748 minutes on HPLC including TIC and UV curve.

(Extract **Int-5a-1** mass chromatogram from full scan data).

11 Modification of Cys-containing proteins

LC-MS method for analysis of protein conjugation.

LC-MS was performed on a Waters SQ Detector2 mass spectrometer coupled to an Acquity UPLC system using an Acquity UPLC Protein BEH C4 column (300,1.7 um, 2.1 mm x 100 mm). Solvents A, water with 0.1% formic acid and B, 100% acetonitrile were used as the mobile phase at a flow rate of 0.3 mL/min. The gradient was programmed as follows: 5% B to 40% B in 8 min, then 70% B for 1 min, 100% B for 1 min. The electrospray source was operated with a capillaryvoltage of 3.0 kV and a cone voltage of 30 V. Nitrogen was used as the desolvation gas at a total flow of 800 L/h. Total mass spectra were reconstructed from the ion series using the MaxEnt algorithm preinstalled on MassLynx software (v.4.1 from Waters) according to the manufacturer's instructions. To obtain the ion series described, the major peak of the chromatogram were selected for integration and further analysis. A typical analysis of a protein is described below. The combined ion series and deconvoluted spectra are shown below.

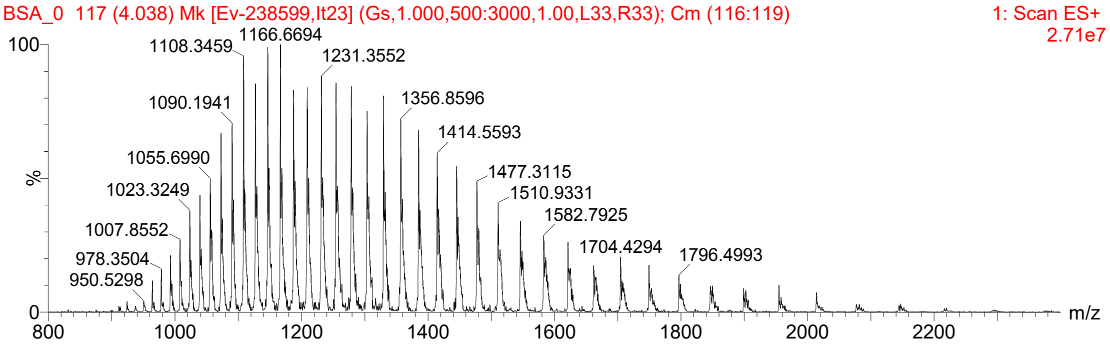

Figure S277. The combined ion series of BSA.

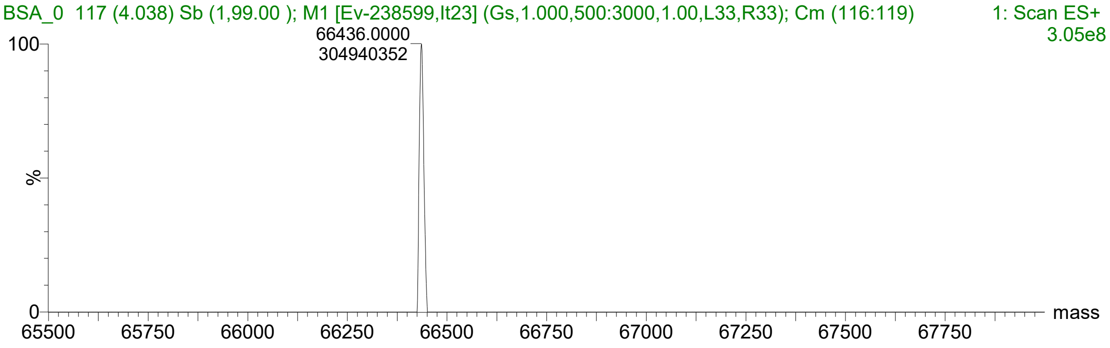

Figure S278. The deconvoluted spectra of BSA. The expected molecular weight was 66430, and the observed molecular weight was 66436. The intensity was 304940352.

The conversion rate of proteins can be calculated using the following formula:

$$Conversion\ rate(\%) = \frac{B2}{B1 + B2} \times 100$$

B1: The mass intensity of BSA in the reaction mixture.

B2: The mass intensity of protein conjugation in the reaction mixture.

11.1 Direct modification of proteins using 2e

BSA (0.1 μmol) was dissolved in PBS buffer (0.5 M, pH = 7.4, 1.8 mL). Compound **2h** of 14 equiv. dissolved in CH_3CN (0.2 mL) was next added to the buffer and the reaction mixture was incubated at room temperature for 5 h. Dilute it to a concentration of 0.5mg/mL and the resulting sample was analyzed by 10% SDS-PAGE. SDS loading buffer: 50 mM Tris, pH 6.8, glycol 10% (v/v), SDS 2% (w/v), bromophenol 0.1% (w/v). In the Tanon imaging system, intra gel fluorescence imaging was performed (excitation light source in 530 nm, emission filter in 590 nm), and after imaging, coomassie blue staining was performed to obtain the following results, as showed in **Figure S5**.

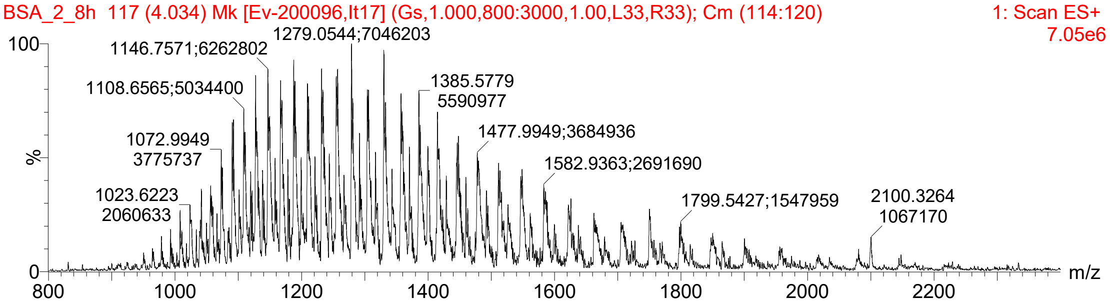

Figure S279. The combined ion series of BSA modification by **2e**. Incubated in buffer at 37 °C for 8 hours.

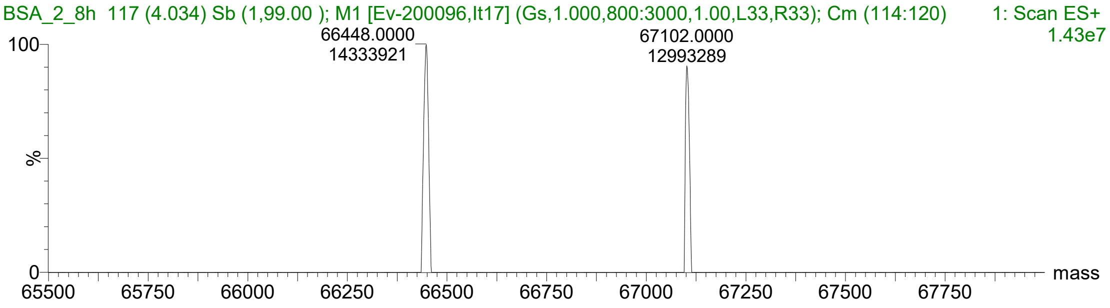

Figure S280. The deconvoluted spectra of BSA modification by **2e**. Incubated in buffer at 37 °C for 8 hours. The expected molecular weight was 67102, and the observed molecular weight was 67102. The intensity of protein conjugation was 12993289, and the intensity of BSA was 14333921. The conversion rate was 48%.

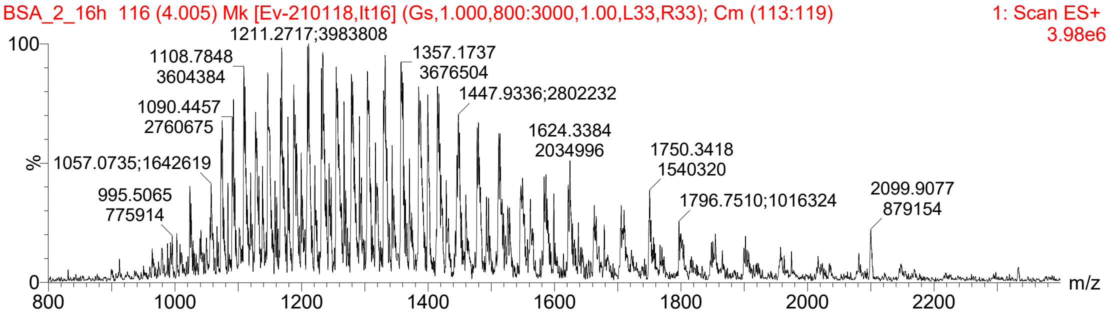

Figure S281. The combined ion series of BSA modification by **2e**. Incubated in buffer at 37 °C for 16 hours.

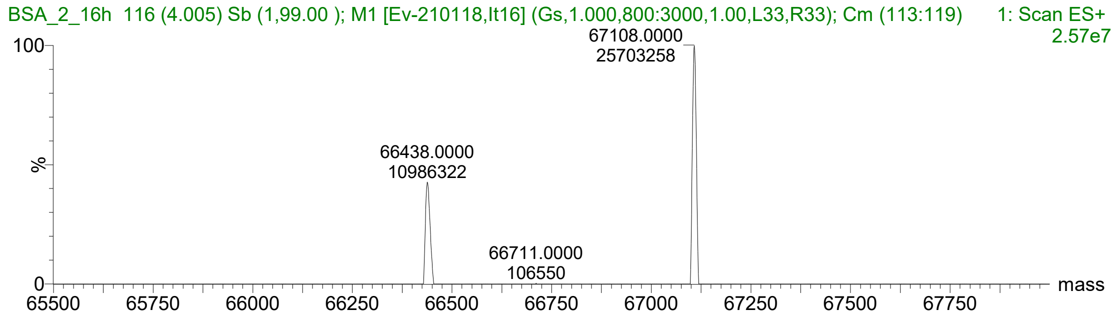

Figure S282. The deconvoluted spectra of BSA modification by **2e**. Incubated in buffer at 37 °C for 16 hours. The expected molecular weight was 67102, and the observed molecular weight was 67108. The intensity of protein conjugation was 25703258, and the intensity of BSA was 10986322. The conversion rate was 70%.

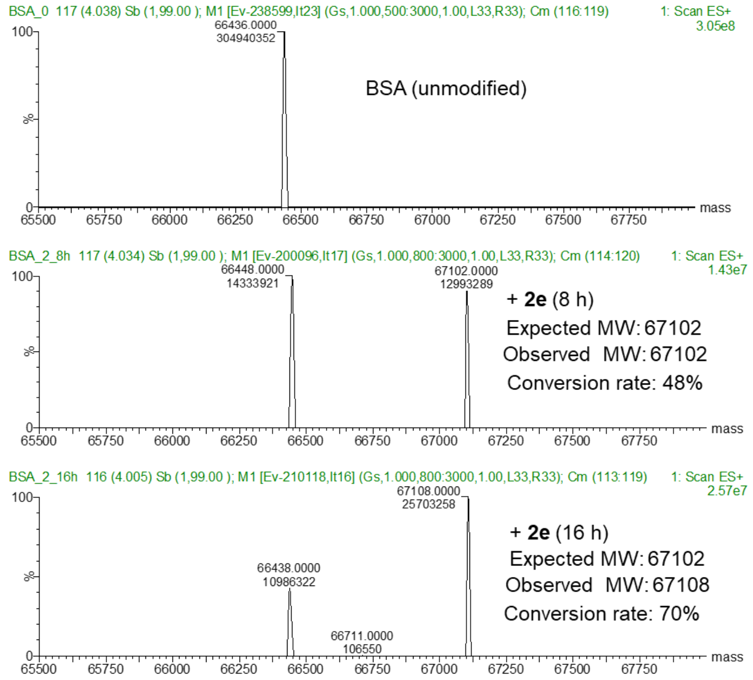

Figure S283. Overlay of deconvoluted spectra of BSA modification by **2e.** Incubated in buffer at 37 °C.

11.2 Secondary labeling of proteins using 2i and TCO-Cy5

A mixture of 1.8 mL PBS buffer (0.5 M, pH = 7.4, dissolving 0.1 μmol BSA) and 0.2 mL CH_3CN dissolving **2i** of 10 equiv., was incubated at room temperature for 5 h. Take 50 μL of it and dilute it to 0.5 mg/mL concentration as a stock solution for SDS-PAGE. Then, **TCO-Cy5** of 12 equiv. was next added to the resulting sample and incubated at room temperature for 1 h. The solution was diluted to a concentration of 0.5 mg/mL before analyzed by 10% SDS-PAGE. In the Tanon imaging system, intra gel fluorescence imaging was performed (excitation light source in 620 nm, emission filter in 699 nm), and after imaging, coomassie blue staining was performed to obtain the following results, as showed in **Figure 4C**.

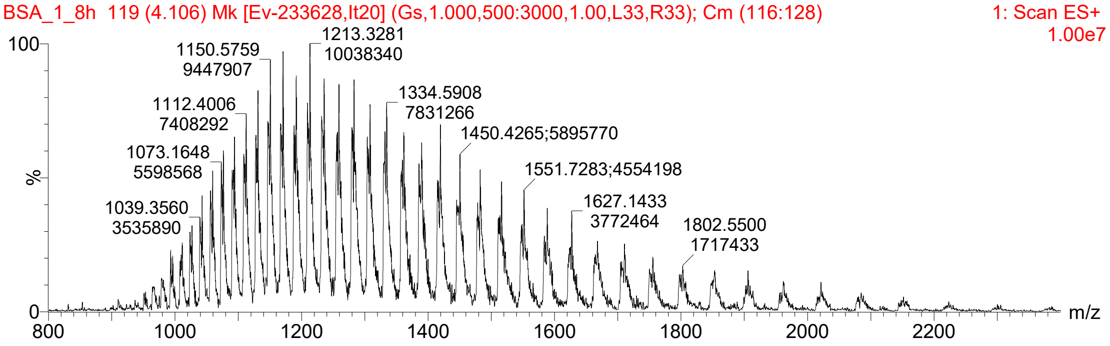

Figure S284. The combined ion series of BSA modification by **2i**. Incubated in buffer at 37 °C for 8 hours.

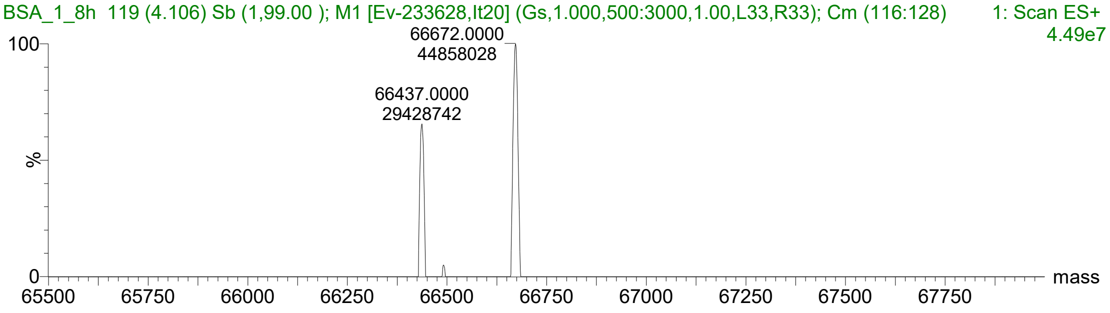

Figure S285. The deconvoluted spectra of BSA modification by **2i**. Incubated in buffer at 37 °C for 8 hours. The expected molecular weight was 66670, and the observed molecular weight was 66672. The intensity of protein conjugation was 44858028, and the intensity of BSA was 29428742. The conversion rate was 60%.

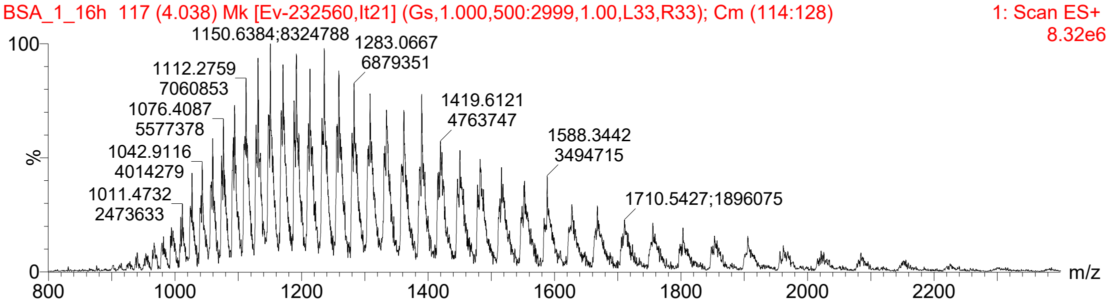

Figure S286. The combined ion series of BSA modification by **2i**. Incubated in buffer at 37 °C for 16 hours.

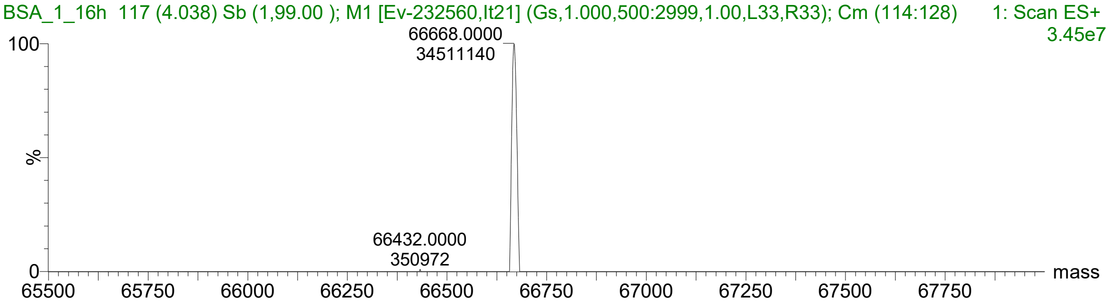

Figure S287. The deconvoluted spectra of BSA modification by **2i**. Incubated in buffer at 37 °C for 16 hours. The expected molecular weight was 66670, and the observed molecular weight was 66668. The intensity of protein conjugation was 34511140, and the intensity of BSA was 350972. The conversion rate was 99%.

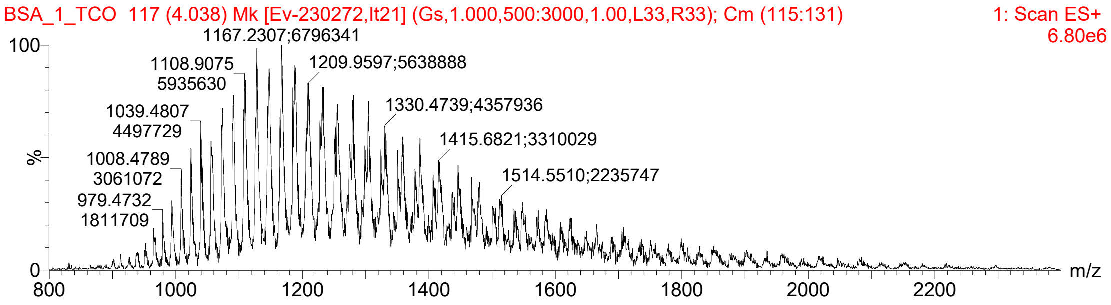

Figure S288. The combined ion series of BSA modification by **2i** and **Tco-Cy5**. Add TCO-Cy5 to the reaction solution of **2i** and BSA, which incubated in buffer at 37 °C for 16 hours, and then incubated for 1 hour.

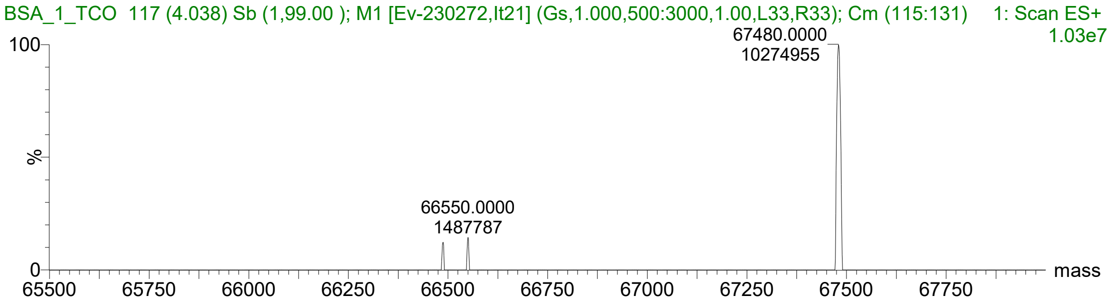

Figure S289. The deconvoluted spectra of BSA modification by **2i** and **Tco-Cy5**. Add TCO-Cy5 to the reaction solution of **2i** and BSA, which incubated in buffer at 37 °C for 16 hours, and then incubated for 1 hour. The expected molecular weight was 67478, and the observed molecular weight was 67480. The conversion rate was above 99%.

12 Spectra of new compounds

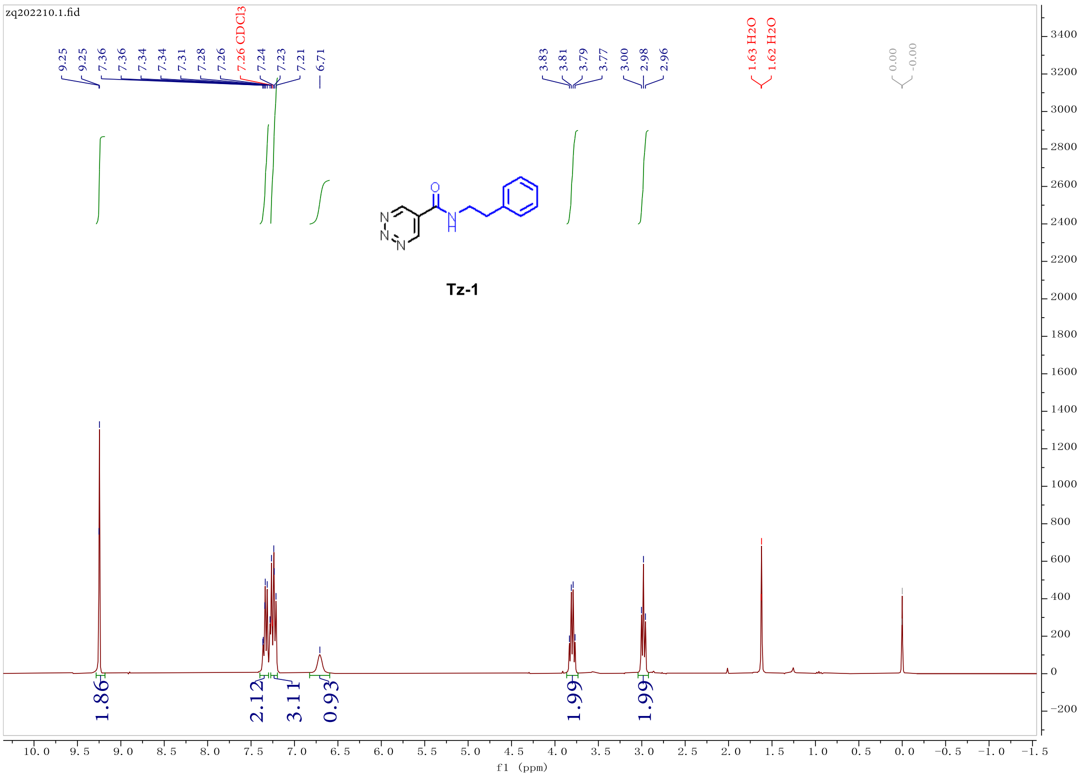

Figure S290. ^{1}H NMR spectrum of **Tz-1**.

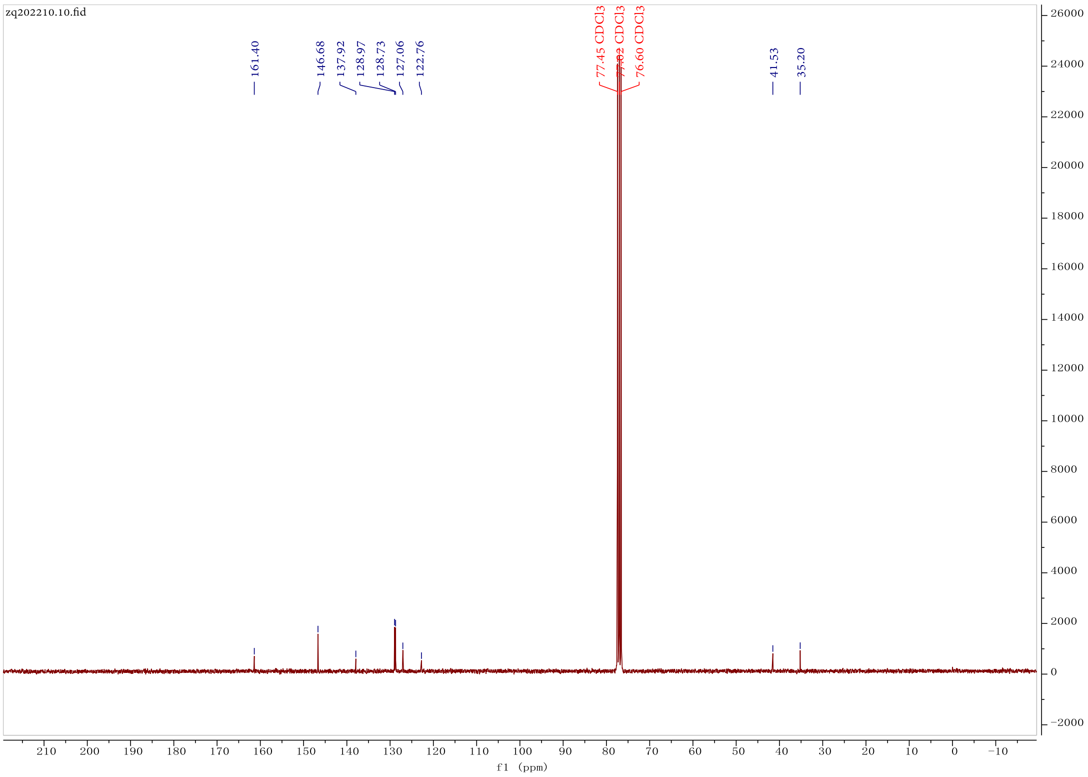

Figure S291. ^{13}C NMR spectrum of **Tz-1**.

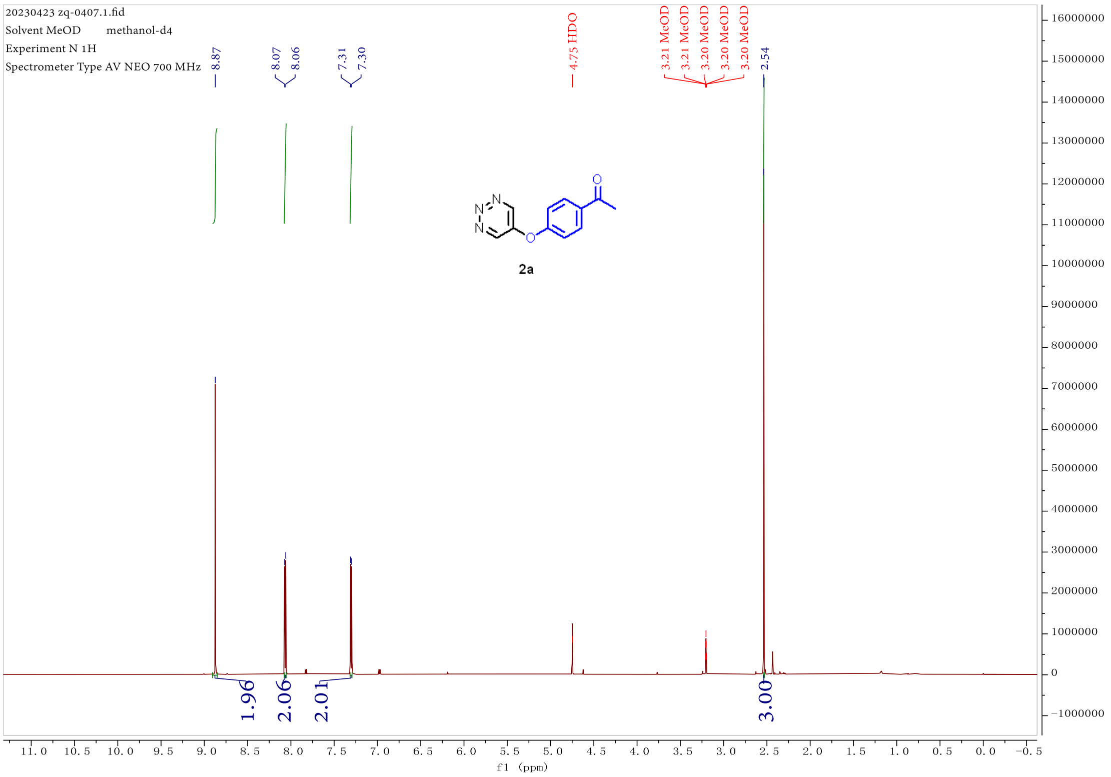

Figure S292. ^{1}H NMR spectrum of **2a**.

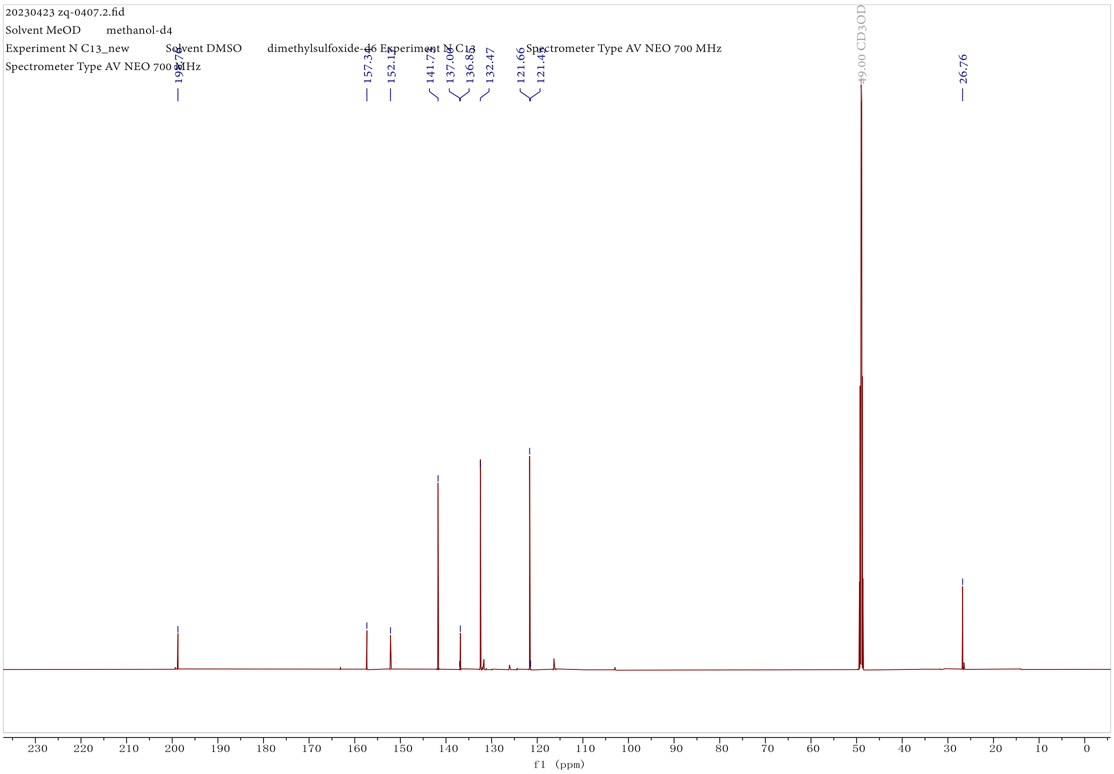

Figure S293. ^{13}C NMR spectrum of **2a**.

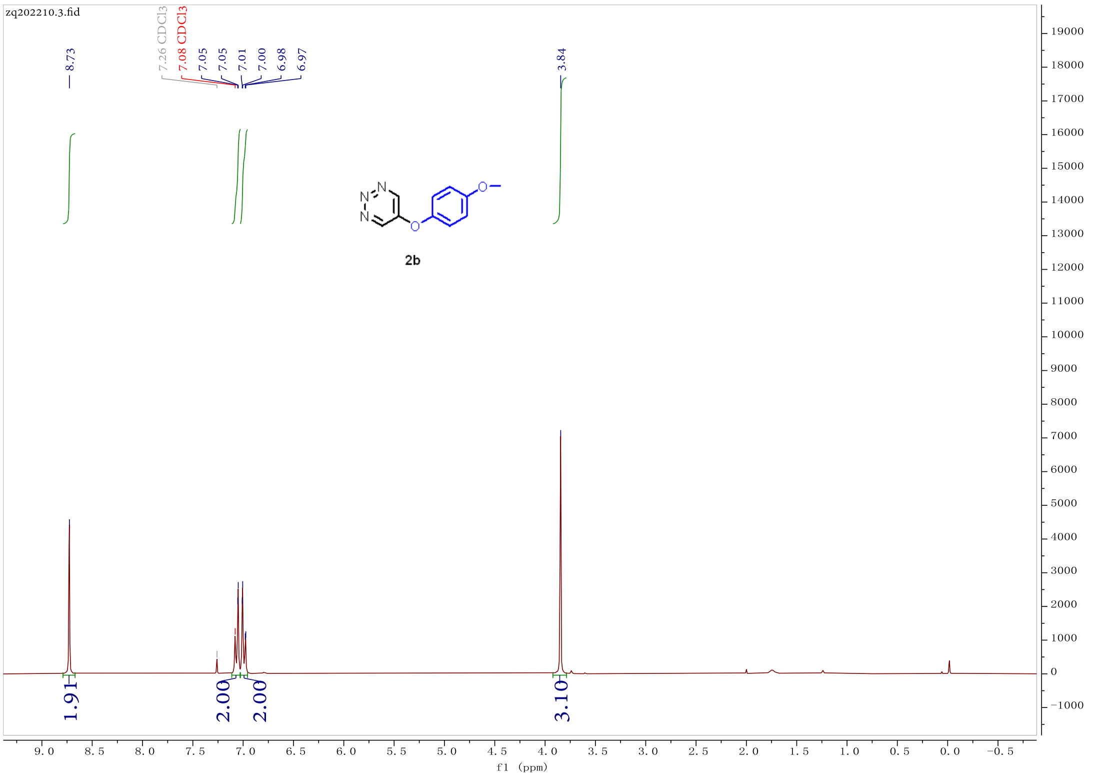

Figure S294. ^{1}H NMR spectrum of **2b**.

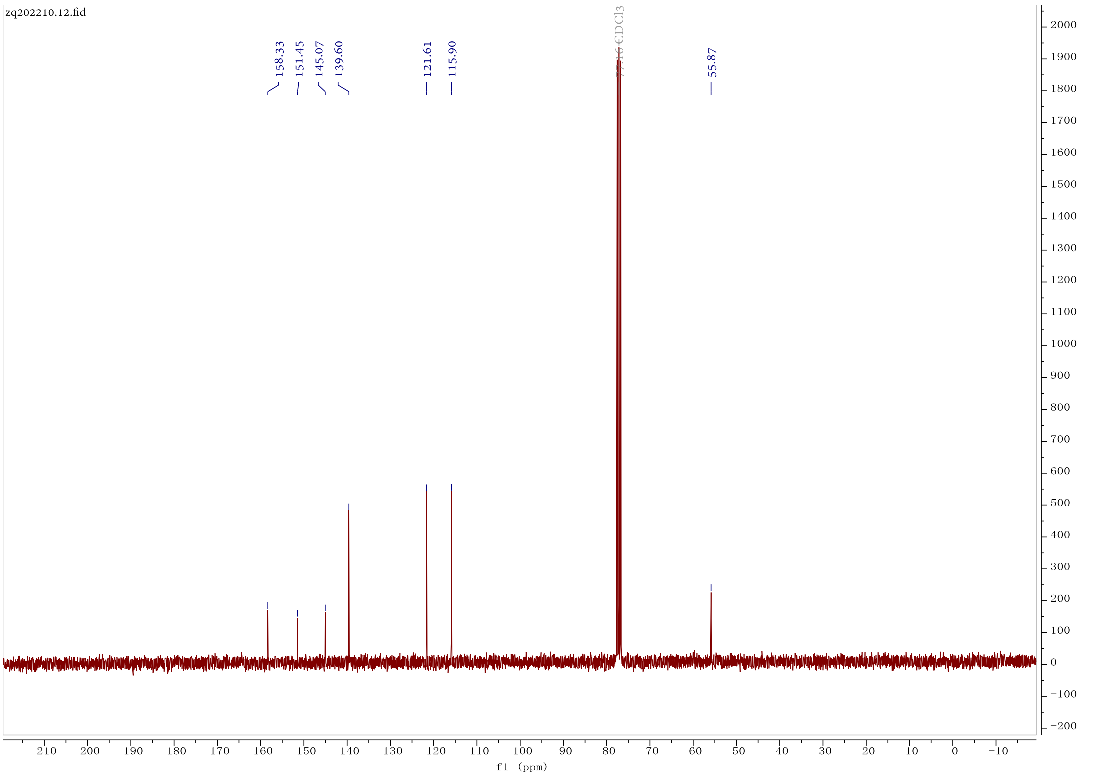

Figure S295. ^{13}C NMR spectrum of **2b**.

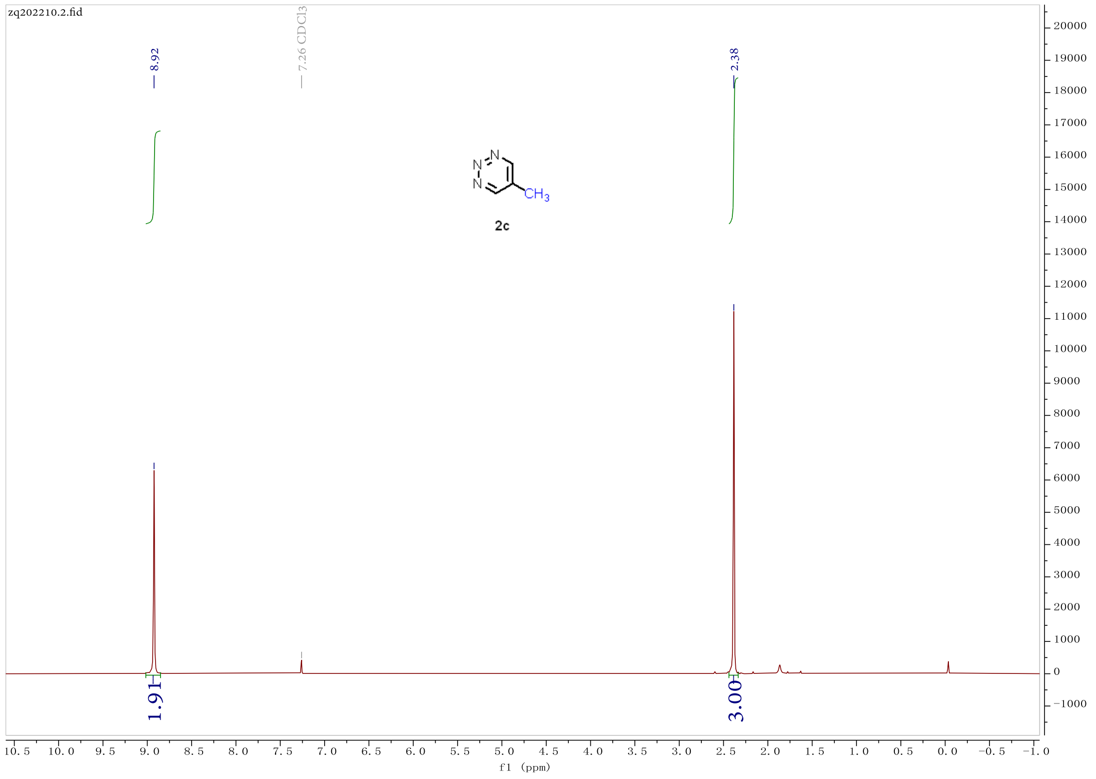

Figure S296. ^{1}H NMR spectrum of **2c**.

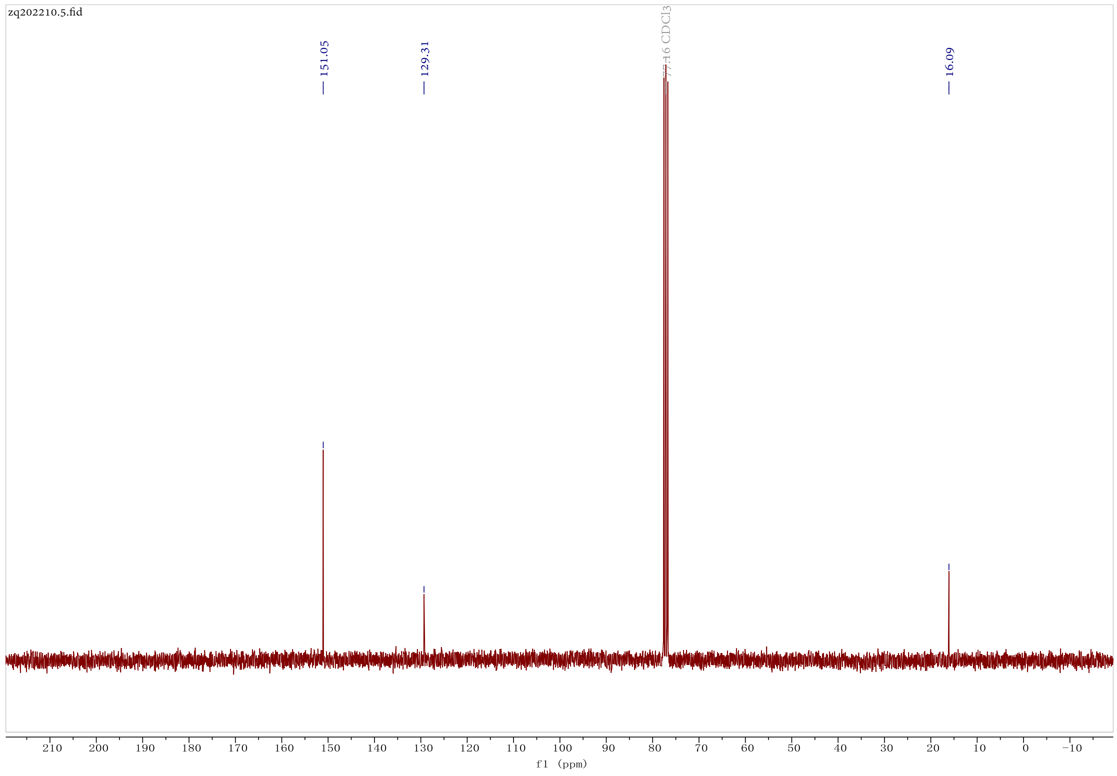

Figure S297. ^{13}C NMR spectrum of **2c**.

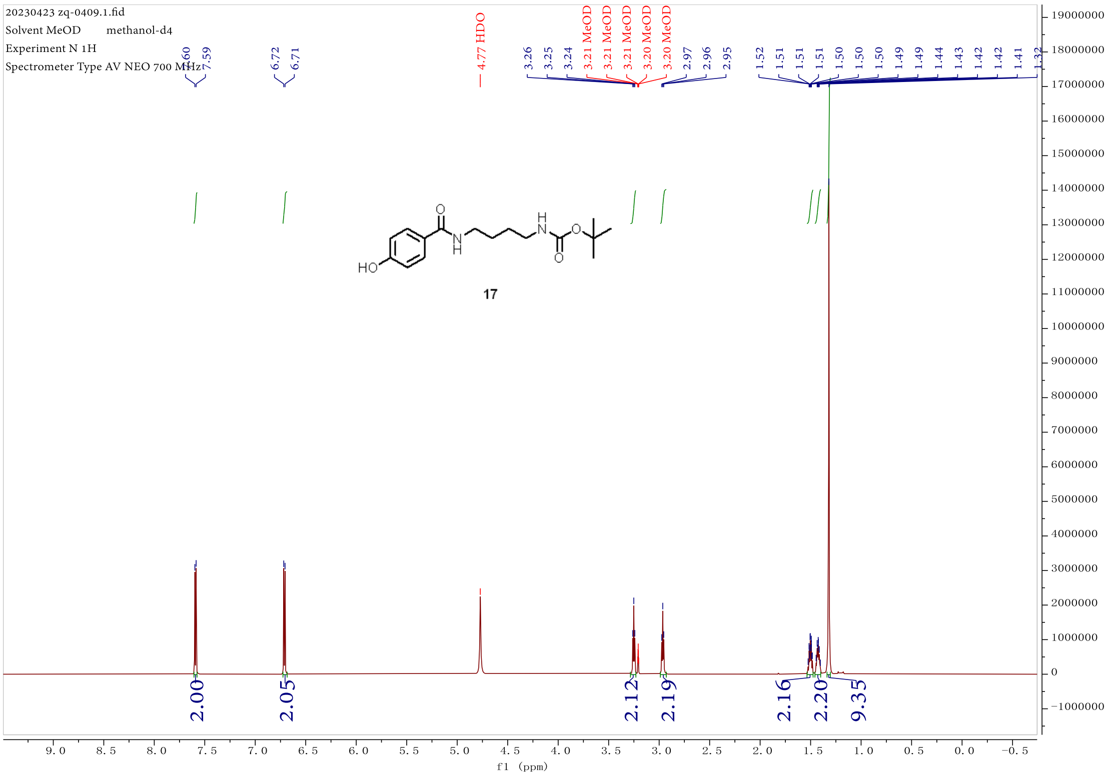

Figure S298. ^{1}H NMR spectrum of **17**.

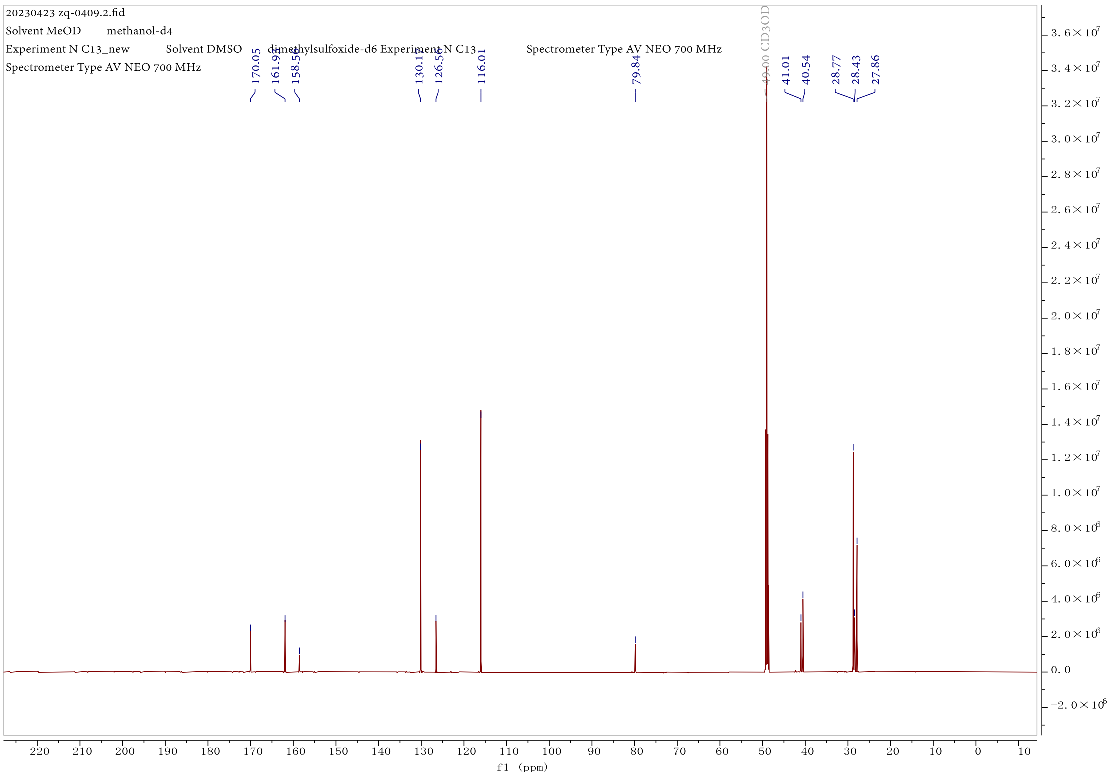

Figure S299. ^{13}C NMR spectrum of **17**.

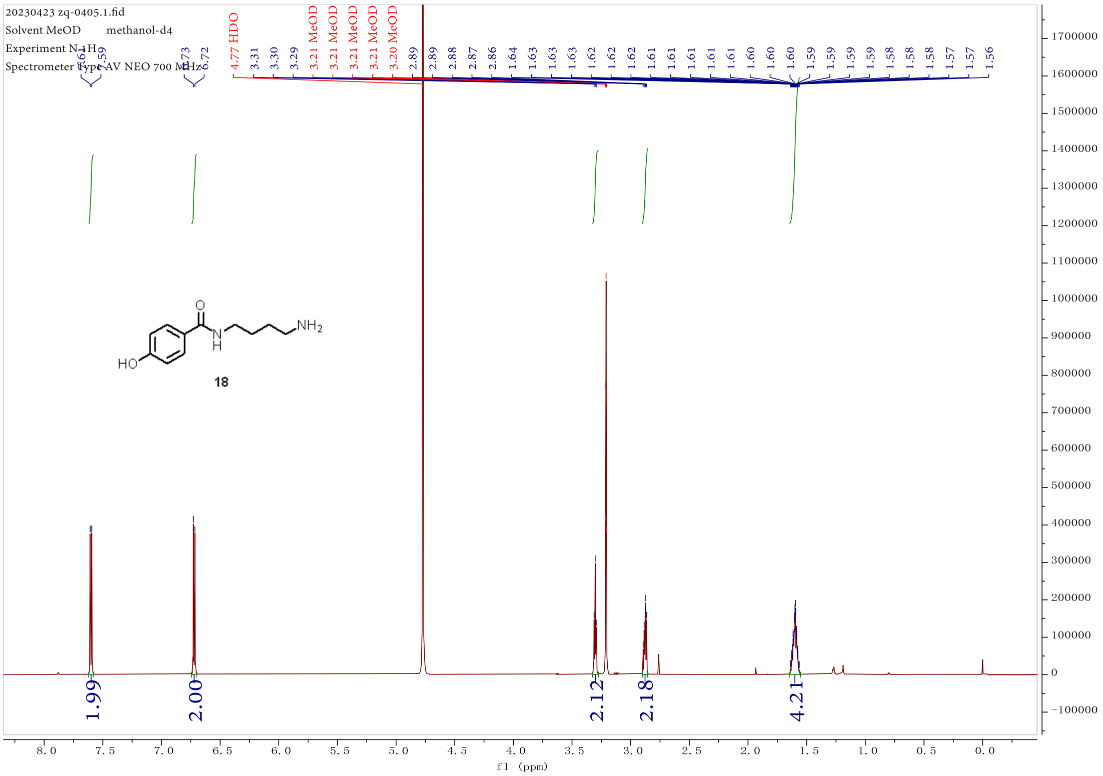

Figure S300. ^{1}H NMR spectrum of **18**.

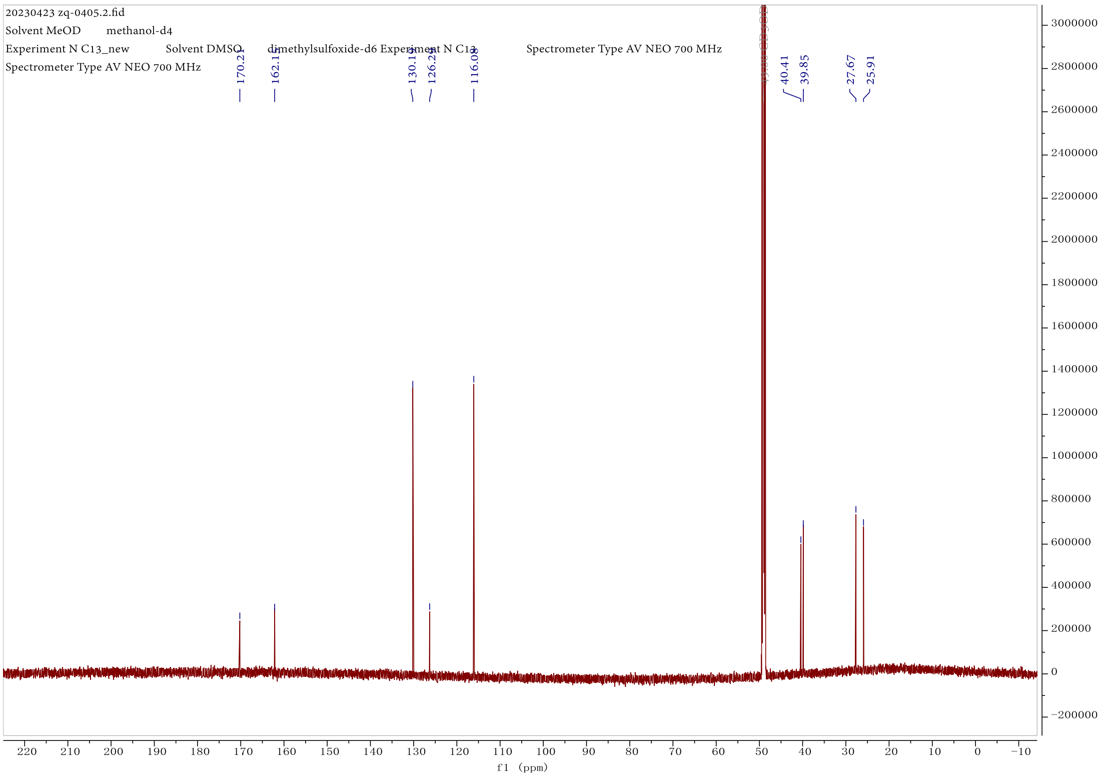

Figure S301. ^{13}C NMR spectrum of **18**.

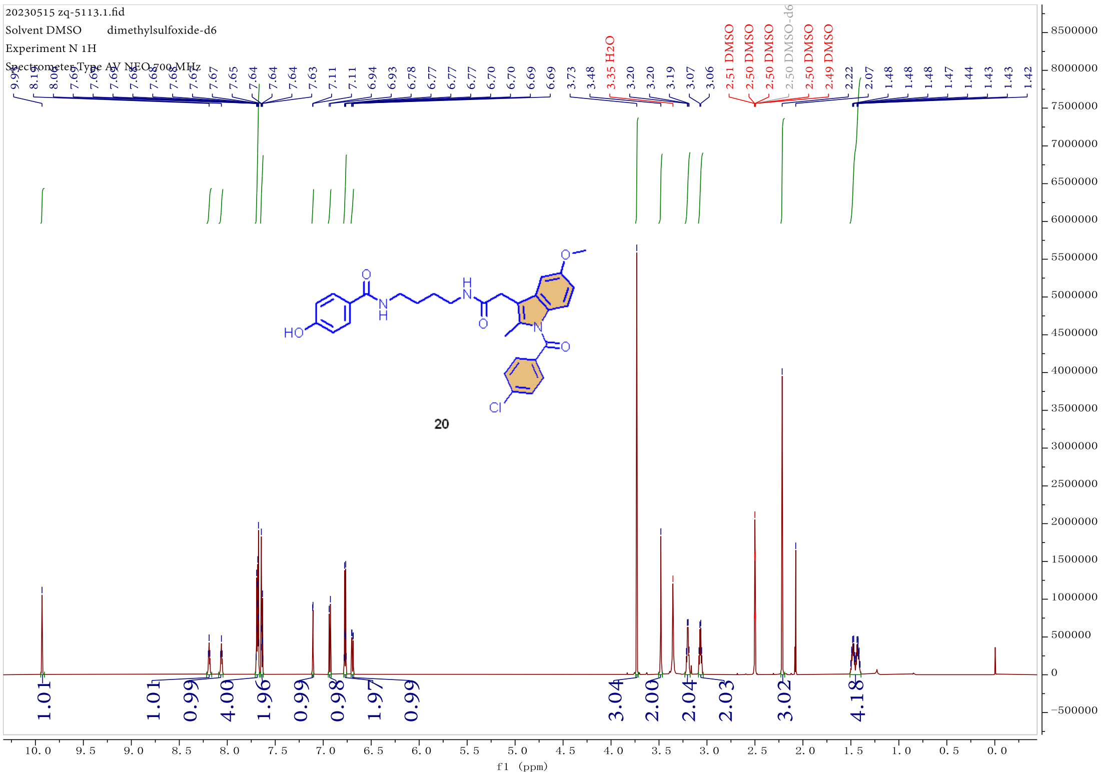

Figure S302. ^{1}H NMR spectrum of **20**.

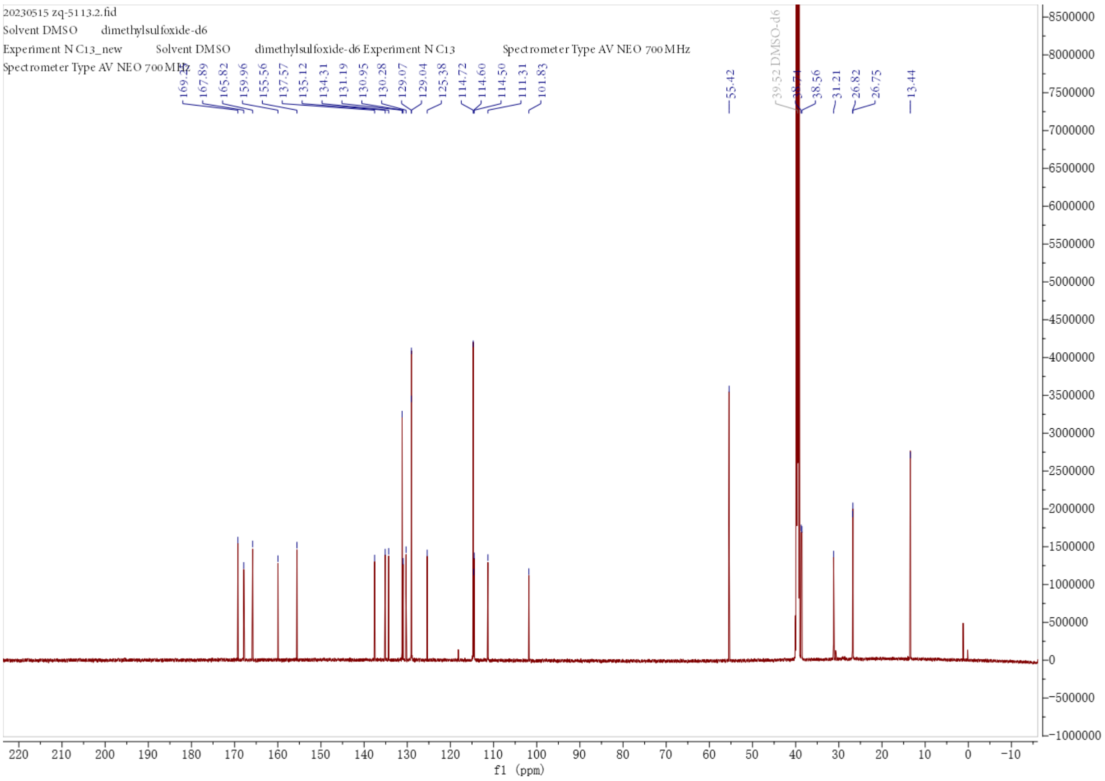

Figure S303. ^{13}C NMR spectrum of **20**.

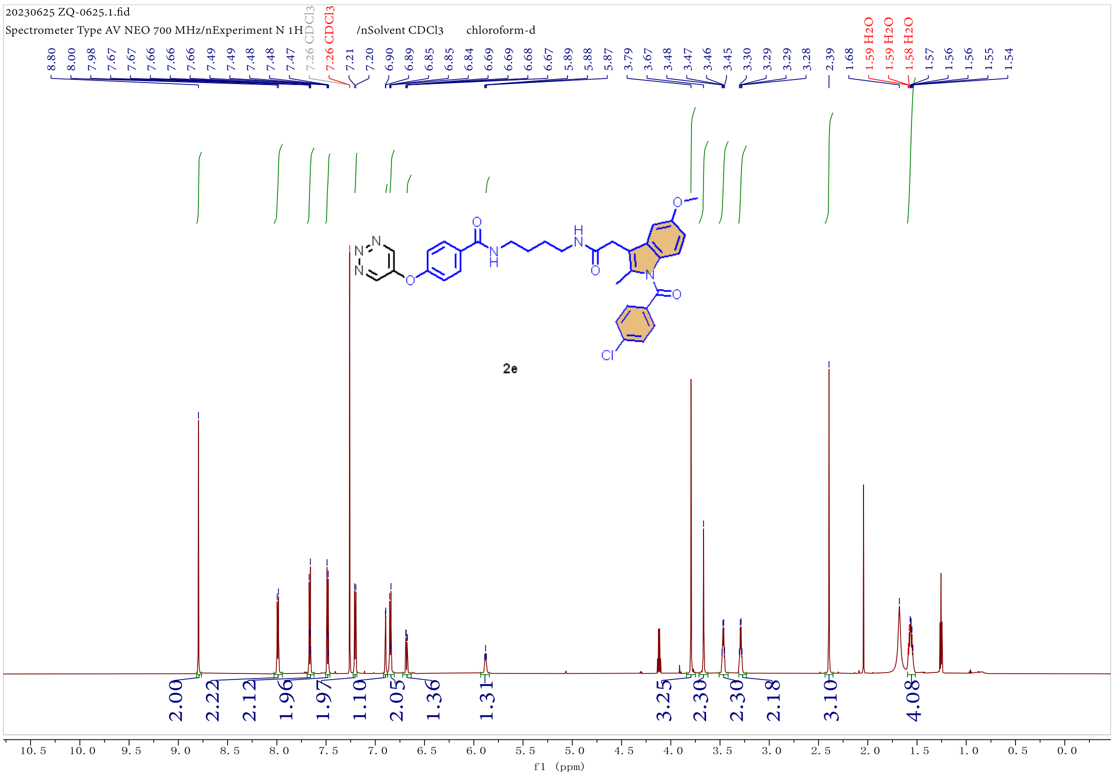

Figure S304. ^{1}H NMR spectrum of **2e**.

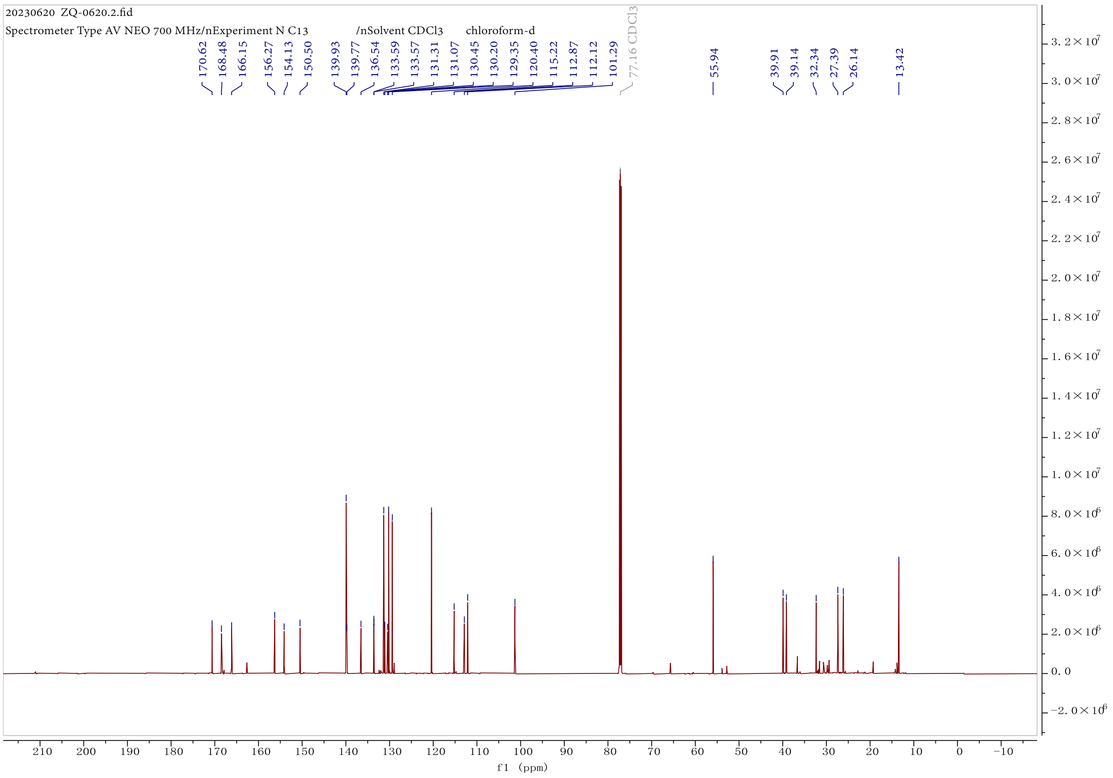

Figure S305. ^{13}C NMR spectrum of **2e**.

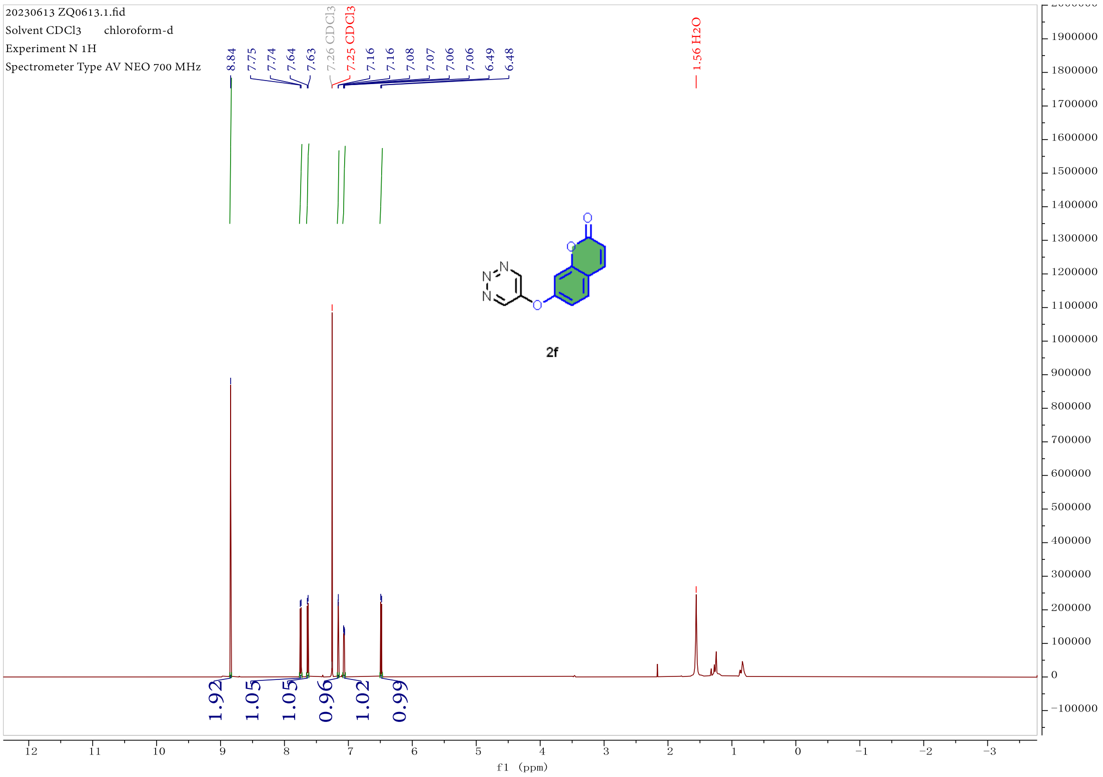

Figure S306. ^{1}H NMR spectrum of **2f**.

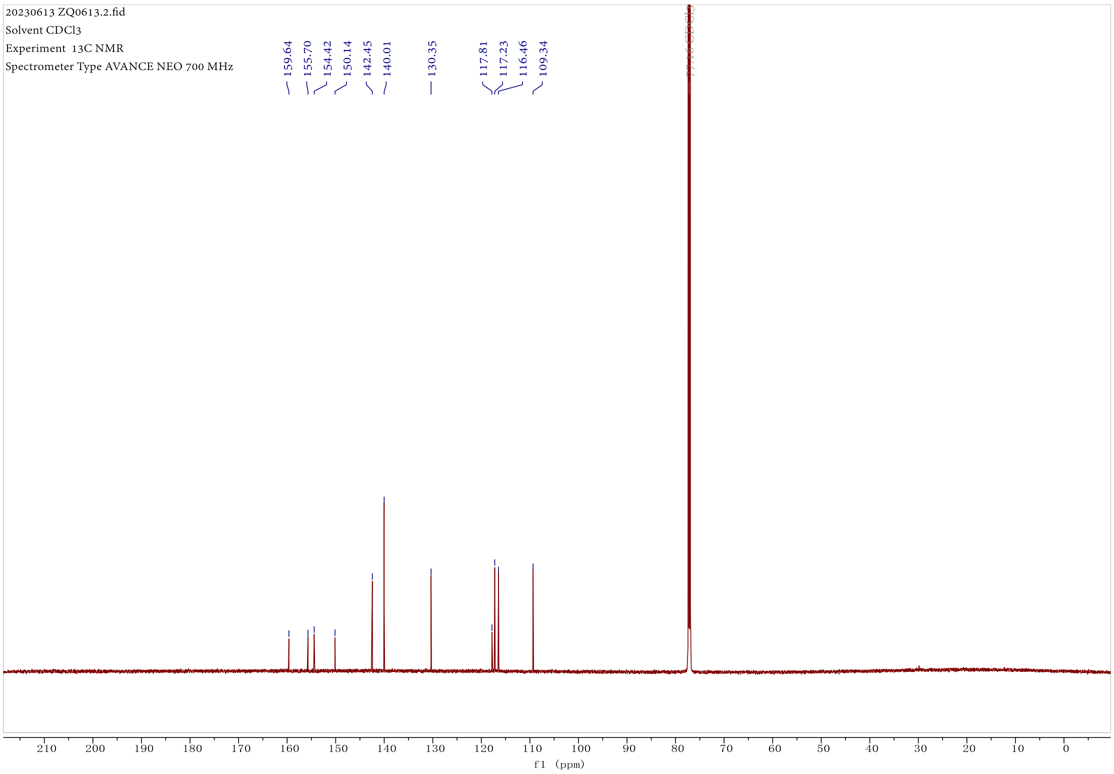

Figure S307. ^{13}C NMR spectrum of **2f**.

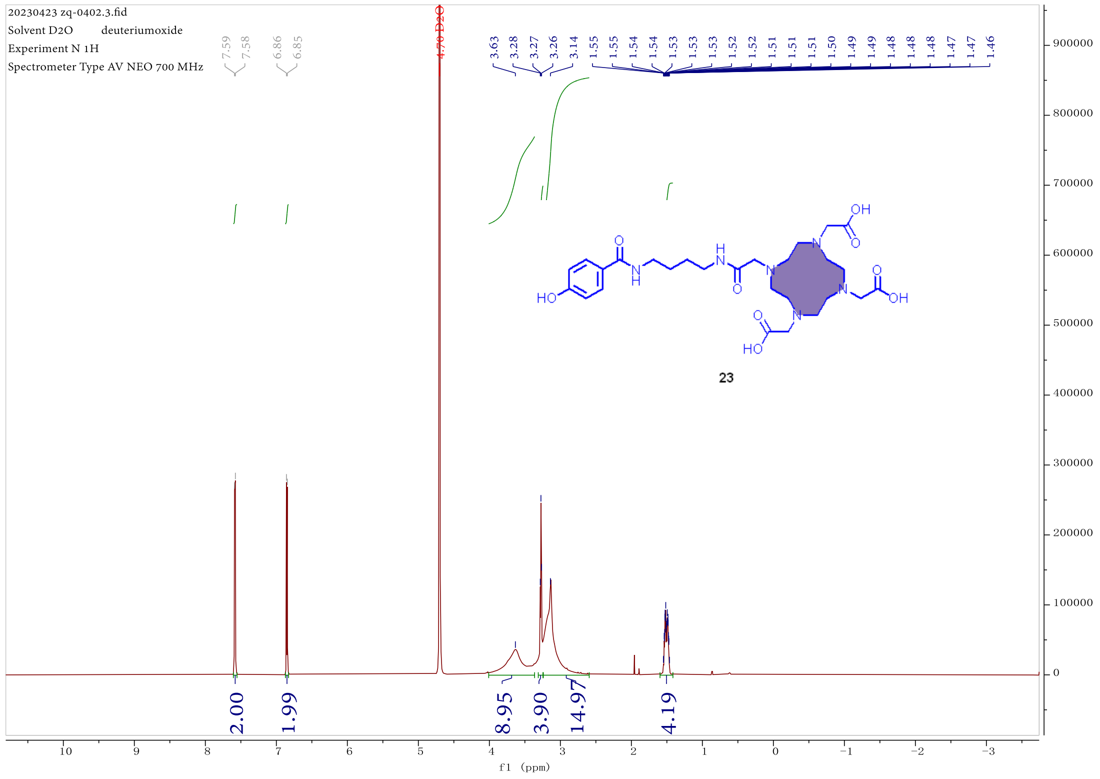

Figure S308. ^{1}H NMR spectrum of **23**.

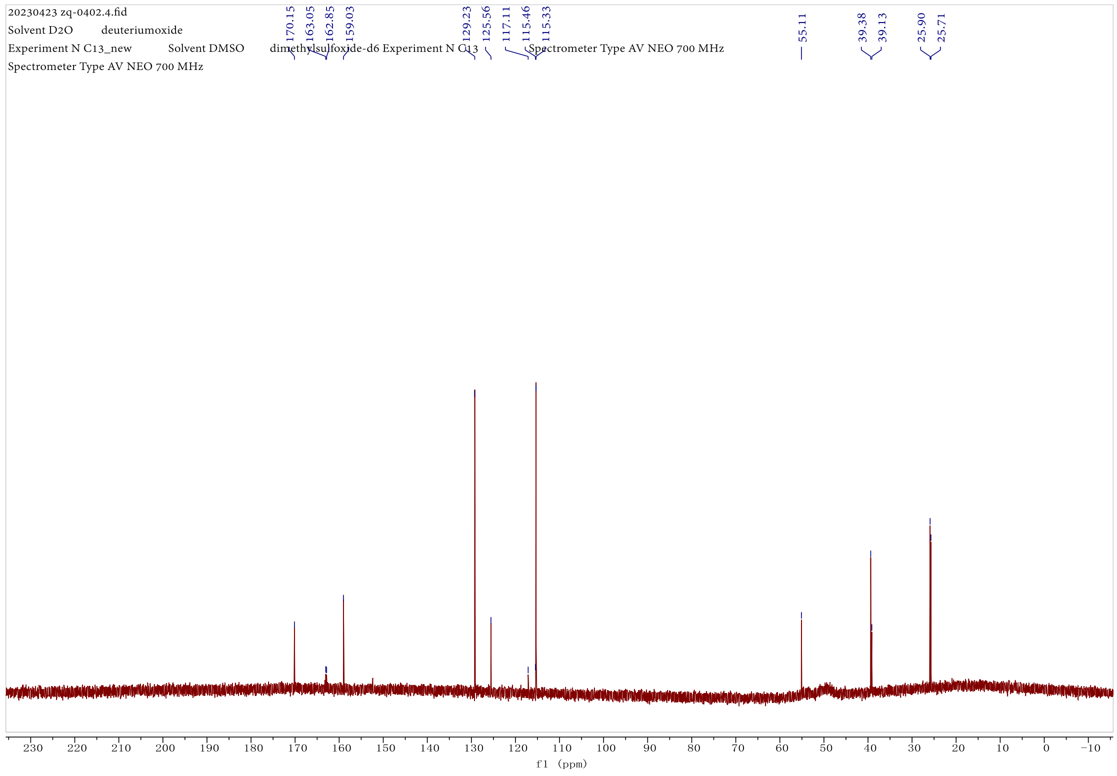

Figure S309. ^{13}C NMR spectrum of **23**.

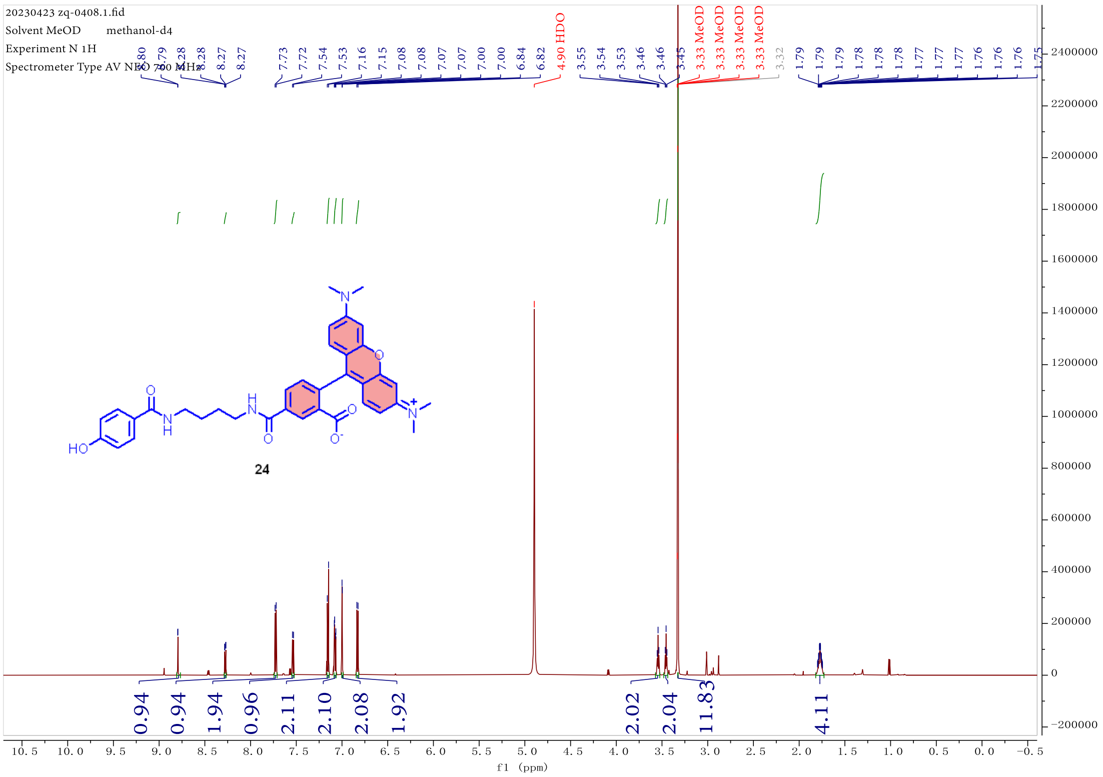

Figure S310. ^{1}H NMR spectrum of **24**.

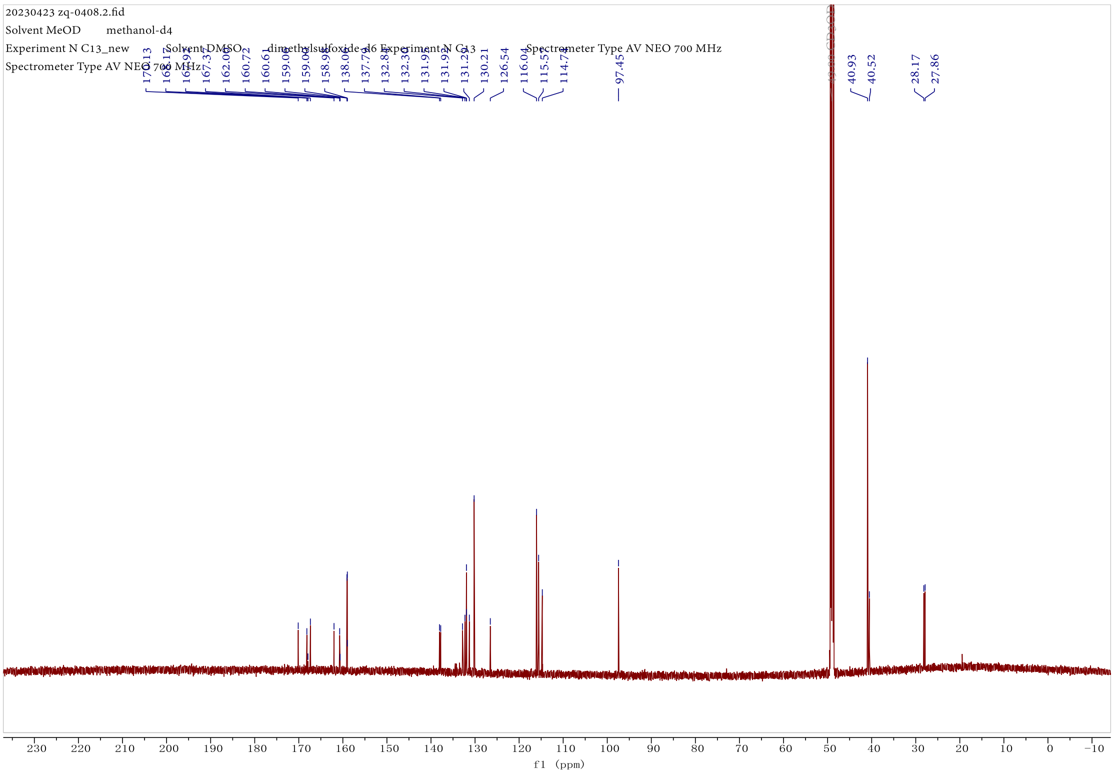

Figure S311. ^{13}C NMR spectrum of **24**.

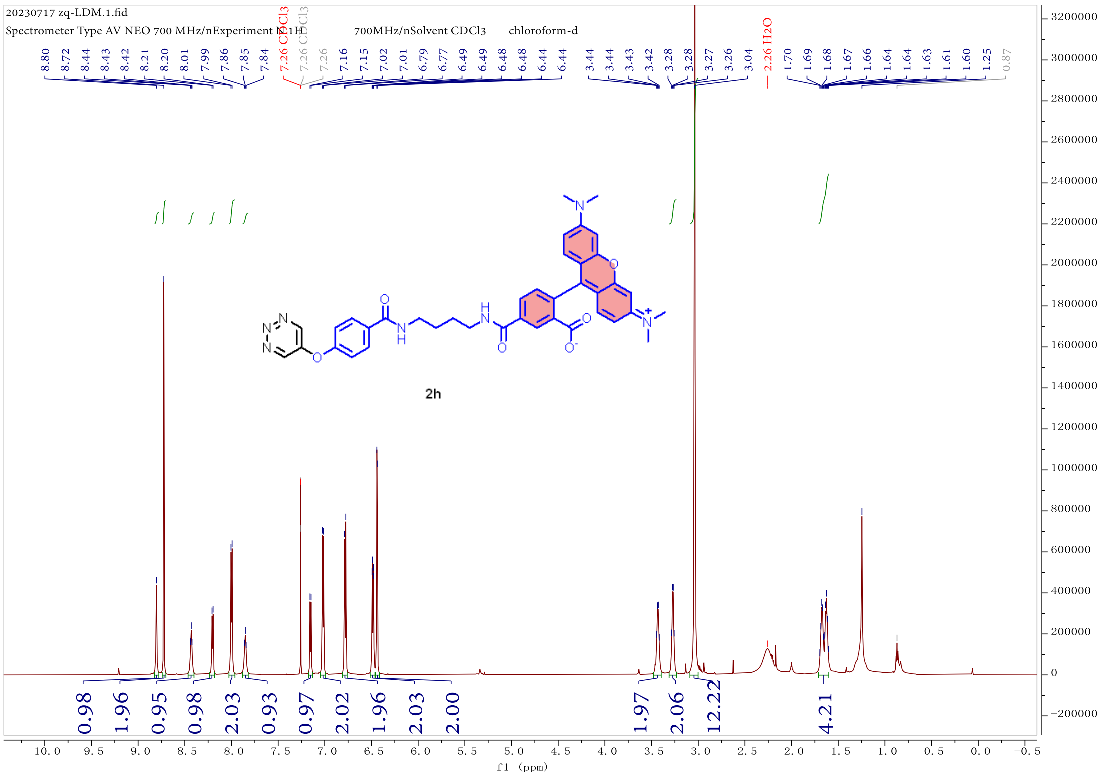

Figure S312. ^{1}H NMR spectrum of **2h**.

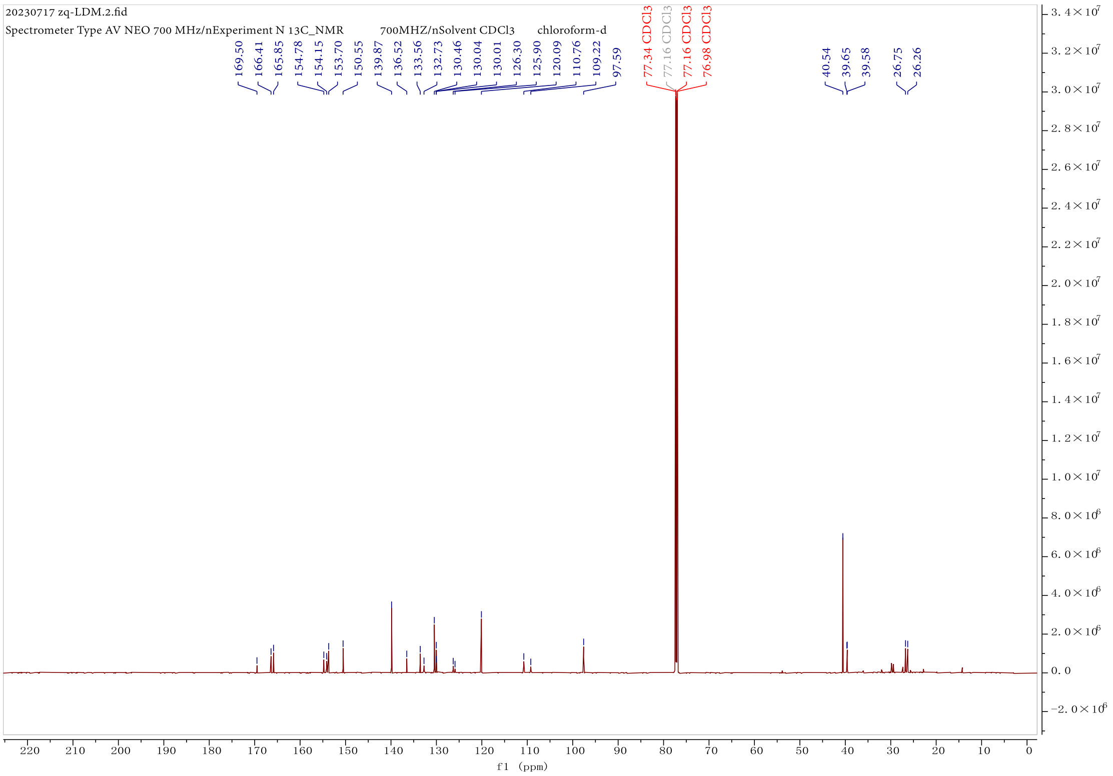

Figure S313. ^{13}C NMR spectrum of **2h**.

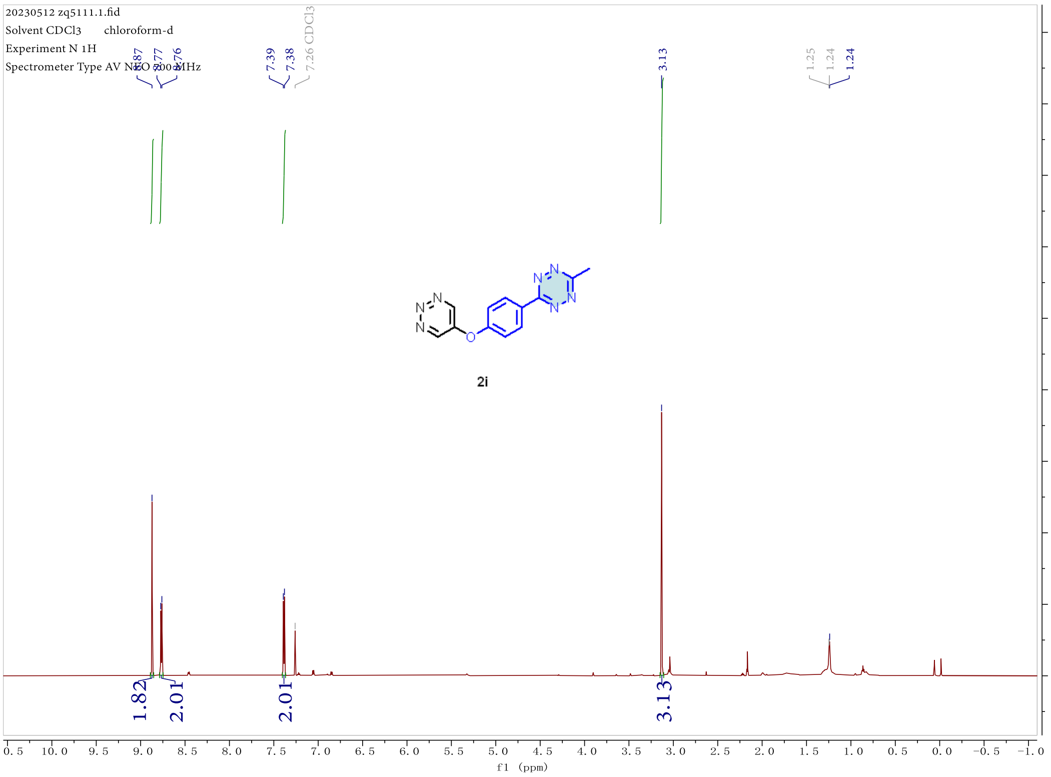

Figure S314. ^{1}H NMR spectrum of **2i**.

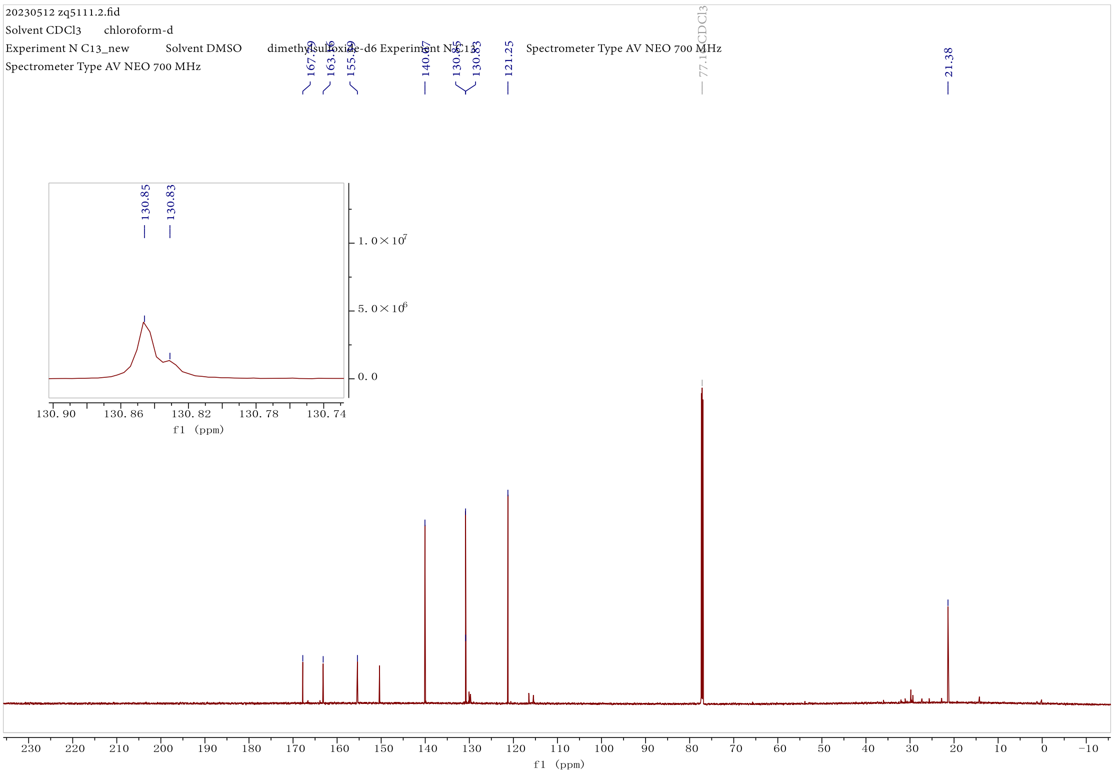

Figure S315. ^{13}C NMR spectrum of **2i**.

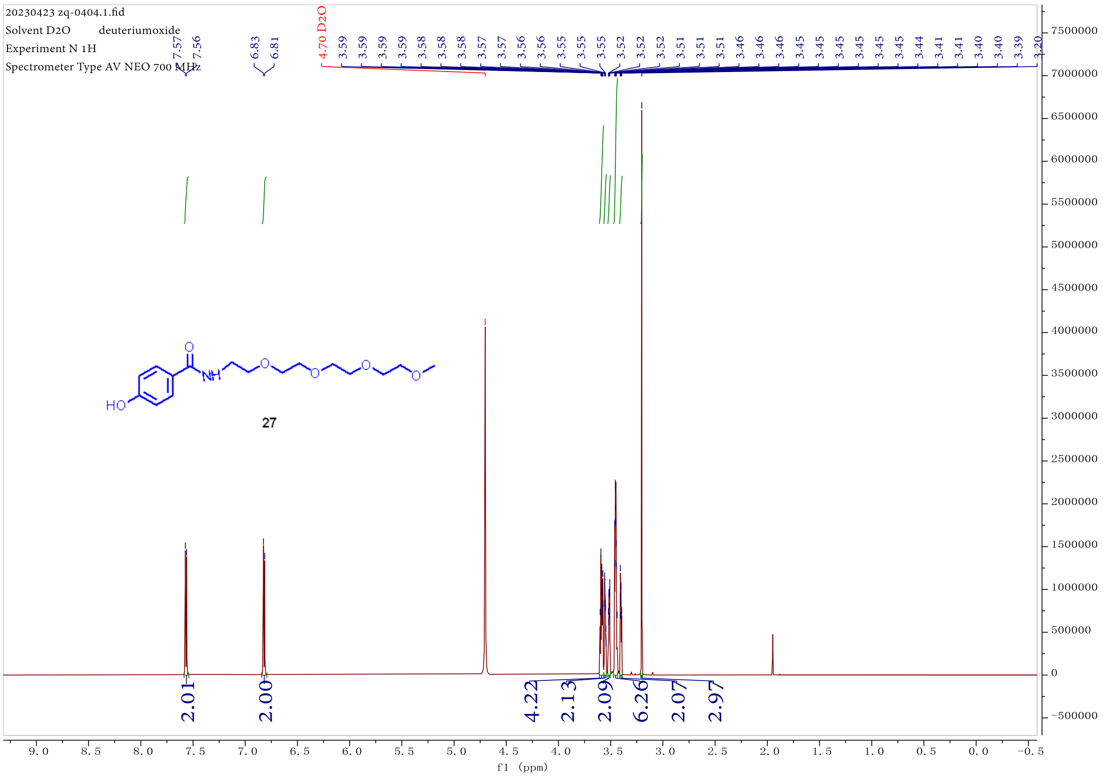

Figure S316. ^{1}H NMR spectrum of **27**.

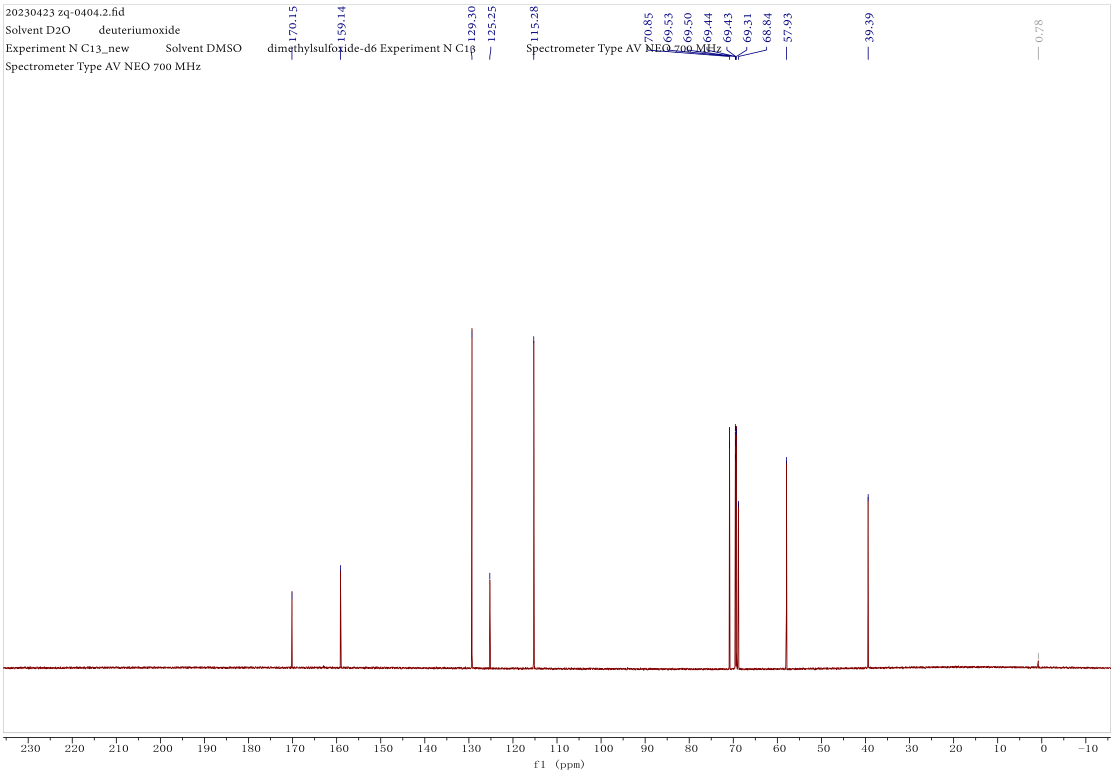

Figure S317. ^{13}C NMR spectrum of **27**.

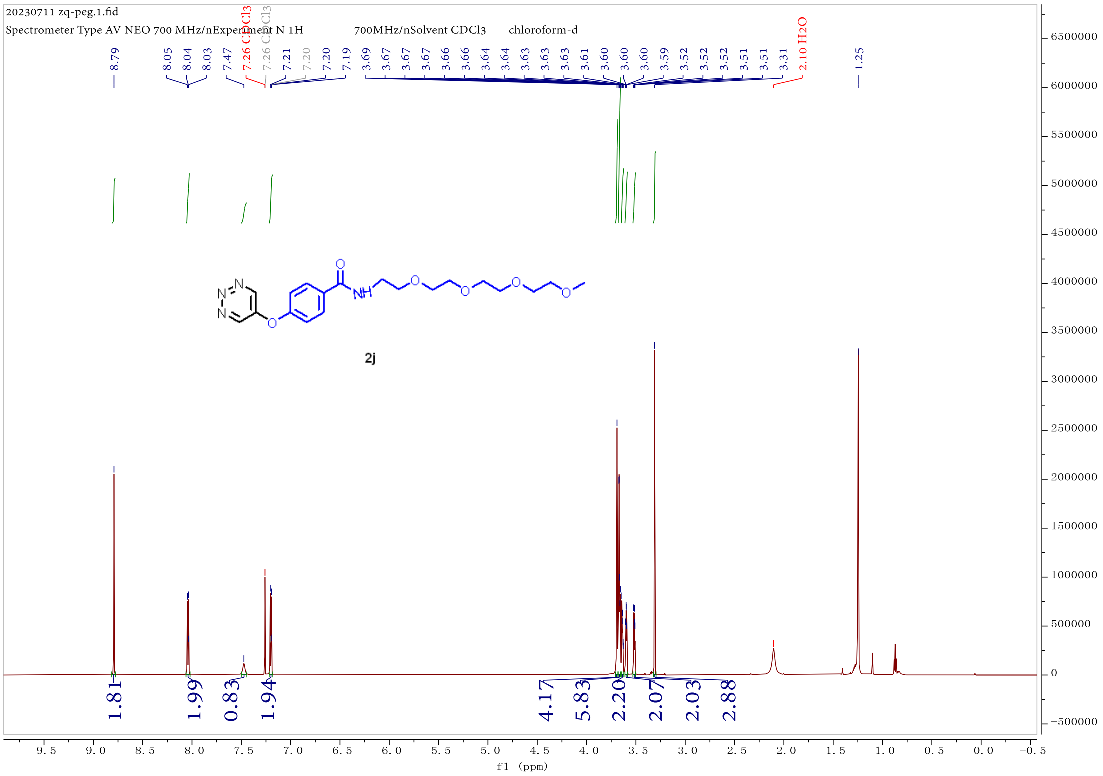

Figure S318. ^{1}H NMR spectrum of **2j**.

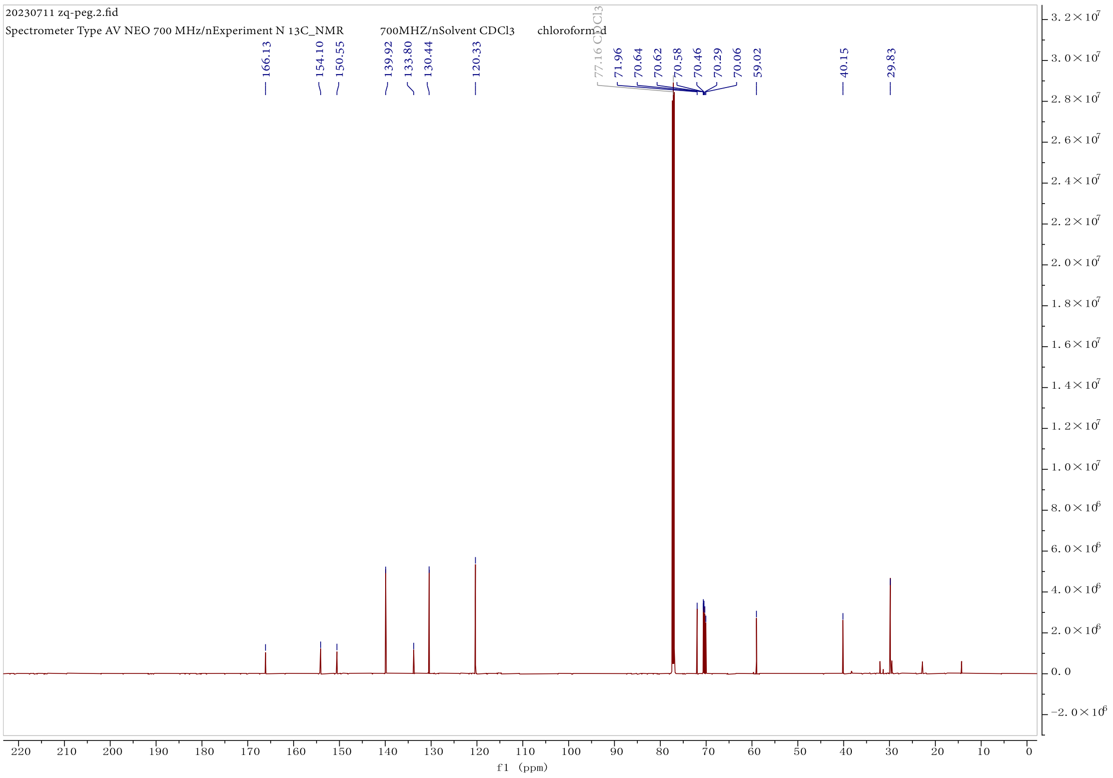

Figure S319. ^{13}C NMR spectrum of **2j**.

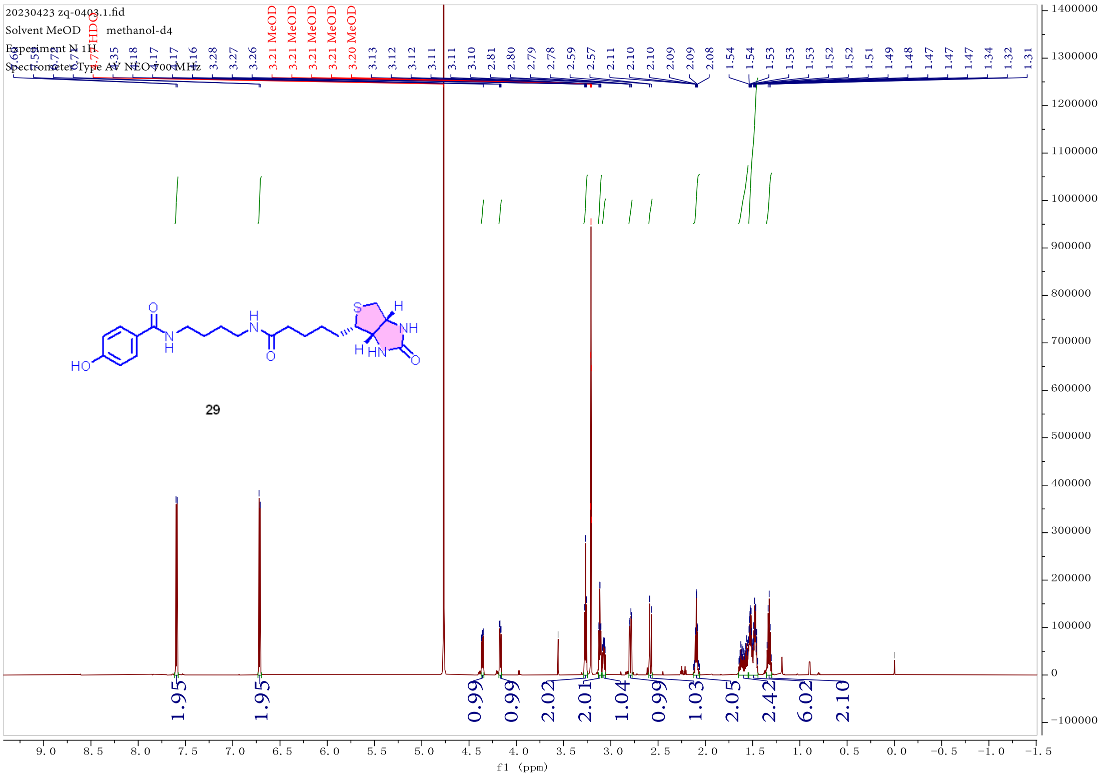

Figure S320. ^{1}H NMR spectrum of **29**.

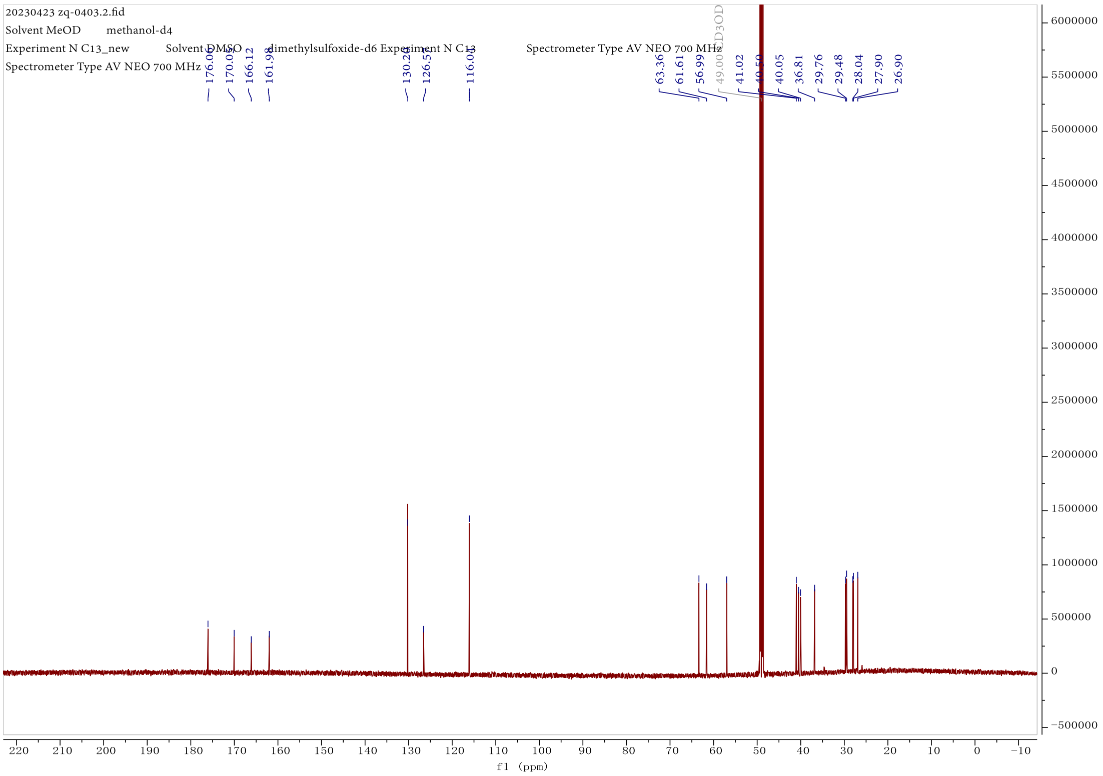

Figure S321. ^{13}C NMR spectrum of **29**.

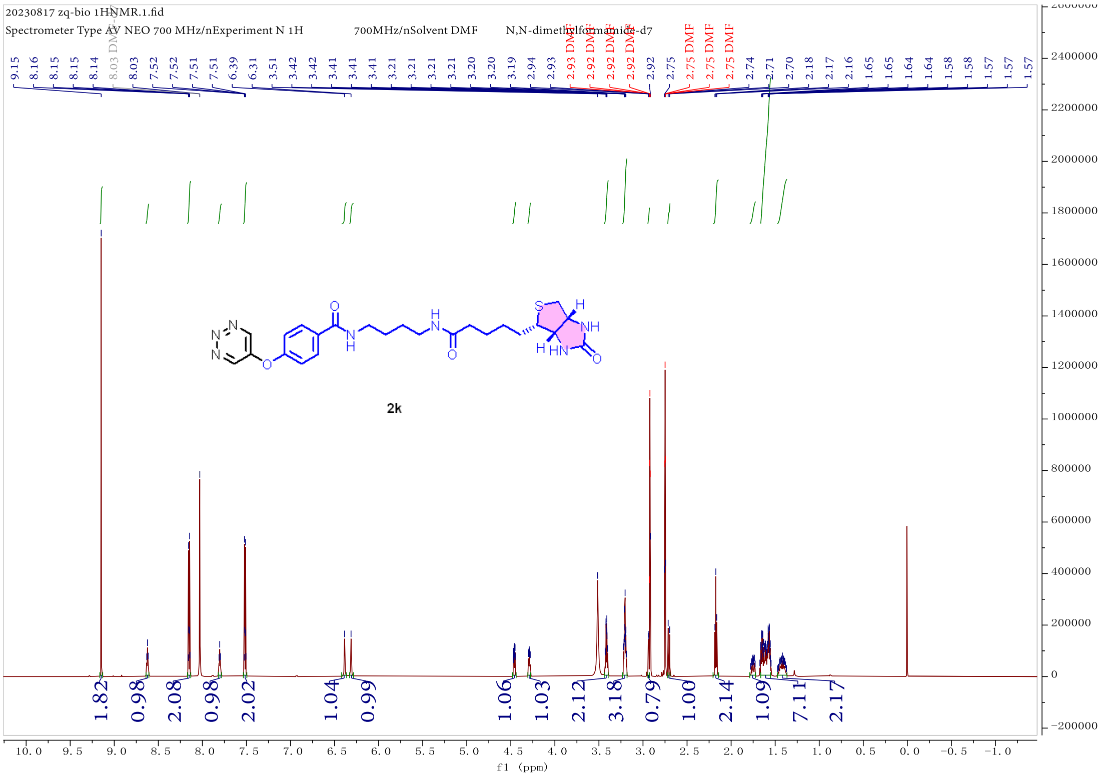

Figure S322. ^{1}H NMR spectrum of **2k**.

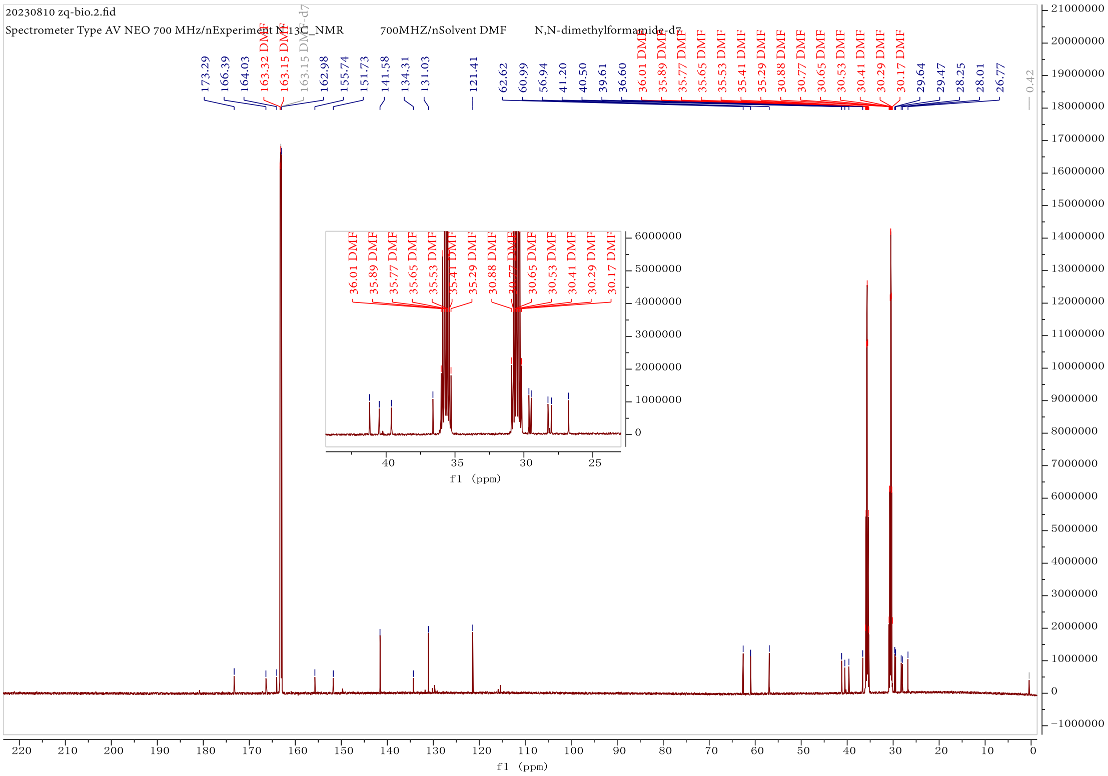

Figure S323. ^{13}C NMR spectrum of **2k**.

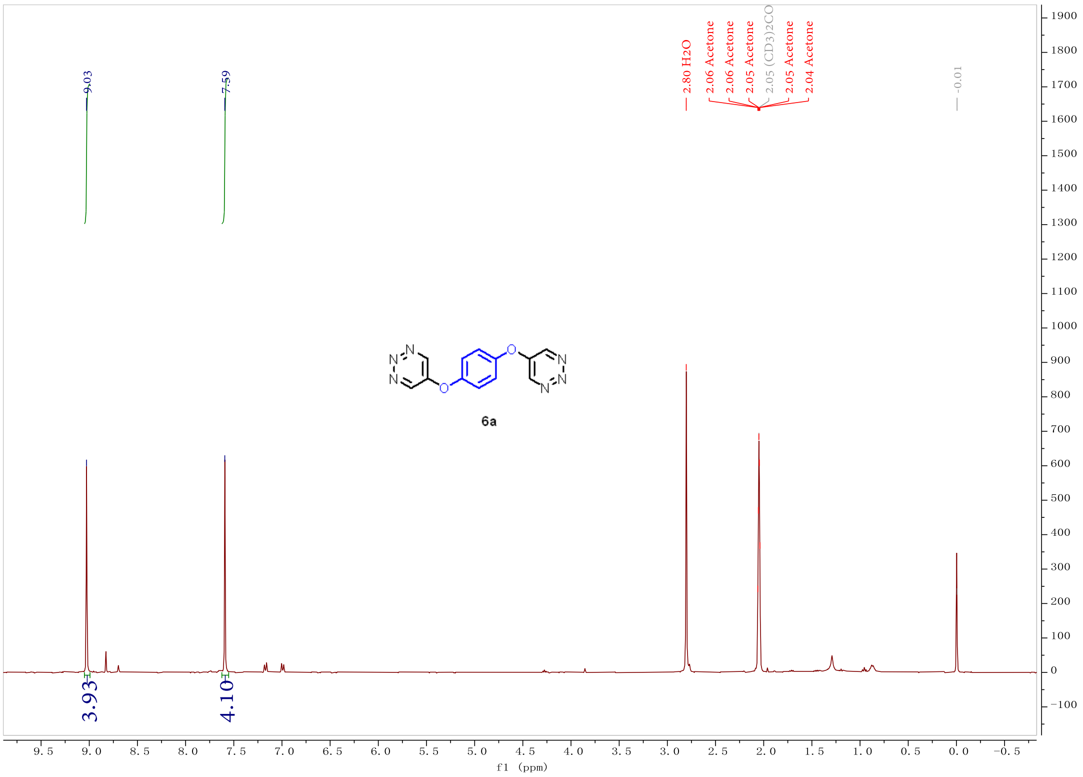

Figure S324. ^{1}H NMR spectrum of **6a**.

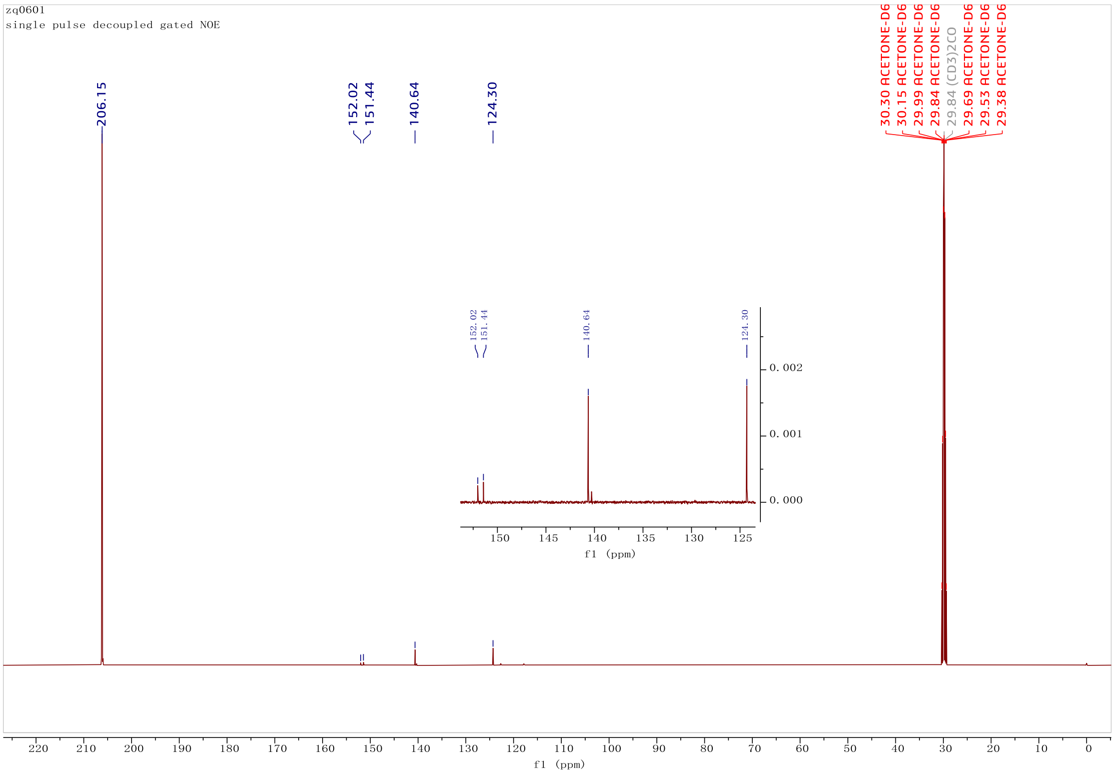

Figure S325. ^{13}C NMR spectrum of **6a**.

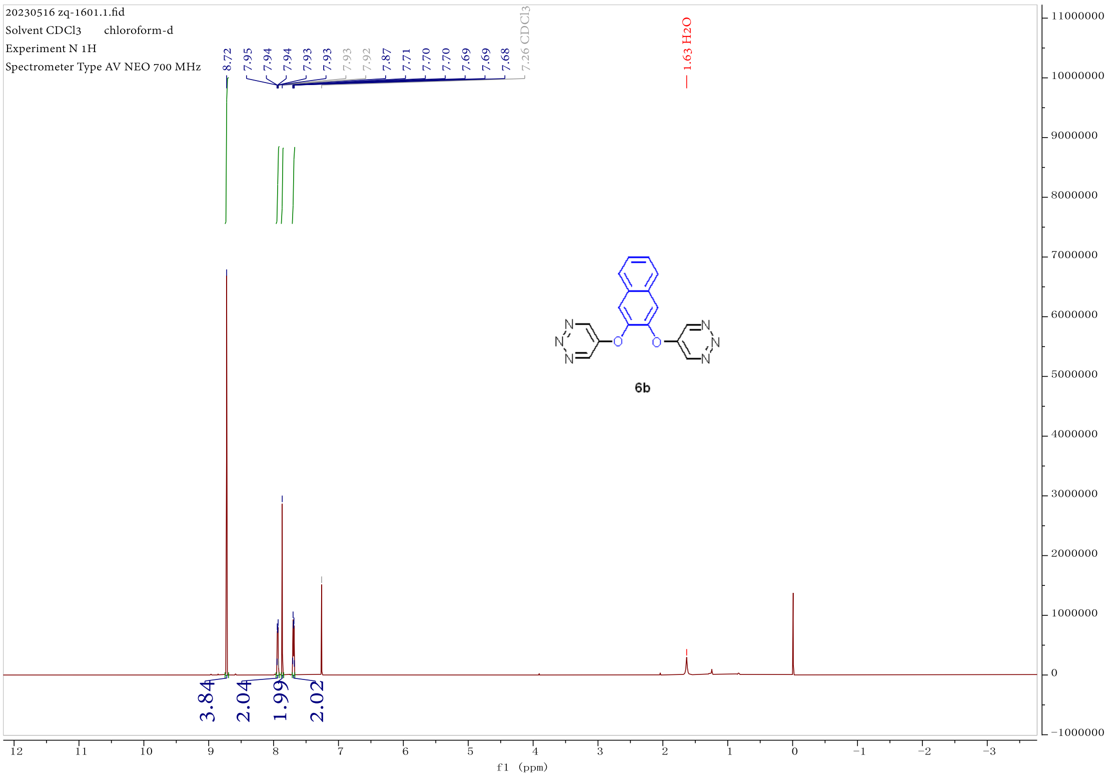

Figure S326. ^{1}H NMR spectrum of **6b**.

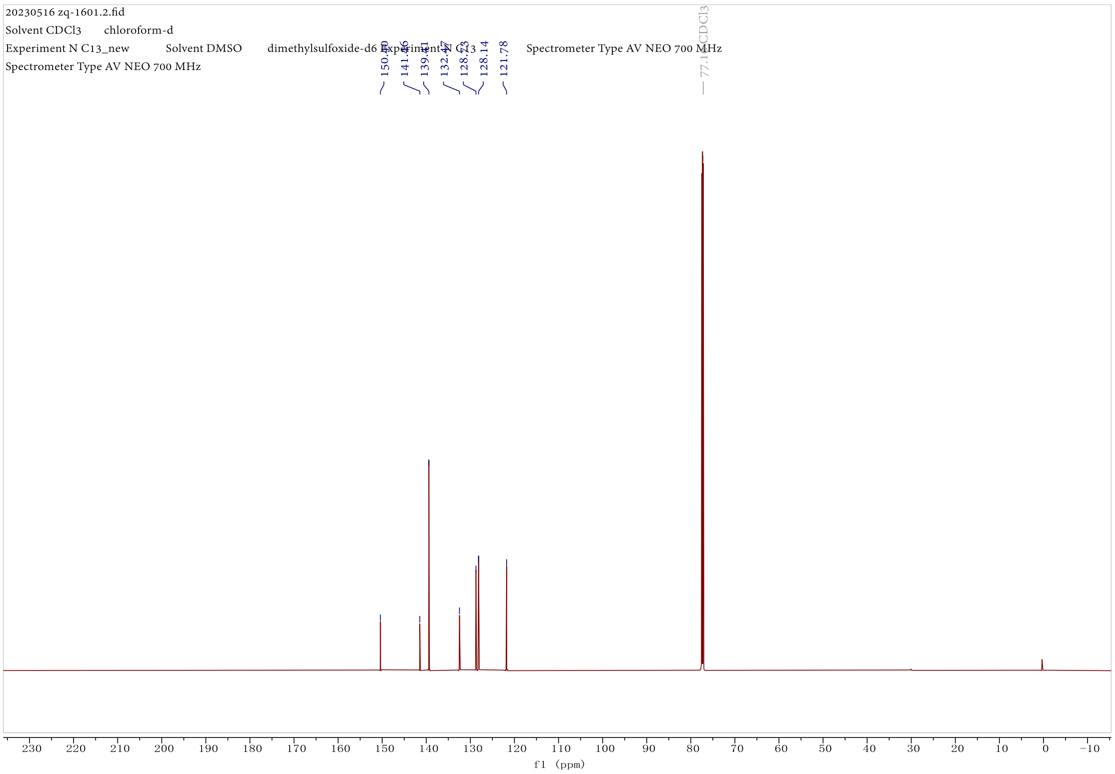

Figure S327. ^{13}C NMR spectrum of **6b**.

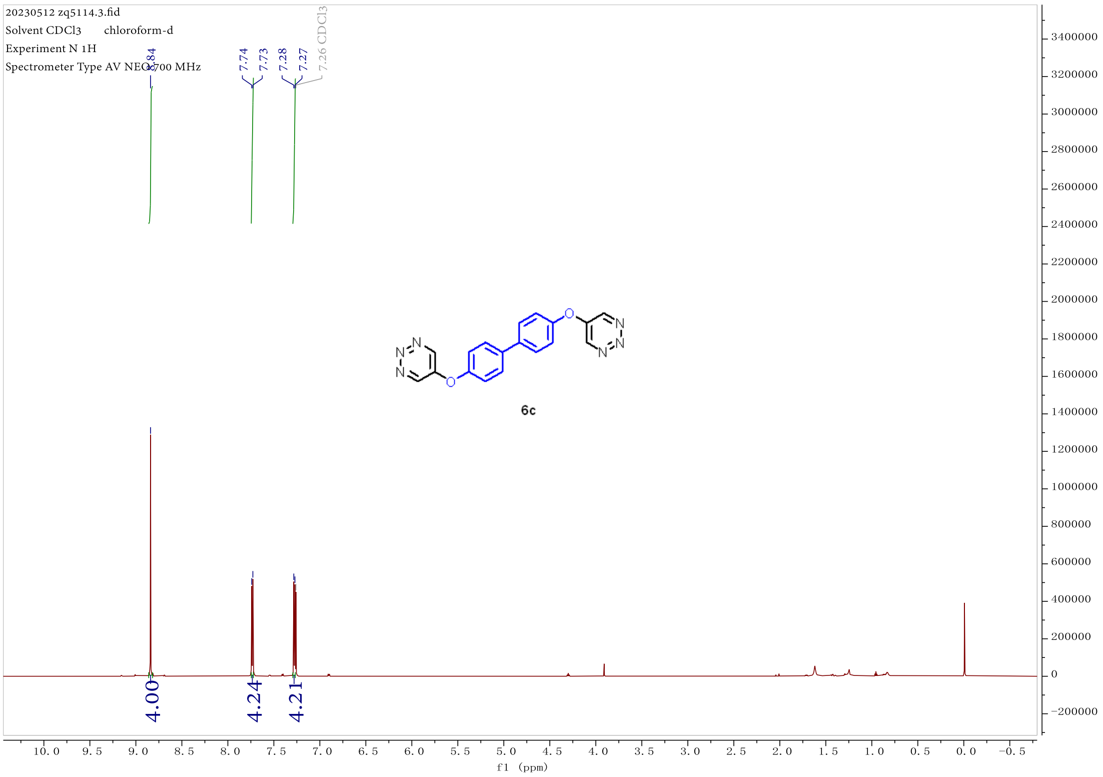

Figure S328. ^{1}H NMR spectrum of **6c.**

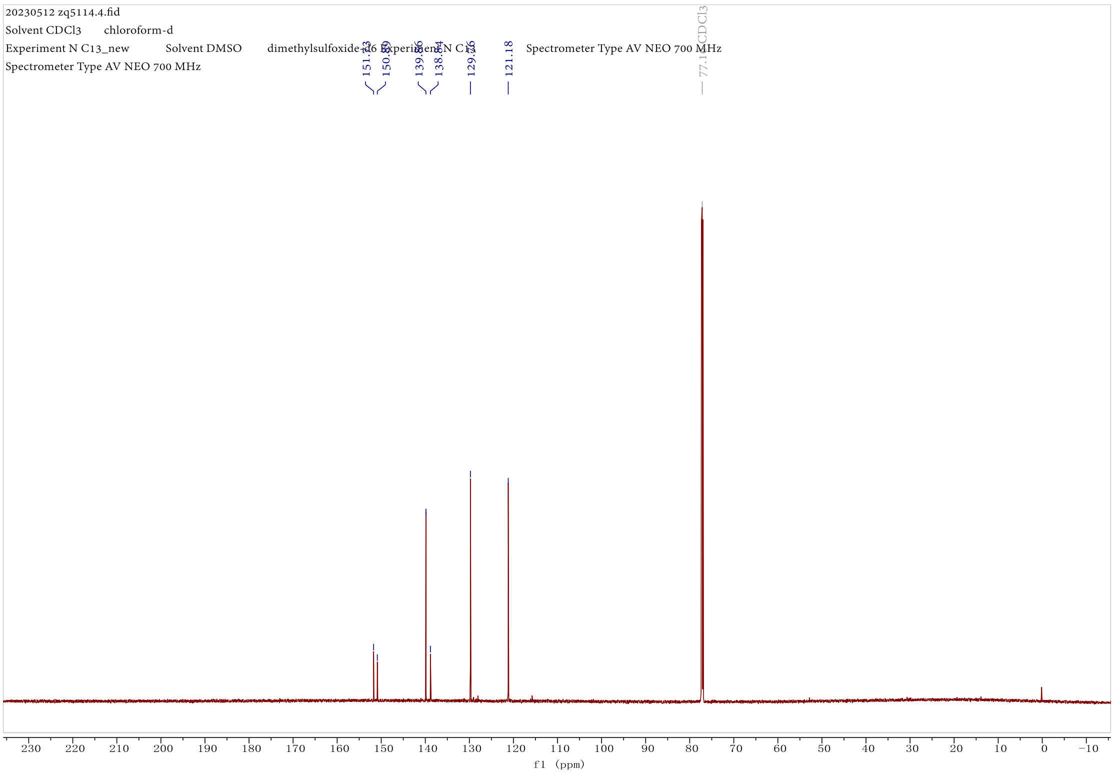

Figure S329. ^{13}C NMR spectrum of **6c.**

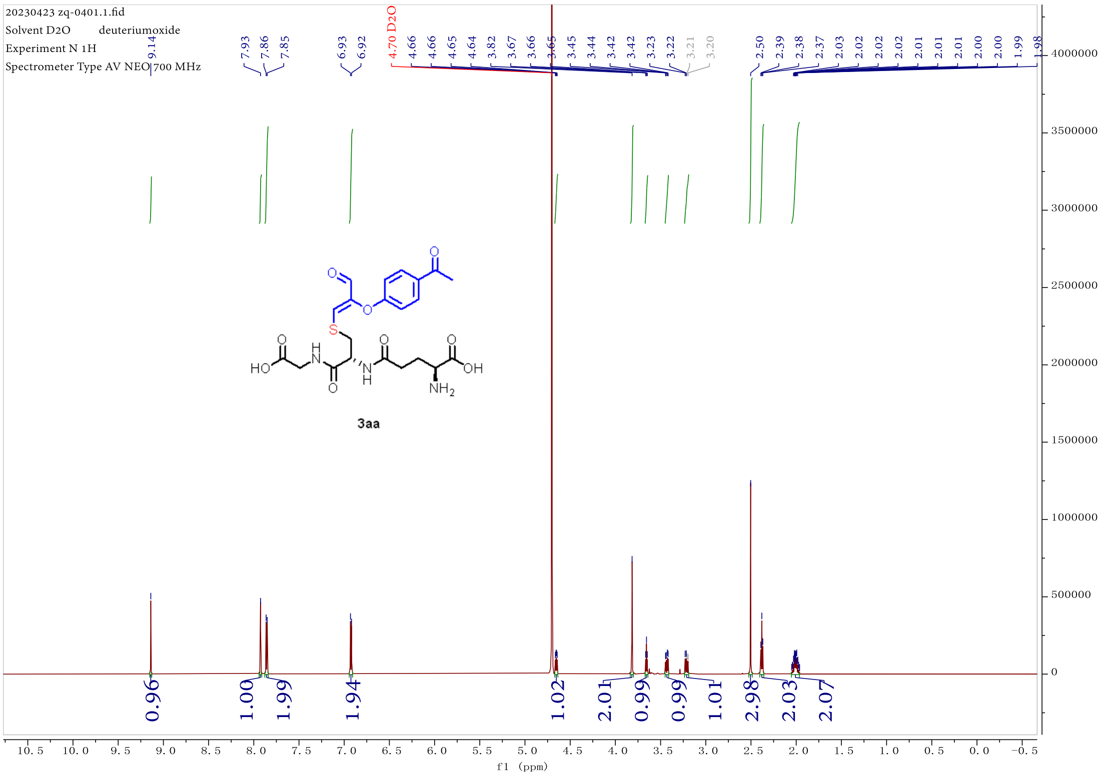

Figure S330. ^{1}H NMR spectrum of **3aa**.

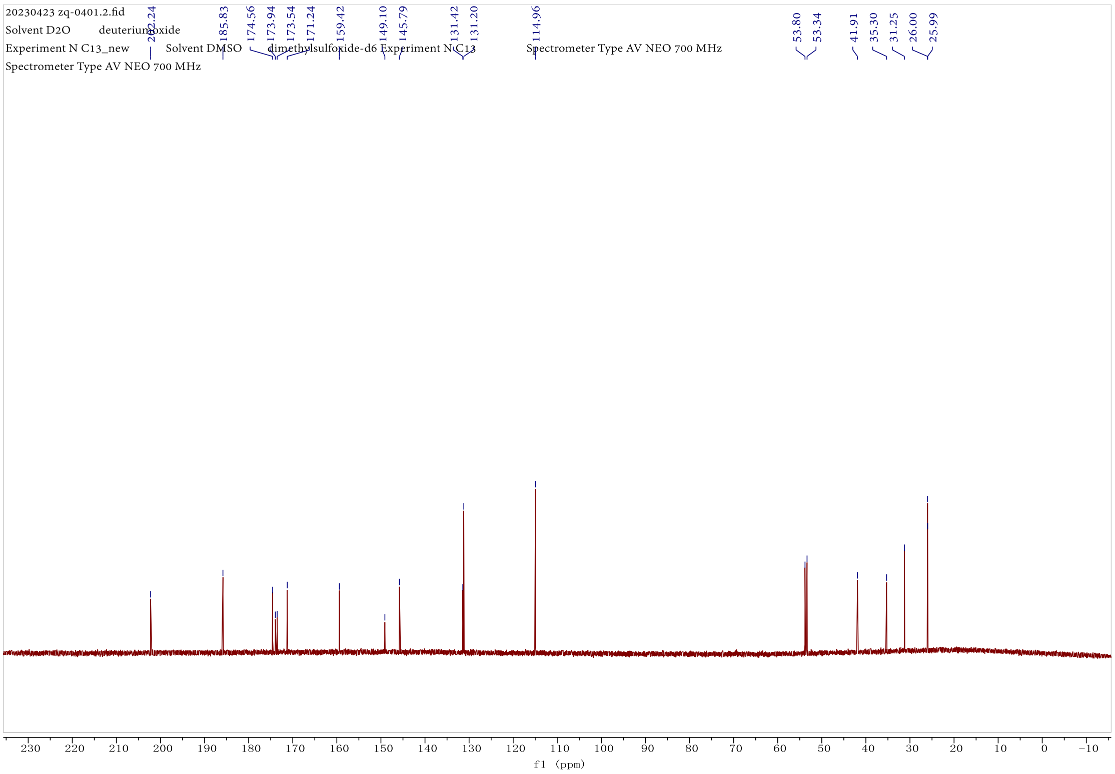

Figure S331. ^{13}C NMR spectrum of **3aa**.

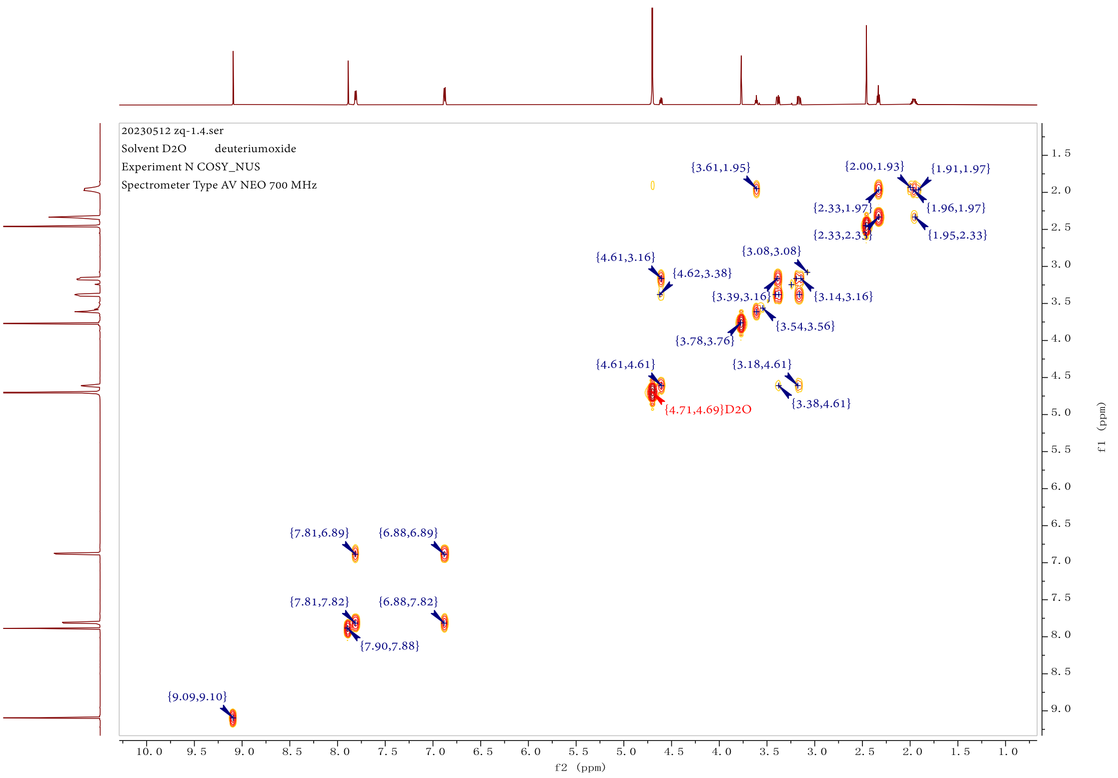

Figure S332. ^{1}H-^{1}H COSY spectrum of **3aa**.

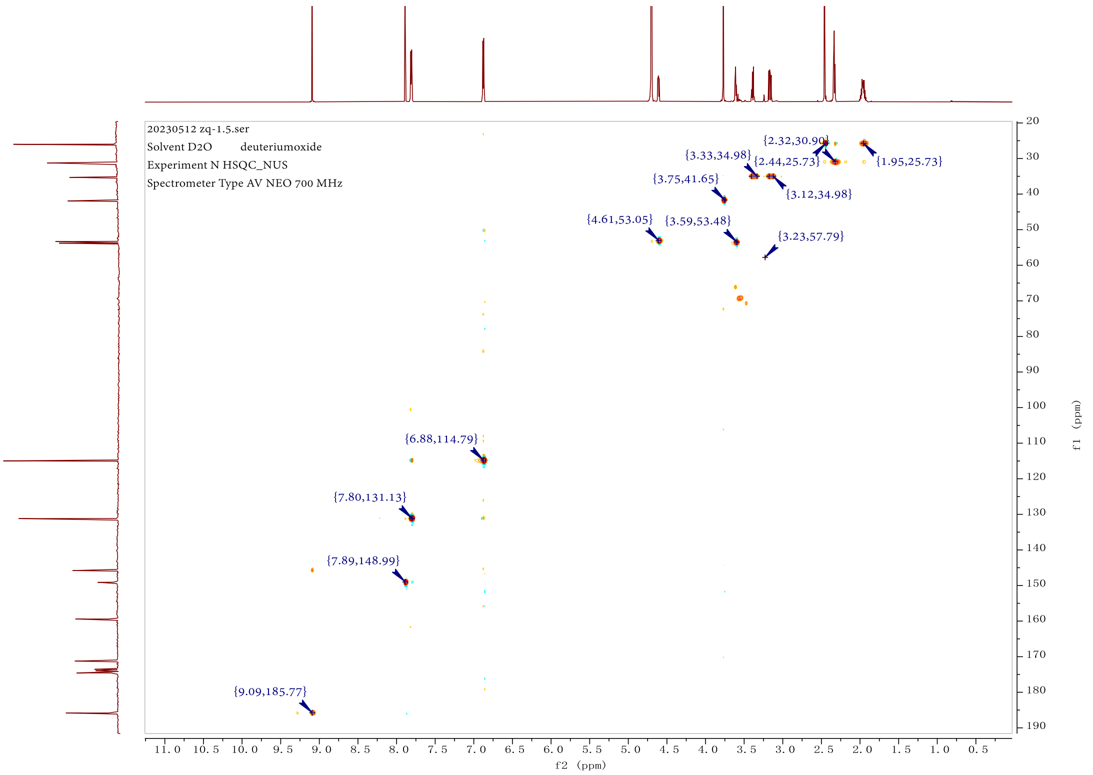

Figure S333. HSQC spectrum of **3aa**.

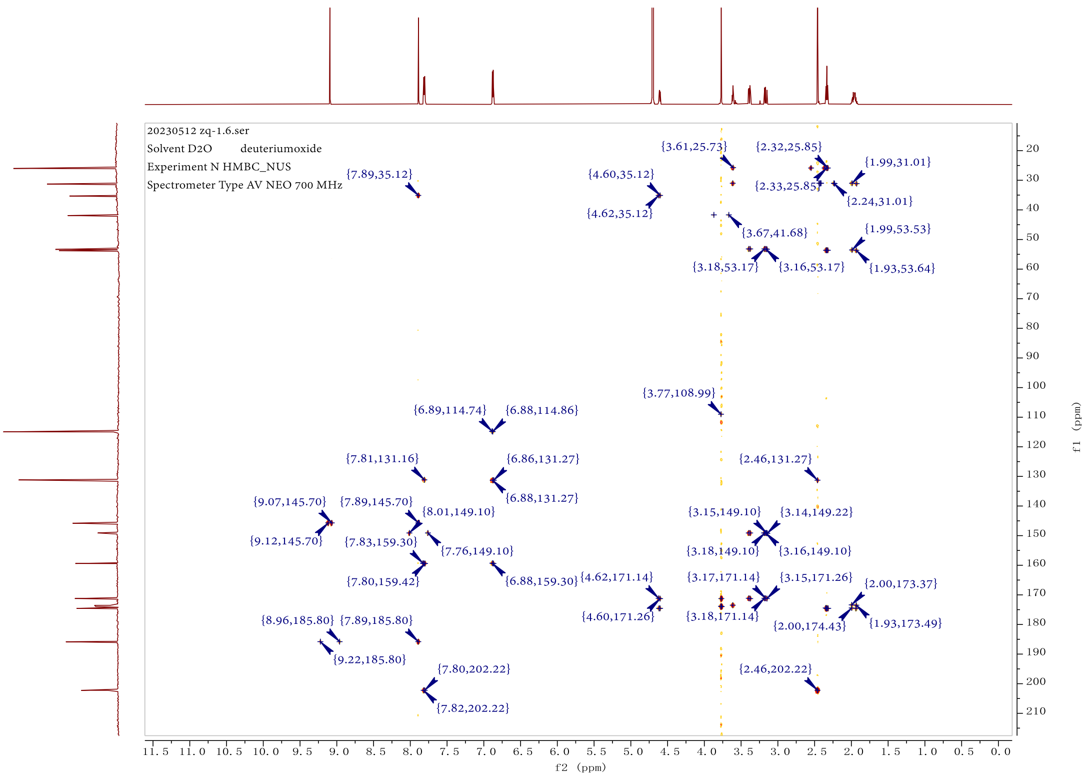

Figure S334. HMBC spectrum of **3aa**.

References

[1] R. Tessier, J. Ceballos, N. Guidotti, R. Simonet-Davin, B. Fierz, J. Waser, *Chem* **2019**, *5*, 2243–2263.

[2] a) H. Luo, Y. Li, Y. Zhang, Q. Lu, Q. An, M. Xu, S. Li, J. Li, B. Li, *J. Org. Chem.* **2022**, *87*, 2590–2600; b) A. Ohsawa, H. Arai, H. Ohnishi, T. Itoh, T. Kaihoh, M. Okada, H. Igeta, *J. Org. Chem.* **1985**, *50*, 5520-5523.

[3] G. Voerman, S. Cavalli, G. A. van der Marel, W. Pfleiderer, J. H. van Boom, D. V. Filippov, *J. Nat. Prod.* **2005**, *68*, 938-941.

[4] a) H. Luo, Q. Lu, M. Xu, M. Gu, B. Li, *Synthesis* **2022**, *54*, 4472–4480; b) R. E. Quiñones, C. M. Glinkerman, K. Zhu, D. L. Boger, *Org. Lett.* **2017**, *19*, 3568–3571; c) S. J. Siegl, M. Vrabel, *Eur. J. Org. Chem.* **2018**, *37*, 5081–5085.

Author Contributions

Q.Z. has been involved in: Conceptualization (lead), Data curation (lead), Formal Analysis (lead), Investigation (lead), Methodology (lead), Validation (lead), Visualization (lead), Writing – original draft (lead) and Writing – review & editing (lead). R.W. and W.S. has been involved in: Mentoring of Q.Z. (lead), Funding acquisition (lead), Project administration (lead), Resources (lead) and Writing – review & editing (supporting). Y.L. and G.B. has been involved in: Investigation (equal) and Writing – review & editing (supporting). X.L., L.C., Z.H., X.S., and R.E. has been involved in: Methodology (supporting), and Validation (supporting). P.W., Y.S., and H.L. has been involved in: Mentoring of Q.Z. (supporting), Funding acquisition (supporting), Project administration (supporting).
